# 1,2,3-Benzotriazine Synthesis by Heterocyclization of *p*-Tosylmethyl Isocyanide Derivatives

**DOI:** 10.1021/acs.joc.3c01675

**Published:** 2023-09-18

**Authors:** Francisco Maqueda-Zelaya, José Luis Aceña, Estíbaliz Merino, Juan J. Vaquero, David Sucunza

**Affiliations:** Departamento de Química Orgánica y Química Inorgánica, Instituto de Investigación Química “Andrés M. del Río” (IQAR), Universidad de Alcalá, IRYCIS, 28805, Alcalá deHenares, Madrid, Spain

## Abstract

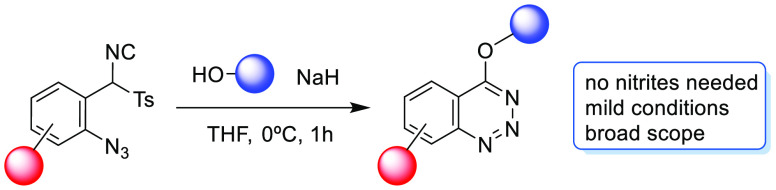

An efficient methodology to form 4-alkoxy- and 4-aryloxybenzo[*d*][1,2,3]triazines via an intramolecular heterocyclization of 1-azido-2-[isocyano(*p*-tosyl)methyl]benzenes under basic conditions has been developed. DFT calculations have been performed to further understand the mechanism of this heterocyclization, which occurs in good to excellent yields with a broad scope.

## Introduction

**Figure 1:**
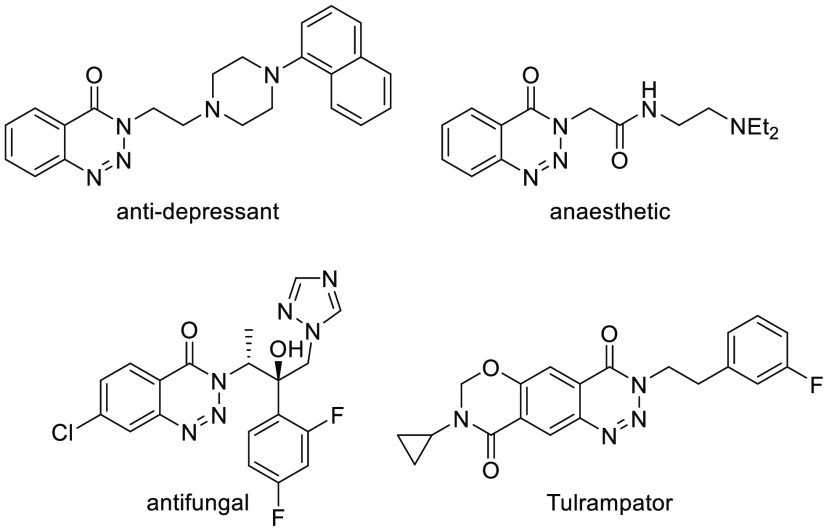
Examples of biologically active benzotriazines.

1,2,3-Benzotriazines are important *N*-heterocycles that exhibit a wide range of biological activities.^1^ For instance, this heterocyclic scaffold has been reported in derivatives (Figure 1) that act as antidepressants (binding to 5-HT_1A_ receptors),^2^ anesthetics,^3^ antifungal agents,^4^ and antihypertensives,^5^ among others. Moreover, the AMPAR-positive allosteric modulator (AMPA-PAM) tulrampator is in clinical trials as a possible treatment for Alzheimer’s disease, dementia, and mild cognitive impairment.^6^

In addition, compounds of this kind have also been described as pesticides,^7^ dyes,^8^ recording and imaging materials,^1a^ and synthetic intermediates. Thus, this scaffold is a versatile building block for the synthesis of *ortho*-arylated,^9^ alkenylated,^10^ and hydroxylated^11^ benzamides, as well as various azaheterocycles^12^ via metal-catalyzed denitrogenative transannulation reactions.

1,2,3-Benzotriazines are typically prepared via the diazotization of 2-aminobenzamides in the presence of NaNO_2_ and a strong acid,^1^ although the harsh acidic conditions needed for this method and the use of sodium nitrite can lead to the release of toxic nitrogen oxides and the potential formation of nitrosamines, the latter of which are especially undesirable in drugs, even as traces at ppb levels.^13^ Consequently, milder reagents and reaction conditions have been reported recently for this transformation, despite most of them still requiring the use of nitrites or similar *N*-atom donor reagents.^14^ As such, the search for different and efficient approaches to these molecules remains of great significance.

*p*-Tosylmethyl isocyanide (TosMIC) and its derivatives are densely functionalized building blocks with three groups that can engage in a multitude of reactions: the isocyanide moiety, acidic protons in the α-position, and the tosyl group.^15^ As part of a research program aimed at expanding TosMIC chemistry to the preparation of six-membered heterocycles,^16^ our group has recently developed a new method for the synthesis of isoquinolines and γ-carbolines via a heterocyclization that takes advantage of the capacity of isocyanides to act both as nucleophiles and electrophiles.^17^

Herein we report the use of these reagents to synthesize a different azaheterocyclic scaffold—1,2,3-benzotriazine—that occurs thanks to another of their remarkable properties: their acidic α-carbon atom and the tendency of the isocyanide and tosyl groups to act as a leaving group. This new methodology involves an intramolecular heterocyclization of 1-azido-2-[isocyano(*p*-tosyl)methyl]benzenes under basic conditions, followed by the insertion of a phenol or an alkyl alcohol.

## Results and Discussion

**Scheme 1:**
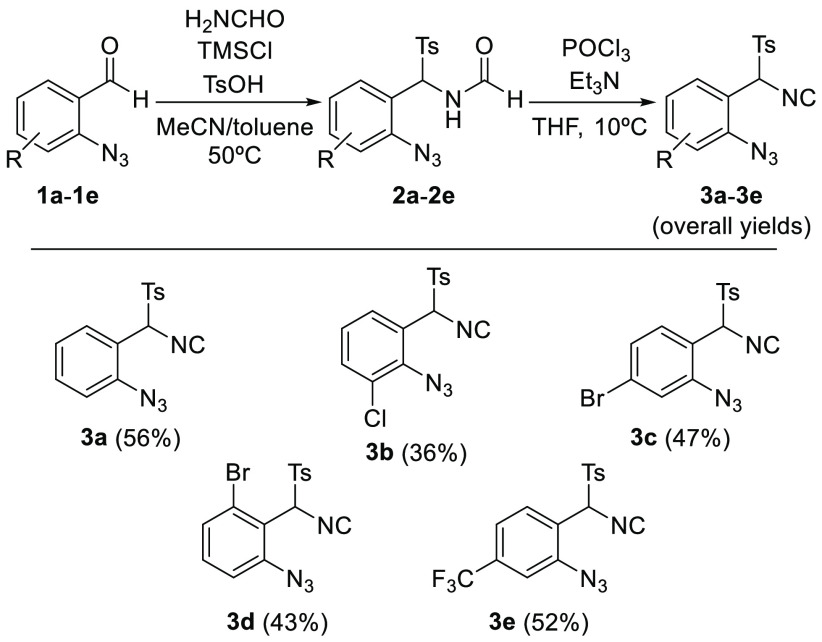
Synthesis of TosMIC Derivatives **3a**–**3e**

In order to test the feasibility of the proposed heterocyclization, 1-azido-2-[isocyano(*p*-tosyl)methyl]benzene (**3a**, R = H) was prepared in two steps: treatment of commercially available 2-azidobenzaldehyde (**1a**) with formamide, chlorotrimethylsilane, and *p*-toluenesulfonic acid to form *N*-(α-tosylbenzyl)formamide **2a**, and dehydration of this intermediate using phosphorus oxychloride and triethylamine (Scheme 1).^18^ In a similar manner, we also synthesized the TosMIC derivatives **3b**–**3e** in good overall yields (36–52% in two steps), although an extra initial reaction to prepare the 2-azidobenzaldehydes **1b**–**1e** is necessary in those cases. Compounds **3b**, **3c**, and **3d** contain a halogen in different positions of the aryl moiety, and **3e** contains a trifluoromethyl group.

**Scheme 2:**
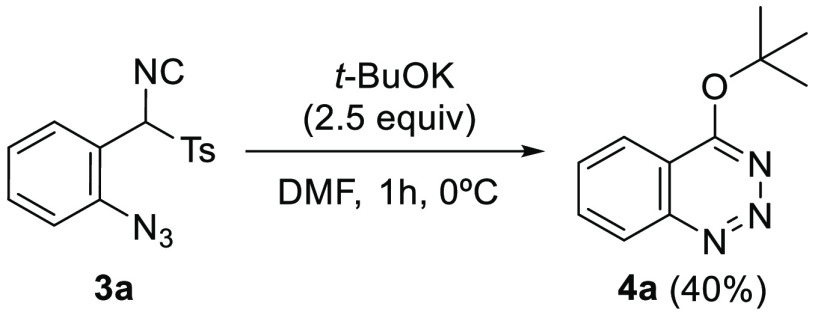
Synthesis of 4-*tert*-Butyloxybenzotriazine **4a**

Our first attempt at the proposed heterocyclization involved the treatment of isonitrile **3a** with a bulky base, such as *t*-BuOK in a polar solvent such as DMF (Scheme 2). The idea was to force α-deprotonation of the TosMIC moiety, expecting a subsequent nucleophilic attack of the anion formed at the electrophilic azide^19^ to carry out the desired cyclization and finally form the benzo[*d*][1,2,3]triazin-4(3*H*)-one. Interestingly, despite using a non-nucleophilic base in the process, the main product achieved in the reaction was 4-(*tert*-butyloxy)benzotriazine (**4a**) in 40% yield.

Taking this result into account, we speculated that a more nucleophilic alkoxide could improve the result of the cyclization, and for that reason, we tried the same reaction with an allyloxy anion. As we anticipated, the treatment of isonitrile **3a** with three equivalents of sodium allyloxide, prepared *in situ* from allylic alcohol and NaH, in DMF as solvent, afforded 4-(allyloxy)benzotriazine (**4b**) in a better yield (54%, Table 1, entry 1). The use of other polar solvents led to variable yields, and THF was found to offer the best result of all those employed (entries 2–4). The temperature and concentration of the starting material in the reaction mixture were also studied (entries 5–8), and the best conditions found were 0.04 M at 0 °C (entry 6). Thus, benzotriazine **4b** was isolated in an excellent yield (88%, 93% when calculated by ^1^H NMR using ethylene carbonate as the internal standard) after 1 h under the optimized conditions.

**Table 1:**
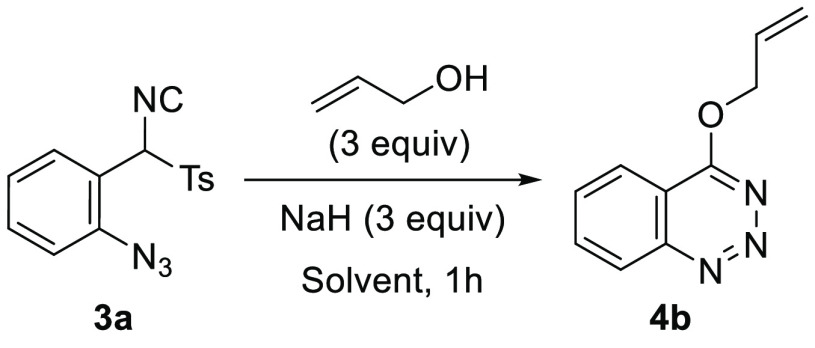
Optimization of the Formation of **4b**

entry	solvent	conc [M]	*T* (°C)	yield (%)^a^
1	DMF	0.08	0	54
2	MeCN	0.08	0	75
3	DMSO	0.08	rt	7
4	THF	0.08	0	78
5	THF	0.08	–20	66
6	THF	0.04	0	93
7	THF	0.04	–20	63
8	THF	0.02	0	82

a Calculated by ^1^H NMR employing ethylene carbonate as internal standard.

**Scheme 3:**
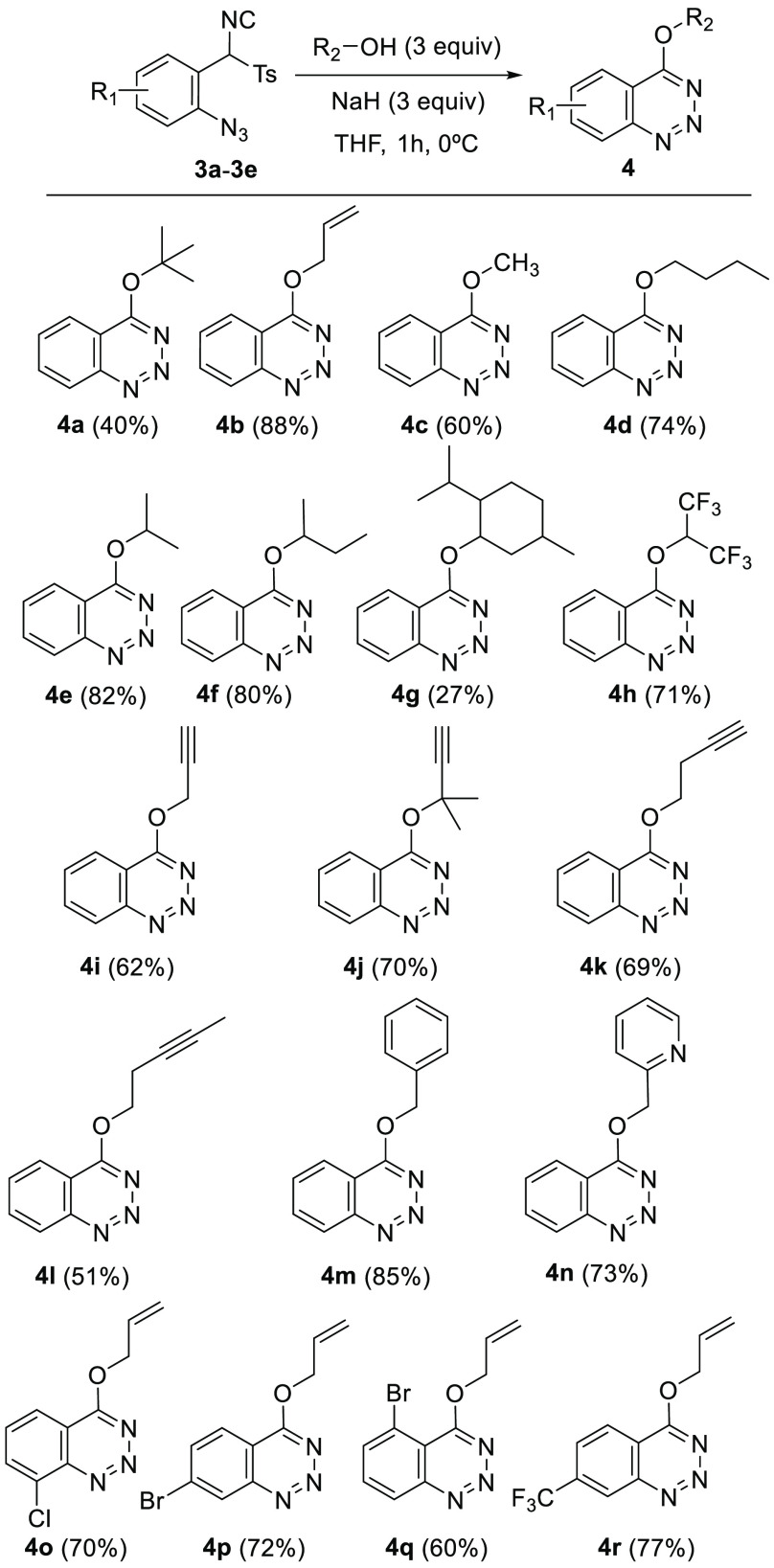
Substrate Scope for 4-Alkyloxybenzotriazines **4**

With the optimized conditions in hand, the scope of the heterocyclization was examined (Scheme 3). The procedure proved successful with a variety of sodium alkoxides prepared *in situ* from an alcohol and NaH. The list includes primary alcohols such as methyl, butyl, allyl, propargyl, benzyl, and pyridin-2-ylmethyl alcohol; secondary alcohols such as isopropyl, *s*-butyl, 1,1,1,3,3,3-hexafluoropropan-2-yl alcohol, and dl-menthol; and tertiary alcohols such as *tert*-butyl and 2-methylbut-3-yn-2-yl alcohol. Additionally, TosMIC derivatives **3b**–**3e** were tested, and 4-(allyloxy)benzotriazines **4o**–**4r** were obtained in high yields from sodium allyloxide prepared *in situ*. In general, no decrease in the reaction yields was detected when bulkier alcohols were employed.

After demonstrating the feasibility of synthesizing 4-(alkyloxy)benzotriazines **4** from TosMIC derivatives, we considered the possibility of expanding the scope of these cyclizations to the use of phenols. Thus, isonitrile **3a** was treated with 3 equiv of sodium phenoxide, prepared *in situ* from phenol and NaH, in THF as solvent at 0 °C for 1 h to afford 4-phenoxybenzotriazine (**5a**) in 79% yield. Although this was a good result, we explored other reaction conditions in an attempt to improve the yield achieved (Table 2).

**Table 2:**
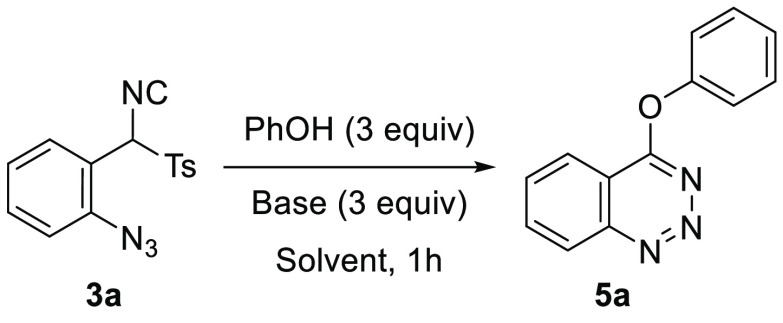
Optimization of the Formation of **5a**

entry	base	solvent	*T* (°C)	yield (%)^a^
1	NaH	THF	0	79
2	NaH	DMF	0	54
3	Na_2_CO_3_	THF	0	0^b^
4	Na_2_CO_3_	DMF	0	72
5^c^	Na_2_CO_3_	DMF	0	74
6^d^	Na_2_CO_3_	DMF	0	55
7^c^	Na_2_CO_3_	DMF	rt	66
8^c^	Na_2_CO_3_	DMF	–20	38
9^c^,^e^	Na_2_CO_3_	DMF	0	50
10^c^,^f^	Na_2_CO_3_	DMF	0	23
11	Na_2_CO_3_	MeCN	0	23
12	Na_2_CO_3_	NMP	0	42
13	Na_2_CO_3_	DMSO	0	59
14	K_2_CO_3_	DMF	0	65
15	Cs_2_CO_3_	DMF	0	61
16	K_3_PO_4_	DMF	0	72
17	DBU	DMF	0	52
18	DABCO	DMF	0	0^b^
19	DBN	DMF	0	48

a Calculated by ^1^H NMR employing ethylene carbonate as internal standard.

b Starting material recovered.

c 0.08 M THF solution of the starting material.

d 0.16 M THF solution of the starting material.

e 2 equiv of phenol and base added.

f 1 equiv of phenol and base added.

First, we tested a weaker base, namely Na_2_CO_3_, which is able to deprotonate the acidic proton of phenols, and although the formation of **5a** was not observed using THF as solvent, a 72% yield was achieved with DMF. Next, different concentrations of the starting material, temperatures, and number of equivalents of sodium phenoxide were examined, but no improvement was found. Finally, an evaluation of the use of other polar solvents, such as MeCN, NMP, and DMSO, and bases, such as K_2_CO_3_, CsCO_3_, K_3_PO_4_, DBU, DABCO, and DBN, in the reaction also led to lower yields than the initial attempt.

**Scheme 4:**
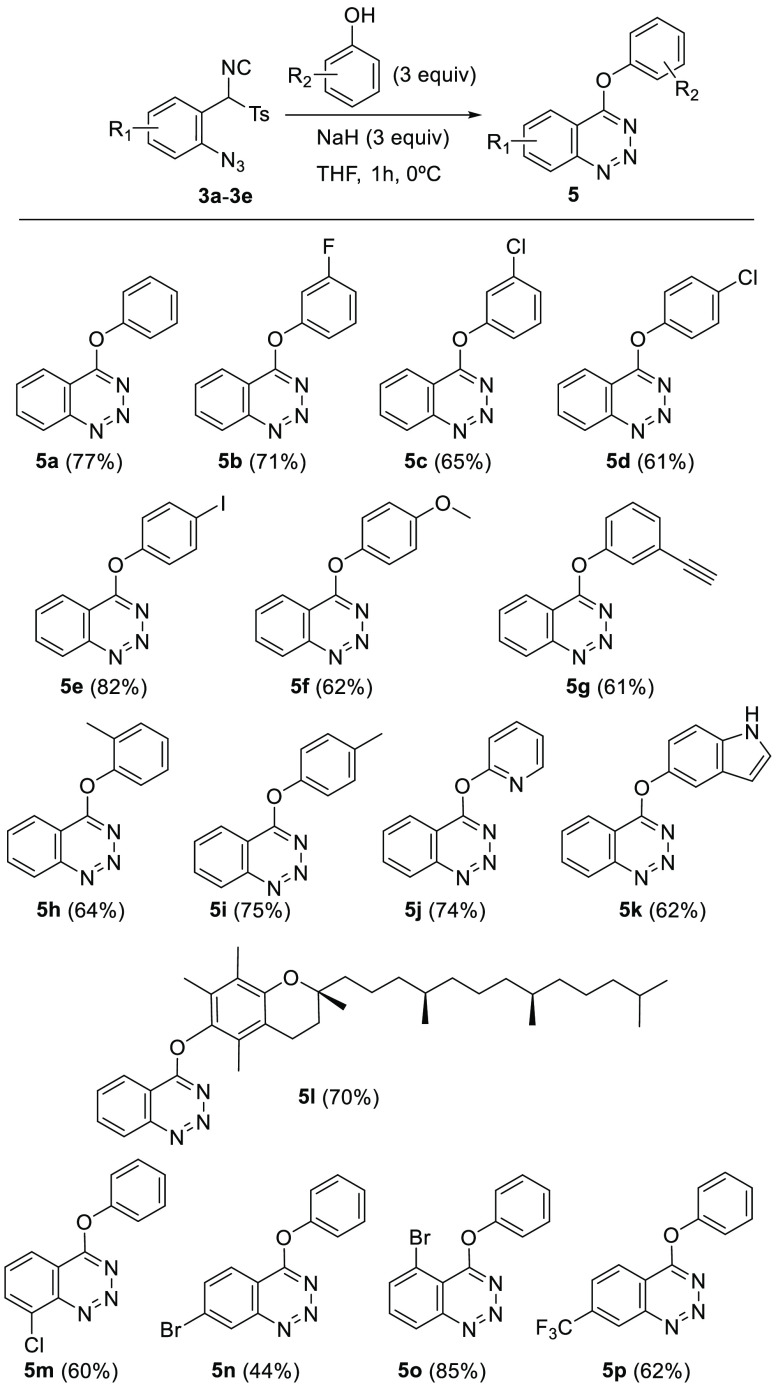
Substrate Scope of 4-Aryloxybenzotriazines **5**

Once again, the optimized conditions were employed to expand the scope of the cyclization. Isonitriles **3a**–**3e** were treated with 3 equiv of a variety of sodium aryloxides, prepared *in situ* from substituted phenol derivatives and NaH, in THF as solvent to afford 4-aryloxybenzotriazines **5** in moderate to high yields (Scheme 4). Substituents of phenol derivatives include electron-withdrawing and -donating groups at different positions of the aromatic ring. Heterocycles such as 2-hydroxypyridine and 5-hydroxyindole were also used successfully, as was the natural product α-tocopherol.

The heterocyclization reaction developed was rationalized by way of a plausible mechanistic hypothesis that involves an initial α-deprotonation of the TosMIC moiety followed by a nucleophilic attack of the anion formed on the electrophilic azide,^19^ which would lead to a cyclization process and loss of the tosyl group. Thereafter, the presence of a second equivalent of the aryl- or alkyloxide would allow a nucleophilic substitution in which the isonitrile would act as a leaving group, thus forming the final benzotriazine.

**Scheme 5:**
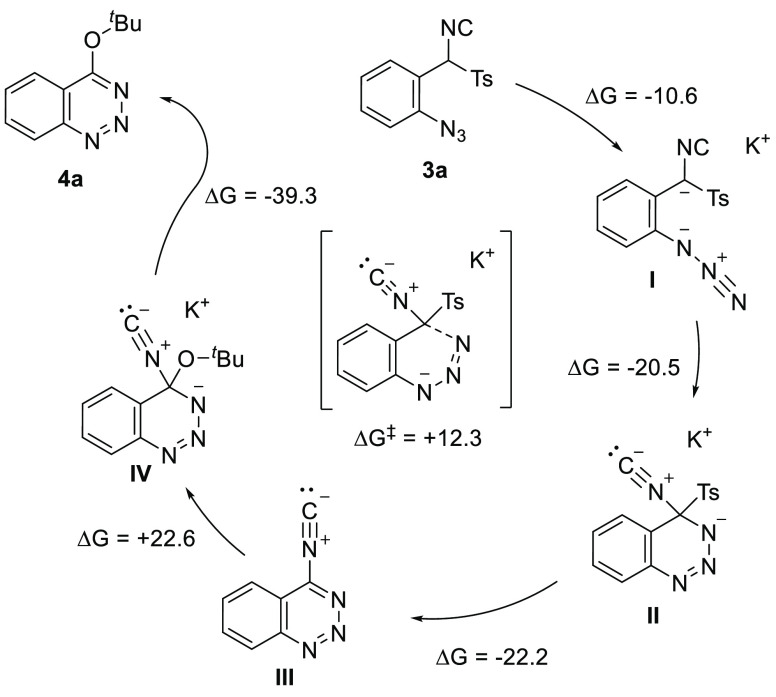
Mechanistic Hypothesis and DFT Calculations

To gain insight into this mechanistic hypothesis, density functional calculations were performed (Scheme 5). In the presence of a base (*t*BuOK), the TosMIC anion **I** is generated, and N1 undergoes a 6-*endo*-*trig* cyclization with the isocyanide, thus resulting in the formation of **II** via TS (Δ*G*^‡^ = +12.3 kcal/mol). Upon losing the tosyl group, the nonaromatic benzotriazine **III** is generated in an exergonic process. This compound can be attacked by a *tert*-butoxide group and thermodynamically driven elimination of CN, thus, leading to the formation of **4a**.

## Conclusions

In summary, a novel and efficient synthetic methodology to afford 4-alkoxy- and 4-aryloxybenzo[*d*][1,2,3]triazines has been developed. This process involves an intramolecular heterocyclization of 1-azido-2-[isocyano(*p*-tosyl)methyl]benzenes under basic conditions, followed by the insertion of an alkyl alcohol or a phenol, and occurs in good to excellent yields with a broad scope. DFT calculations have been performed to further understand the mechanism of this heterocyclization, which opens up a new way to obtain 1,2,3-benzotriazines without the need for harsh conditions or metal catalysis.

## Experimental Section

### General Experimental Details

All reactions involving air-sensitive compounds were carried out under an inert atmosphere (Ar). Dry solvents, where necessary, were dried with an MBRAUN MB-SPS-800 apparatus. Starting materials sourced from commercial suppliers were used as received unless otherwise stated. 2-Azidobenzaldehydes were prepared from the corresponding 2-nitro- or 2-fluorobenzaldehydes according to literature procedures.^20^ Reactions were monitored using analytical TLC plates (Merck; silica gel 60 F254, 0.25 mm), and compounds were visualized with UV radiation. Silica gel grade 60 (70–230 mesh, Merck) was used for column chromatography. All melting points were determined in open capillary tubes on a Stuart Scientific SMP3 melting point apparatus (uncorrected). ^1^H and ^13^C NMR spectra were recorded on a Varian Mercury VX-300, Varian Unity 500 MHz, and Bruker Ascend 400 MHz spectrometer at room temperature. Chemical shifts are given in parts per million (δ) downfield from tetramethylsilane, with calibration on the residual protiosolvent used (δ_H_ = 7.26 ppm and δ_C_ = 77.2 ppm for CDCl_3_). Coupling constants (*J*) are in hertz (Hz), and signals are described as follows: s, singlet; d, doublet; t, triplet; q, quadruplet; bs, broad singlet; dd, double doublet; apt, apparent triplet; dq, double quadruplet; ddd, double doublet of doublets, and m, multiplet. High-resolution analysis (HRMS) were performed on an Agilent 6210 time of-flight LC/MS.

### General Procedure for the Formation of *N*-((2-Azidophenyl)(tosyl)methyl)formamides 2a–2e

A 250 mL dry Schlenk flask was charged with 2 M acetonitrile and 2 M toluene, 1 equiv of the appropriate benzaldehyde (**1**), 2.5 equiv of formamide, and 1.1 equiv of chlorotrimethylsilane under an argon atmosphere. After heating the solution in a sand bath at 50 °C for 4–5 h, 1.5 equiv of *p*-toluenesulfonic acid prepared by a previous procedure^18^ was added and heating was continued for an additional 4–5 h. The solution was cooled to room temperature, and 55 mL of diethyl ether were added. The resulting mixture was cooled to 0 °C and held there for 1 h, and the precipitated white solid was collected using a Büchner funnel. The reaction flask was washed with 35 mL of diethyl ether, and this rinse was poured over the filter cake. After washing with diethyl ether a second time, the solid was dried under vacuum for 5–10 h to give the corresponding *N*-(α-tosyl-2-azido-benzyl)formamide **2** which was used in the next step without further purification.

#### N-((2-Azidophenyl)(tosyl)methyl)formamide (**2a**)

Obtained as a white solid (7.10 g, 21.5 mmol, 93%) following the general procedure outlined above using commercially available compound **1a**(21) (3.40 g, 23.1 mmol). R_*f*_ = 0.3 (Hexane/EtOAc 1:1). Mp 151 °C (decomposition). ^1^H NMR (400 MHz, DMSO-d_6_) δ 9.84 (dd, *J* = 10.5, 1.5 Hz, 1H), 8.04 (d, *J* = 1.3 Hz, 1H), 7.67 (dd, *J* = 7.8, 1.5 Hz, 1H), 7.62–7.57 (m, 2H), 7.52 (td, *J* = 7.7, 1.4 Hz, 1H), 7.45–7.40 (m, 2H), 7.36–7.20 (m, 2H), 6.62 (d, *J* = 10.5 Hz, 1H), 2.40 (s, 3H). ^13^C{^1^H} NMR (101 MHz, DMSO-d_6_) δ: 160.6, 145.2, 138.7, 133.3, 131.4, 129.9, 129.7, 129.1, 125.2, 121.9, 118.8, 64.6, 21.1. HRMS (ESI-TOF) calcd for C_15_H_15_N_4_O_3_S [M + H]^+^: 331.0859. Found: 331.0864.

#### N-((2-Azido-3-chlorophenyl)(tosyl)methyl)formamide (**2b**)

Obtained as a white solid (4.2 g, 11.4 mmol, 94%) following the general procedure outlined above using compound **1b**(22) (3.93 g, 21.6 mmol). R_*f*_ = 0.48 (Hexane/EtOAc 1:1). Mp 160 °C (decomposition). ^1^H NMR (400 MHz, DMSO-d_6_) δ 9.92 (dd, *J* = 10.5, 1.5 Hz, 1H), 8.03 (d, *J* = 1.3 Hz, 1H), 7.67 (td, *J* = 8.1, 6.6 Hz, 4H), 7.50–7.41 (m, 3H), 6.81 (d, *J* = 10.6 Hz, 1H), 2.41 (s, 3H). ^13^C{^1^H} NMR (101 MHz, CDCl_3_) δ: 160.6, 145.5, 135.1, 133.0, 132.0, 129.9, 129.0, 128.6, 127.6, 126.6, 125.5, 65.6, 21.2. HRMS (ESI-TOF) calcd for C_15_H_14_ClN_4_O_3_S [M + H]^+^: 365.0470. Found: 365.0472.

#### N-((2-Azido-4-bromophenyl)(tosyl)methyl)formamide (**2c**)

Obtained as a white solid (5.9 g, 14.4 mmol, 65%) following the general procedure outlined above using compound **1c**(22) (5.11 g, 22.6 mmol). R_*f*_ = 0.3 (Hexane/EtOAc 1:1). Mp 155 °C (decomposition). ^1^H NMR (400 MHz, DMSO-d_6_) δ 9.90 (d, *J* = 10.4 Hz, 1H), 8.02 (s, 1H), 7.60 (dd, *J* = 19.7, 9.7 Hz, 4H), 7.48–7.40 (m, 3H), 6.56 (d, *J* = 10.3 Hz, 1H), 2.41 (s, 3H). ^13^C{^1^H} NMR (400 MHz, DMSO-d_6_) δ: 160.5, 145.3, 140.5, 133.5, 131.5, 129.8, 129.1, 128.2, 124.0, 121.8, 121.2, 64.3, 21.2. HRMS (ESI-TOF) calcd for C_15_H_14_BrN_4_O_3_S [M + H]^+^: 408.8907. Found: 408.8902.

#### N-((2-Azido-6-bromophenyl)(tosyl)methyl)formamide (**2d**)

Obtained as a white solid (7.76 g, 19.0 mmol, 90%) following the general procedure outlined above using compound **1d**(23) (4.8 g, 21.2 mmol). R_*f*_ = 0.4 (Hexane/EtOAc 1:1). Mp 144 °C (decomposition). ^1^H NMR (400 MHz, DMSO-d_6_) δ 9.48 (dd, *J* = 10.6, 1.5 Hz, 1H), 8.09 (d, *J* = 1.3 Hz, 1H), 7.74–7.65 (m, 2H), 7.53 (dd, *J* = 7.2, 2.0 Hz, 1H), 7.49–7.38 (m, 4H), 7.06 (d, *J* = 10.5 Hz, 1H), 2.41 (s, 3H). ^13^C{^1^H} NMR (101 MHz, DMSO-d_6_) δ: 161.1, 145.2, 140.7, 134.7, 132.3, 129.8, 129.5, 128.8, 127.6, 121.4, 120.1, 70.3, 21.1. HRMS (ESI-TOF) calcd for C_15_H_14_BrN_4_O_3_S [M + Na]^+^: 430.9784. Found: 430.9786.

#### N-((2-azido-4-(trifluoromethyl)phenyl)(tosyl)methyl)formamide (**2e**)

Obtained as a white solid (4.3 g, 10.7 mmol, 77%) following the general procedure outlined above using compound **1e**(21) (3.0 g, 13.9 mmol). R_*f*_ = 0.3 (Hexane/EtOAc 1:1). Mp 168 °C (decomposition). ^1^H NMR (400 MHz, DMSO-d_6_) δ 9.96 (dd, *J* = 10.5, 1.5 Hz, 1H), 8.06–8.00 (m, 1H), 7.89 (d, *J* = 8.2 Hz, 1H), 7.73 (dd, *J* = 8.4, 1.8 Hz, 1H), 7.69–7.61 (m, 3H), 7.51–7.41 (m, 3H), 6.69 (d, *J* = 10.5 Hz, 1H), 2.42 (s, 3H). ^13^C{^1^H} NMR (400 MHz, DMSO-d_6_) δ: 162.9, 160.6, 145.5, 140.1, 133.1, 130.9, 129.8, 129.1, 128.1, 126.0, 125.5, 121.7 (q, *J* = 3.7 Hz), 116.2 (q, *J* = 4.0 Hz), 64.3, 21.2. ^19^F NMR (376 MHz, DMSO-d_6_) δ −61.41. HRMS (ESI-TOF) calcd for C_16_H_14_BrF_3_N_4_O_3_S [M + H]^+^: 399.0733. Found: 399.0721.

### General Procedure for the Formation of 1-Azido-2-(isocyano(tosyl)methyl)benzenes 3a–3e

A 250 mL dry Schlenk flask was charged with 1 equiv of the appropriate *N*-(α-tosyl-2-azido-benzyl)formamide **2** and 48 mM of THF. Phosphorus oxychloride (2 equiv) was added, and the resulting solution was stirred for 5 min at 25 °C. After the solution was cooled to 0 °C, 6 equiv of triethylamine were added slowly over 30–45 min while keeping the internal reaction temperature below 10 °C. After the triethylamine addition is complete, the reaction was warmed to room temperature and held there for 30–45 min. Ethyl acetate (140 mL) and water (140 mL) were added sequentially to the reaction, the mixture was stirred for 5 min, and, after transfer of the mixture to a separatory funnel, the aqueous layer was removed. The organic layer was washed with water (2 × 140 mL), saturated NaHCO_3_ solution (140 mL), and brine (70 mL), and the combined organic layers were dried over anhydrous Na_2_SO_4_, filtered, and evaporated under reduced pressure. The residue was purified by flash chromatography using mixtures of hexane and EtOAc as eluents to obtain the corresponding α-tosyl-2-azidobenzyl isocyanide **3**.

#### 1-Azido-2-(isocyano(tosyl)methyl)benzene (**3a**)

Obtained as a yellow solid (571.9 mg, 1.83 mmol, 60%) following the general procedure outlined above using compound **2a** (1.0 g, 3.03 mmol). R_*f*_ = 0.3 (Hexane/EtOAc 2:1). Mp 106–111 °C (decomposition). ^1^H NMR (400 MHz, CDCl_3_) δ 7.74 (d, *J* = 8.3 Hz, 2H), 7.51 (ddd, *J* = 8.0, 7.5, 1.5 Hz, 1H), 7.47 (dd, *J* = 7.9, 1.5 Hz, 1H), 7.39 (d, *J* = 8.5 Hz, 2H), 7.23 (td, *J* = 7.6, 0.9 Hz, 1H), 7.18 (dd, *J* = 8.1, 0.8 Hz, 1H), 6.06 (s, 1H), 2.49 (s, 3H). ^13^C{^1^H} NMR (125 MHz, CDCl_3_) δ: 165.5, 146.9, 139.4, 132.4, 131.3, 130.6, 130.0, 129.7, 125.4, 118.6, 118.5, 70.4, 22.0. HRMS (ESI-TOF) calcd for C_15_H_13_N_4_O_2_S [M + H]^+^: 313.0754. Found: 313.0764.

#### 2-Azido-1-chloro-3-(isocyano(tosyl)methyl)benzene (**3b**)

Obtained as a yellow solid (356.7 mg, 1.03 mmol, 38%) following the general procedure outlined above using compound **2b** (1.0 g, 2.74 mmol). R_*f*_ = 0.3 (Hexane/EtOAc 5:1). Mp 129 °C (decomposition). ^1^H NMR (400 MHz, CDCl_3_) δ 7.80–7.73 (m, 2H), 7.54–7.47 (m, 1H), 7.41 (d, *J* = 8.0 Hz, 2H), 7.36 (d, *J* = 7.8 Hz, 1H), 7.29–7.17 (m, 1H), 6.26 (d, *J* = 2.5 Hz, 1H), 2.50 (s, 3H). ^13^C{^1^H} NMR (101 MHz, CDCl_3_) δ: 166.3, 147.2, 135.9, 133.3, 130.9, 130.6, 130.4, 130.2, 127.8, 126.9, 122.9, 71.3, 22.0. HRMS (ESI-TOF) calcd for C_15_H_12_ClN_4_O_2_S [M + Na]^+^: 369.0183. Found: 369.0190.

#### 2-Azido-4-bromo-1-(isocyano(tosyl)methyl)benzene (**3c**)

Obtained as a yellow solid (350.6 mg, 0.90 mmol, 73%) following the general procedure outlined above using compound **2c** (500 mg, 1.22 mmol). R_*f*_ = 0.3 (Hexane/EtOAc 3:1). Mp 105 °C (decomposition). ^1^H NMR (400 MHz, CDCl_3_) δ 7.77 (d, *J* = 7.9 Hz, 2H), 7.41 (d, *J* = 8.0 Hz, 2H), 7.39–7.30 (m, 3H), 5.98 (s, 1H), 2.50 (s, 3H). ^13^C{^1^H} NMR (101 MHz, CDCl_3_) δ: 166.0, 147.1, 140.7, 131.1, 130.9, 130.6, 130.2, 128.8, 126.4, 121.7, 117.6, 70.0, 22.0. HRMS (ESI-TOF) calcd for C_15_H_12_BrN_4_O_2_S [M + Na]^+^: 412.9678. Found: 412.9678.

#### 1-Azido-3-bromo-2-(isocyano(tosyl)methyl)benzene (**3d**)

Obtained as a yellow solid (450.7 mg, 1.15 mmol, 48%) following the general procedure outlined above using compound **2d** (1.0 g, 2.44 mmol). R_*f*_ = 0.3 (Hexane/EtOAc 1.5:1). Mp 130 °C (violent decomposition). ^1^H NMR (400 MHz, CDCl_3_) δ 7.90–7.81 (m, 2H), 7.52–7.38 (m, 3H), 7.33 (td, *J* = 8.1, 2.7 Hz, 1H), 7.16 (ddd, *J* = 8.1, 6.2, 1.1 Hz, 1H), 6.44 (s, 1H), 2.49 (d, *J* = 2.1 Hz, 3H). ^13^C{^1^H} NMR (101 MHz, CDCl_3_) δ: 167.2, 146.9, 141.8, 133.3, 132.7, 130.3, 130.2, 129.8, 128.3, 119.4, 117.8, 74.6, 22.0. HRMS (ESI-TOF) calcd for C_15_H_12_BrN_4_O_2_S [M + Na]^+^: 412.9678. Found: 412.9676.

#### 2-Azido-1-(isocyano(tosyl)methyl)-4-(trifluoromethyl)benzene (**3e**)

Obtained as a yellow solid (637,6 mg, 1.68 mmol, 67%) following the general procedure outlined above using compound **2e** (1.0 g, 2.51 mmol). R_*f*_ = 0.3 (Hexane/EtOAc 3:1). Mp 93 °C (decomposition). ^1^H NMR (400 MHz, CDCl_3_) δ 7.82–7.75 (m, 2H), 7.63 (d, *J* = 8.2 Hz, 1H), 7.48 (dd, *J* = 8.4, 1.7 Hz, 1H), 7.45–7.39 (m, 3H), 6.08 (s, 1H), 2.51 (s, 3H). ^13^C{^1^H} NMR (101 MHz, CDCl_3_) δ: 166.4, 147.3, 140.4, 134.6 (q, *J* = 33.4 Hz), 131.0, 130.6, 130.5, 130.3, 122.14, 122.05 (q, *J* = 3.7 Hz), 122.0, 115.5 (q, *J* = 3.8 Hz), 69.9, 22.0. ^19^F NMR (376 MHz, CDCl_3_) δ −63.2. HRMS (ESI-TOF) calcd for C_15_H_12_F_3_N_4_O_2_S [M + H]^+^: 381.0628. Found: 381.0627.

### General Procedure for the Formation of Benzo[*d*][1,2,3]triazines 4 and 5

NaH (3.0 equiv) was dissolved in THF (0.2 M) and stirred for about 5 min under Ar. The corresponding alcohol or phenol (3.0 equiv) was added at rt. After 2 min, the mixture was cooled to 0 °C and a solution of the corresponding isocyanide (**3**) (1 equiv) in THF (0.04 M) was added. The reaction mixture was stirred at 0 °C for 1 h. After this time, the reaction mixture was warmed to room temperature and diluted with ethyl acetate and water. The aqueous layer was extracted 2 times with ethyl acetate, and the collected organic phases were then washed one more time with brine. After evaporation of the solvent, the crude material was purified by flash chromatography using mixtures of hexane/EtOAc as eluents to obtain the corresponding triazine **4** or **5**.

#### 4-(tert-Butoxy)benzo[d][1,2,3]triazine (**4a**)

Obtained as a light brown solid (12.9 mg, 0.06 mmol, 40%) following the general procedure outlined above using compound **3a** (50 mg, 0.16 mmol). R_*f*_ = 0.3 (Hexane/EtOAc 4:1). Mp 137 °C (decomposition). ^1^H NMR (400 MHz, CDCl_3_) δ: 8.30 (d, *J* = 8.3 Hz, 1H), 8.14 (d, *J* = 8.1 Hz, 1H), 7.98 (t, *J* = 7.6 Hz, 1H), 7.85 (t, *J* = 7.6 Hz, 1H), 1.82 (s, 9H). ^13^C{^1^H} NMR (101 MHz, CDCl_3_) δ: 160.3, 146.0, 134.3, 132.5, 127.8, 122.4, 112.3, 85.1, 28.4. HRMS (ESI-TOF) calcd for C_11_H_14_N_3_O [M + H]^+^: 204.1131. Found: 204.1130.

#### 4-(Allyloxy)benzo[d][1,2,3]triazine (**4b**)

Obtained as a green-brown solid (165.2 mg, 0.88 mmol, 88%) from compound **3a** (1.0 mmol, 312.4 mg) following the same general procedure described above. R_*f*_ = 0.3 (Hexane/EtOAc 4:1). Mp 110 °C (decomposition). ^1^H NMR (400 MHz, CDCl_3_) δ 8.31 (d, *J* = 8.4 Hz, 1H), 8.16 (d, *J* = 8.2 Hz, 1H), 7.98 (ddd, *J* = 8.4, 7.1, 1.4 Hz, 1H), 7.85 (ddd, *J* = 8.2, 7.1, 1.1 Hz, 1H), 6.16 (ddt, *J* = 17.1, 10.4, 5.8 Hz, 1H), 5.48 (dq, *J* = 17.2, 1.4 Hz, 1H), 5.33 (dq, *J* = 10.4, 1.2 Hz, 1H), 5.21 (dt, *J* = 5.8, 1.3 Hz, 2H). ^13^C{^1^H} NMR (101 MHz, CDCl_3_) δ: 160.4, 146.0, 134.8, 132.9, 131.8, 128.0, 122.0, 119.6, 111.2, 68.9. HRMS (ESI-TOF) calcd for C_10_H_10_N_3_O [M + H]^+^: 188.0818. Found: 188.0826.

#### 4-Methoxybenzo[d][1,2,3]triazine (**4c**)

Obtained as a light brown solid (9.3 mg, 0.06 mmol, 60%) following the general procedure outlined above using compound **3a** (30.0 mg, 0.09 mmol). R_*f*_ = 0.3 (Hexane/EtOAc 4:1). Mp 99 °C (decomposition). ^1^H NMR (400 MHz, CDCl_3_) δ: 8.38 (d, *J* = 8.4 Hz, 1H), 8.19 (d, *J* = 9.4 Hz, 1H), 8.05 (t, *J* = 7.0 Hz, 1H), 7.91 (t, *J* = 8.2 Hz, 1H), 4.35 (s, 3H). ^13^C{^1^H} NMR (101 MHz, CDCl_3_) δ: 161.0, 145.9, 134.8, 132.9, 128.0, 122.0, 111.1, 55.6. HRMS (ESI-TOF) calcd for C_8_H_8_N_3_O [M + H]^+^: 162.0662. Found: 162.0666.

#### 4-Butoxybenzo[d][1,2,3]triazine (**4d**)

Obtained as a white solid (29.0 mg, 0.1 mmol, 74%) from **3a** (60.0 mg, 0.2 mmol) following the general method. R_*f*_ = 0.39 (Hexane/EtOAc 4:1). Mp 133 °C (decomposition). ^1^H NMR (400 MHz, CDCl_3_) δ: 8.36 (d, *J* = 8.6 Hz, 1H), 8.19 (d, *J* = 8.2 Hz, 1H), 8.03 (t, *J* = 7.7 Hz, 1H), 7.89 (t, *J* = 7.6 Hz, 1H), 4.76 (t, *J* = 6.2 Hz, 2H), 1.94 (quintet, *J* = 7.2 Hz, 2H), 1.57 (sextet, *J* = 7.6 Hz, 2H), 1.02 (t, *J* = 7.7 Hz, 3H). ^13^C{^1^H} NMR (101 MHz, CDCl_3_) δ: 160.8, 145.9, 134.7, 132.8, 127.9, 122.0, 111.2, 68.5, 30.9, 19.4, 13.9. HRMS (ESI-TOF) calcd for C_11_H_14_N_3_O [M + H]^+^: 204.1131. Found: 204.1130.

#### 4-Isopropoxybenzo[d][1,2,3]triazine (**4e**)

Obtained as a light brown solid (29.8 mg, 0.16 mmol, 82%) from **3a** (60.0 mg, 0.19 mmol) following the general method. R_*f*_ = 0.3 (Hexane/EtOAc 4:1). Mp 129 °C (decomposition). ^1^H NMR (400 MHz, CDCl_3_) δ: 8.28 (d, *J* = 8.4 Hz, 1H), 8.12 (d, *J* = 8.2 Hz, 1H), 7.95 (t, *J* = 7.8 Hz, 1H), 7.81 (t, *J* = 7.6 Hz, 1H), 5.87–5.76 (m, 1H), 1.47 (dd, *J* = 6.3, 2.2 Hz, 6H). ^13^C{^1^H} NMR (101 MHz, CDCl_3_) δ: 160.3, 146.0, 134.6, 132.6, 127.9, 122.1, 111.5, 72.1, 22.0. HRMS (ESI-TOF) calcd for C_10_H_12_N_3_O [M + H]^+^: 190.0975. Found: 190.0974.

#### 4-(sec-Butoxy)benzo[d][1,2,3]triazine (**4f**)

Obtained as a yellow oil (15.7 mg, 0.07 mmol, 80%) from **3a** (30 mg, 0.1 mmol) following the general method. R_*f*_ = 0.3 (Hexane/EtOAc 4:1). Mp 128 °C (decomposition). ^1^H NMR (400 MHz, CDCl_3_) δ: 8.35 (dt, *J* = 8.4, 1.0 Hz, 1H), 8.19 (dt, *J* = 8.1, 1.0 Hz, 1H), 8.02 (ddd, *J* = 8.4, 7.1, 1.4 Hz, 1H), 7.88 (ddd, *J* = 8.2, 7.1, 1.2 Hz, 1H), 5.73 (h, *J* = 6.2 Hz, 1H), 1.95 (ddd, *J* = 14.2, 7.6, 6.7 Hz, 1H), 1.82 (dqd, *J* = 14.8, 7.5, 5.7 Hz, 1H), 1.51 (d, *J* = 6.2 Hz, 3H), 1.04 (t, *J* = 7.5 Hz, 3H). ^13^C{^1^H} NMR (101 MHz, CDCl_3_) δ: 160.6, 146.0, 134.6, 132.6, 127.9, 122.1, 111.6, 76.6, 29.0, 19.3, 9.8. HRMS (ESI-TOF) calcd for C_11_H_14_N_3_O [M + H]^+^: 204.1131. Found: 204.1132.

#### 4-((2-Isopropyl-5-methylcyclohexyl)oxy)benzo[d][1,2,3]triazine (**4g**)

Obtained as a white solid (14.7 mg, 0.05 mmol, 27%) from **3a** (60 mg, 0.2 mmol) following the general method. R_*f*_ = 0.3 (Hexane/EtOAc 5:1). Mp 120 °C (decomposition). ^1^H NMR (400 MHz, CDCl_3_) δ 8.28 (dt, *J* = 8.3, 1.0 Hz, 1H), 8.12 (dt, *J* = 8.1, 1.0 Hz, 1H), 7.96 (ddd, *J* = 8.4, 7.1, 1.4 Hz, 1H), 7.82 (ddd, *J* = 8.2, 7.1, 1.2 Hz, 1H), 5.55 (td, *J* = 10.6, 4.4 Hz, 1H), 2.42–2.32 (m, 1H), 1.97 (pd, *J* = 6.9, 2.6 Hz, 1H), 1.77–1.55 (m, 4H), 1.24–1.00 (m, 2H), 0.99–0.90 (m, 1H), 0.88 (dd, *J* = 6.8, 3.9 Hz, 6H), 0.74 (d, *J* = 6.9 Hz, 3H). ^13^C{^1^H} NMR (101 MHz, CDCl_3_) δ: 160.7, 146.1, 134.6, 132.6, 128.0, 122.1, 111.6, 78.5, 47.8, 40.2, 34.5, 31.5, 27.0, 24.1, 22.2, 20.8, 17.1. HRMS (ESI-TOF) calcd for C_17_H_24_N_3_O [M + H]^+^: 286.1914. Found: 286.1915.

#### 4-((1,1,1,3,3,3-Hexafluoropropan-2-yl)oxy)benzo[d][1,2,3]triazine (**4h**)

Obtained as a white solid (40.5 mg, 0.14 mmol, 71%) from **3a** (60.0 mg, 0.2 mmol) following the general method. R_*f*_ = 0.3 (Hexane/EtOAc 5:1). Mp 136 °C (decomposition). ^1^H NMR (400 MHz, CDCl_3_) δ: 8.53 (d, *J* = 8.5, 1H), 8.30 (dd, *J* = 8.1, 1.4, 1H), 8.20 (ddd, *J* = 8.5, 7.1, 1.4 Hz, 1H), 8.06 (ddd, *J* = 8.2, 7.1, 1.2 Hz, 1H), 7.02 (hept, *J* = 6.0 Hz, 1H). ^13^C{^1^H} NMR (101 MHz, CDCl_3_) δ: 159.0, 146.8, 136.2, 134.3, 130.1, 128.6, 122.1, 121.5, 110.1, 68.7 (q, *J* = 70.3, 35.0 Hz). ^19^F-NMR (376 MHz, CDCl_3_) δ – 72.97. HRMS (ESI-TOF) calcd for C_10_H_6_F_6_N_3_O [M + H]^+^: 298.0410. Found: 298.0410.

#### 4-(Prop-2-yn-1-yloxy)benzo[d][1,2,3]triazine (**4i**)

Obtained a yellow oil (18.3 mg, 0.10 mmol, 62%) following the general procedure outlined above using compound **3a** (50 mg, 0.16 mmol). R_*f*_ = 0.3 (Hexane/EtOAc 4:1). ^1^H NMR (400 MHz, CDCl_3_) δ: 8.41 (d, *J* = 8.3 Hz, 1H), 8.25 (d, *J* = 8.1 Hz, 1H), 8.08 (ddd, *J* = 8.4, 7.1, 1.4 Hz, 1H), 7.94 (ddd, *J* = 8.2, 7.2, 1.1 Hz, 1H), 5.40 (d, *J* = 2.4 Hz, 2H), 2.62 (t, *J* = 2.4 Hz, 1H). ^13^C{^1^H} NMR (101 MHz, CDCl_3_) δ: 159.8, 146.1, 135.1, 133.2, 128.1, 122.0, 110.9, 77.3, 76.3, 55.8. HRMS (ESI-TOF) calcd for C_10_H_8_N_3_O [M + H]^+^: 186.0662. Found: 186.0663.

#### 4-((2-Methylbut-3-yn-2-yl)oxy)benzo[d][1,2,3]triazine (**4j**)

Obtained as a brown solid (28.8 mg, 0.14 mmol, 70%) following the general procedure outlined above using compound **3a** (60 mg, 0.19 mmol). R_*f*_ = 0.3 (Hexane/EtOAc 4:1). Mp 138 °C (decomposition). ^1^H NMR (400 MHz, CDCl_3_) δ 8.36 (dd, *J* = 7.9, 1.0 Hz, 1H), 8.00–7.79 (m, 3H), 2.76 (s, 1H), 2.11 (s, 6H). ^13^C{^1^H} NMR (101 MHz, CDCl_3_) δ: 168.0, 146.4, 134.6, 133.8, 126.8, 125.8, 118.0, 83.0, 75.8, 73.9, 30.1. HRMS (ESI-TOF) calcd for C_12_H_12_N_3_O [M + H]^+^: 214.0975. Found: 214.0982.

#### 4-(But-3-yn-1-yloxy)benzo[d][1,2,3]triazine (**4k**)

Obtained a yellow oil (23.3 mg, 0.12 mmol, 69%) following the general procedure outlined above using compound **3a** (60 mg, 0.19 mmol). R_*f*_ = 0.25 (Hexane/EtOAc 4:1). ^1^H NMR (400 MHz, CDCl_3_) δ 8.37 (d, *J* = 8.4 Hz, 1H), 8.22 (d, *J* = 8.2 Hz, 1H), 8.05 (t, *J* = 7.7 Hz, 1H), 7.91 (t, *J* = 7.6 Hz, 1H), 4.91–4.83 (m, 2H), 2.87 (td, *J* = 6.6, 2.9 Hz, 2H), 2.05 (t, *J* = 2.7 Hz, 1H). ^13^C{^1^H} NMR (101 MHz, CDCl_3_) δ: 160.4, 146.0, 134.9, 133.0, 128.0, 122.0, 111.0, 79.9, 70.4, 66.0, 19.2. HRMS (ESI-TOF) calcd for C_11_H_10_N_3_O [M + H]^+^: 200.0818. Found: 200.0819.

#### 4-(Pent-3-yn-1-yloxy)benzo[d][1,2,3]triazine (**4l**)

Obtained as a yellow-brown solid (20.9. mg, 0.01 mmol, 51%) from **3a** (60 mg, 0.20 mmol) following the general method. R_*f*_ = 0.3 (Hexane/EtOAc 4:1). Mp 125 °C (decomposition). ^1^H NMR (400 MHz, CDCl_3_) δ 8.38 (dd, *J* = 8.4, 1.0 Hz, 1H), 8.23 (dd, *J* = 8.1, 1.3 Hz, 1H), 8.05 (ddt, *J* = 8.2, 7.2, 1.0 Hz, 1H), 7.91 (tt, *J* = 8.0, 1.0 Hz, 1H), 4.82 (t, *J* = 6.8 Hz, 2H), 2.80 (tq, *J* = 7.0, 2.6 Hz, 2H), 1.79 (t, *J* = 2.6 Hz, 3H). ^13^C{^1^H} NMR (101 MHz, CDCl_3_) δ: 160.6, 146.0, 134.8, 132.9, 128.0, 122.1, 111.1, 77.9, 74.6, 66.8, 19.5, 3.6. HRMS (ESI-TOF) calcd for C_12_H_12_N_3_O [M + H]^+^: 214.0975. Found: 214.0978.

#### 4-(Benzyloxy)benzo[d][1,2,3]triazine (**4m**)

Obtained as a light yellow solid (19.4 mg, 0.08 mmol, 85%) from **3a** (30 mg, 0.10 mmol) following the general method. R_*f*_ = 0.3 (Hexane/EtOAc 3:1). Mp 152 °C (decomposition). ^1^H NMR (400 MHz, CDCl_3_) δ 8.39 (dd, J = 8.4, 1.0 Hz, 1H), 8.22 (dt, J = 8.1, 1.0 Hz, 1H), 8.05 (ddd, J = 8.4, 7.1, 1.4 Hz, 1H), 7.89 (ddd, J = 8.2, 7.1, 1.2 Hz, 1H), 7.66–7.54 (m, 2H), 7.48–7.35 (m, 3H), 5.81 (s, 2H). ^13^C{^1^H} NMR (101 MHz, CDCl_3_) δ: 160.6, 146.1, 135.5, 134.8, 133.0, 128.9, 128.8, 128.0, 122.1, 111.2, 70.0. HRMS (ESI-TOF) calcd for C_14_H_12_N_3_O [M + H]^+^: 238.0975. Found: 238.0973.

#### 4-(Pyridin-2-ylmethoxy)benzo[d][1,2,3]triazine (**4n**)

Obtained as a light green solid (16.7 mg, 0.07 mmol, 73%) from **3a** (30 mg, 0.10 mmol) following the general method. R_*f*_ = 0.3 (Hexane/EtOAc 3:1). Mp 166 °C (decomposition). ^1^H NMR (400 MHz, CDCl_3_) δ 8.59 (ddd, *J* = 4.9, 1.8, 1.0 Hz, 1H), 8.33 (dt, *J* = 8.4, 1.0 Hz, 1H), 8.21 (dt, *J* = 8.2, 1.0 Hz, 1H), 7.99 (ddd, *J* = 8.4, 7.1, 1.4 Hz, 1H), 7.85 (ddd, *J* = 8.2, 7.1, 1.2 Hz, 1H), 7.69 (td, *J* = 7.7, 1.8 Hz, 1H), 7.53 (dt, *J* = 7.8, 1.1 Hz, 1H), 7.22 (ddd, *J* = 7.6, 4.8, 1.2 Hz, 1H), 5.84 (s, 2H). ^13^C{^1^H} NMR (101 MHz, CDCl_3_) δ: 160.5, 155.3, 149.9, 146.1, 137.0, 134.9, 133.1, 128.0, 123.4, 122.8, 122.1, 111.1, 70.4. HRMS (ESI-TOF) calcd for C_13_H_11_N_4_O [M + H]^+^: 239.0927. Found: 239.0931.

#### 4-(Allyloxy)-8-chlorobenzo[d][1,2,3]triazine (**4o**)

Obtained as a yellow solid (13.5 mg, 0.07 mmol, 70%) from **3b** (30 mg, 0.1 mmol) following the general method. R_*f*_ = 0.3 (Hexane/EtOAc 4:1). Mp 131 °C (decomposition). ^1^H NMR (400 MHz, CDCl_3_) δ 8.15 (dd, *J* = 8.2, 1.3 Hz, 1H), 8.09 (dd, *J* = 7.7, 1.3 Hz, 1H), 7.82 (t, *J* = 7.9 Hz, 1H), 6.21 (ddt, *J* = 17.3, 10.4, 5.9 Hz, 1H), 5.55 (dq, *J* = 17.1, 1.5 Hz, 1H), 5.41 (dq, *J* = 10.5, 1.2 Hz, 1H), 5.28 (dt, *J* = 5.9, 1.4 Hz, 2H). ^13^C{^1^H} NMR (101 MHz, CDCl_3_) δ: 160.1, 142.7, 135.1, 133.7, 132.9, 131.5, 120.8, 120.0, 112.8, 69.5. HRMS (ESI-TOF) calcd for C_10_H_9_ClN_3_O [M + H]^+^: 222.0429. Found: 222.0430.

#### 4-(Allyloxy)-7-bromobenzo[d][1,2,3]triazine (**4p**)

Obtained as a light gray solid (29.5 mg, 0.11 mmol, 72%) from **3c** (60.0 mg, 0.15 mmol) following the general method. R_*f*_ = 0.3 (Hexane/EtOAc 5:1). Mp 87–88 °C (decomposition). ^1^H NMR (400 MHz, CDCl_3_) δ 8.54 (d, *J* = 1.9 Hz, 1H), 8.09 (d, *J* = 8.6 Hz, 1H), 7.99 (dd, *J* = 8.7, 1.9 Hz, 1H), 6.21 (ddt, *J* = 17.2, 10.4, 5.9 Hz, 1H), 5.55 (dq, *J* = 17.2, 1.5 Hz, 1H), 5.41 (dq, *J* = 10.4, 1.2 Hz, 1H), 5.27 (dt, *J* = 5.9, 1.3 Hz, 2H). ^13^C{^1^H} NMR (101 MHz, CDCl_3_) δ: 160.2, 146.5, 136.6, 131.5, 130.5, 129.4, 123.7, 120.0, 109.8, 69.2. HRMS (ESI-TOF) calcd for C_10_H_9_BrN_3_O [M + H]^+^: 265.9924. Found: 265.9923.

#### 4-(Allyloxy)-5-bromobenzo[d][1,2,3]triazine (**4q**)

Obtained as a light yellow solid (12.3 mg, 0.05 mmol, 60%) from **3d** (30 mg, 0.08 mmol) following the general method. R_*f*_ = 0.3 (Hexane/EtOAc 5:1). Mp 126 °C (decomposition). ^1^H NMR (400 MHz, CDCl_3_) δ 8.34 (d, *J* = 8.1 Hz, 1H), 8.15 (d, *J* = 7.8 Hz, 1H), 7.83 (t, *J* = 8.1 Hz, 1H), 6.24 (ddt, *J* = 16.5, 10.8, 5.9 Hz, 1H), 5.62 (d, *J* = 17.3 Hz, 1H), 5.40 (d, *J* = 10.5 Hz, 1H), 5.29 (d, *J* = 5.6 Hz, 2H). ^13^C{^1^H} NMR (101 MHz, CDCl_3_) δ: 159.0, 147.3, 138.8, 134.8, 131.6, 128.0, 119.6, 116.5, 111.3, 69.8. HRMS (ESI-TOF) calcd for C_10_H_9_BrN_3_O [M + H]^+^: 265.9924. Found: 265.9928.

#### 4-(Allyloxy)-7-(trifluoromethyl)benzo[d][1,2,3]triazine (**4r**)

Obtained as a yellow solid (25.8. mg, 0.10 mmol, 77%) from **3e** (50 mg, 0.13 mmol) following the general method. R_*f*_ = 0.3 (Hexane/EtOAc 4:1). Mp 92 °C (decomposition). ^1^H NMR (400 MHz, CDCl_3_) δ 8.67 (dt, *J* = 1.8, 0.9 Hz, 1H), 8.37 (dt, *J* = 8.5, 0.8 Hz, 1H), 8.09 (dd, *J* = 8.5, 1.7 Hz, 1H), 6.22 (ddt, *J* = 17.2, 10.4, 5.9 Hz, 1H), 5.57 (dq, *J* = 17.2, 1.4 Hz, 1H), 5.43 (dq, *J* = 10.4, 1.2 Hz, 1H), 5.30 (dt, *J* = 5.9, 1.3 Hz, 2H). ^13^C{^1^H} NMR (101 MHz, CDCl_3_) δ: 160.0, 145.2, 136.6 (q, *J* = 33.6 Hz), 131.3, 128.7 (q, *J* = 3.1 Hz), 125.9 (q, *J* = 4.3 Hz), 123.8, 123.0 (q, *J* = 274.7 Hz), 120.3, 112.9, 69.5. ^19^F NMR (376 MHz, CDCl_3_) δ −63.3. HRMS (ESI-TOF) calcd for C_11_H_9_F_3_N_3_O [M + H]^+^: 256.0692. Found: 256.0696.

#### 4-Phenoxybenzo[d][1,2,3]triazine (**5a**)

Obtained as a brown solid (32.8 mg, 0.15 mmol, 77%) following the general procedure outlined above using compound **3a** (60 mg, 0.19 mmol). R_*f*_ = 0.3 (Hexane/EtOAc 4:1). Mp 166 °C (decomposition). ^1^H NMR (400 MHz, CDCl_3_) δ 8.48–8.37 (m, 2H), 8.13 (ddd, *J* = 8.4, 7.1, and 1.4 Hz, 1H), 8.01 (ddd, *J* = 8.2, 7.1, and 1.2 Hz, 1H), 7.55–7.46 (m, 2H), 7.39–7.30 (m, 3H). ^13^C{^1^H} NMR (101 MHz, CDCl_3_) δ: 161.5, 152.2, 146.6, 135.3, 133.4, 130.0, 128.2, 126.5, 122.1, 121.7, 111.0. HRMS (ESI-TOF) calcd for C_13_H_10_N_3_O [M + H]^+^: 224.0818. Found: 224.0824.

#### 4-(3-Fluorophenoxy)benzo[d][1,2,3]triazine (**5b**)

Obtained as a light yellow solid (16.5 mg, 0.07 mmol, 71%) from **3a** (30 mg, 0.10 mmol) following the general method. R_*f*_ = 0.3 (Hexane/EtOAc 4:1). Mp 144 °C (decomposition). ^1^H NMR (400 MHz, CDCl_3_) δ 8.47 (d, *J* = 8.4, 1H), 8.39 (dd, *J* = 8.1, 1.4, 1H), 8.15 (ddd, *J* = 8.4, 7.1, 1.4 Hz, 1H), 8.03 (ddd, *J* = 8.2, 7.1, 1.2 Hz, 1H), 7.47 (td, *J* = 8.2, 6.4 Hz, 1H), 7.19–7.02 (m, 3H). ^13^C{^1^H} NMR (101 MHz, CDCl_3_) δ: 163.3 (d, *J* = 248.2 Hz), 161.3, 152.8 (d, *J* = 10.9 Hz), 146.7, 135.5, 133.6, 130.8 (d, *J* = 9.4 Hz), 128.3, 122.0, 117.6 (d, *J* = 3.5 Hz), 113.6 (d, *J* = 21.1 Hz), 110.0 (d, *J* = 24.7 Hz), 109.9. ^19^F-NMR (376 MHz, CDCl_3_) δ – 110.24. HRMS (ESI-TOF) calcd for C_13_H_9_FN_3_O [M + H]^+^: 242.0724. Found: 242.0726.

#### 4-(3-Chlorophenoxy)benzo[d][1,2,3]triazine (**5c**)

Obtained as a light brown solid (16.2 mg, 0.06 mmol, 65%) from **3a** (30.0 mg, 0.1 mmol) following the general method. R_*f*_ = 0.3 (Hexane/EtOAc 4:1). Mp 178 °C (decomposition). ^1^H NMR (400 MHz, CDCl_3_) δ 8.40 (dt, *J* = 8.4, 1.0 Hz, 1H), 8.31 (dd, *J* = 8.1, 1.4, 1H), 8.08 (ddd, *J* = 8.5, 7.1, 1.4 Hz, 1H), 7.96 (ddd, *J* = 8.2, 7.1, 1.2 Hz, 1H), 7.37 (t, *J* = 8.1 Hz, 1H), 7.35–7.22 (m, 2H), 7.19 (ddd, *J* = 8.2, 2.3, 1.0 Hz, 1H). ^13^C{^1^H} NMR (101 MHz, CDCl_3_) δ: 161.3, 152.5, 146.7, 135.5, 135.3, 133.7, 130.7, 128.3, 126.8, 122.5, 121.9, 120.2, 110.8. HRMS (ESI-TOF) calcd for C_13_H_9_ClN_3_O [M + H]^+^: 258.0429. Found: 258.0431.

#### 4-(4-Chlorophenoxy)benzo[d][1,2,3]triazine (**5d**)

Obtained as a white solid (15.1 mg, 0.06 mmol, 61%) from **3a** (30 mg, 0.10 mmol) following the general method. R_*f*_ = 0.3 (Hexane/EtOAc 4:1). Mp 155 °C (decomposition). ^1^H NMR (400 MHz, CDCl_3_) δ 8.46 (dt, *J* = 8.4, 1.0 Hz, 1H), 8.39 (dd, *J* = 8.1, 1.4, 1H), 8.14 (ddd, *J* = 8.4, 7.1, 1.4 Hz, 1H), 8.03 (ddd, *J* = 8.2, 7.1, 1.2 Hz, 1H), 7.51–7.42 (m, 2H), 7.35–7.24 (m, 2H). ^13^C{^1^H} NMR (101 MHz, CDCl_3_) δ: 161.4, 150.6, 146.6, 135.5, 133.6, 131.9, 130.1, 128.3, 123.2, 121.9, 110.9. HRMS (ESI-TOF) calcd for C_13_H_9_ClN_3_O [M + H]^+^: 258.0429. Found: 258.0430.

#### 4-(4-Iodophenoxy)benzo[d][1,2,3]triazine (**5e**)

Obtained as a brown solid (27.5 mg, 0.79 mmol, 82%) from **3a** (30.0 mg, 0.10 mmol) following the general method. R_*f*_ = 0.3 (Hexane/EtOAc 4:1). Mp 158 °C (decomposition). ^1^H NMR (400 MHz, CDCl_3_) δ 8.46 (d, *J* = 8.4, 1H), 8.38 (ddd, *J* = 8.1, 1.5, 1H), 8.14 (dd, *J* = 8.5, 7.1, 1.4 Hz, 1H), 8.02 (ddd, *J* = 8.2, 7.1, 1.2 Hz, 1H), 7.85–7.77 (m, 2H), 7.16–7.08 (m, 2H). ^13^C{^1^H} NMR (101 MHz, CDCl_3_) δ: 161.3, 152.0, 146.7, 139.1, 135.5, 133.6, 128.3, 124.0, 122.0, 110.9, 90.6. HRMS (ESI-TOF) calcd for C_13_H_9_IN_3_O [M + H]^+^: 349.9785. Found: 349.9787.

#### 4-(4-Methoxyphenoxy)benzo[d][1,2,3]triazine (**5f**)

Obtained as a brown solid (15.2 mg, 0.06 mmol, 62%) from **3a** (30 mg, 0.1 mmol) following the general method. R_*f*_ = 0.3 (Hexane/EtOAc 3:1). Mp 171 °C (decomposition). ^1^H NMR (400 MHz, CDCl_3_) δ 8.35 (dd, *J* = 15.8, 8.2 Hz, 2H), 8.05 (t, *J* = 7.7 Hz, 1H), 7.93 (t, *J* = 7.6 Hz, 1H), 7.18 (d, *J* = 8.1 Hz, 2H), 6.93 (d, *J* = 8.6 Hz, 2H), 3.78 (s, 3H). ^13^C{^1^H} NMR (101 MHz, CDCl_3_) δ: 161.8, 157.8, 146.5, 145.6, 135.2, 133.3, 128.1, 122.5, 122.1, 115.0, 111.1, 55.8. HRMS (ESI-TOF) calcd for C_14_H_12_N_3_O_2_ [M + H]^+^: 254.0924. Found: 254.0925.

#### 4-(3-Ethynylphenoxy)benzo[d][1,2,3]triazine (**5g**)

Obtained as a light brown solid (14.4 mg, 0.06 mmol, 61%) from **3a** (30 mg, 0.1 mmol) following the general method. R_*f*_ = 0.3 (Hexane/EtOAc 3:1). Mp 148 °C (decomposition). ^1^H NMR (400 MHz, CDCl_3_) δ 8.39 (d, *J* = 8.4 Hz, 1H), 8.35–8.29 (m, 1H), 8.07 (t, *J* = 7.8 Hz, 1H), 8.00–7.91 (m, 1H), 7.40 (t, *J* = 2.4 Hz, 3H), 7.28 (dt, *J* = 6.7, 2.2 Hz, 1H), 3.09–3.03 (m, 1H). ^13^C{^1^H} NMR (101 MHz, CDCl_3_) δ: 161.4, 151.9, 146.7, 135.5, 133.6, 130.3, 130.0, 128.3, 125.4, 124.1, 122.6, 122.0, 110.9, 82.6, 78.6. HRMS (ESI-TOF) calcd for C_15_H_10_N_3_O [M + H]^+^: 248.0818. Found: 248.0820.

#### 4-(o-Tolyloxy)benzo[d][1,2,3]triazine (**5h**)

Obtained as a brown solid (14.5 mg, 0.06 mmol, 64%) from **3a** (30 mg, 0.10 mmol) following the general method. R_*f*_ = 0.3 (Hexane/EtOAc 4:1). Mp 149 °C (decomposition). ^1^H NMR (400 MHz, CDCl_3_) δ 8.38 (t, *J* = 8.6 Hz, 2H), 8.07 (t, *J* = 7.8 Hz, 1H), 7.95 (t, *J* = 7.7 Hz, 1H), 7.30–7.22 (m, 2H), 7.20 (d, *J* = 7.1 Hz, 1H), 7.15 (d, *J* = 8.2 Hz, 1H), 2.13 (s, 3H). ^13^C{^1^H} NMR (101 MHz, CDCl_3_) δ: 161.1, 150.8, 146.6, 135.3, 133.4, 131.8, 130.3, 128.2, 127.5, 126.7, 122.0, 121.9, 110.8, 16.4. HRMS (ESI-TOF) calcd for C_14_H_12_N_3_O [M + H]^+^: 238.0975. Found: 238.0976.

#### 4-(p-Tolyloxy)benzo[d][1,2,3]triazine (**5i**)

Obtained as a brown solid (17.1 mg, 0.07 mmol, 75%) from **3a** (30 mg, 0.10 mmol) following the general method. R_*f*_ = 0.3 (Hexane/EtOAc 4:1). Mp 150 °C (decomposition). ^1^H NMR (400 MHz, CDCl_3_) δ 8.42 (dd, *J* = 14.4, 8.3 Hz, 2H), 8.12 (t, *J* = 7.8 Hz, 1H), 8.00 (t, *J* = 7.6 Hz, 1H), 7.29 (d, *J* = 7.4 Hz, 2H), 7.21 (dd, *J* = 8.7, 2.3 Hz, 2H), 2.41 (s, 3H). ^13^C{^1^H} NMR (101 MHz, CDCl_3_) δ: 161.7, 149.9, 146.5, 136.2, 135.2, 133.3, 130.5, 128.1, 122.1, 121.3, 111.1, 21.1. HRMS (ESI-TOF) calcd for C_14_H_12_N_3_O [M + H]^+^: 238.0975. Found: 238.0977.

#### 4-(Pyridin-3-yloxy)benzo[d][1,2,3]triazine (**5j**)

Obtained as a white solid (15.9 mg, 0.07 mmol, 74%) from **3a** (30 mg, 0.10 mmol) following the general method. R_*f*_ = 0.3 (Hexane/EtOAc 1:4). Mp 165 °C (decomposition). ^1^H NMR (400 MHz, CDCl_3_) δ 8.69 (d, *J* = 2.7 Hz, 1H), 8.62 (dd, *J* = 4.8, 1.4 Hz, 1H), 8.50 (dt, *J* = 8.5, 0.9 Hz, 1H), 8.43 (ddd, *J* = 8.0, 1.3, 0.7 Hz, 1H), 8.18 (ddd, *J* = 8.4, 7.1, 1.4 Hz, 1H), 8.06 (ddd, *J* = 8.2, 7.1, 1.2 Hz, 1H), 7.80 (ddd, *J* = 8.4, 2.8, 1.4 Hz, 1H), 7.49 (ddd, *J* = 8.4, 4.7, 0.7 Hz, 1H). ^13^C{^1^H} NMR (101 MHz, CDCl_3_) δ: 161.3, 149.0, 147.5, 146.8, 143.5, 135.7, 133.8, 129.9, 128.4, 124.5, 121.9, 110.8. HRMS (ESI-TOF) calcd for C_12_H_9_N_4_O [M + H]^+^: 225.0771. Found: 225.0769.

#### 4-((1H-Indol-5-yl)oxy)benzo[d][1,2,3]triazine (**5k**)

Obtained as a light yellow solid (15.5 mg, 0.06 mmol, 62%) from **3a** (30 mg, 0.1 mmol) following the general method. R_*f*_ = 0.3 (Hexane/EtOAc 3:1). Mp 230 °C (decomposition). ^1^H NMR (400 MHz, CDCl_3_) δ 8.50–8.41 (m, 2H), 8.36 (s, 1H), 8.12 (ddd, *J* = 8.5, 7.3, 1.4 Hz, 1H), 8.01 (ddd, *J* = 8.2, 7.1, 1.1 Hz, 1H), 7.56 (d, *J* = 2.3 Hz, 1H), 7.46 (d, *J* = 8.7 Hz, 1H), 7.31–7.24 (m, 1H), 7.13 (dd, *J* = 8.7, 2.3 Hz, 1H), 6.59 (td, *J* = 2.4, 1.1 Hz, 1H). ^13^C{^1^H} NMR (101 MHz, CDCl_3_) δ: 162.2, 146.5, 146.0, 135.1, 134.1, 133.2, 128.6, 128.1, 125.9, 122.3, 116.0, 112.8, 112.1, 111.3, 103.4. HRMS (ESI-TOF) calcd for C_15_H_11_N_4_O [M + H]^+^: 263.0927. Found: 263.0923.

#### 4-(((R)-2,5,7,8-Tetramethyl-2-((4R,8R)-4,8,12-trimethyltridecyl)chroman-6-yl)oxy)benzo[d][1,2,3]triazine (**5l**)

Obtained as a light yellow solid (37.5 mg, 0.07 mmol, 70%) from **3a** (30 mg, 0.1 mmol) following the general method. R_*f*_ = 0.3 (Hexane/EtOAc 5:1). Mp 155 °C (decomposition). ^1^H NMR (400 MHz, CDCl_3_) δ 8.45 (t, *J* = 6.9 Hz, 2H), 8.12 (t, *J* = 7.8 Hz, 1H), 8.01 (t, *J* = 7.6 Hz, 1H), 2.64 (t, *J* = 6.7 Hz, 2H), 2.14 (s, 3H), 2.01 (s, 3H), 1.97 (s, 3H), 1.90–1.76 (m, 2H), 1.70–1.33 (m, 8H), 1.33–1.19 (m, 11H), 1.18–1.04 (m, 6H), 0.87 (m, 12H). ^13^C{^1^H} NMR (101 MHz, CDCl_3_) δ: 160.9, 150.0, 146.5, 142.0, 135.1, 133.3, 128.2, 126.8, 125.0, 123.7, 122.1, 117.9, 110.7, 75.3, 39.5, 37.63, 37.60, 37.56, 37.53, 37.4, 32.94, 32.92, 28.1, 25.0, 24.9, 24.6, 22.9, 22.8, 21.2, 20.8, 19.9, 19.8, 13.3, 12.4, 12.0. HRMS (ESI-TOF) calcd for C_36_H_53_N_3_O_2_ [M + H]^+^: 560.4211. Found: 560.4212.

#### 4-(Allyloxy)-8-chlorobenzo[d][1,2,3]triazine (**5m**)

Obtained as a light brown solid (13 mg, 0.05 mmol, 60%) from **3b** (30 mg, 0.09 mmol) following the general method. R_*f*_ = 0.3 (Hexane/EtOAc 5:1). Mp 146 °C (decomposition). ^1^H NMR (400 MHz, CDCl_3_) δ 8.27 (dd, *J* = 8.1, 1.3 Hz, 1H), 8.11 (dd, *J* = 7.7, 1.2 Hz, 1H), 7.86 (t, *J* = 8.0 Hz, 1H), 7.50–7.39 (m, 2H), 7.33–7.21 (m, 3H). ^13^C{^1^H} NMR (101 MHz, CDCl_3_) δ: 161.3, 152.1, 143.3, 135.5, 134.0, 133.4, 130.1, 126.7, 121.6, 120.9, 112.7. HRMS (ESI-TOF) calcd for C_13_H_9_ClN_3_O [M + H]^+^: 258.0259. Found: 258.0250.

#### 7-Bromo-4-phenoxybenzo[d][1,2,3]triazine (**5n**)

Obtained as a brown solid (10.2 mg, 0.03 mmol, 44%) from **3c** (30 mg, 0.08 mmol) following the general method. R_*f*_ = 0.3 (Hexane/EtOAc 5:1). Mp 158 °C (decomposition). ^1^H NMR (400 MHz, CDCl_3_) δ 8.62 (d, *J* = 1.8 Hz, 1H), 8.28 (d, *J* = 8.6 Hz, 1H), 8.10 (dd, *J* = 8.7, 1.9 Hz, 1H), 7.56–7.46 (m, 2H), 7.40–7.31 (m, 2H), 7.35–7.28 (m, 1H). ^13^C{^1^H} NMR (101 MHz, CDCl_3_) δ: 161.4, 152.0, 147.1, 137.1, 130.6, 130.1, 130.0, 126.7, 123.8, 121.6, 109.7. HRMS (ESI-TOF) calcd for C_13_H_9_BrN_3_O [M + H]^+^: 301.9924. Found: 301.9922.

#### 5-Bromo-4-phenoxybenzo[d][1,2,3]triazine (**5o**)

Obtained as a light yellow solid (19.7 mg, 0.06 mmol, 85%) from **3d** (30 mg, 0.08 mmol) following the general method. R_*f*_ = 0.3 (Hexane/EtOAc 4:1). Mp 131 °C (decomposition). ^1^H NMR (400 MHz, CDCl_3_) δ 8.32 (dd, *J* = 8.3, 1.1 Hz, 1H), 8.15 (dd, *J* = 7.7, 1.1 Hz, 1H), 7.82 (t, *J* = 8.0 Hz, 1H), 7.47–7.37 (m, 2H), 7.31–7.21 (m, 3H). ^13^C{^1^H} NMR (101 MHz, CDCl_3_) δ: 160.0, 151.8, 147.9, 139.2, 135.3, 130.0, 128.2, 126.5, 121.2. HRMS (ESI-TOF) calcd for C_13_H_9_BrN_3_O [M + H]^+^: 301.9924. Found: 301.9926.

#### 4-Phenoxy-7-(trifluoromethyl)benzo[d][1,2,3]triazine (**5p**)

Obtained as a white solid (23.7. mg, 0.08 mmol, 62%) from **3e** (50 mg, 0.13 mmol) following the general method. R_*f*_ = 0.4 (Hexane/EtOAc 4:1). Mp 198 °C (decomposition). ^1^H NMR (400 MHz, CDCl_3_) δ 8.75 (s, 1H), 8.57 (d, *J* = 8.8 Hz, 1H), 8.20 (d, *J* = 8.6 Hz, 1H), 7.53 (t, *J* = 7.9 Hz, 2H), 7.39 (d, *J* = 7.3 Hz, 1H), 7.34 (d, *J* = 8.1 Hz, 2H). ^13^C{^1^H} NMR (101 MHz, CDCl_3_) δ: 161.2, 151.9, 145.8, 137.0 (q, *J* = 33.8 Hz), 130.2, 129.2 (q, *J* = 3.0 Hz), 126.9, 126.0 (q, *J* = 4.3 Hz), 124.3, 123.9, 121.5, 112.8. ^19^F NMR (376 MHz, CDCl_3_) δ −63.3. HRMS (ESI-TOF) calcd for C_14_H_9_F_3_N_3_O [M + H]^+^: 292.0692. Found: 292.0696.

## Data Availability

The data underlying this study are available in the published article and its Supporting Information.

## Supporting Information Available

^1^H, ^13^C, and ^19^F NMR spectral data, mass spectrometry data of new compounds and computational data (PDF)
