# Supplementary material for: 1,2,3-Benzotriazine Synthesis by Heterocyclization of p-Tosylmethyl Isocyanide Derivatives
Source: J Org Chem. 2023 Sep 18;88(19):14131–9. doi: 10.1021/acs.joc.3c01675 (PMC10563127; doi:10.1021/acs.joc.3c01675)
Supplement: Supplementary file 1 — jo3c01675_si_001.pdf [file jo3c01675_si_001.pdf]

Supporting Information

1,2,3-Benzotriazine Synthesis by Heterocyclization of *p*-Tosylmethyl Isocyanide Derivatives

Francisco Maqueda-Zelaya, José Luis Aceña,* Estíbaliz Merino, Juan J. Vaquero, and David Sucunza*

Departamento de Química Orgánica y Química Inorgánica, Instituto de Investigación Química “Andrés M. del Río” (IQAR), Universidad de Alcalá, IRYCIS, 28805, Alcalá de Henares, Madrid, Spain.

E-mail: jose.acena@uah.es; david.sucunza@uah.es

Table of Contents

General DFT Calculations Details-- S2

Cartesian Coordinates of the Computed Structures --- S3

Copies of ^{1}H, ^{13}C{^{1}H} and ^{19}F-NMR spectra for novel compounds --------------------- S7

DFT Calculations

All reported structures were optimized at Density Functional Theory level as implemented in Gaussian 16.[1] The geometry optimizations were performed using M062X functional[2] with 6-31+G(d,p) basis set for all the atoms. Solvent effects were considered in all the calculations applying the solvation model based on density (SMD)[3] using *N*,*N*-dimethylformamide as solvent at 298.15 K. Reported energy values correspond to Gibbs Free (G) energies in kcal·mol^{-1}. All structures were optimized without geometrical constraint. Stationary points were characterized by frequency calculations (no negative frequency for minima and one negative frequency for transition states).

[1] Gaussian 16, Revision C.01; Frisch, M. J. et al. Gaussian, Inc., Wallingford CT, 2016.
[2] Zhao, Y. & Truhlar, D. G. *Theor. Chem. Acc.* **2008**, *120*, 215-241.
[3] Marenich, A. V.; Cramer, C. J.; Truhlar, D. G. *J. Phys. Chem. B* **2009**, *113*, 6378-6396.

CARTESIAN COORDINATES OF THE COMPUTED STRUCTURES

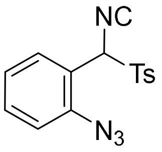

C	-3.87164600	2.04548300	0.08215800
C	-4.69059300	0.92133000	0.18893900
C	-2.52097900	1.88425800	-0.21149600
C	-4.17025500	-0.35540200	0.00095400
H	-5.74543800	1.03494300	0.41569800
C	-1.98478400	0.61104500	-0.40220400
C	-2.81322300	-0.51623900	-0.29170800
H	-4.81986100	-1.22195600	0.07854000
H	-4.28103000	3.03888600	0.22604200
N	-2.19821100	-1.77323200	-0.51075800
N	-2.92123000	-2.76993900	-0.39153700
N	-3.49029800	-3.73782600	-0.30415800
H	-1.87142500	2.74994500	-0.29918600
C	-0.51777500	0.42406500	-0.67381700
H	-0.30437500	-0.56221400	-1.09453000
S	0.39420000	0.46522500	0.94885600
O	0.33745300	1.84241400	1.44292400
O	-0.17978100	-0.62316000	1.74124100
C	2.06178200	0.07119000	0.51345100
C	2.42015800	-1.26911900	0.37617000
C	2.96674000	1.10541100	0.27959800
C	3.72313900	-1.57241000	-0.00319900
H	1.69679200	-2.05478200	0.57122900
C	4.26445900	0.77793900	-0.09816800
H	2.65821900	2.13901700	0.39808100
C	4.65988100	-0.55835600	-0.24283300
H	4.01879600	-2.61161900	-0.11169600
H	4.98212700	1.57174300	-0.28299900
C	6.07655600	-0.89784200	-0.62143200
H	6.49819600	-0.14043600	-1.28587700
H	6.70529800	-0.94181300	0.27394800
H	6.13087400	-1.86984700	-1.11585800
N	0.03775300	1.42782500	-1.49535300
C	0.52440100	2.26909700	-2.14904000

E(RM062X) = -1345.93685277
Zero-point correction= 0.250591 (Hartree/Particle)
Thermal correction to Energy= 0.270614
Thermal correction to Enthalpy= 0.271559
Thermal correction to Gibbs Free Energy= 0.199401
Sum of electronic and zero-point Energies= -1345.686262
Sum of electronic and thermal Energies= -1345.666238
Sum of electronic and thermal Enthalpies= -1345.665294
Sum of electronic and thermal Free Energies= -1345.737452

^{t}BuOK

C	1.12756700	0.00019300	0.00003500
O	-0.24959900	-0.00001600	-0.00014000
K	-2.69451900	-0.00022500	-0.00006300
C	1.67839000	-0.95016700	1.08526900
H	2.77555900	-0.97436900	1.10985700
H	1.31827200	-0.63224400	2.07093400
H	1.31458500	-1.96824000	0.90260500
C	1.67729800	1.41546900	0.28011300
H	2.77440500	1.45116900	0.28466600
H	1.31197400	2.10988200	-0.48565400
H	1.31653000	1.76402000	1.25510300
C	1.67875700	-0.46493200	-1.36524300
H	2.77587700	-0.47524600	-1.39798400
H	1.31729700	-1.47692000	-1.58279800
H	1.31608300	0.20298100	-2.15545200

E(RM062X) = -832.912362459
Zero-point correction= 0.123398 (Hartree/Particle)
Thermal correction to Energy= 0.131904
Thermal correction to Enthalpy= 0.132848
Thermal correction to Gibbs Free Energy= 0.088399
Sum of electronic and zero-point Energies= -832.788964
Sum of electronic and thermal Energies= -832.780458
Sum of electronic and thermal Enthalpies= -832.779514
Sum of electronic and thermal Free Energies= -832.823964

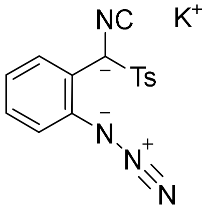

C	4.62863100	-0.13069800	-0.44000300
C	4.52155800	1.25472500	-0.30764900
C	3.49265300	-0.93199100	-0.37776500
C	3.26293500	1.83153900	-0.15814000
H	5.40404700	1.88445000	-0.34197500
C	2.21204500	-0.38150600	-0.19401300
C	2.12712500	1.02374000	-0.12678900
H	3.15933800	2.91202000	-0.10156100
H	5.60214600	-0.59133300	-0.57481700
N	0.80466000	1.56996500	-0.12018400
N	0.65896400	2.66240800	0.43403100
N	0.41804000	3.65184300	0.92219500
H	3.58754600	-2.01110500	-0.45443600
C	1.01514200	-1.22507100	-0.11149500
S	-0.01562600	-1.13373600	1.28683000
O	-0.28759700	-2.49674600	1.78617200
O	0.50566400	-0.13103700	2.23372900
C	-1.61137200	-0.52277000	0.74586000
C	-2.02306200	0.76271100	1.07769100
C	-2.41626700	-1.35285500	-0.03935400
C	-3.25035400	1.22931500	0.60299800
H	-1.39314000	1.38339300	1.70674900
C	-3.63356400	-0.87180400	-0.50882400
H	-2.09072100	-2.36162600	-0.27872600
C	-4.06836300	0.42600500	-0.19644600
H	-3.57426200	2.23330100	0.86234600
H	-4.26148200	-1.51296200	-1.12163000
C	-5.39810400	0.92203600	-0.70123500
H	-5.48391800	0.77696800	-1.78170800
H	-6.21731800	0.36957500	-0.23072200
H	-5.53329100	1.98296500	-0.48237900
C	1.16122600	-3.57155700	-1.10740800

N	1.09826700	-2.49984200	-0.62996800
K	-0.61443900	0.29657200	-2.27035400

E(RM062X) = -1345.45266840
Zero-point correction= 0.237191 (Hartree/Particle)
Thermal correction to Energy= 0.257041
Thermal correction to Enthalpy= 0.257985
Thermal correction to Gibbs Free Energy= 0.185445
Sum of electronic and zero-point Energies= -1345.215478
Sum of electronic and thermal Energies= -1345.195627
Sum of electronic and thermal Enthalpies= -1345.194683
Sum of electronic and thermal Free Energies= -1345.267224

*t*BuOH

C	0.00622000	-0.00000300	0.01491600
O	-0.06591400	-0.00064800	1.44900400
C	1.49411100	-0.00477400	-0.31176000
H	1.64658900	-0.00463800	-1.39462000
H	1.97677900	0.88224200	0.10922300
H	1.97092500	-0.89542300	0.10820600
C	-0.66513400	1.26081300	-0.52861700
H	-0.59093500	1.29971300	-1.61959800
H	-1.72725500	1.27869000	-0.26016600
H	-0.18609300	2.15235900	-0.11330400
C	-0.67361000	-1.25560400	-0.53013500
H	-0.60070800	-1.29311000	-1.62125400
H	-0.19984500	-2.15088800	-0.11679700
H	-1.73556800	-1.26713700	-0.26063500
H	-0.99609400	0.00078200	1.71049900

E(RM062X) = -233.581134065
Zero-point correction= 0.136537 (Hartree/Particle)
Thermal correction to Energy= 0.143172
Thermal correction to Enthalpy= 0.144116
Thermal correction to Gibbs Free Energy= 0.107647
Sum of electronic and zero-point Energies= -233.444597
Sum of electronic and thermal Energies= -233.437962
Sum of electronic and thermal Enthalpies= -233.437018
Sum of electronic and thermal Free Energies= -233.473487

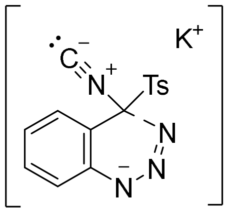

C	4.09143600	-2.07233000	0.17957200
C	2.71085300	-2.12452800	0.08805100
C	1.94563400	-1.00822700	-0.31493300
C	2.64520100	0.15560300	-0.72186400
C	4.04569200	0.20526400	-0.59904500
C	4.76433200	-0.88342900	-0.13642700
H	4.64456200	-2.94300000	0.51662700
H	2.18571200	-3.03609700	0.35877200
H	4.54914500	1.11682500	-0.90872700
H	5.84360000	-0.82023900	-0.04229500
C	0.47025000	-1.08763000	-0.30540000
N	2.07722300	1.30377000	-1.34427900
N	0.96483500	1.01489500	-1.89693700
N	0.01785500	0.37589300	-2.02175300
S	-0.33445500	-0.19581300	0.97306800
O	-0.34619000	-0.93668200	2.25087400
O	0.26263600	1.15765600	0.99749800
C	-2.03495100	-0.03288900	0.47271500
C	-2.44480200	1.11214800	-0.20790600
C	-2.93164200	-1.05608600	0.77341600
C	-3.77640800	1.22473900	-0.59439100
H	-1.73114100	1.90025500	-0.42324400
C	-4.25853800	-0.92961100	0.37032100
H	-2.59688900	-1.93214800	1.31943800
C	-4.69937300	0.20874600	-0.31433300
H	-4.10558900	2.11560500	-1.12194500
H	-4.96273000	-1.72508700	0.59669600
C	-6.14384100	0.35664800	-0.71509000
H	-6.23654100	0.89926000	-1.65885700
H	-6.62558500	-0.61750500	-0.82275600
H	-6.69406500	0.91960000	0.04613100
C	-0.60525700	-3.39179600	-0.56504400
N	-0.08808300	-2.34569900	-0.42353200
K	2.01446900	3.10452600	0.83851300

E(RM062X) = -1945.26994106
Zero-point correction= 0.237109 (Hartree/Particle)
Thermal correction to Energy= 0.258173
Thermal correction to Enthalpy= 0.259118
Thermal correction to Gibbs Free Energy= 0.184741
Sum of electronic and zero-point Energies= -1945.032832
Sum of electronic and thermal Energies= -1945.011768
Sum of electronic and thermal Enthalpies= -1945.010823
Sum of electronic and thermal Free Energies= -1945.085200
Frequency= -385.4500

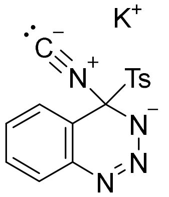

C	-4.52418900	-1.34368900	0.45875600
C	-3.18011400	-1.28816300	0.79976500
C	-2.40800600	-0.19854100	0.38337100
C	-2.97048200	0.84577900	-0.35836500
C	-4.33305100	0.77715100	-0.69933200
C	-5.09488800	-0.30891400	-0.30034200
H	-5.12971000	-2.18741300	0.77170300
H	-2.71927300	-2.08466800	1.37679100
H	-4.76697100	1.59518600	-1.26610300
H	-6.14540400	-0.35670300	-0.56940100
C	-0.94679000	-0.08031900	0.60950500
N	-2.25556100	1.99069800	-0.70929100
N	-1.06959100	2.12579200	-0.22382800
N	-0.41360400	1.23087800	0.47666900
S	-0.08878800	-1.20373600	-0.73874900
O	-0.40456000	-2.61690000	-0.46237900
O	-0.49625200	-0.62072700	-2.02865400
C	1.65339300	-0.95344300	-0.52639700
C	2.26971600	0.11802000	-1.17320600

C	2.36419200	-1.80581100	0.31572000
C	3.62789400	0.33185800	-0.96601800
H	1.69148200	0.75867800	-1.83150200
C	3.72360800	-1.57454100	0.50919300
H	1.86229300	-2.63724200	0.80020900
C	4.37182400	-0.50861300	-0.12558400
H	4.12178800	1.15978800	-1.46708300
H	4.28966700	-2.23455500	1.15991300
C	5.84837100	-0.28145400	0.06306800
H	6.21163400	-0.77217000	0.96819000
H	6.40584300	-0.68782400	-0.78716000
H	6.07647400	0.78527500	0.12674500
C	-0.10628600	-1.16479200	2.81069300
N	-0.50359900	-0.66959900	1.82676500
K	1.29761100	3.35215200	0.63519900

E(RM062X) = -1945.32374770
Zero-point correction=0.239015 (Hartree/Particle)
Thermal correction to Energy= 0.260176
Thermal correction to Enthalpy= 0.261120
Thermal correction to Gibbs Free Energy= 0.186236
Sum of electronic and zero-point Energies= -1945.084733
Sum of electronic and thermal Energies= -1945.063572
Sum of electronic and thermal Enthalpies= -1945.062627
Sum of electronic and thermal Free Energies= -1945.137512

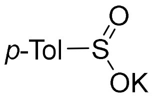

S	1.21638400	0.44889400	1.08309400
O	1.80252200	1.36106600	-0.00633600
O	1.81340400	-0.96003400	0.94740700
C	-0.48184800	0.19486100	0.48615000
C	-1.08972200	-1.05011700	0.61275200
C	-1.19761800	1.27524300	-0.03184200
C	-2.41403500	-1.21814000	0.20241400
H	-0.51921500	-1.88394600	1.01085200
C	-2.51782800	1.09820500	-0.43621000
H	-0.71319400	2.24220700	-0.13480700
C	-3.14659700	-0.14984200	-0.32318100
H	-2.88368100	-2.19459700	0.29057900
H	-3.07145900	1.93794100	-0.84972200
C	-4.58212600	-0.32132300	-0.75171200
H	-5.25420700	0.23254300	-0.08830800
H	-4.73717400	0.05983200	-1.76516800
H	-4.87775000	-1.37257700	-0.73094300
K	3.48661900	-0.44133100	-1.11267200

E(RM062X) = -1419.35537766
Zero-point correction= 0.126959 (Hartree/Particle)
Thermal correction to Energy= 0.138571
Thermal correction to Enthalpy= 0.139515
Thermal correction to Gibbs Free Energy= 0.085920
Sum of electronic and zero-point Energies= -1419.228419
Sum of electronic and thermal Energies= -1419.216806
Sum of electronic and thermal Enthalpies= -1419.215862
Sum of electronic and thermal Free Energies= -1419.269458

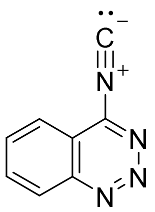

C	0.83329400	0.84428500	0.00019400
C	0.03084700	-0.31445500	-0.00012000
C	-1.35402100	-0.02231200	-0.00038800
H	2.84266500	1.63013500	0.00066400
C	2.24206000	0.72729600	0.00043900
C	0.61682800	-1.59848800	-0.00015200
C	1.98886600	-1.69113100	0.00009500
C	2.80201900	-0.52757800	0.00038300
H	-0.01054600	-2.48322000	-0.00038700
H	2.46189900	-2.66703600	0.00006200
H	3.88090300	-0.63800700	0.00056600
N	0.27295300	2.09450900	0.00023500
N	-0.99756600	2.24586500	0.00023200
N	-1.84516800	1.19133500	-0.00017800
N	-2.27312200	-1.05645600	-0.00039500
C	-3.03899100	-1.94572200	-0.00047700

E(RM062X) = -525.979432307
Zero-point correction= 0.109922 (Hartree/Particle)
Thermal correction to Energy= 0.118351
Thermal correction to Enthalpy= 0.119295
Thermal correction to Gibbs Free Energy= 0.076085
Sum of electronic and zero-point Energies= -525.869510
Sum of electronic and thermal Energies= -525.861082
Sum of electronic and thermal Enthalpies= -525.860138
Sum of electronic and thermal Free Energies= -525.903347

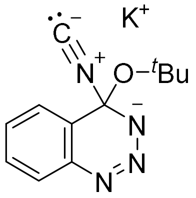

C	1.89939300	0.93142200	-0.62685700
C	1.54277500	-0.05902700	0.29457300
C	0.06522800	-0.25069800	0.43358700
H	3.53570600	1.85472700	-1.67698900
C	3.26092300	1.08423500	-0.96245300
C	2.51316000	-0.85163200	0.91466500
C	3.85137000	-0.68235600	0.58904900
C	4.21871600	0.28347700	-0.36502200
H	2.21074000	-1.60808200	1.63405300
H	4.60923500	-1.30166100	1.05732500
H	5.26503700	0.40996300	-0.62633400
N	0.97696700	1.83060100	-1.14807400
N	-0.19314300	1.86521800	-0.57684400
N	-0.64849200	1.00204100	0.28162000
N	-0.26082500	-0.75293700	1.75657300
C	-0.48749200	-1.11464200	2.84460700
O	-0.28601600	-1.20185000	-0.55145900
C	-1.58389100	-1.85237000	-0.65604700
C	-1.54339900	-3.15873600	0.14058900

H	-1.51955800	-2.97823200	1.21676400
H	-2.43196700	-3.75617600	-0.08556400
H	-0.65720900	-3.73563400	-0.13970400
C	-1.69447900	-2.16117300	-2.14737700
H	-1.69110000	-1.23278500	-2.72574000
H	-0.85076100	-2.77825100	-2.46959000
H	-2.62233300	-2.70244800	-2.35402100
C	-2.76438500	-0.98462300	-0.22092600
H	-2.83532600	-0.08609700	-0.83791600
H	-3.68004300	-1.57065900	-0.34976000
H	-2.69297500	-0.68575600	0.82705300
K	-2.58633600	2.89832500	0.38520100

E(RM062X) = -1358.93321509
Zero-point correction= 0.237558 (Hartree/Particle)
Thermal correction to Energy= 0.255022
Thermal correction to Enthalpy= 0.255966
Thermal correction to Gibbs Free Energy= 0.191143
Sum of electronic and zero-point Energies= -1358.695657
Sum of electronic and thermal Energies= -1358.678193
Sum of electronic and thermal Enthalpies= -1358.677249
Sum of electronic and thermal Free Energies= -1358.742072

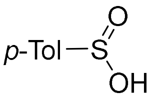

S	2.14837400	-0.06471200	-0.39701600
O	2.47936500	-1.12348800	0.82611800
O	2.60436800	1.30034900	0.00311900
C	0.36073300	-0.02886900	-0.20057600
C	-0.27473100	1.18357300	0.03534300
C	-0.36415600	-1.21397800	-0.34166800
C	-1.66469400	1.20428200	0.15619600
H	0.31380200	2.09085800	0.12764200
C	-1.74760700	-1.17444500	-0.21998900
H	0.14459200	-2.15429400	-0.53588400
C	-2.41681300	0.03287200	0.03266100
H	-2.16993500	2.14705800	0.34541700
H	-2.32099000	-2.09167200	-0.32308700
C	-3.91645400	0.05156300	0.17339700
H	-4.39324000	-0.45928000	-0.66764200
H	-4.22242100	-0.46674200	1.08757000
H	-4.29691100	1.07385600	0.21863200
H	2.04357700	-0.84926500	1.65352700

E(RM062X) = -819.968868178
Zero-point correction= 0.137864 (Hartree/Particle)
Thermal correction to Energy= 0.147864
Thermal correction to Enthalpy= 0.148808
Thermal correction to Gibbs Free Energy= 0.100701
Sum of electronic and zero-point Energies= -819.831004
Sum of electronic and thermal Energies= -819.821005
Sum of electronic and thermal Enthalpies= -819.820060
Sum of electronic and thermal Free Energies= -819.868167

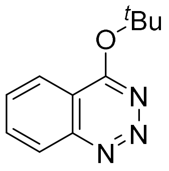

C	2.07722700	0.71061200	-0.00006800
C	1.07956800	-0.27471700	-0.00007000
C	-0.25314300	0.24500000	-0.00022500
H	4.19559600	1.11317800	0.00000400
C	3.43859700	0.33614200	0.00000900
C	1.42131100	-1.64274100	-0.00002000
C	2.75393900	-1.99373700	0.00004300
C	3.76410100	-1.00163000	0.00006000
H	0.63720500	-2.39126900	-0.00007600
H	3.03682700	-3.04096600	0.00004600
H	4.80589700	-1.30428600	0.00012000
N	1.75767800	2.05233100	-0.00017200
N	0.53620300	2.41833000	0.00011500
N	-0.49426300	1.53781200	-0.00002400
O	-1.22354200	-0.65332000	0.00027100
C	-2.65792600	-0.32625800	0.00007200
C	-3.29900400	-1.70895100	-0.00064300
H	-2.99876100	-2.26986500	0.88865200
H	-4.38714700	-1.60819800	-0.00045100
H	-2.99899000	-2.26884600	-0.89068200
C	-3.02338800	0.43401900	-1.27038500
H	-2.60997400	1.44244500	-1.26873700
H	-2.66050700	-0.10589400	-2.15004000
H	-4.11320900	0.50075700	-1.33549900
C	-3.02335500	0.43321100	1.27099900
H	-2.60871500	1.44118100	1.27048500
H	-4.11316000	0.50131100	1.33561800
H	-2.66162000	-0.10799800	2.15031800

E(RM062X) = -666.192939935
Zero-point correction= 0.229192 (Hartree/Particle)
Thermal correction to Energy= 0.242366
Thermal correction to Enthalpy= 0.243310
Thermal correction to Gibbs Free Energy= 0.189603
Sum of electronic and zero-point Energies= -665.963748
Sum of electronic and thermal Energies= -665.950574
Sum of electronic and thermal Enthalpies= -665.949629
Sum of electronic and thermal Free Energies= -666.003337

HCN

N	0.00000000	0.00000000	0.65298600
C	0.00000000	0.00000000	-0.49955700
H	0.00000000	0.00000000	-1.57355900

E(RM062X) = -93.3974012158
Zero-point correction= 0.016697 (Hartree/Particle)
Thermal correction to Energy= 0.019211
Thermal correction to Enthalpy= 0.020155
Thermal correction to Gibbs Free Energy= -0.002663
Sum of electronic and zero-point Energies= -93.380704
Sum of electronic and thermal Energies= -93.378190
Sum of electronic and thermal Enthalpies= -93.377246
Sum of electronic and thermal Free Energies= -93.400064

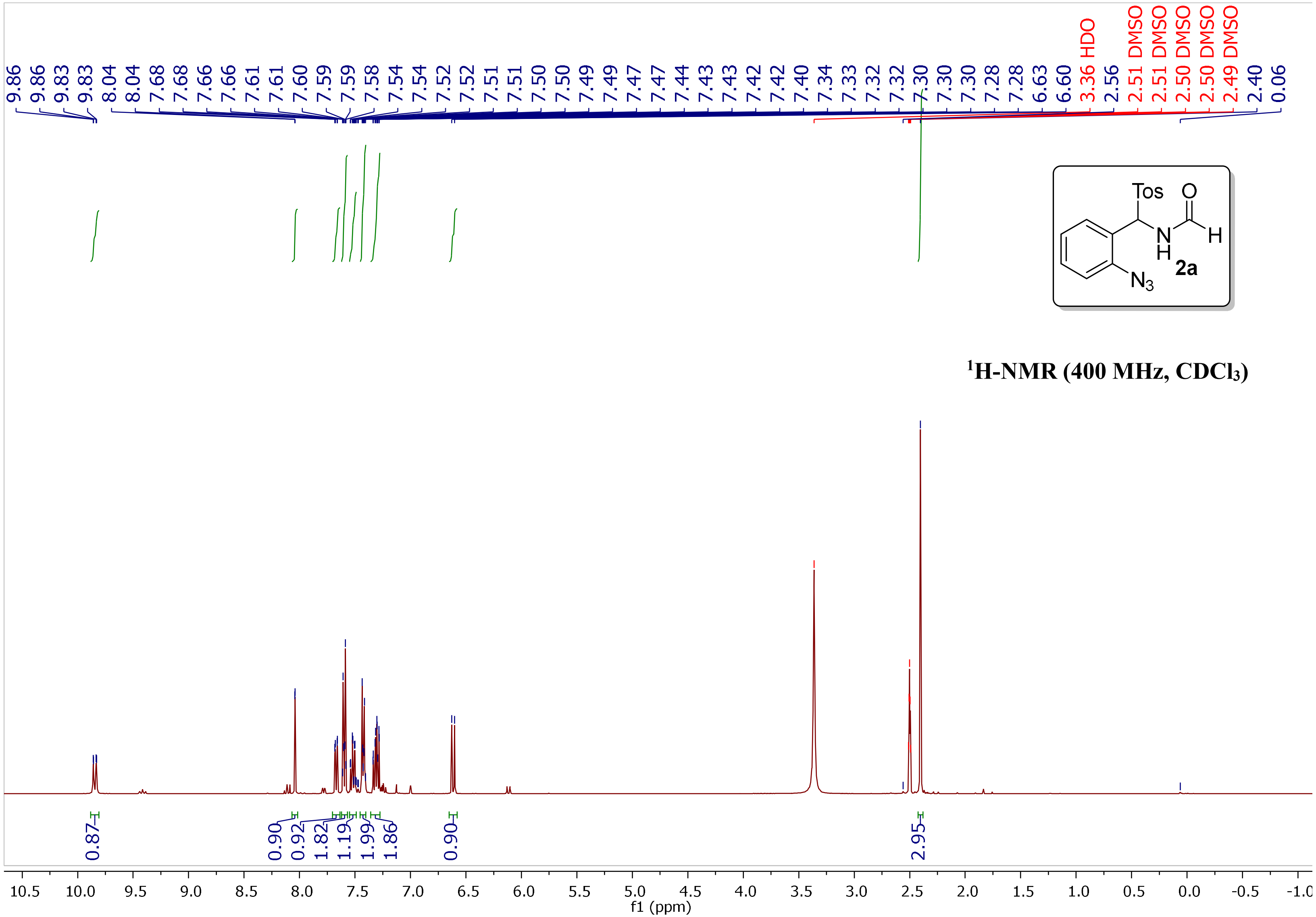

9.86 9.86 9.83 9.83 8.04 8.04 7.68 7.68 7.66 7.66 7.61 7.61 7.60 7.59 7.59 7.58 7.54 7.54 7.52 7.52 7.51 7.51 7.50 7.50 7.49 7.49 7.47 7.47 7.44 7.43 7.43 7.42 7.42 7.40 7.34 7.33 7.32 7.32 7.30 7.30 7.30 7.28 7.28 6.63 6.60
3.36 HDO
2.56
2.51 DMSO
2.51 DMSO
2.50 DMSO
2.50 DMSO
2.49 DMSO
2.40 0.06
Tos
O
N
H
H
N3
2a
¹H-NMR (400 MHz, CDCl3)
0.87 0.90 0.92 1.82 1.19 1.99 1.86 0.90 2.95
10.5 10.0 9.5 9.0 8.5 8.0 7.5 7.0 6.5 6.0 5.5 5.0 4.5 4.0 3.5 3.0 2.5 2.0 1.5 1.0 0.5 0.0 -0.5 -1.0
f1 (ppm)

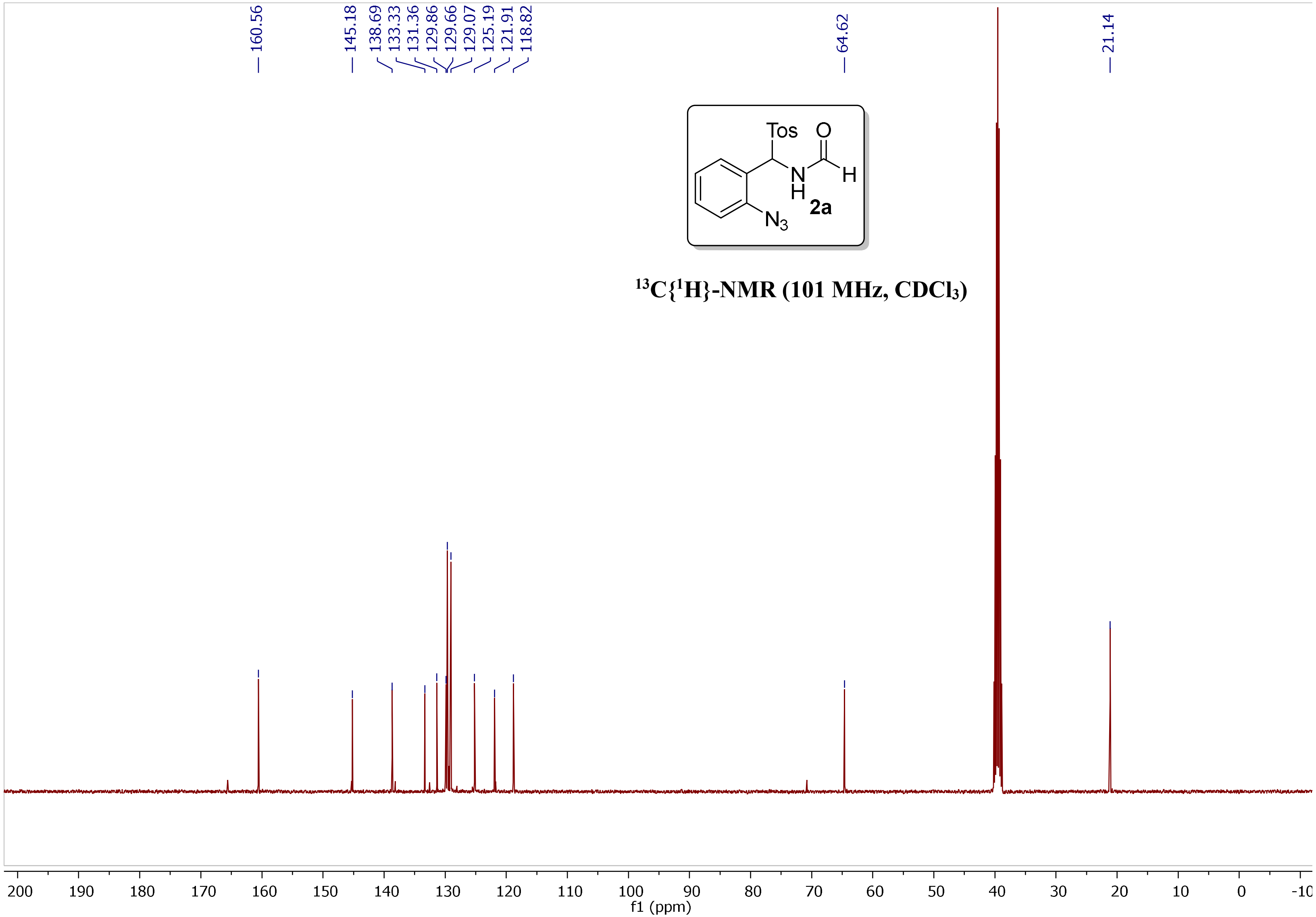

160.56
145.18
138.69
133.33
131.36
129.86
129.66
129.07
125.19
121.91
118.82
64.62
21.14
Tos
O
N
H
H
N3
2a
13C{1H}-NMR (101 MHz, CDCl3)
200
190
180
170
160
150
140
130
120
110
100
90
80
70
60
50
40
30
20
10
0
-10
f1 (ppm)

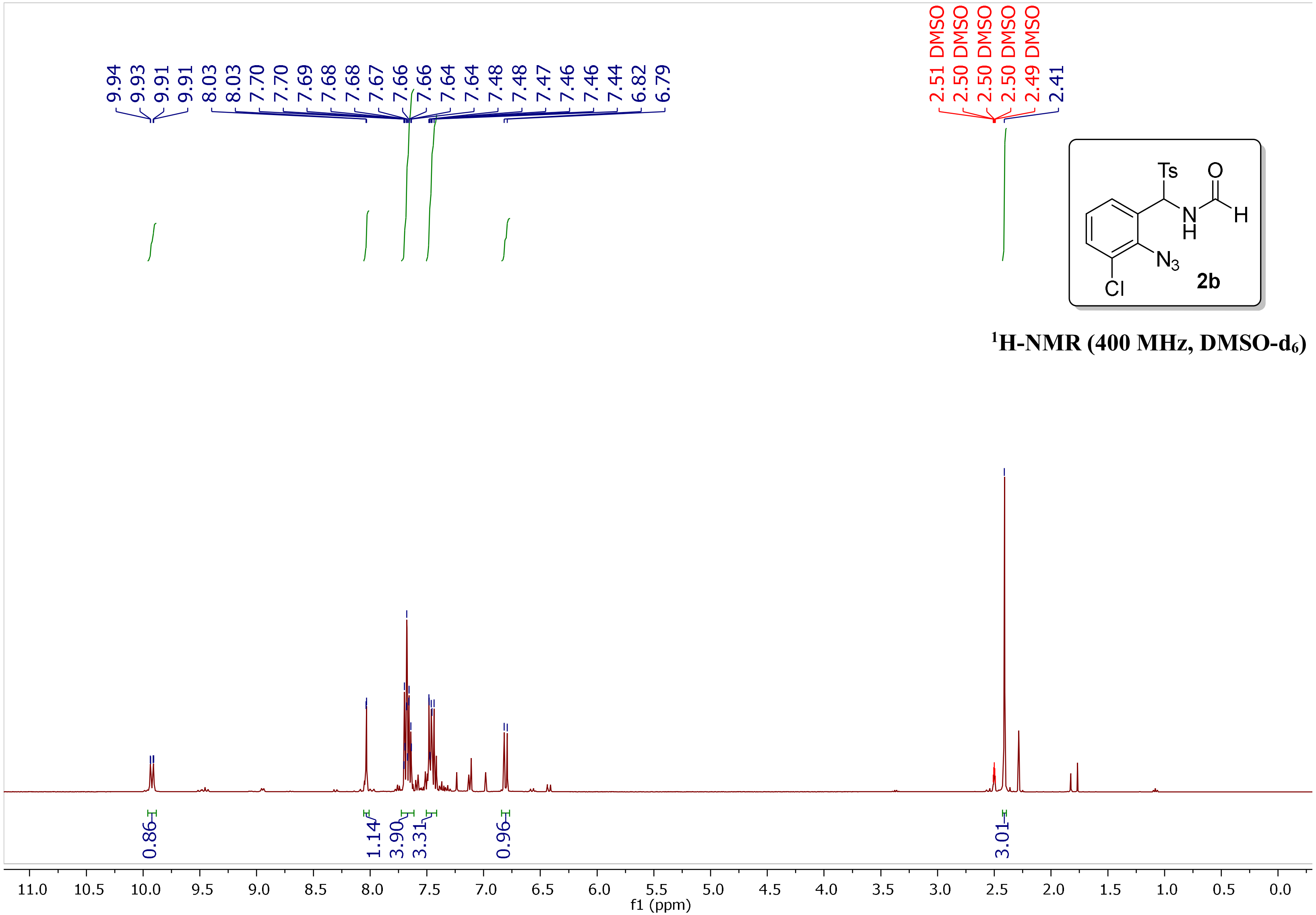

^{1}H-NMR (400 MHz, DMSO-d$_6$)

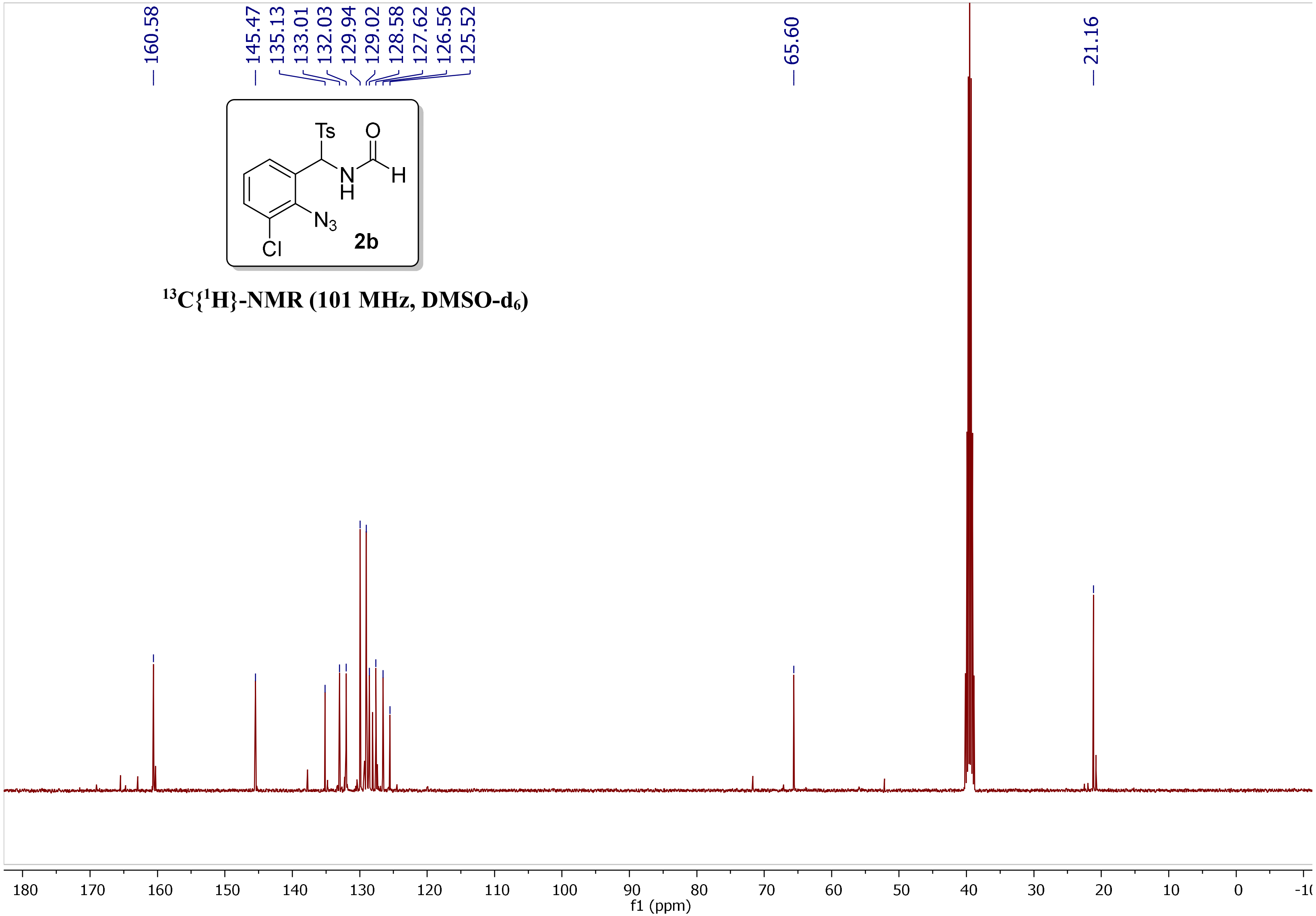
160.58
145.47
135.13
133.01
132.03
129.94
129.02
128.58
127.62
126.56
125.52
65.60
21.16
Ts
O
N
H
H
N3
Cl
2b
13C{1H}-NMR (101 MHz, DMSO-d6)
180
170
160
150
140
130
120
110
100
90
80
70
60
50
40
30
20
10
0
f1 (ppm)

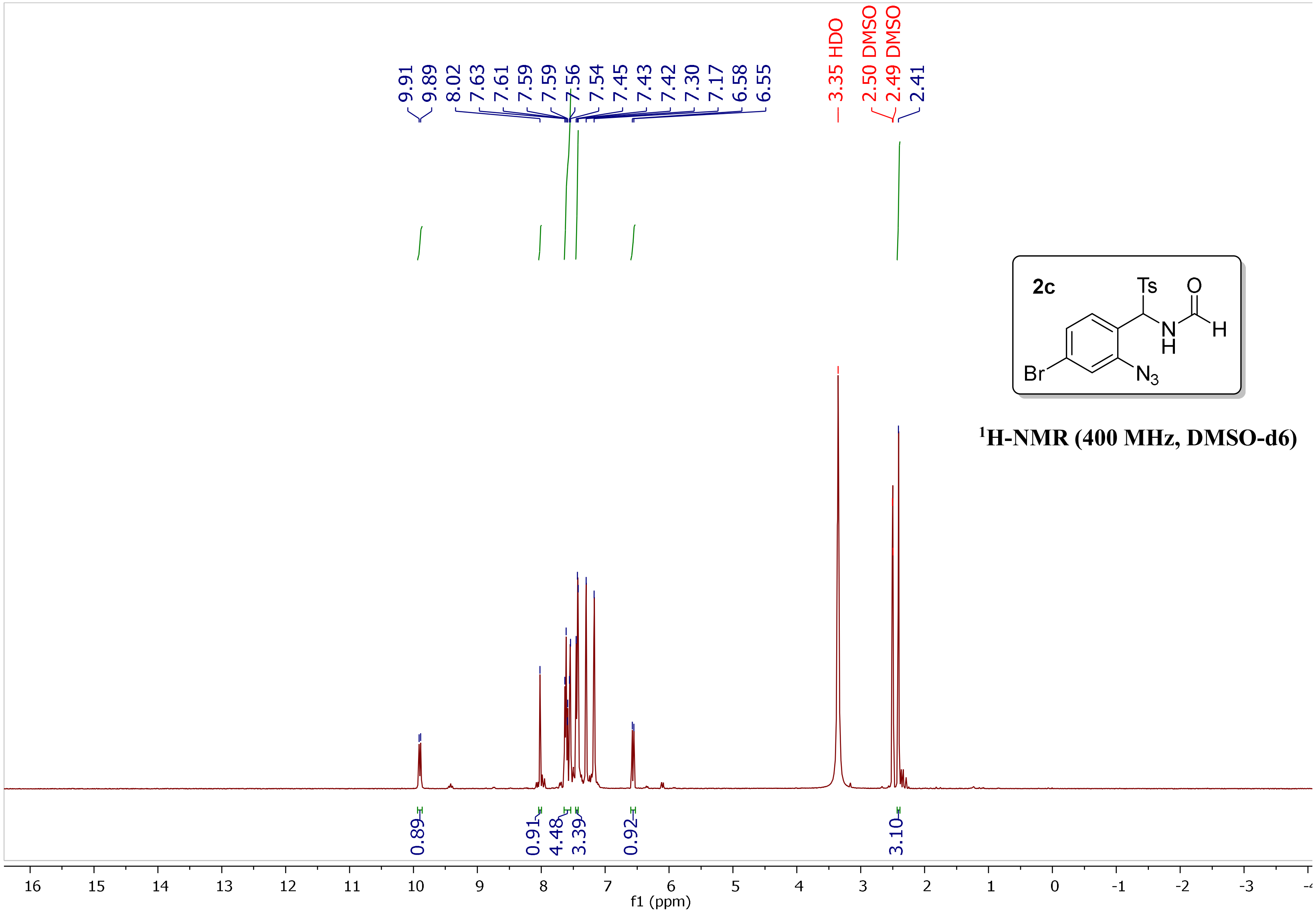

^{1}H-NMR (400 MHz, DMSO-d6)

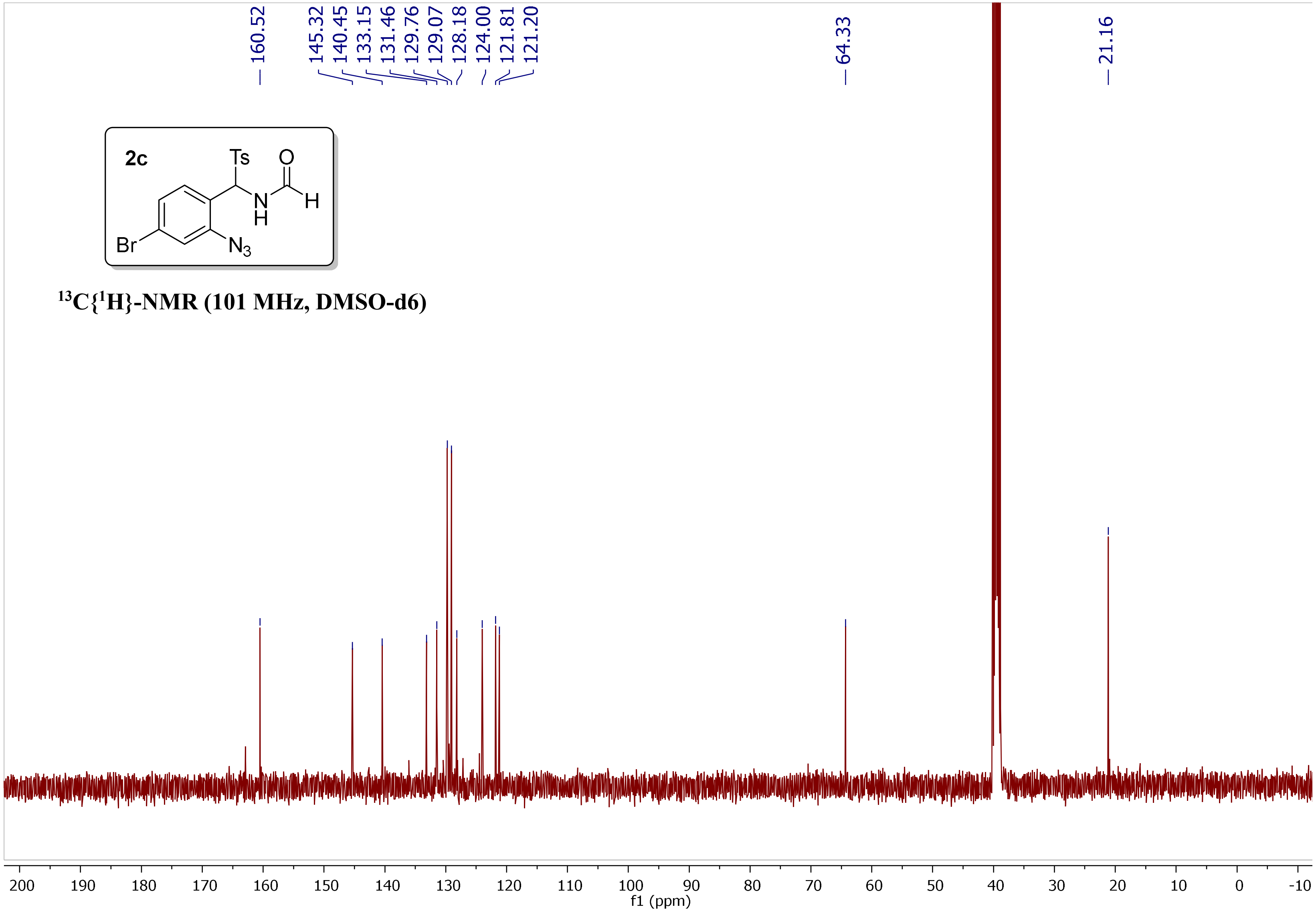

160.52
145.32
140.45
133.15
131.46
129.76
129.07
128.18
124.00
121.81
121.20
64.33
21.16
2c
Ts
O
N
H
H
Br
N3
13C{1H}-NMR (101 MHz, DMSO-d6)
200 190 180 170 160 150 140 130 120 110 100 90 80 70 60 50 40 30 20 10 0 -10
f1 (ppm)

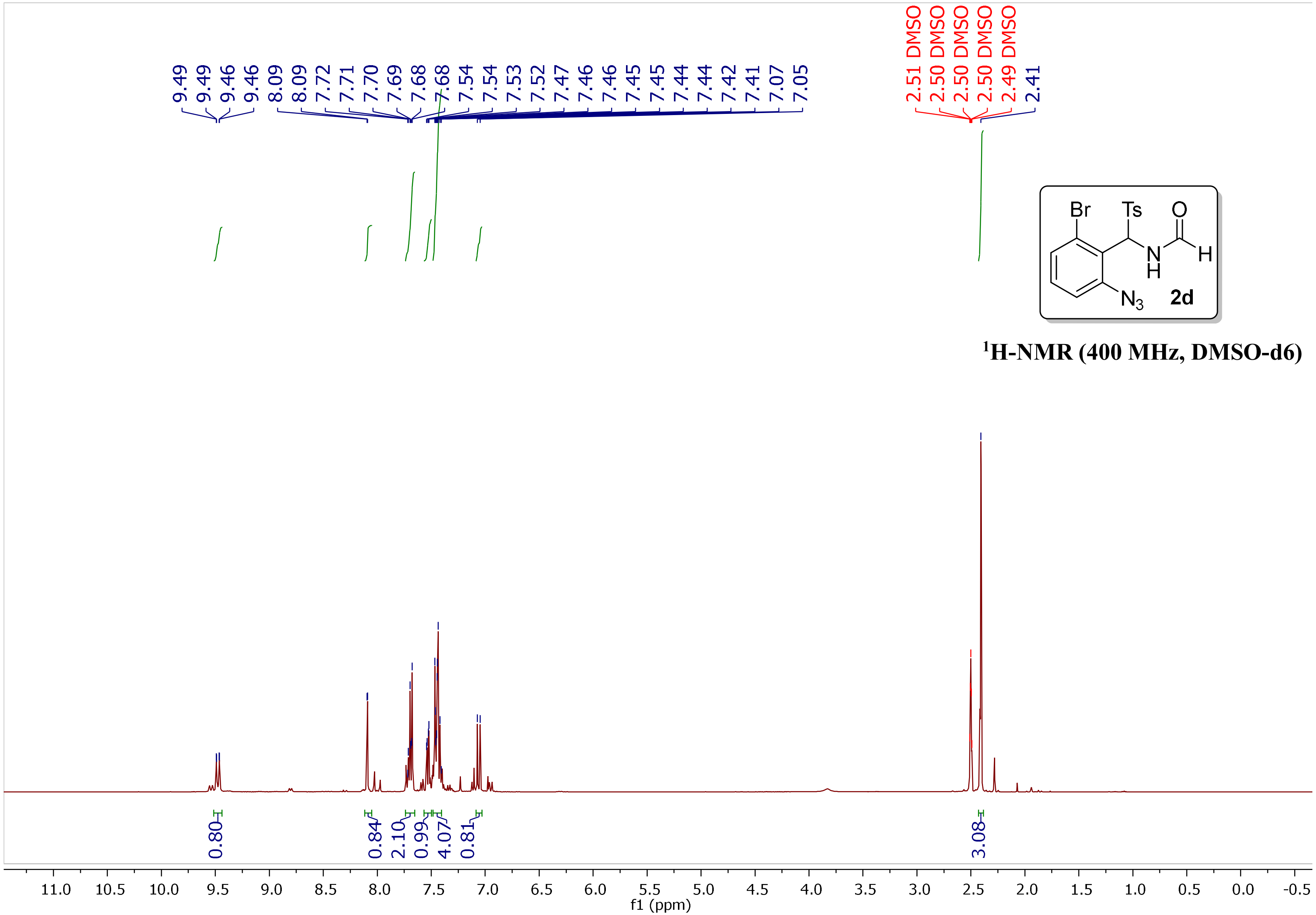

^{1}H-NMR (400 MHz, DMSO-d6)

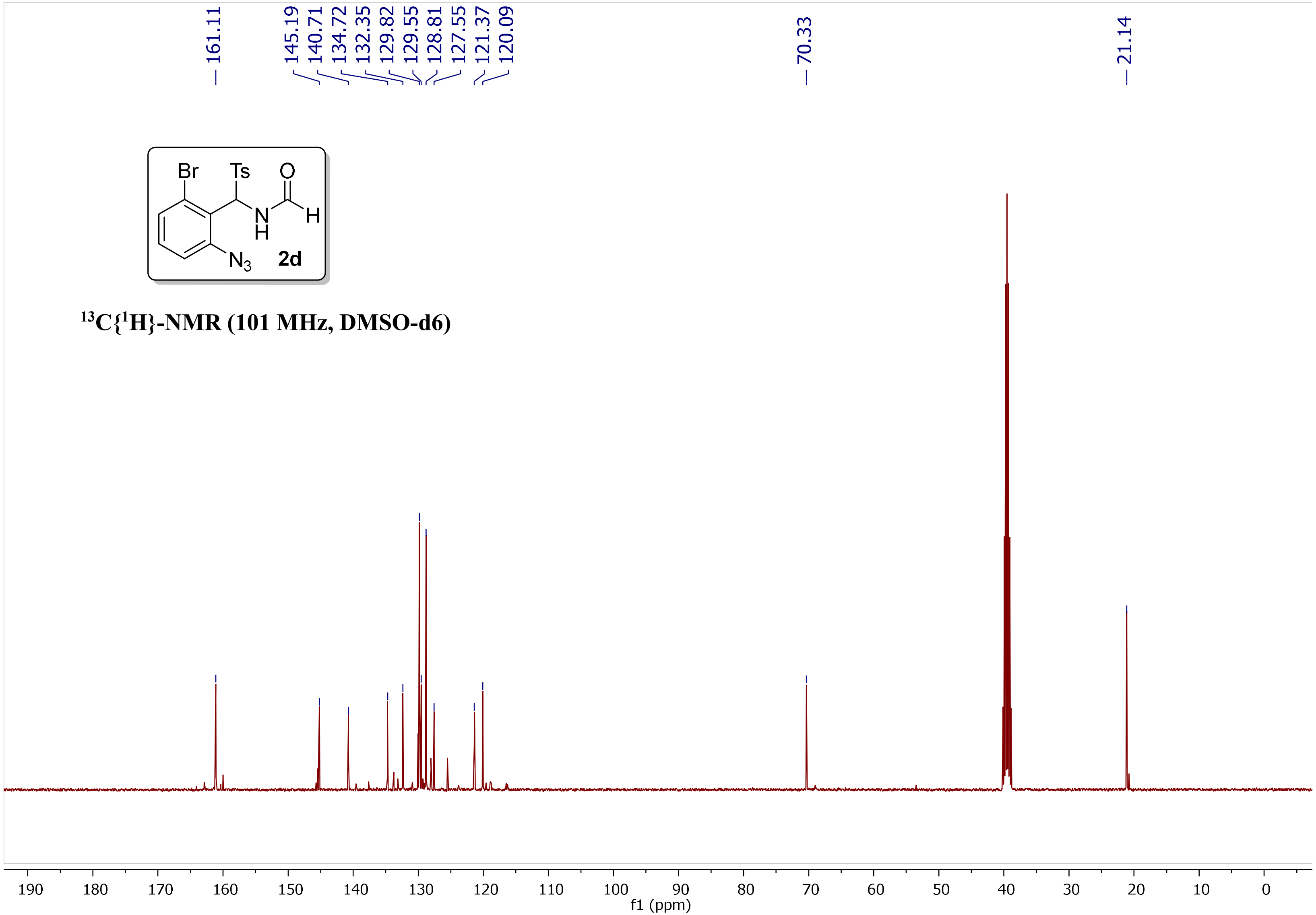

$^{13}C\{^{1}H\}$-NMR (101 MHz, DMSO-d6)

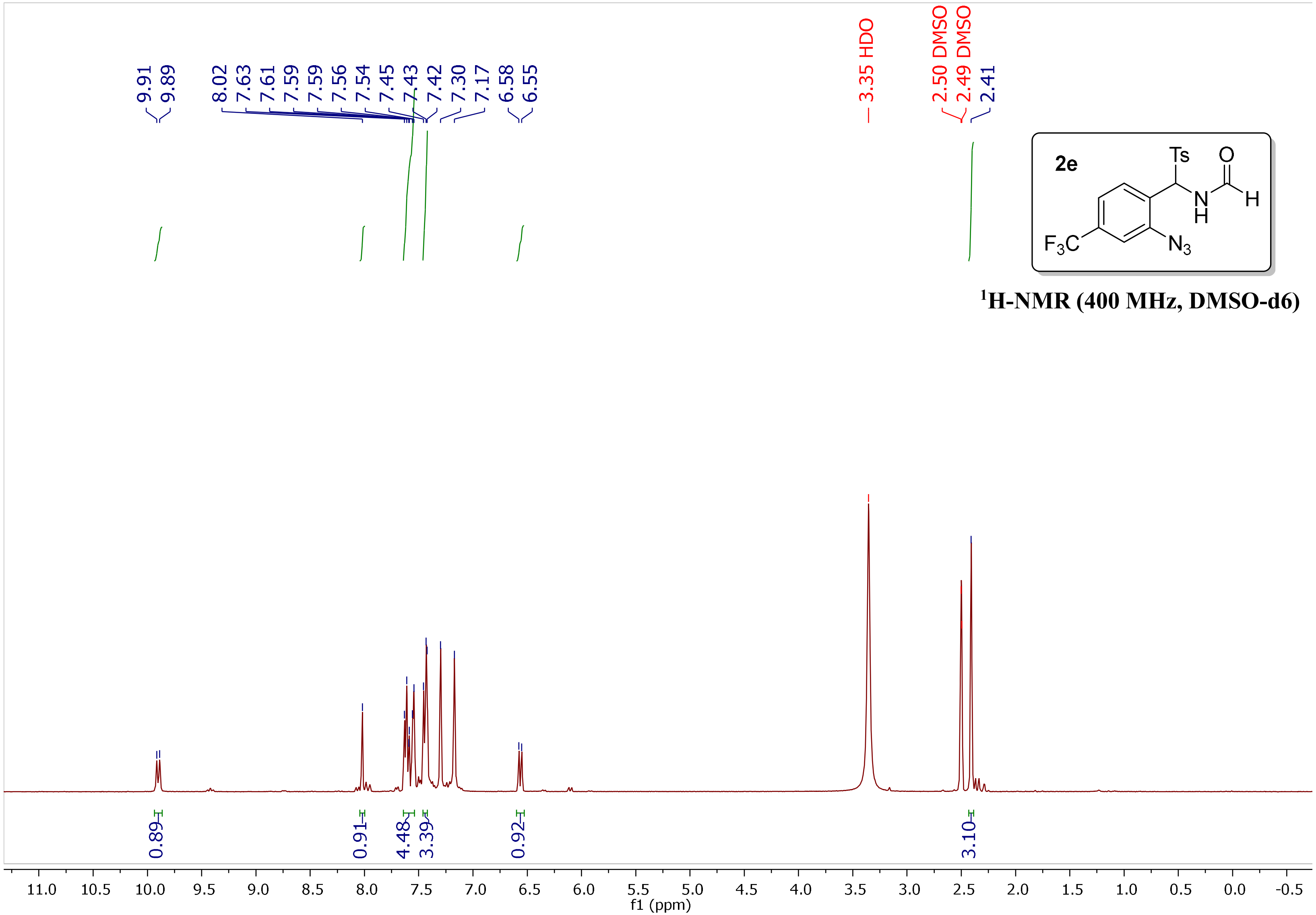

^{1}H-NMR (400 MHz, DMSO-d6)

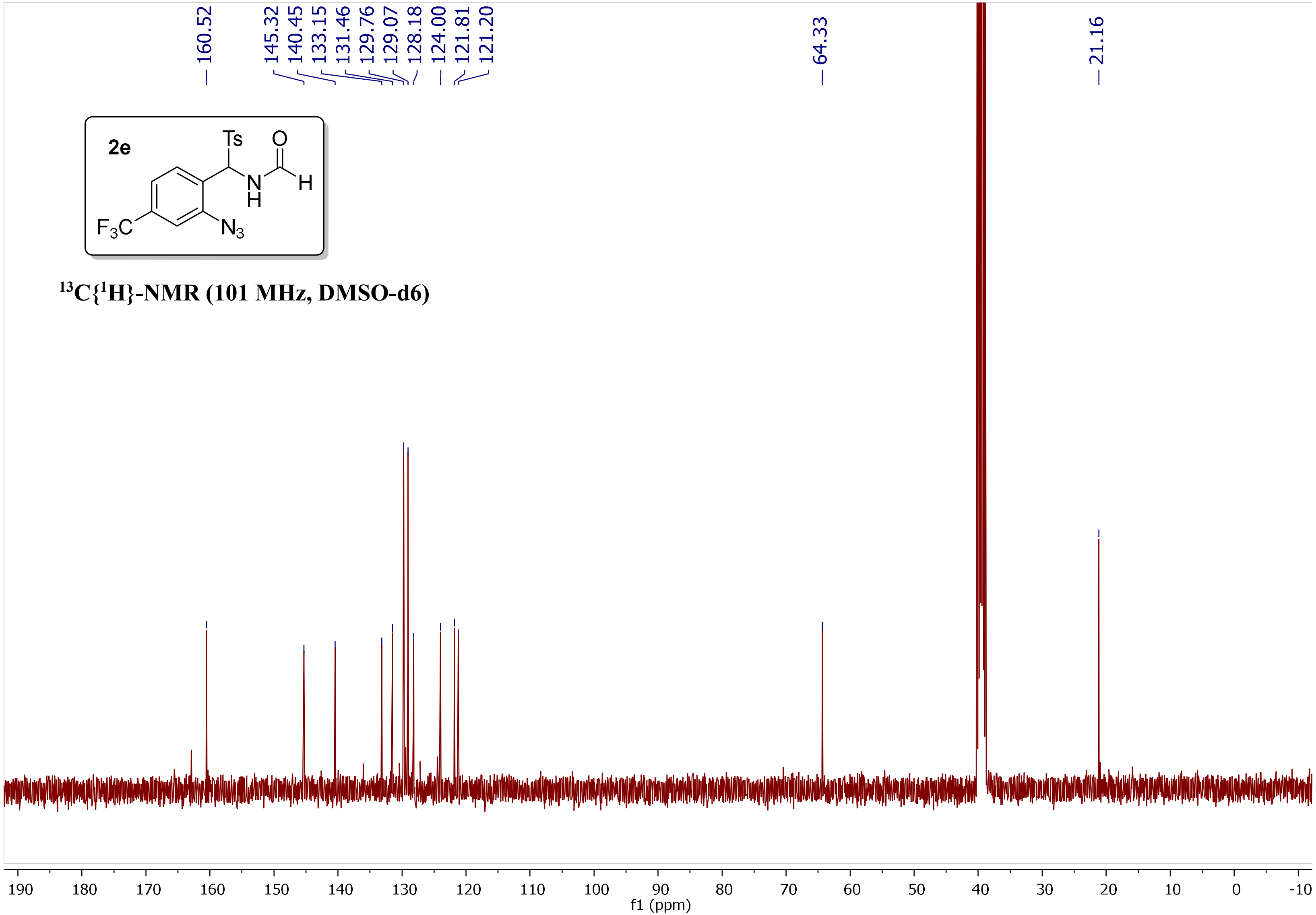
160.52
145.32
140.45
133.15
131.46
129.76
129.07
128.18
124.00
121.81
121.20
64.33
21.16
2e
Ts
O
N
H
H
F_3C
N_3
$^{13}C\{^1H\}$-NMR (101 MHz, DMSO-d6)
190
180
170
160
150
140
130
120
110
100
90
80
70
60
50
40
30
20
10
0
-10
f1 (ppm)

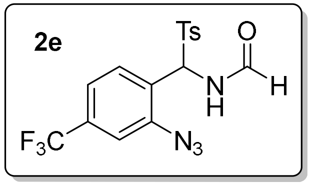

^{19}F-NMR (376 MHz, DMSO-d6)

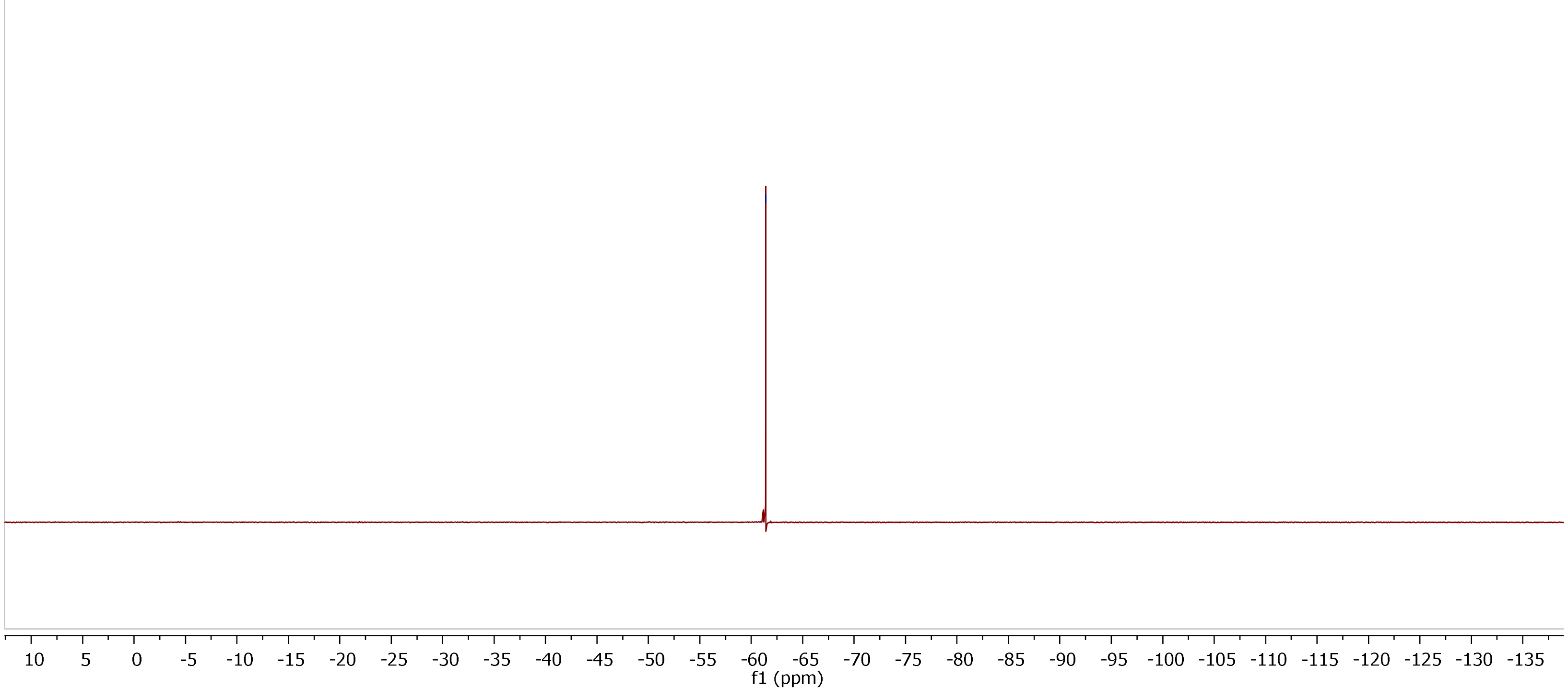

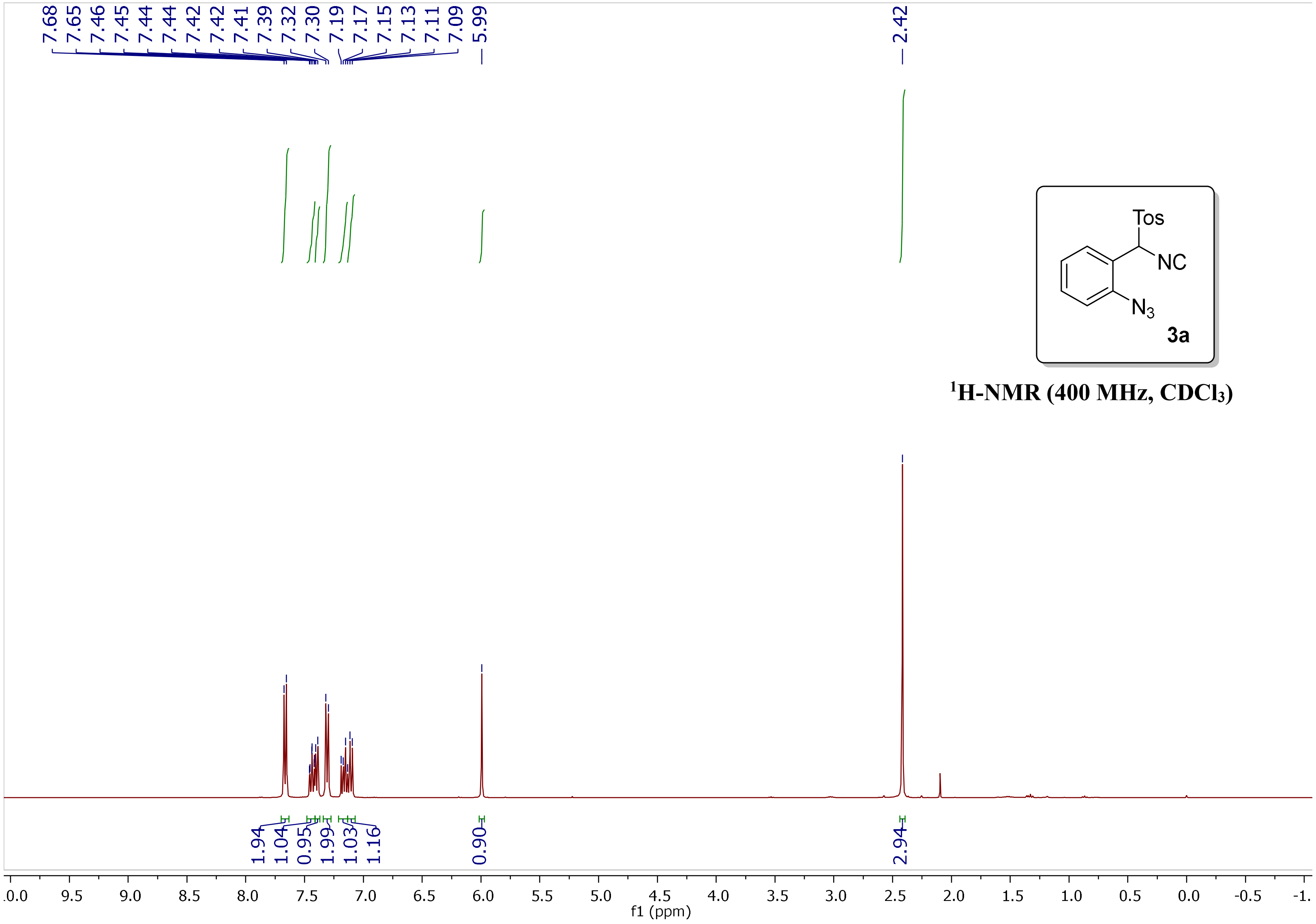

^{1}H-NMR (400 MHz, $CDCl_3$)

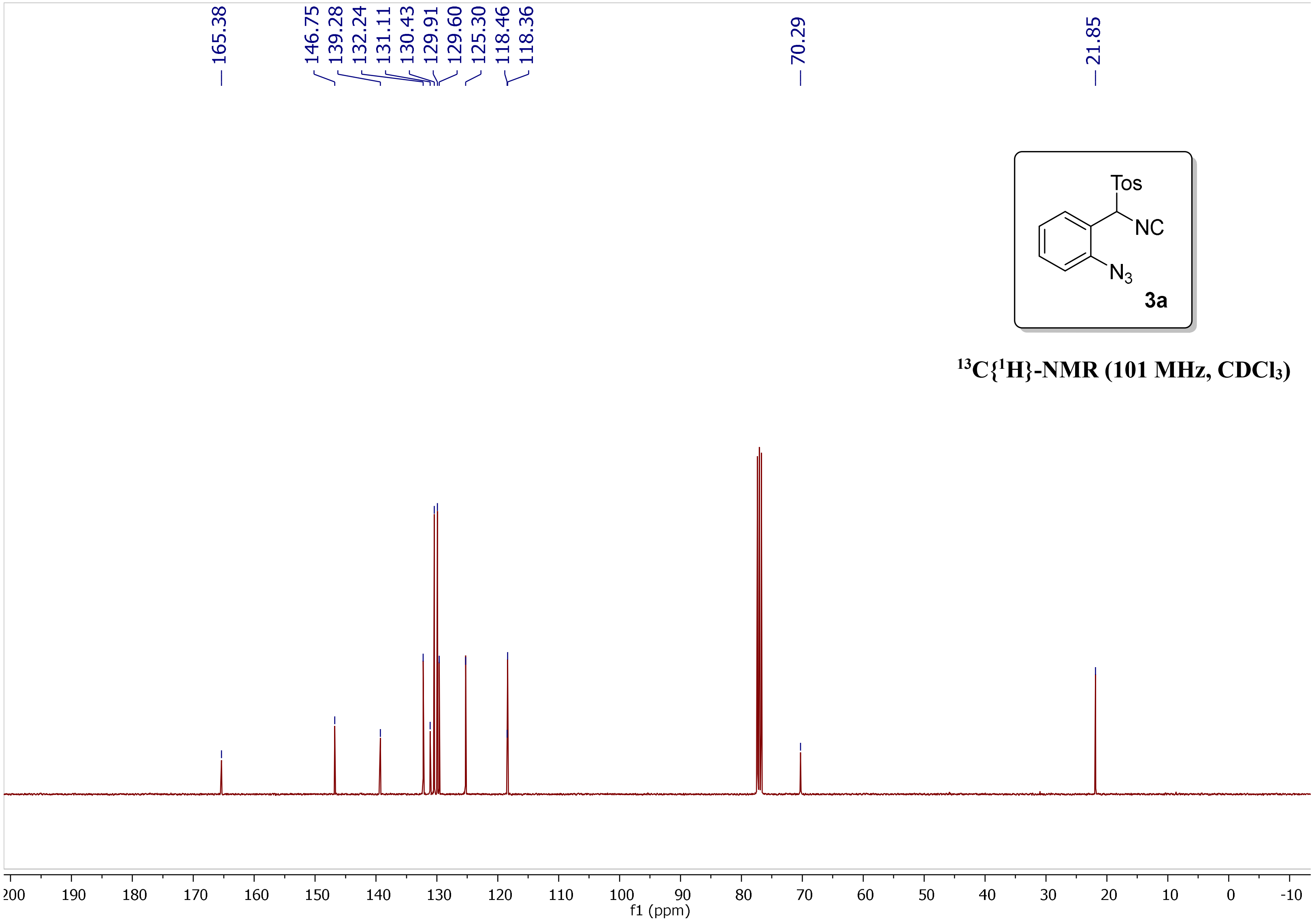
165.38
146.75
139.28
132.24
131.11
130.43
129.91
129.60
125.30
118.46
118.36
70.29
21.85
Tos
NC
N3
3a
13C{1H}-NMR (101 MHz, CDCl3)
200
190
180
170
160
150
140
130
120
110
100
90
80
70
60
50
40
30
20
10
0
-10
f1 (ppm)

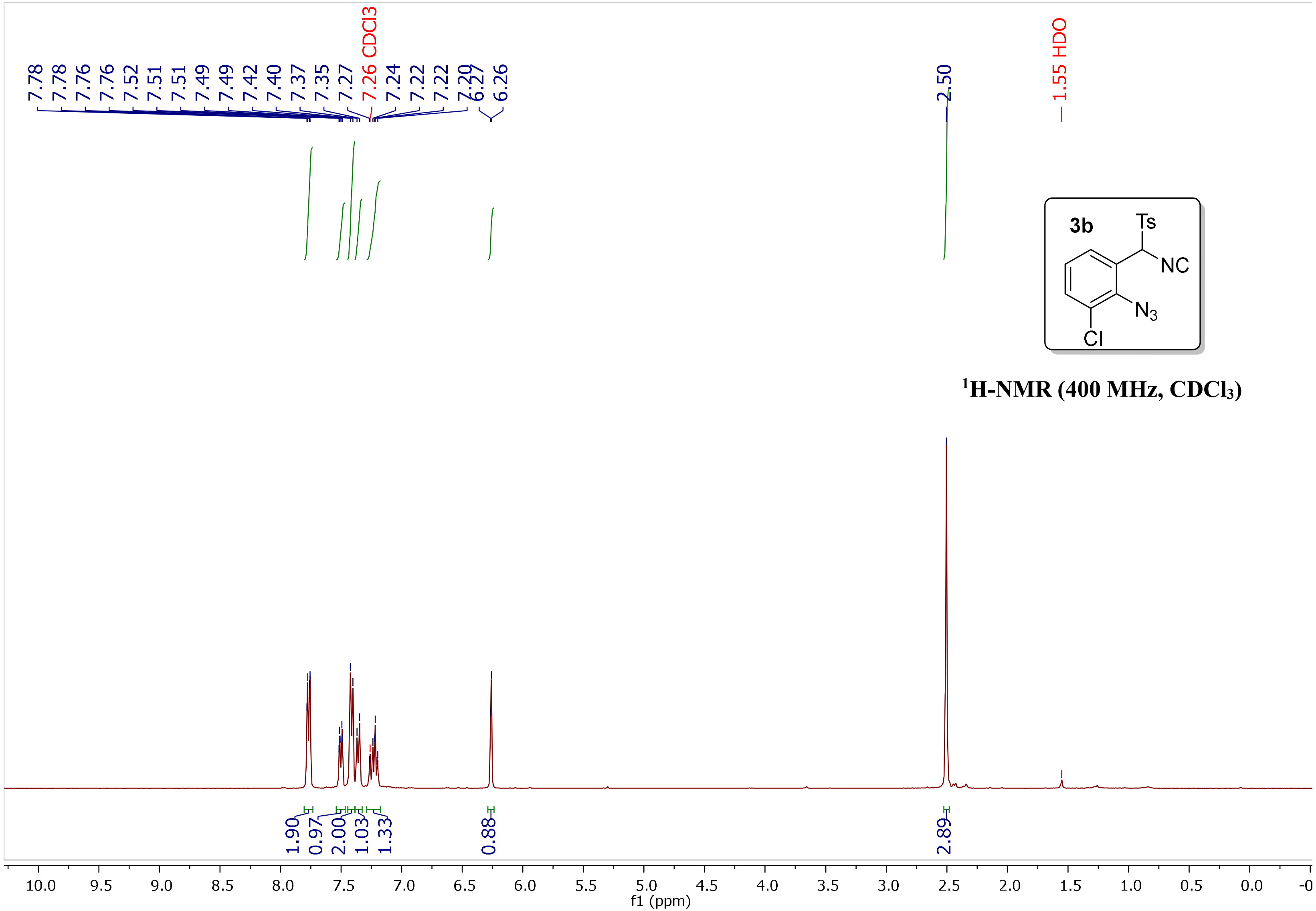

7.78
7.78
7.76
7.76
7.52
7.51
7.51
7.49
7.49
7.42
7.40
7.37
7.35
7.27
7.26 CDCl3
7.24
7.22
7.22
7.20
6.27
6.26
2.50
1.55 HDO
3b
Ts
NC
N3
Cl
1H-NMR (400 MHz, CDCl3)
1.90
0.97
2.00
1.03
1.33
0.88
2.89
10.0
9.5
9.0
8.5
8.0
7.5
7.0
6.5
6.0
5.5
5.0
4.5
4.0
3.5
3.0
2.5
2.0
1.5
1.0
0.5
0.0
-0
f1 (ppm)

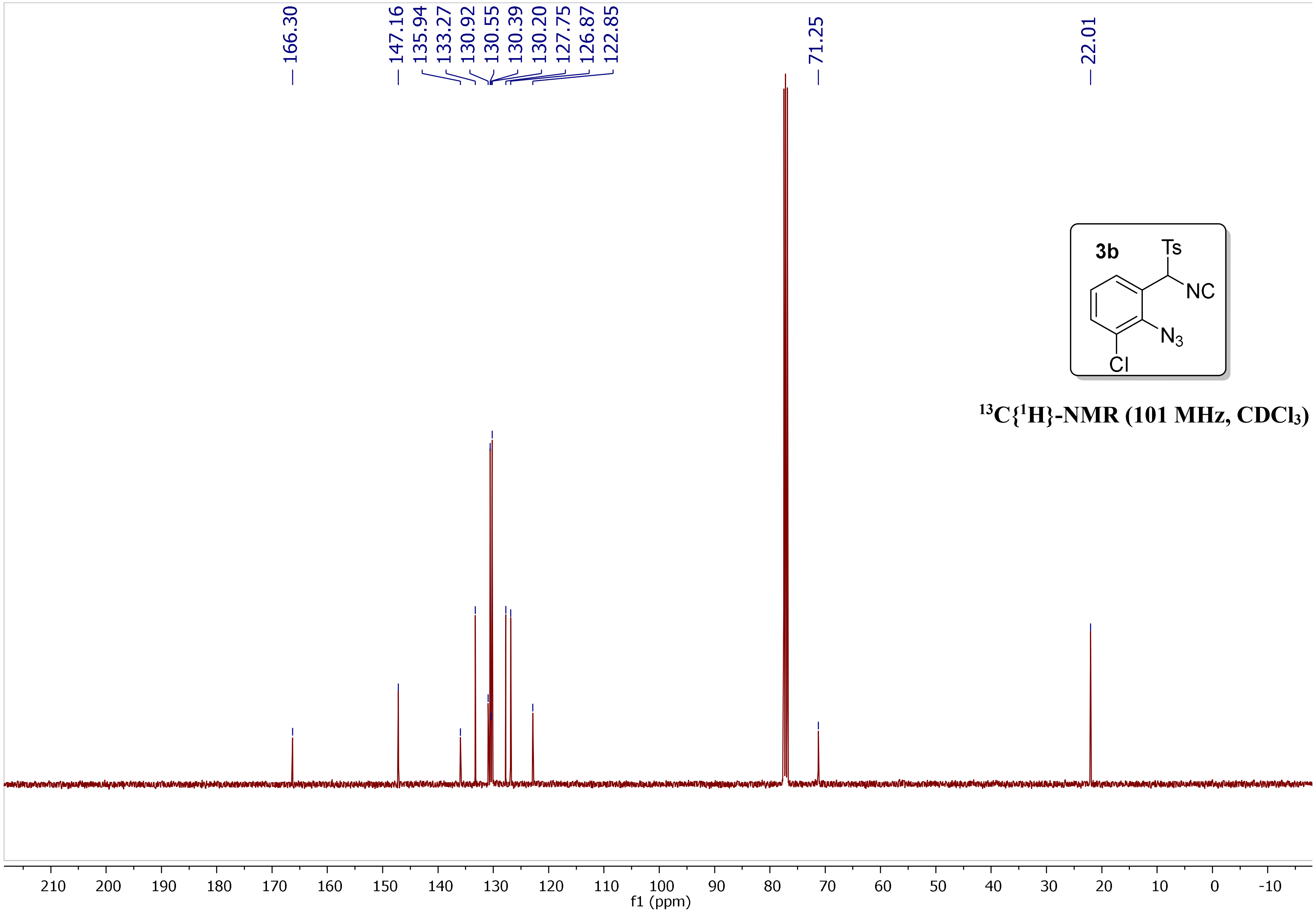
166.30
147.16
135.94
133.27
130.92
130.55
130.39
130.20
127.75
126.87
122.85
71.25
22.01
3b
Ts
NC
N3
Cl
13C{1H}-NMR (101 MHz, CDCl3)
210 200 190 180 170 160 150 140 130 120 110 100 90 80 70 60 50 40 30 20 10 0 -10
f1 (ppm)

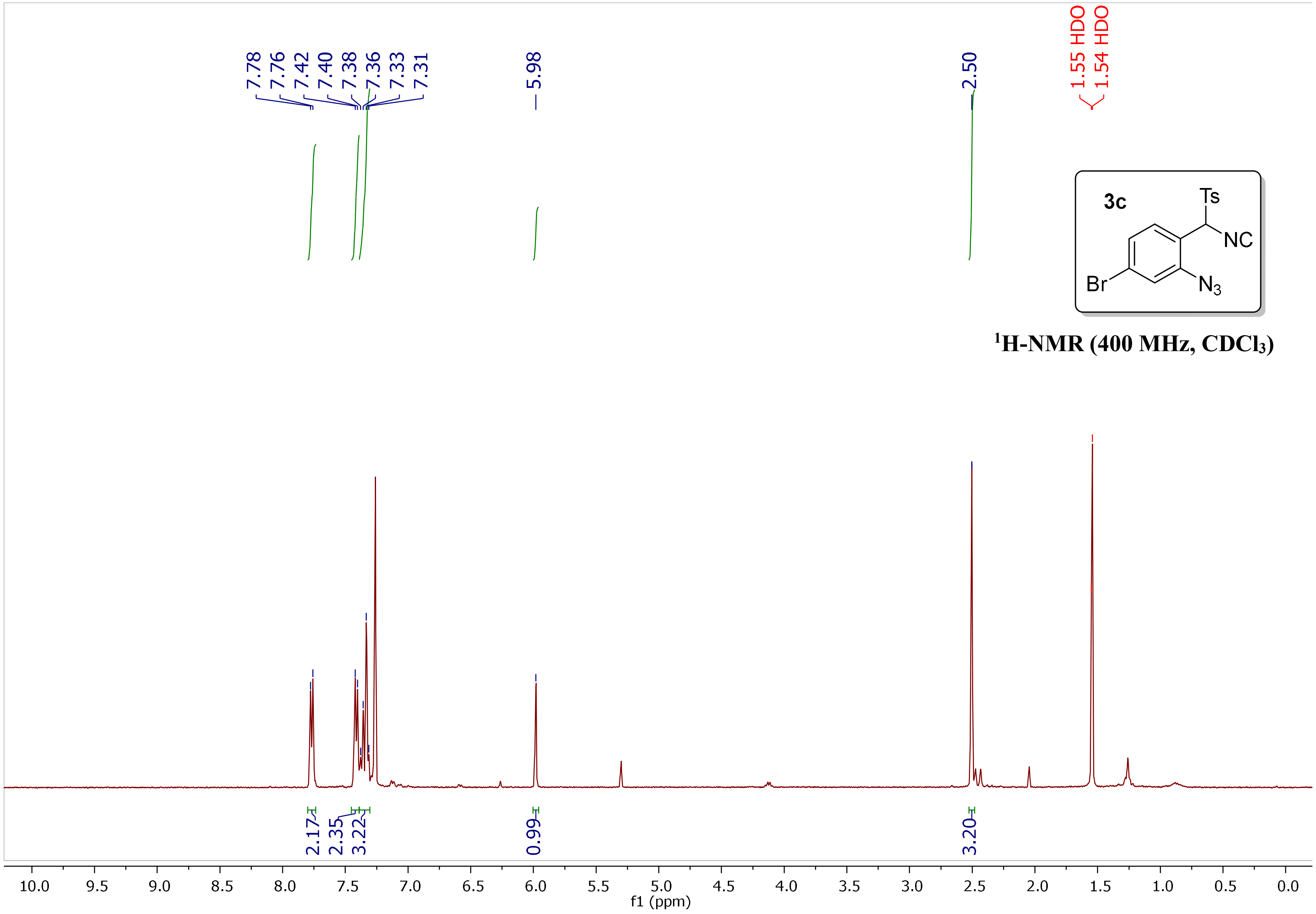

7.78
7.76
7.42
7.40
7.38
7.36
7.33
7.31
5.98
2.50
1.55 HDO
1.54 HDO
3c
Ts
NC
Br
N3
1H-NMR (400 MHz, CDCl3)
2.17
2.35
3.22
0.99
3.20
f1 (ppm)

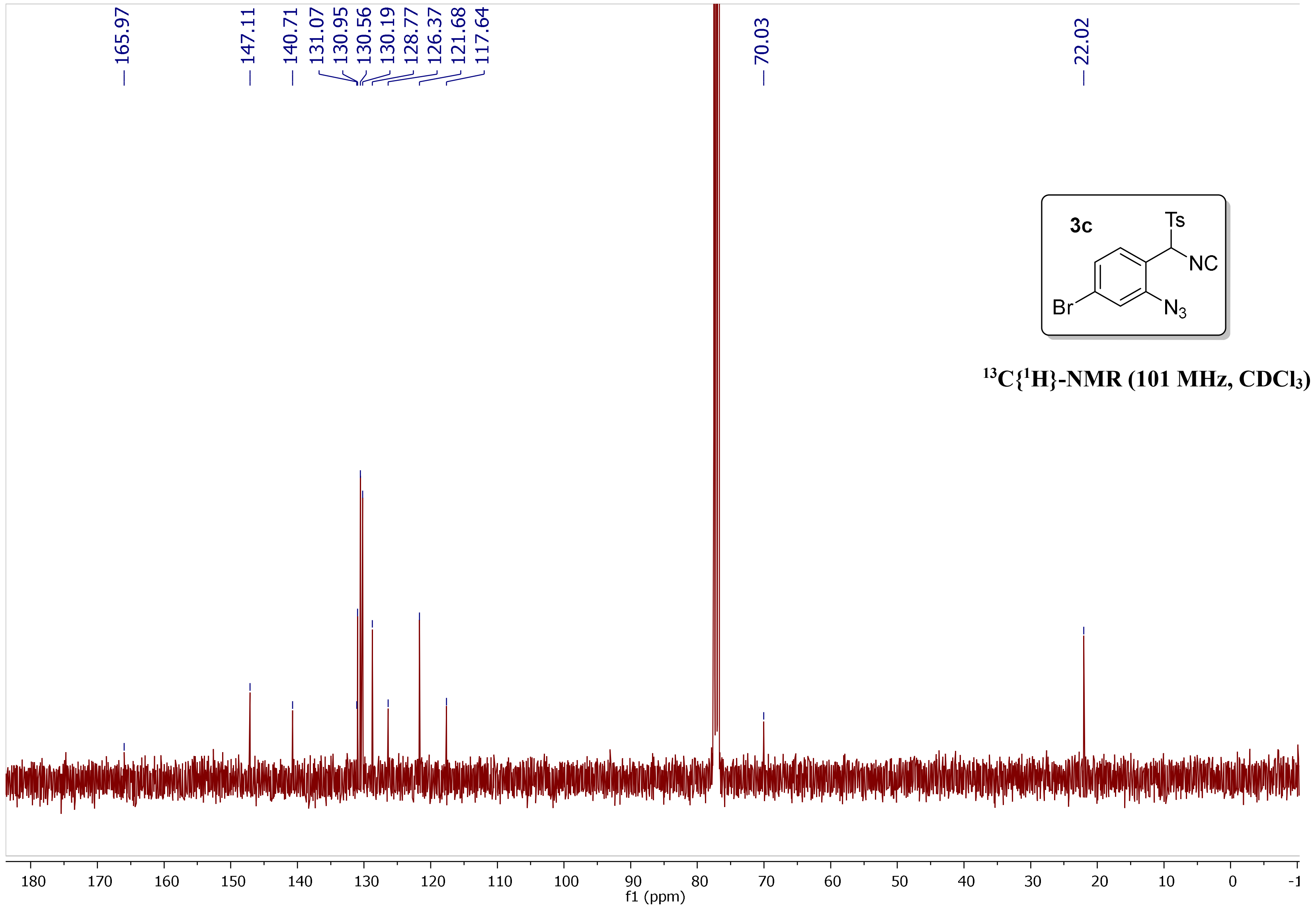
165.97
147.11
140.71
131.07
130.95
130.56
130.19
128.77
126.37
121.68
117.64
70.03
22.02
3c
Ts
NC
Br
N3
13C{1H}-NMR (101 MHz, CDCl3)
180
170
160
150
140
130
120
110
100
90
80
70
60
50
40
30
20
10
0
f1 (ppm)

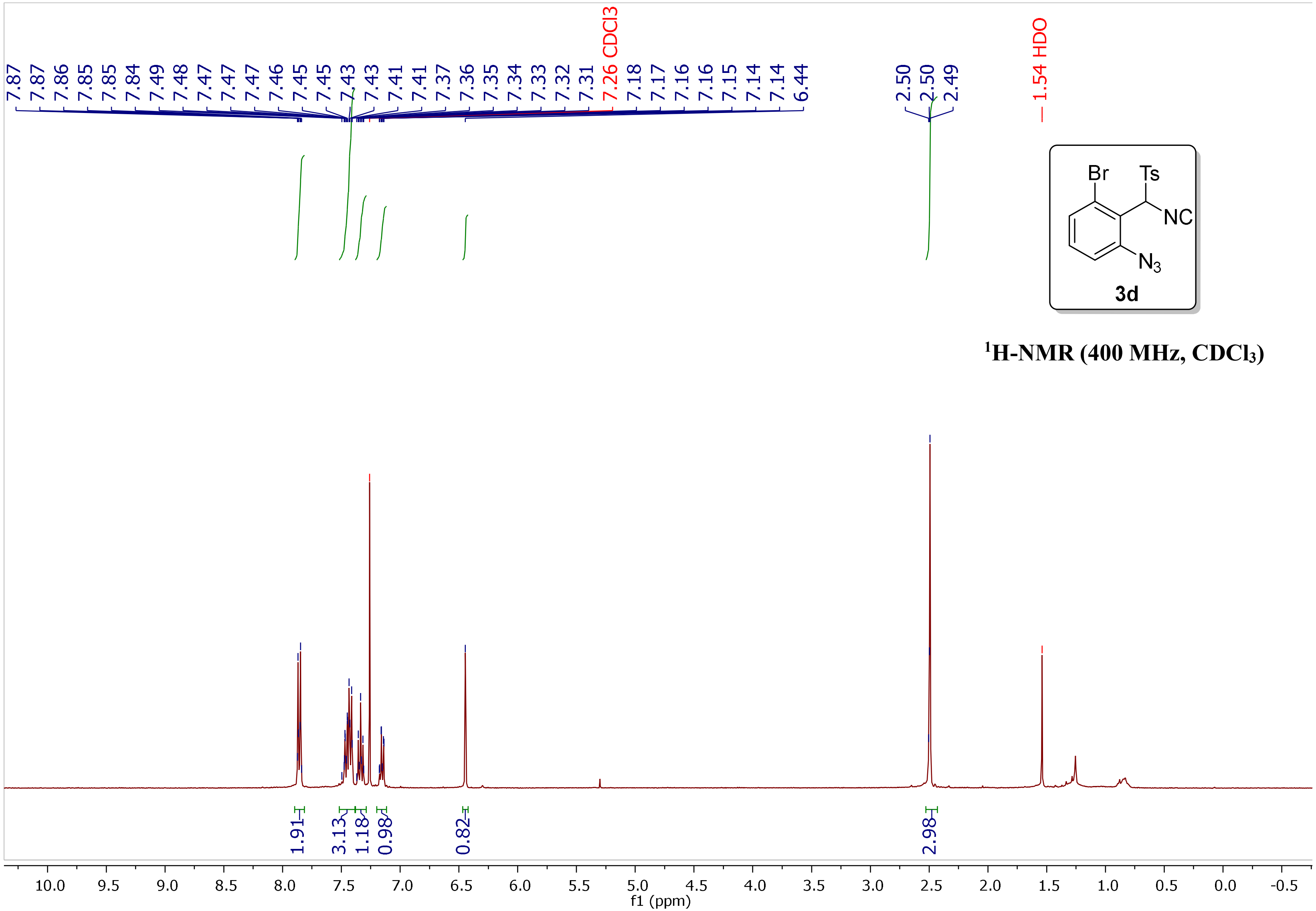

^{1}H-NMR (400 MHz, $CDCl_3$)

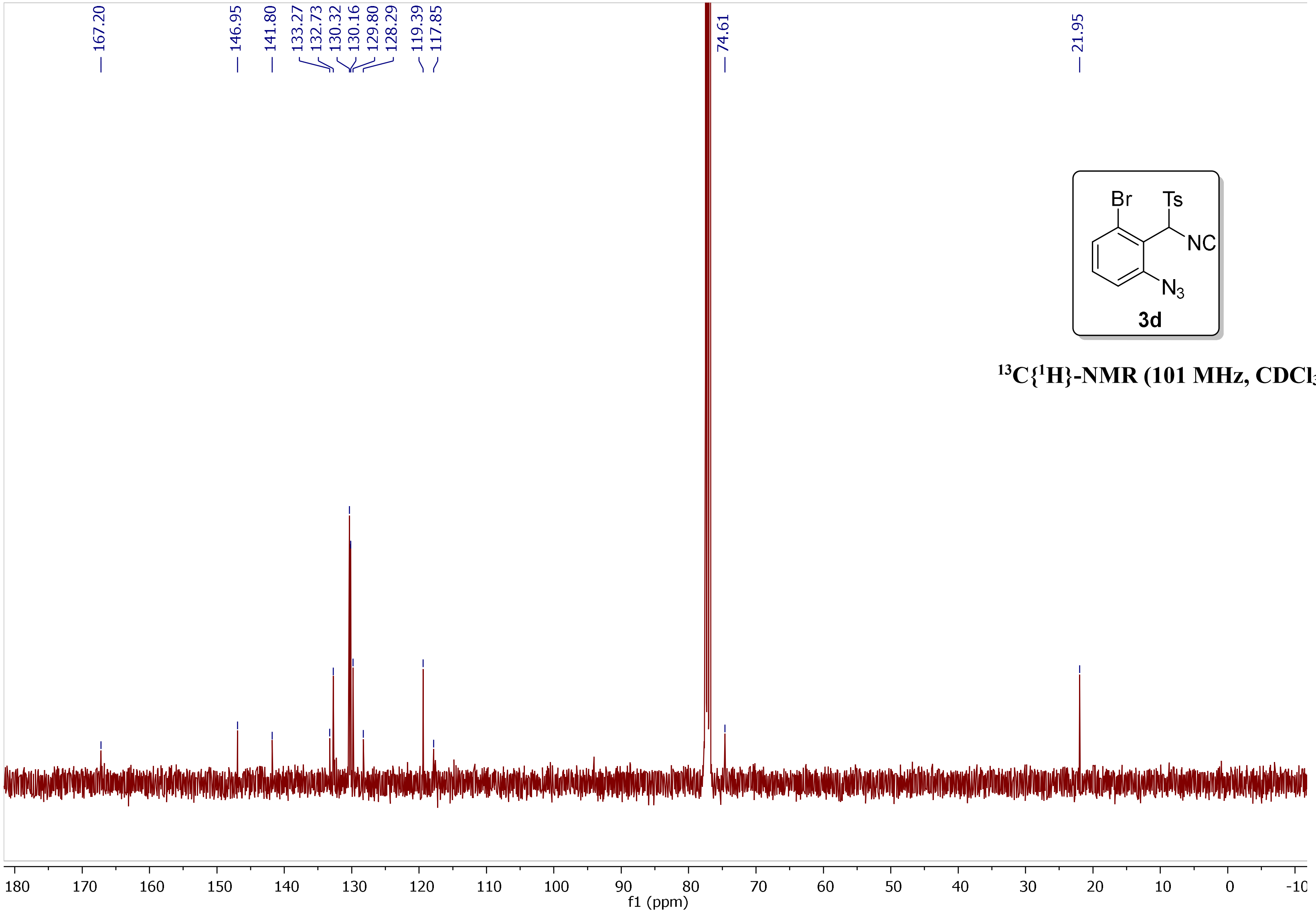

3d

$^{13}C\{^1H\}$-NMR (101 MHz, $CDCl_3$)

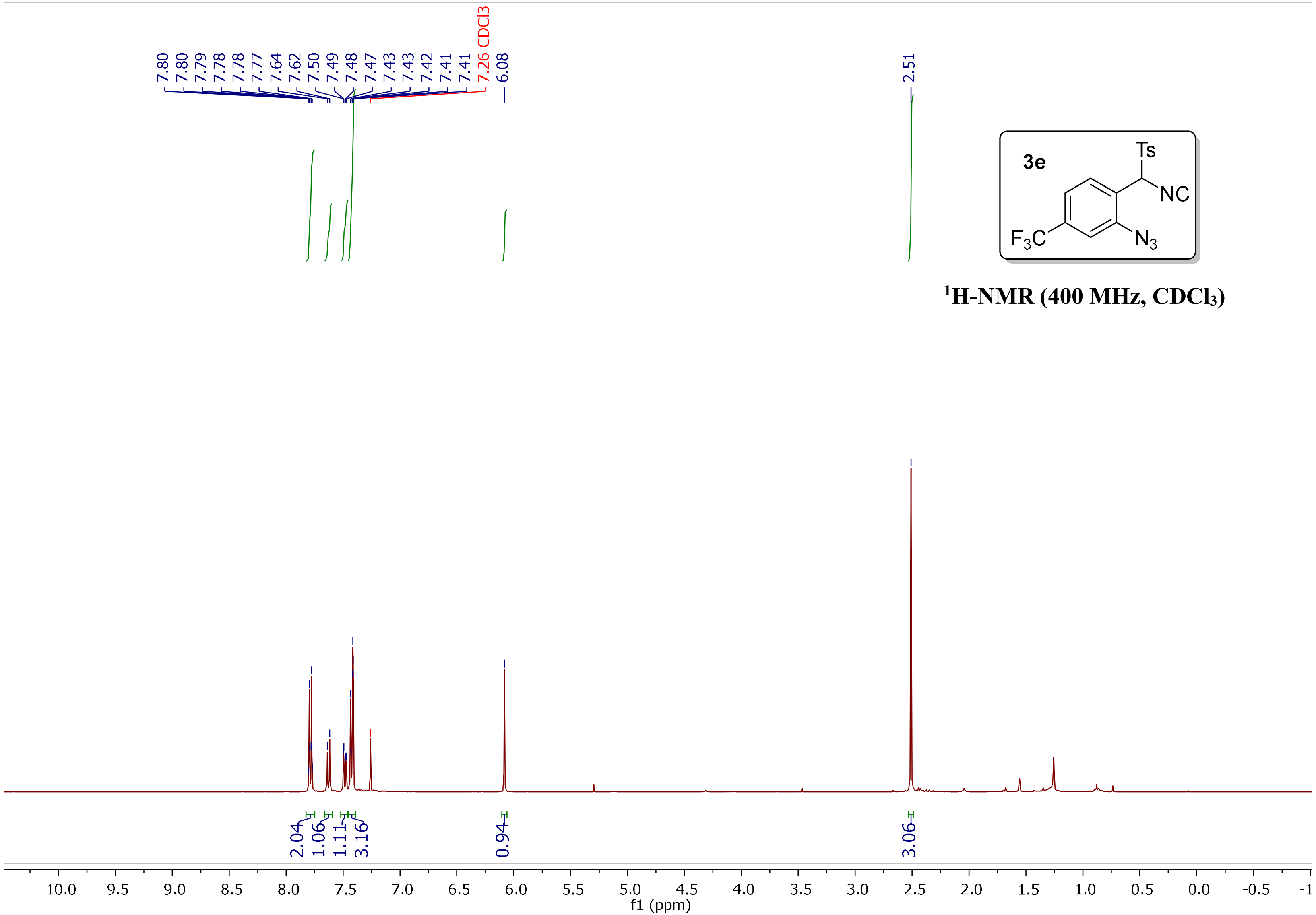

^{1}H-NMR (400 MHz, $CDCl_3$)

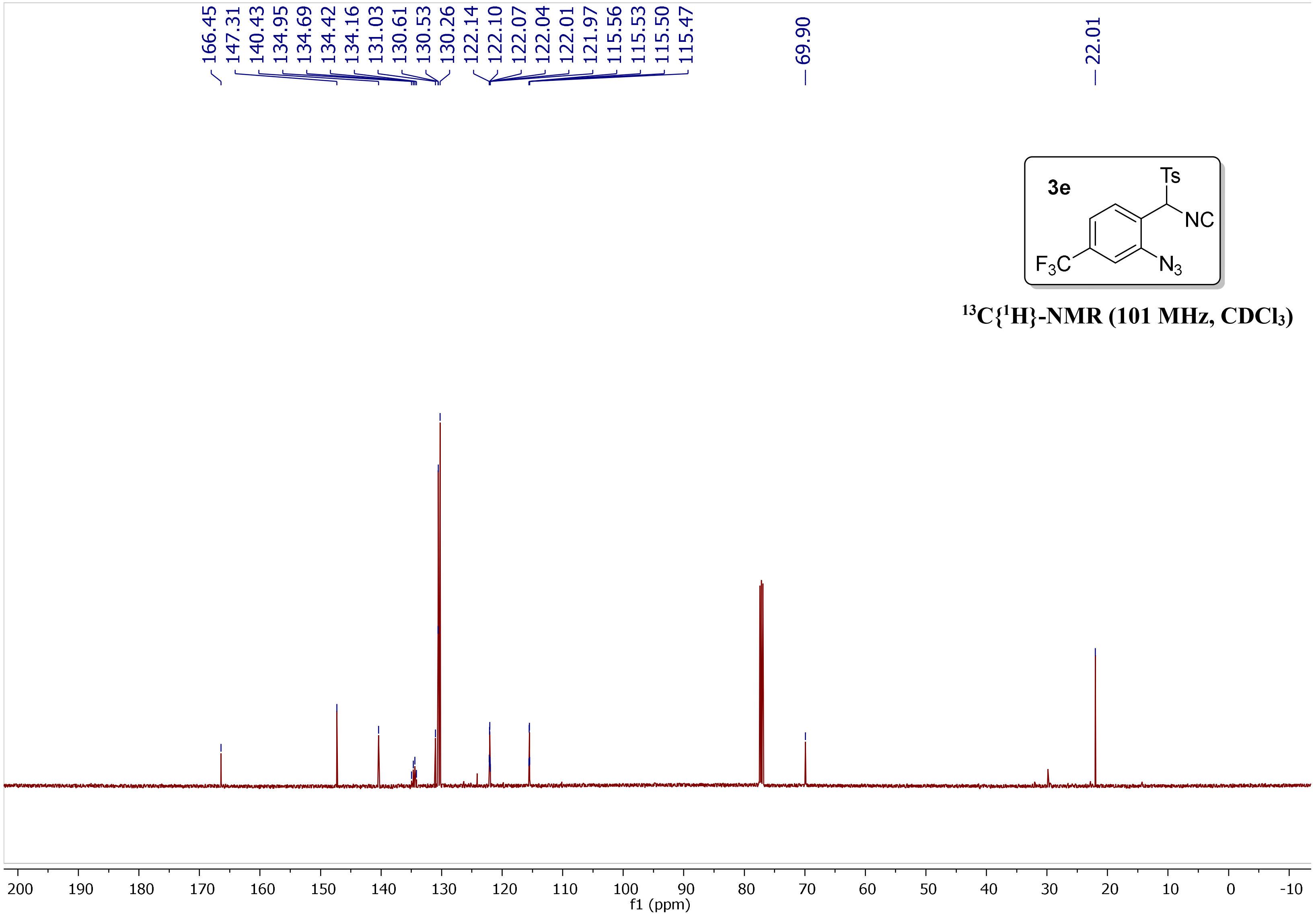
166.45
147.31
140.43
134.95
134.69
134.42
134.16
131.03
130.61
130.53
130.26
122.14
122.10
122.07
122.04
122.01
121.97
115.56
115.53
115.50
115.47
69.90
22.01
3e
Ts
NC
F_3C
N_3
$^{13}C\{^1H\}$-NMR (101 MHz, $CDCl_3$)
200
190
180
170
160
150
140
130
120
110
100
90
80
70
60
50
40
30
20
10
0
-10
f1 (ppm)

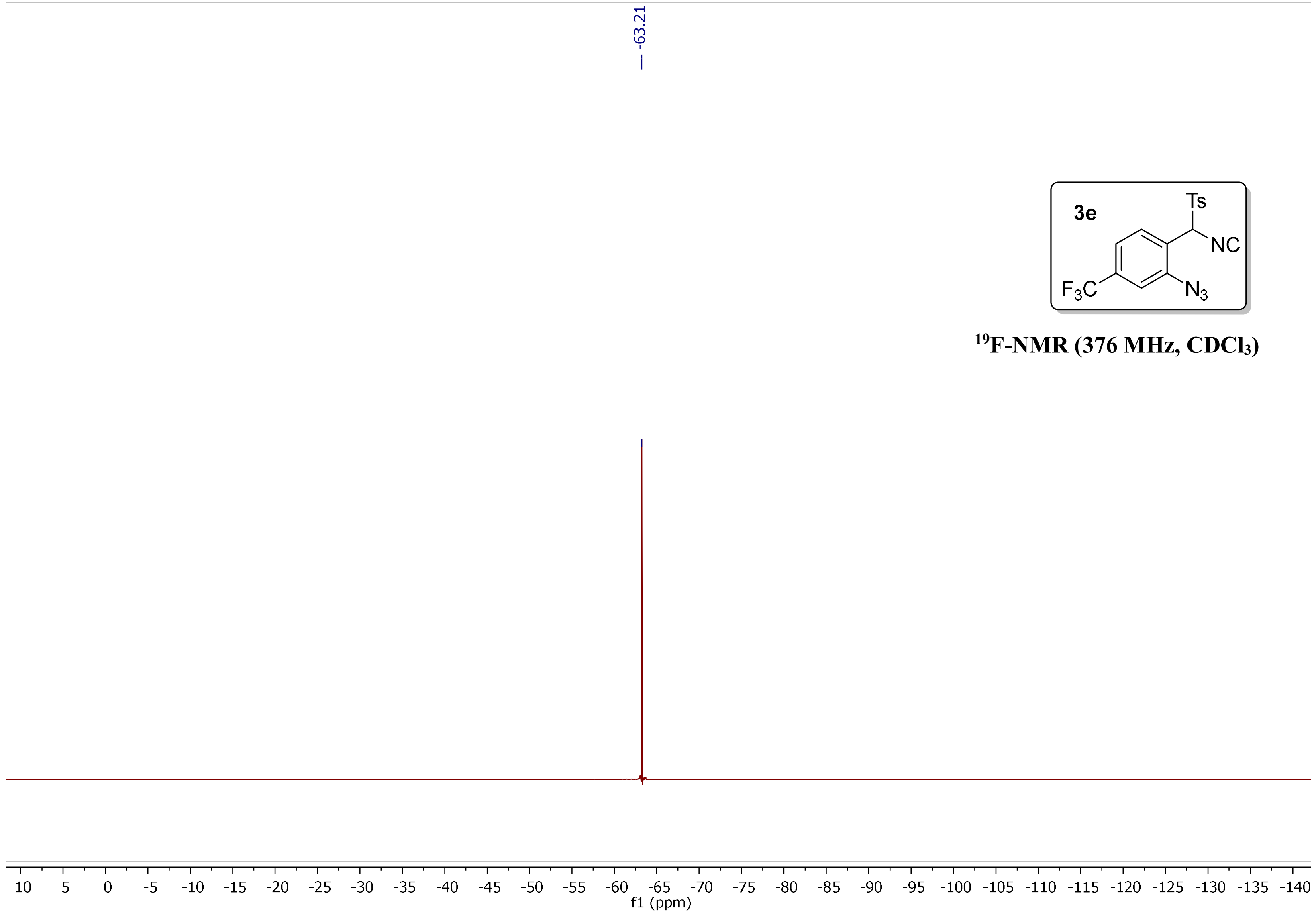

-63.21
3e
Ts
NC
F3C
N3
19F-NMR (376 MHz, CDCl3)
10 5 0 -5 -10 -15 -20 -25 -30 -35 -40 -45 -50 -55 -60 -65 -70 -75 -80 -85 -90 -95 -100 -105 -110 -115 -120 -125 -130 -135 -140
f1 (ppm)

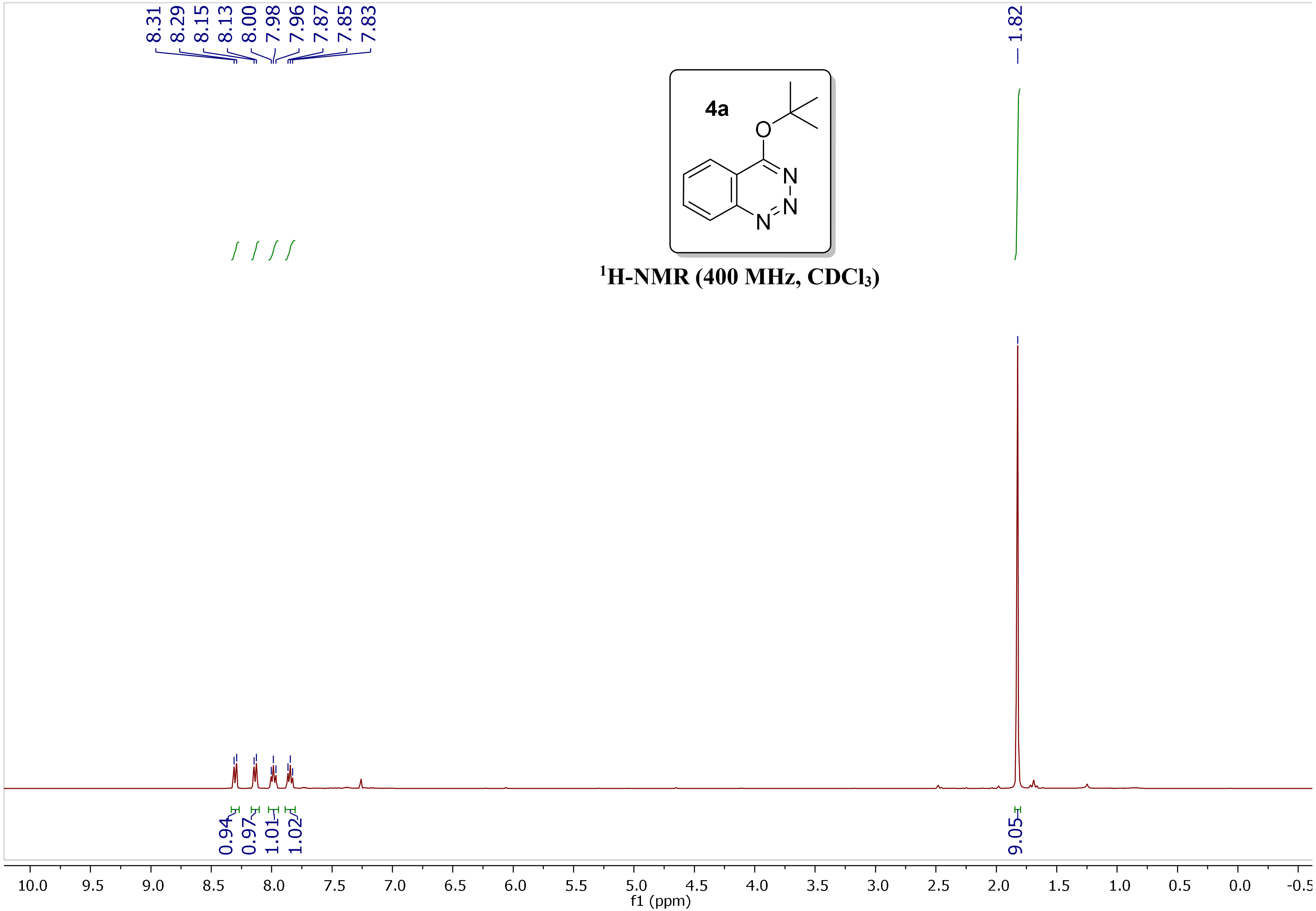

8.31
8.29
8.15
8.13
8.00
7.98
7.96
7.87
7.85
7.83
1.82
4a
O
N
N
N
1H-NMR (400 MHz, CDCl3)
0.94
0.97
1.01
1.02
9.05
10.0
9.5
9.0
8.5
8.0
7.5
7.0
6.5
6.0
5.5
5.0
4.5
4.0
3.5
3.0
2.5
2.0
1.5
1.0
0.5
0.0
-0.5
f1 (ppm)

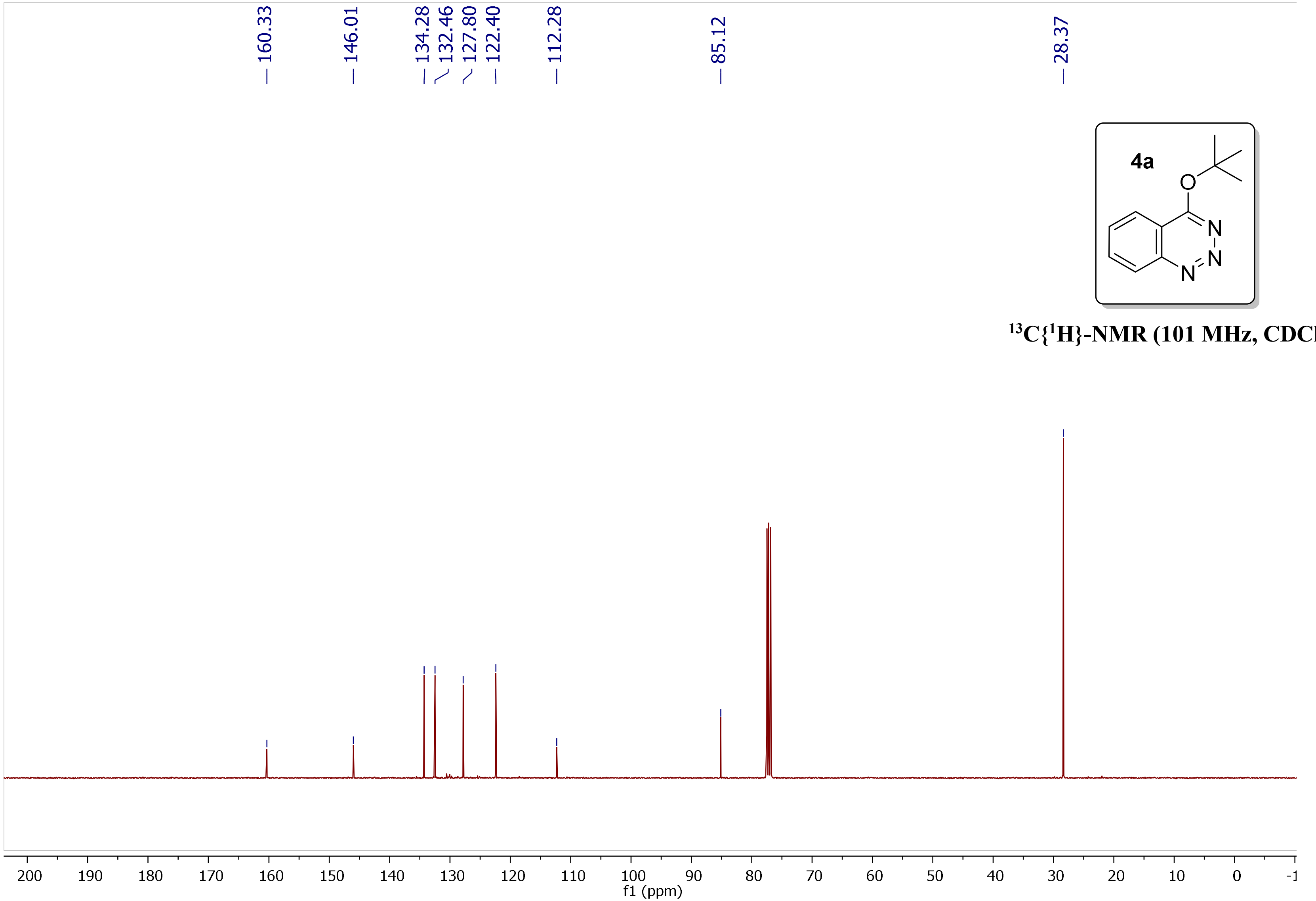

$^{13}C\{^{1}H\}$-NMR (101 MHz, $CDCl_3$)

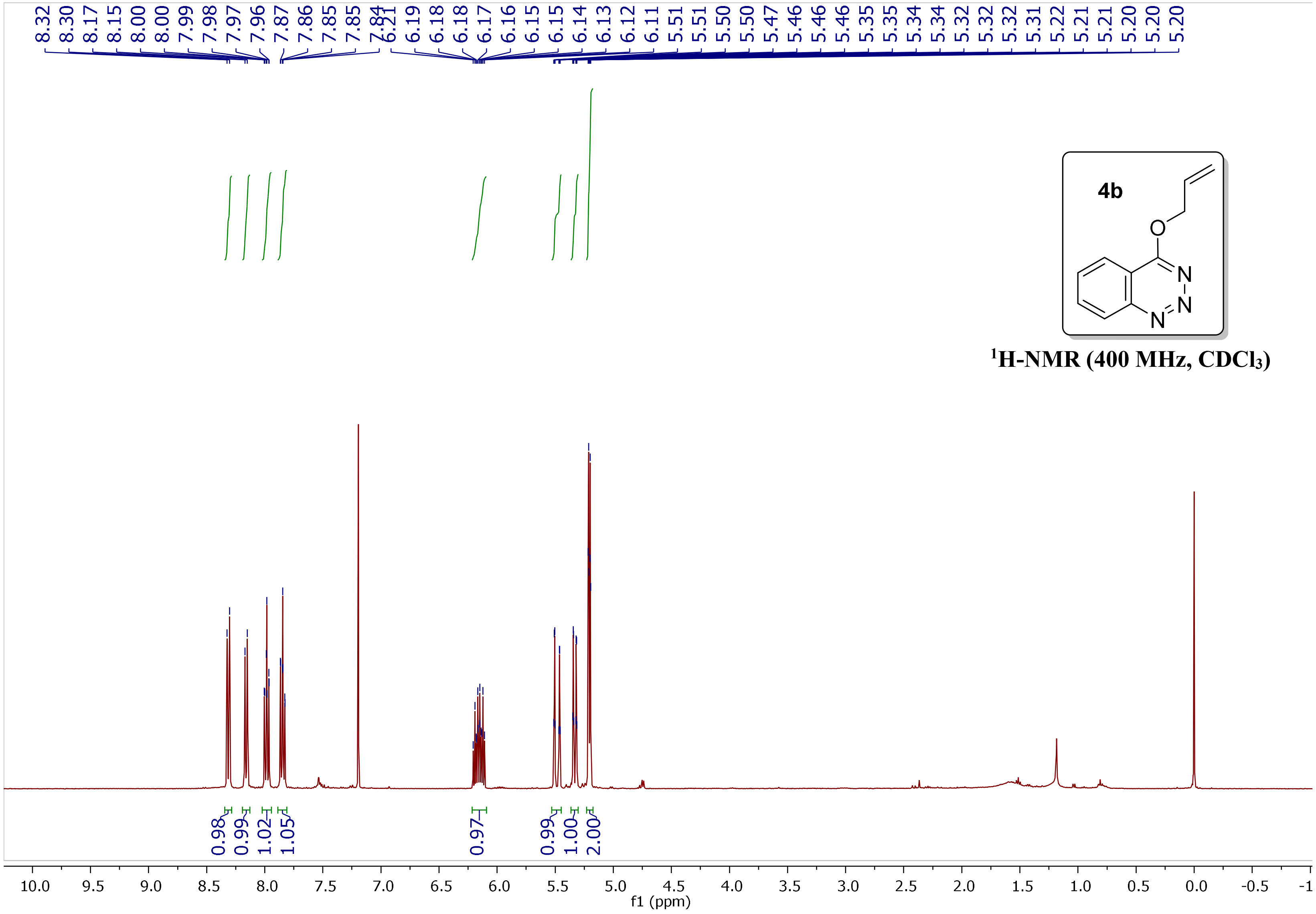
8.32
8.30
8.17
8.15
8.00
8.00
7.99
7.98
7.97
7.96
7.87
7.86
7.85
7.85
7.84
6.21
6.19
6.18
6.18
6.17
6.16
6.15
6.15
6.14
6.13
6.12
6.11
5.51
5.51
5.50
5.50
5.47
5.46
5.46
5.35
5.35
5.34
5.34
5.32
5.32
5.32
5.31
5.22
5.21
5.21
5.20
5.20
5.20
4b
O
N
N
N
^{1}H-NMR (400 MHz, $CDCl_3$)
0.98
0.99
1.02
1.05
0.97
0.99
1.00
2.00
10.0
9.5
9.0
8.5
8.0
7.5
7.0
6.5
6.0
5.5
5.0
4.5
4.0
3.5
3.0
2.5
2.0
1.5
1.0
0.5
0.0
-0.5
-1
f1 (ppm)

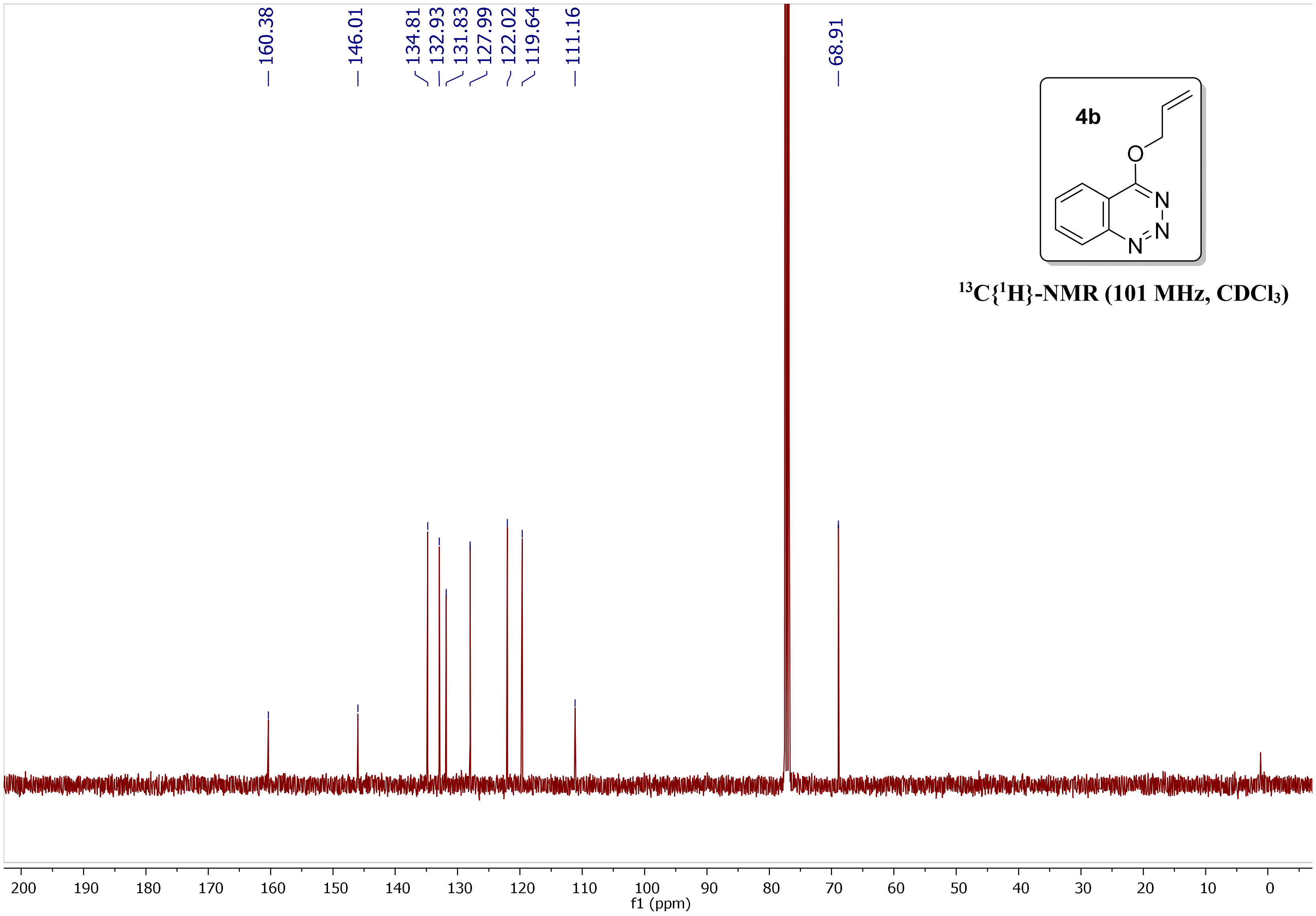
160.38
146.01
134.81
132.93
131.83
127.99
122.02
119.64
111.16
68.91
4b
O
N
N
N
$^{13}C\{^{1}H\}$-NMR (101 MHz, $CDCl_3$)
200 190 180 170 160 150 140 130 120 110 100 90 80 70 60 50 40 30 20 10 0
f1 (ppm)

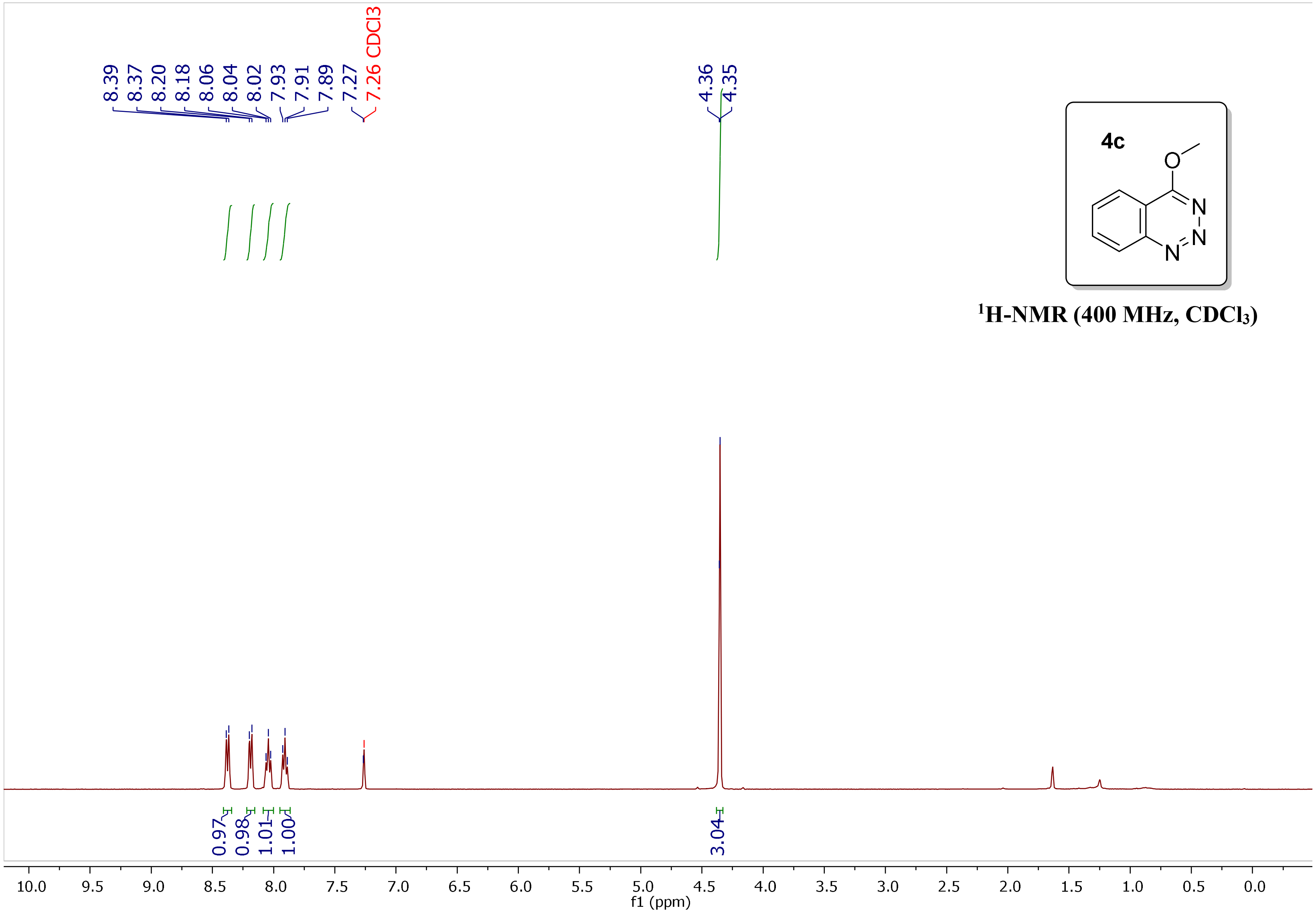

^{1}H-NMR (400 MHz, $CDCl_3$)

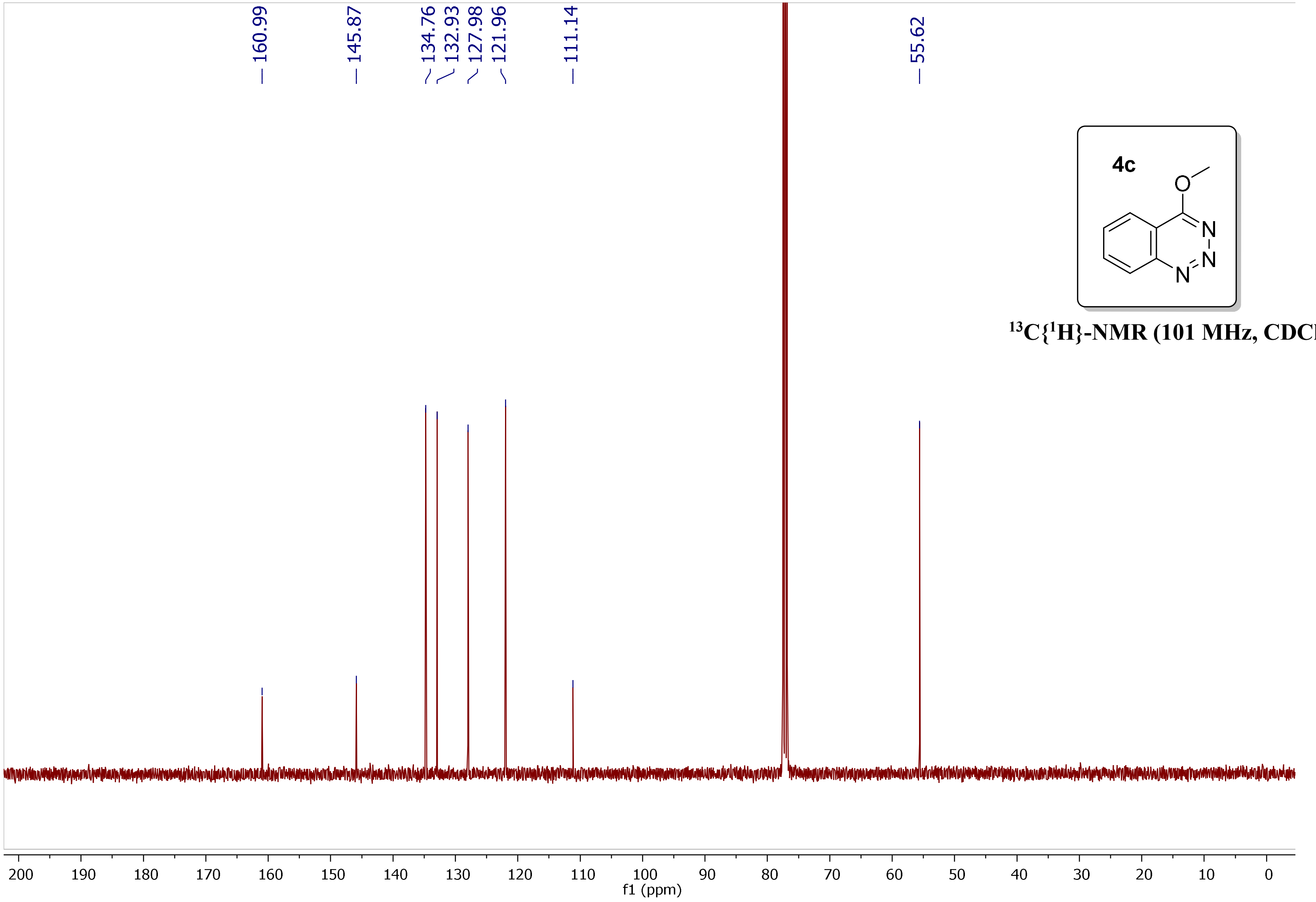

4c

$^{13}C\{^{1}H\}$-NMR (101 MHz, $CDCl_3$)

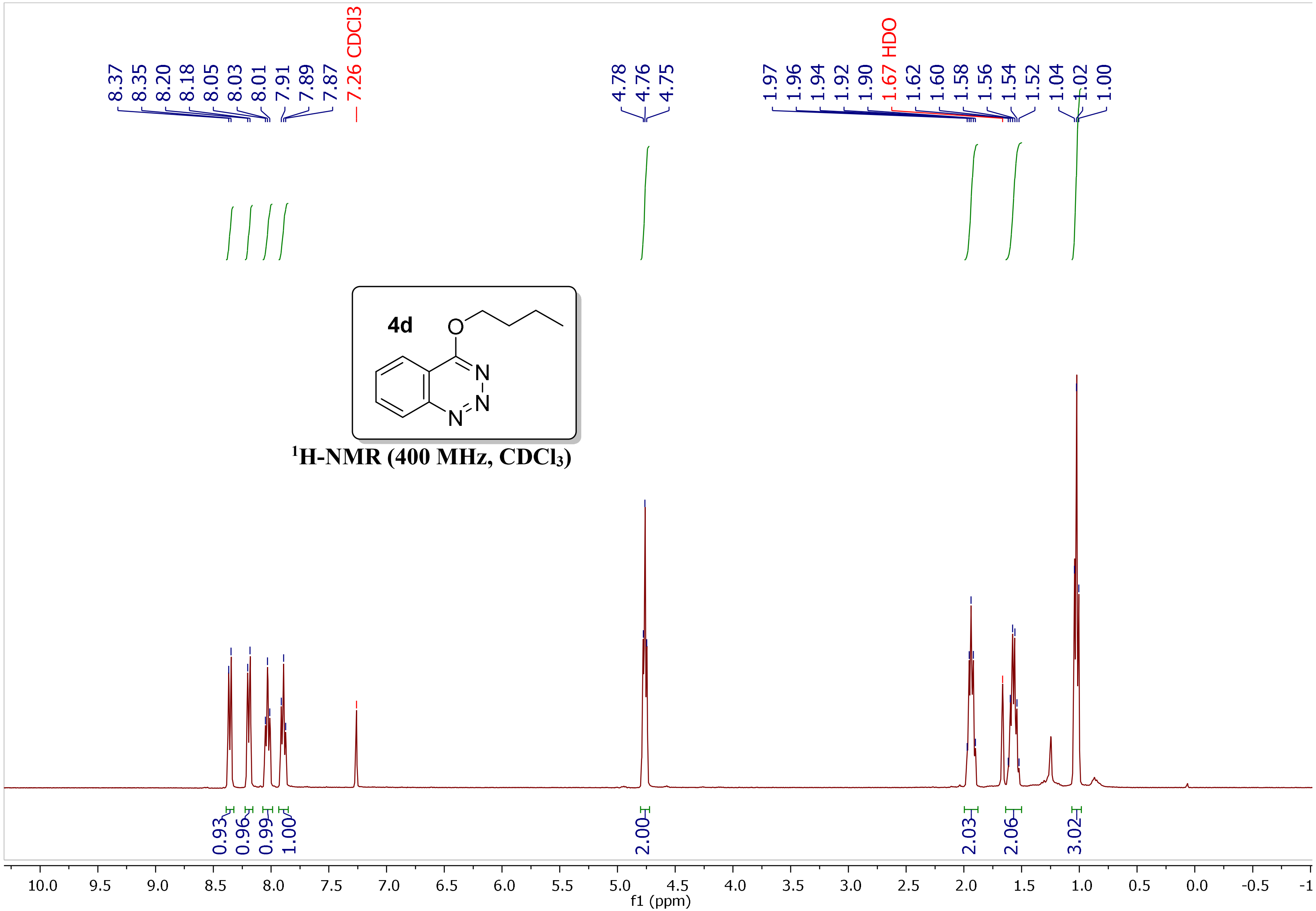

^{1}H-NMR (400 MHz, $CDCl_3$)

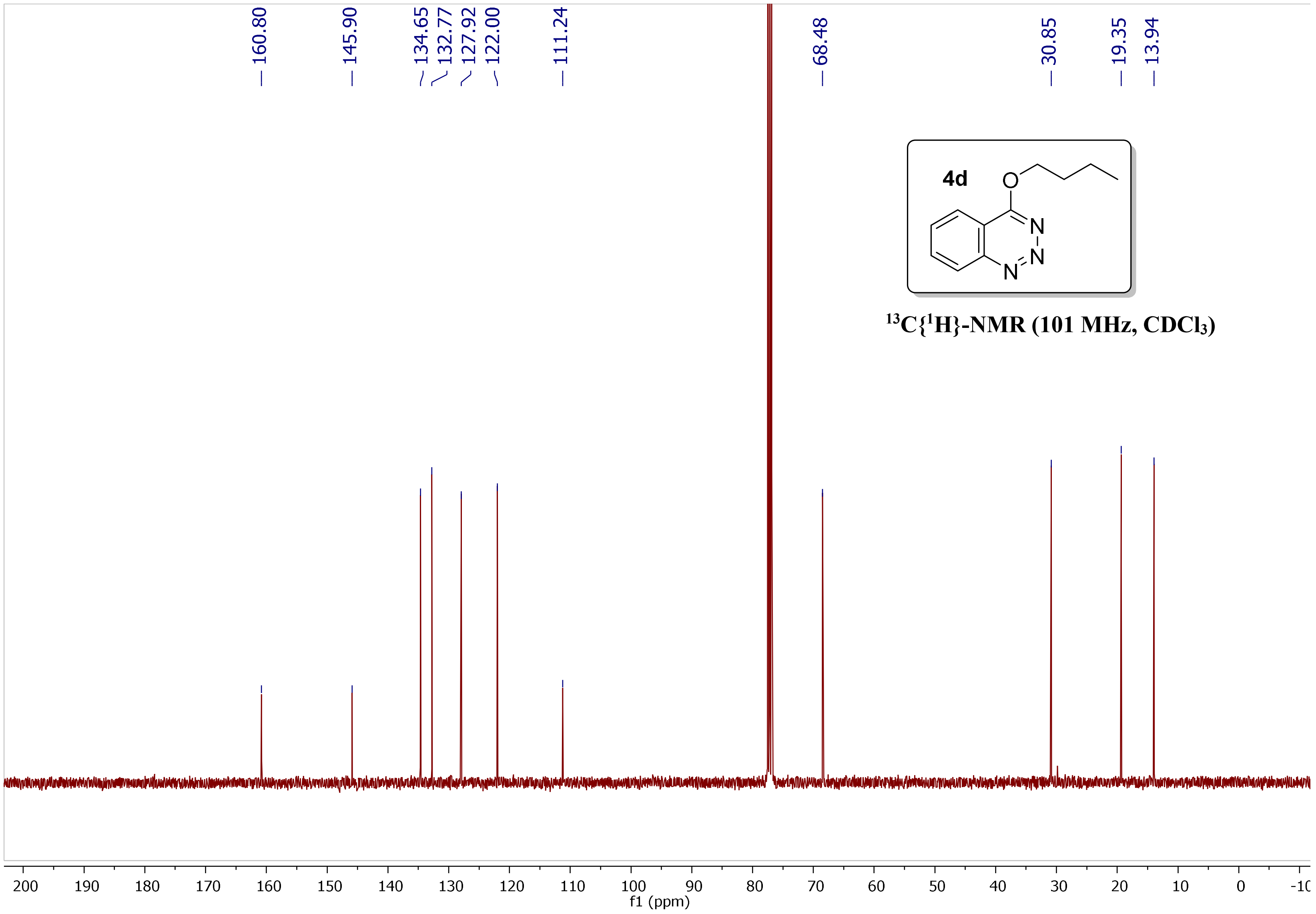
160.80
145.90
134.65
132.77
127.92
122.00
111.24
68.48
30.85
19.35
13.94
4d
O
N
N
N
$^{13}C\{^{1}H\}$-NMR (101 MHz, $CDCl_3$)
200 190 180 170 160 150 140 130 120 110 100 90 80 70 60 50 40 30 20 10 0 -10
f1 (ppm)

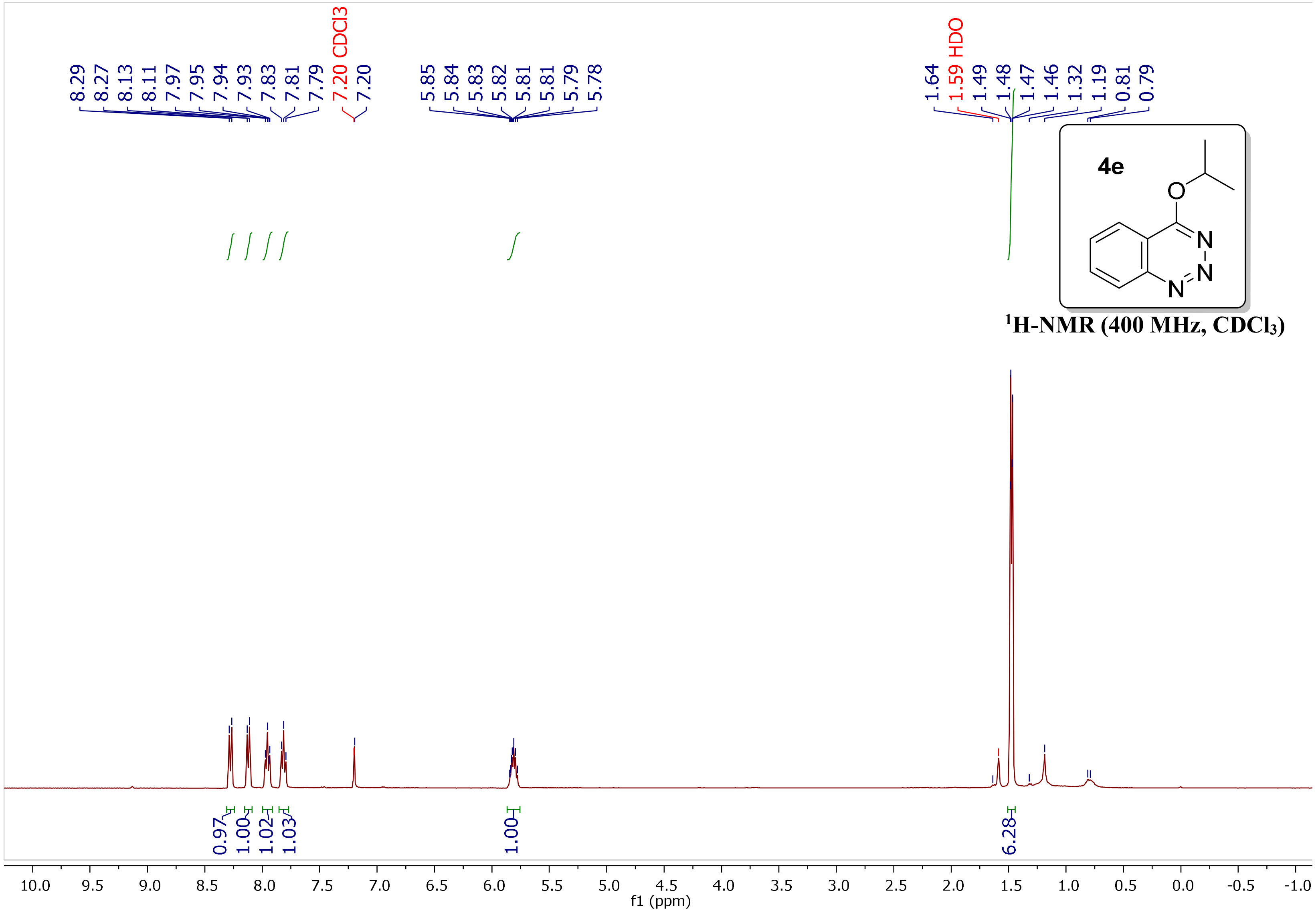

^{1}H-NMR (400 MHz, $CDCl_3$)

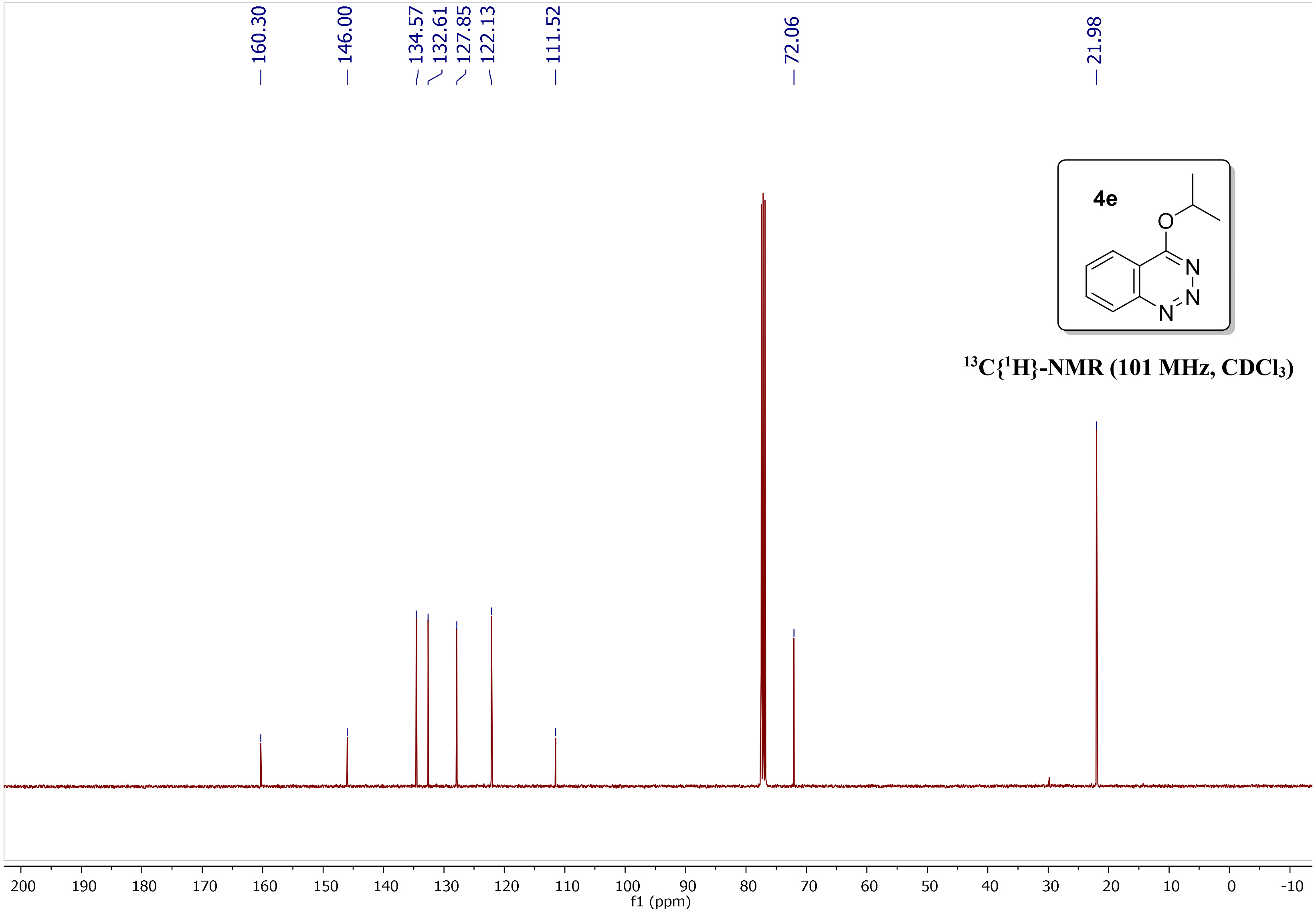

$^{13}C\{^{1}H\}$-NMR (101 MHz, $CDCl_3$)

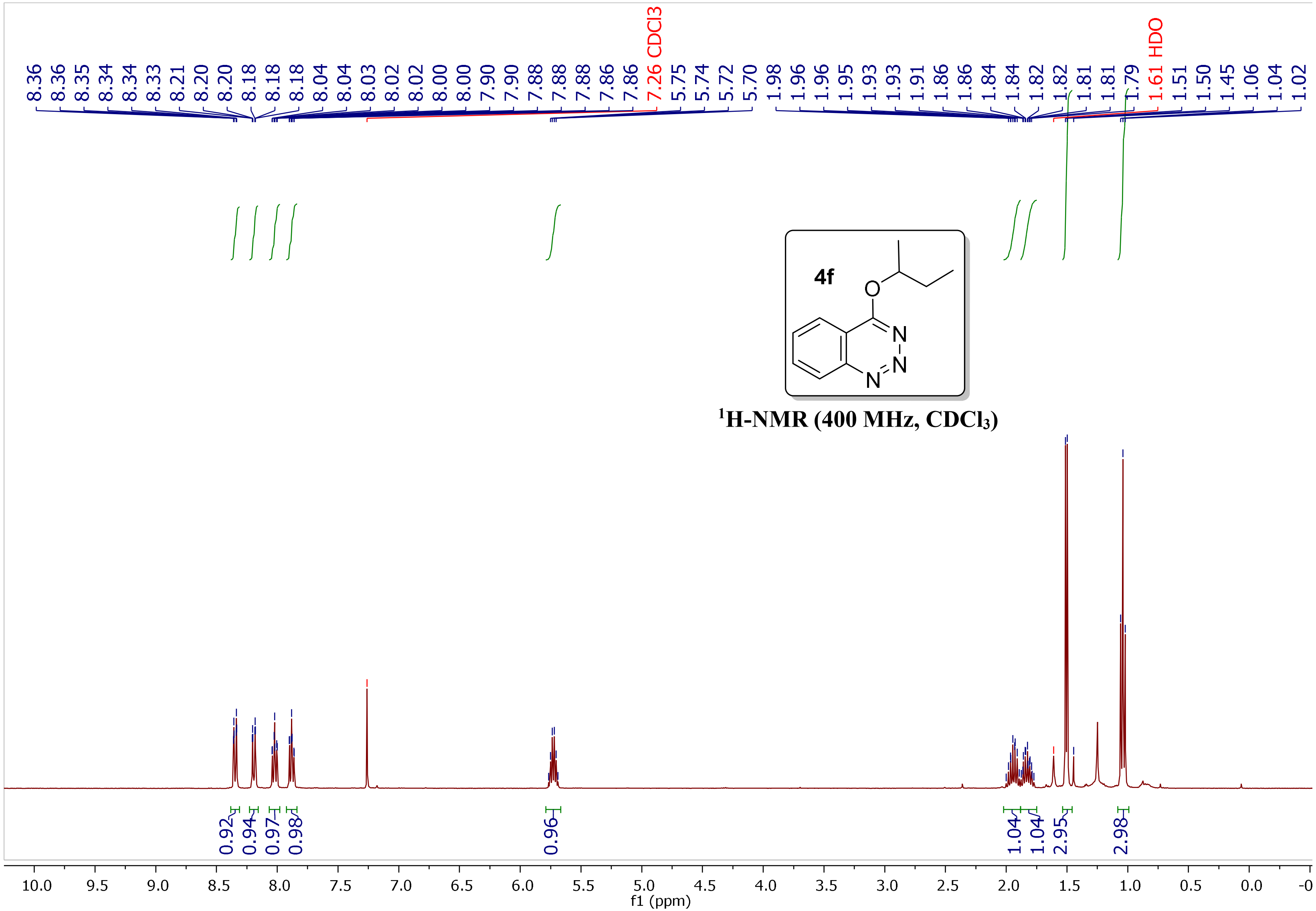
8.36 8.36 8.35 8.34 8.34 8.33 8.21 8.20 8.20 8.18 8.18 8.18 8.04 8.04 8.03 8.02 8.02 8.00 8.00 7.90 7.90 7.88 7.88 7.88 7.86 7.86 7.26 CDCl3 5.75 5.74 5.72 5.70 1.98 1.96 1.96 1.95 1.93 1.93 1.91 1.86 1.86 1.84 1.84 1.82 1.82 1.81 1.81 1.79 1.61 HDO 1.51 1.50 1.45 1.06 1.04 1.02
4f
O
N
N
N
¹H-NMR (400 MHz, CDCl₃)
0.92 0.94 0.97 0.98 0.96 1.04 1.04 2.95 2.98
10.0 9.5 9.0 8.5 8.0 7.5 7.0 6.5 6.0 5.5 5.0 4.5 4.0 3.5 3.0 2.5 2.0 1.5 1.0 0.5 0.0 -0
f1 (ppm)

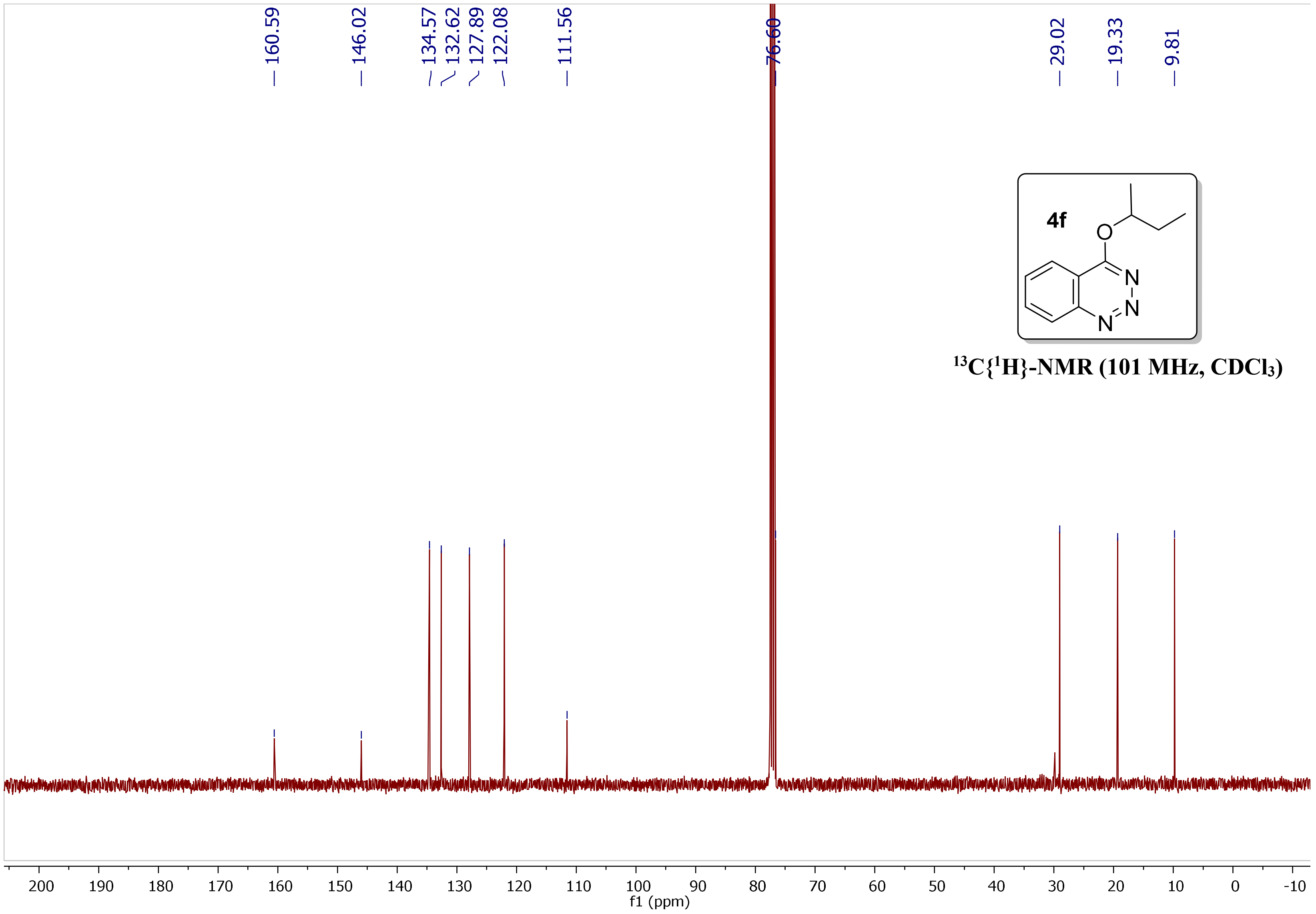

160.59
146.02
134.57
132.62
127.89
122.08
111.56
76.60
29.02
19.33
9.81
4f
O
N
N
N
¹³C{¹H}-NMR (101 MHz, CDCl₃)
200 190 180 170 160 150 140 130 120 110 100 90 80 70 60 50 40 30 20 10 0 -10
f1 (ppm)

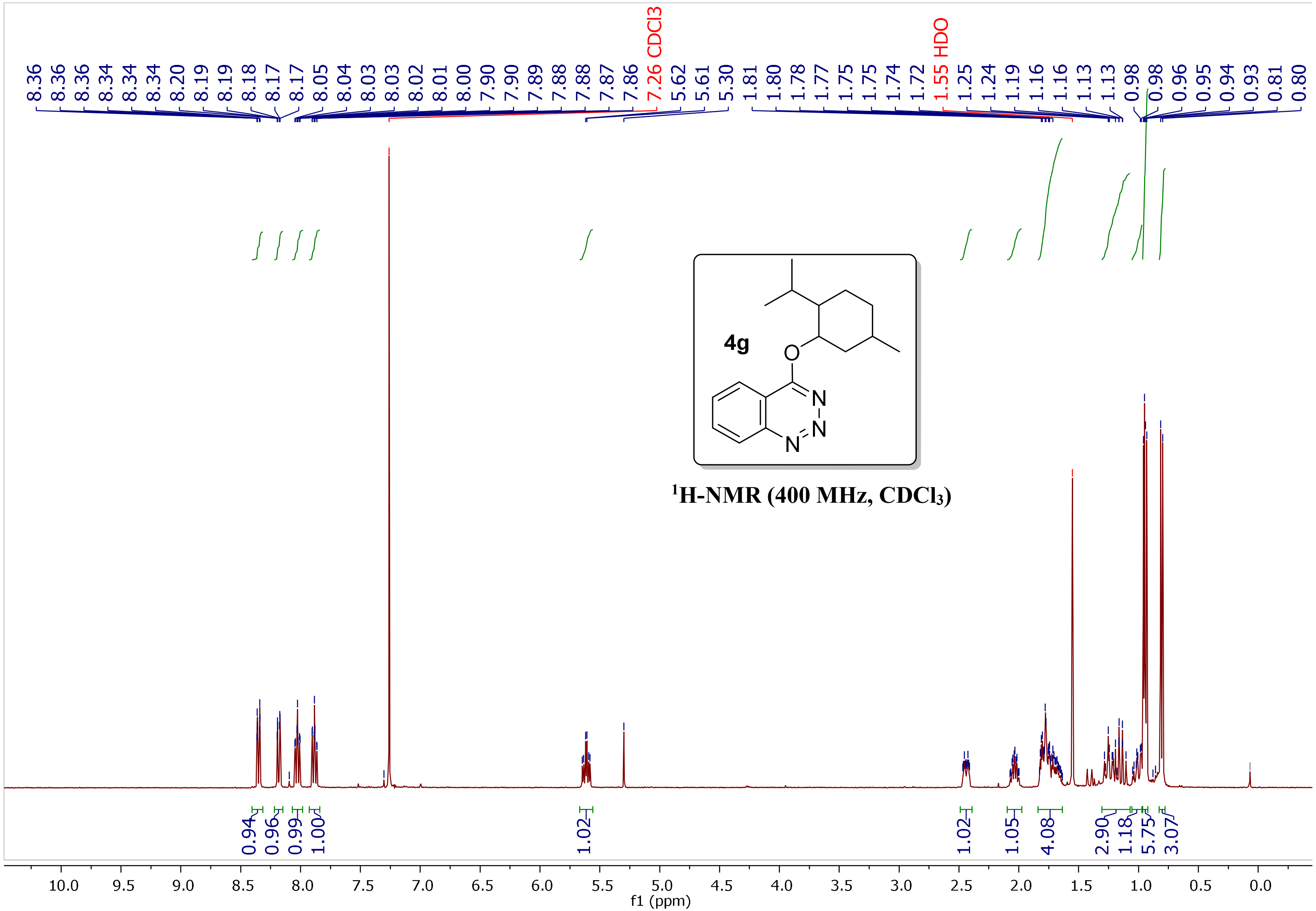

4g
¹H-NMR (400 MHz, CDCl₃)
7.26 CDCl3
1.55 HDO
f1 (ppm)

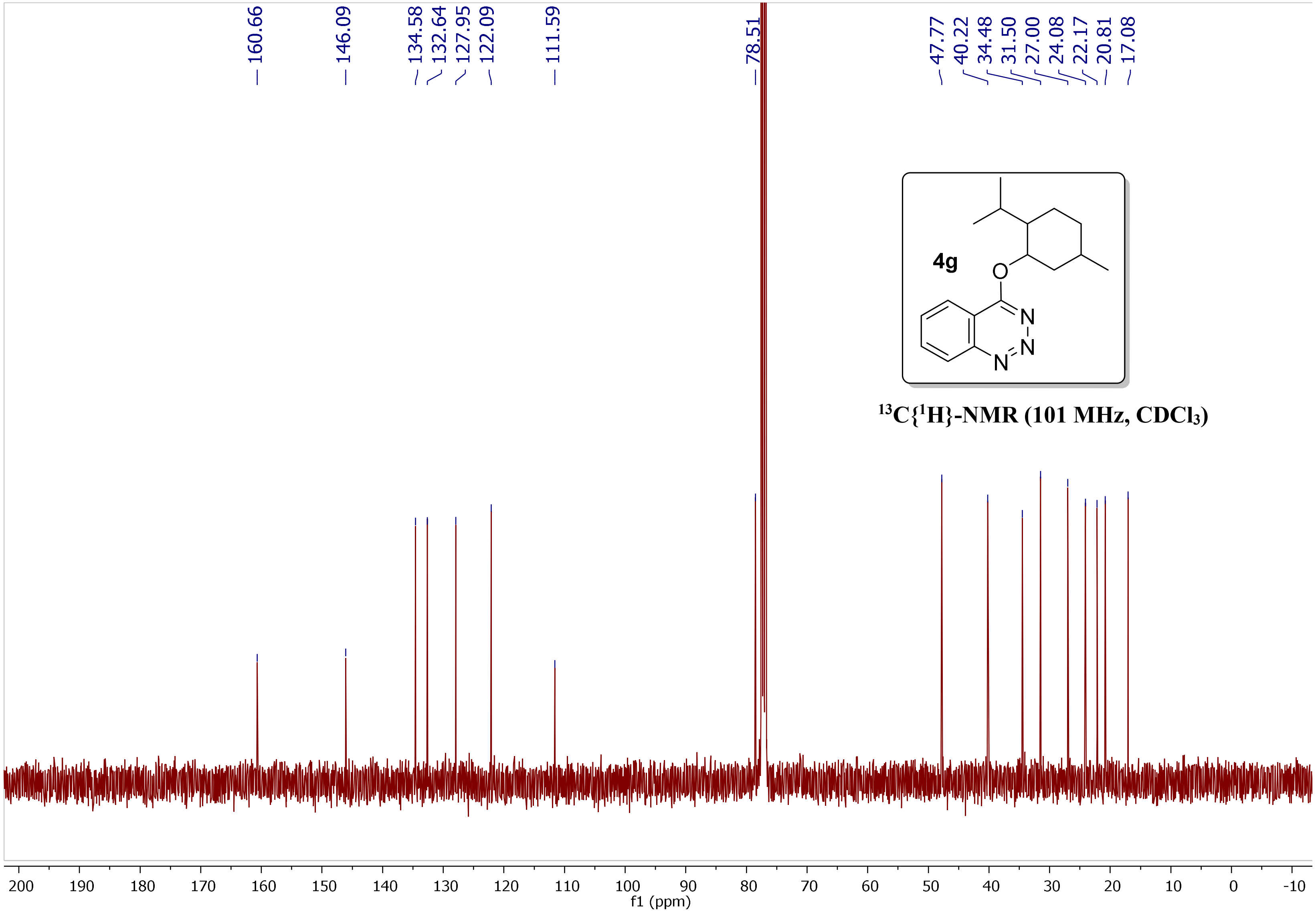
160.66
146.09
134.58
132.64
127.95
122.09
111.59
78.51
47.77
40.22
34.48
31.50
27.00
24.08
22.17
20.81
17.08
4g
O
N
N
N
$^{13}C\{^{1}H\}$-NMR (101 MHz, $CDCl_3$)
200 190 180 170 160 150 140 130 120 110 100 90 80 70 60 50 40 30 20 10 0 -10
f1 (ppm)

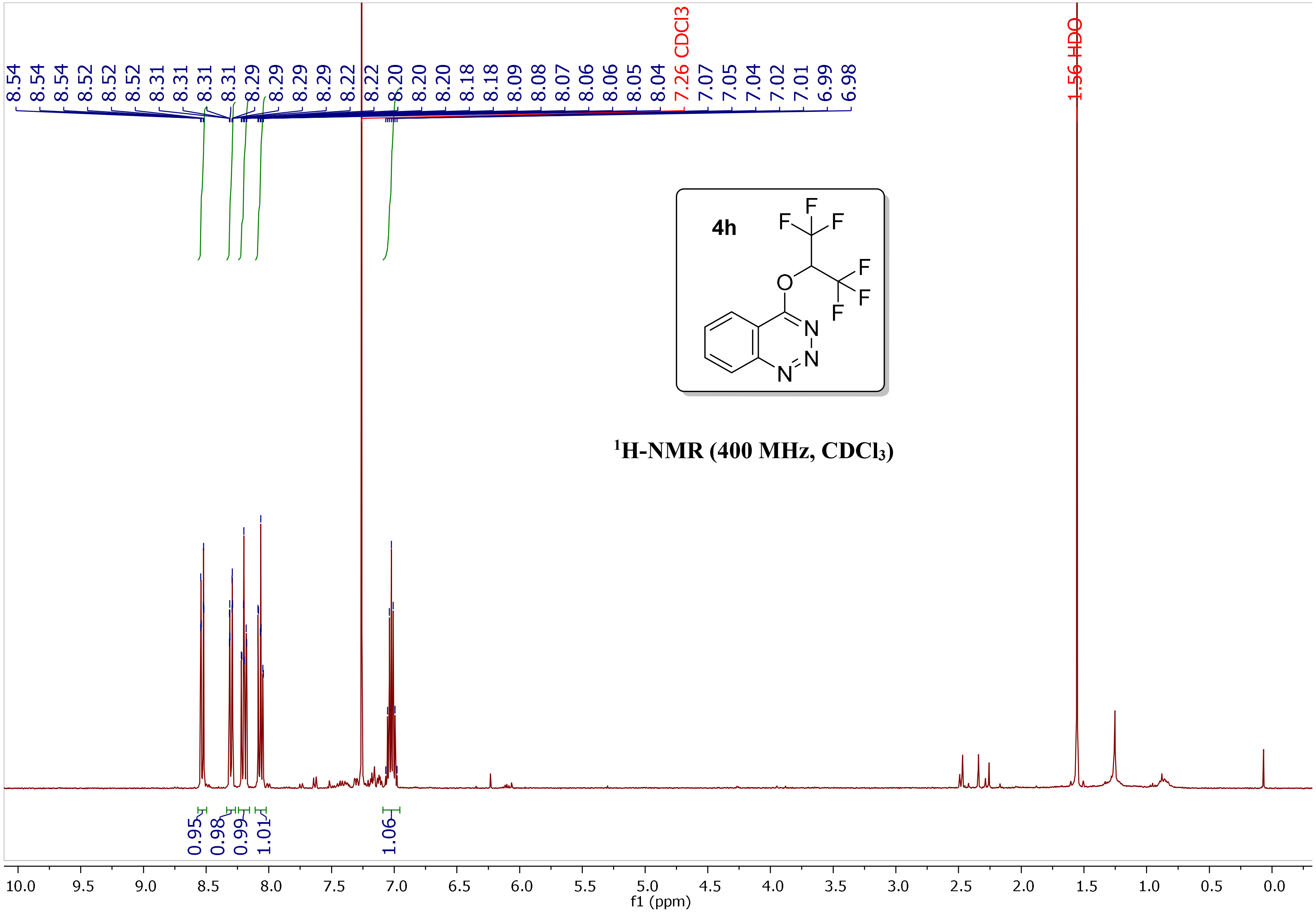
4h
^{1}H-NMR (400 MHz, CDCl$_3$)
8.54
8.54
8.54
8.52
8.52
8.52
8.31
8.31
8.31
8.31
8.29
8.29
8.29
8.29
8.22
8.22
8.20
8.20
8.20
8.18
8.18
8.09
8.08
8.07
8.06
8.06
8.05
8.04
7.26 CDCl3
7.07
7.05
7.04
7.02
7.01
6.99
6.98
1.56 HDO
0.95
0.98
0.99
1.01
1.06
f1 (ppm)

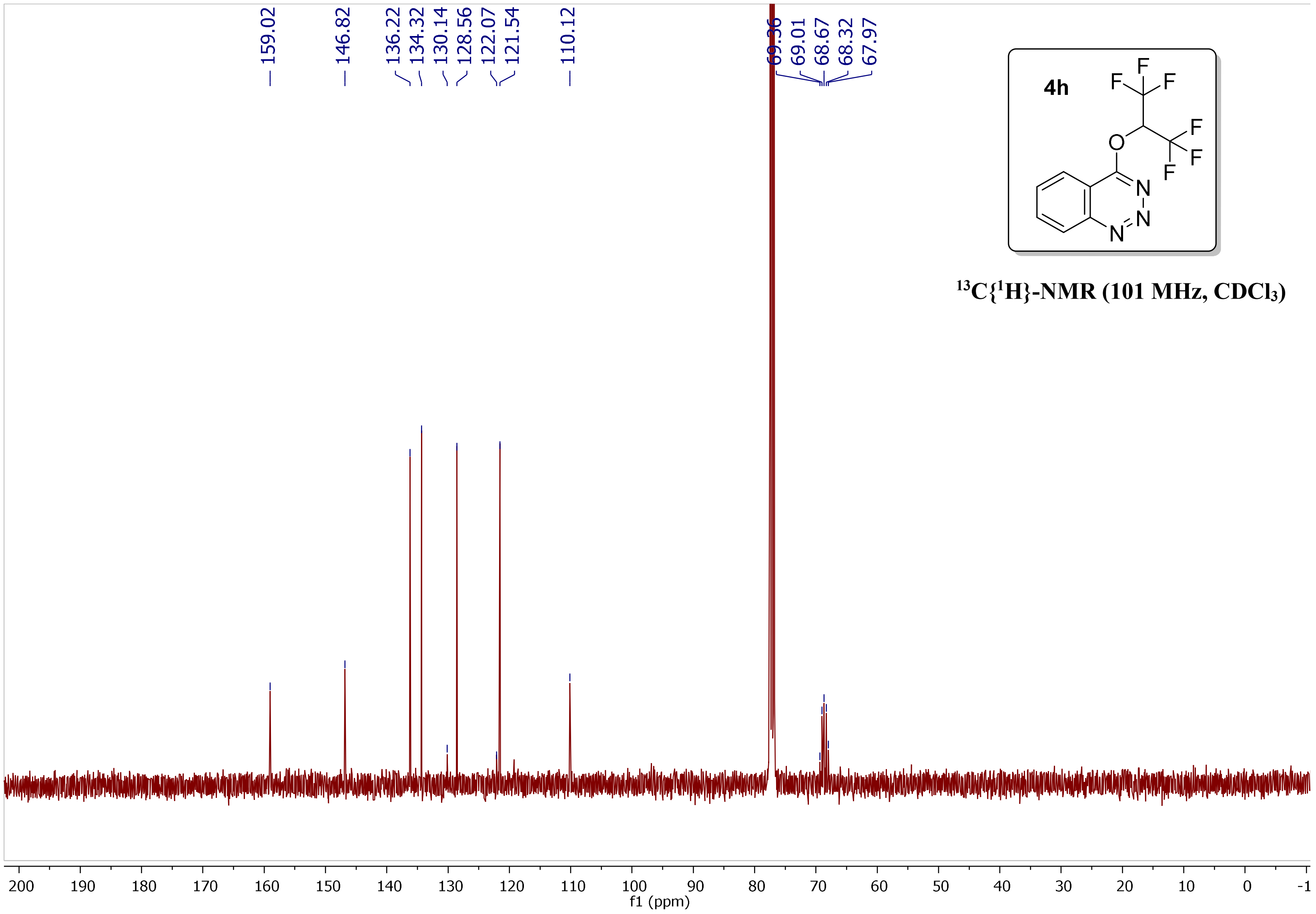
159.02
146.82
136.22
134.32
130.14
128.56
122.07
121.54
110.12
69.36
69.01
68.67
68.32
67.97
4h
F
O
N
N
N
$^{13}C\{^1H\}$-NMR (101 MHz, $CDCl_3$)
200
190
180
170
160
150
140
130
120
110
100
90
80
70
60
50
40
30
20
10
0
-1
f1 (ppm)

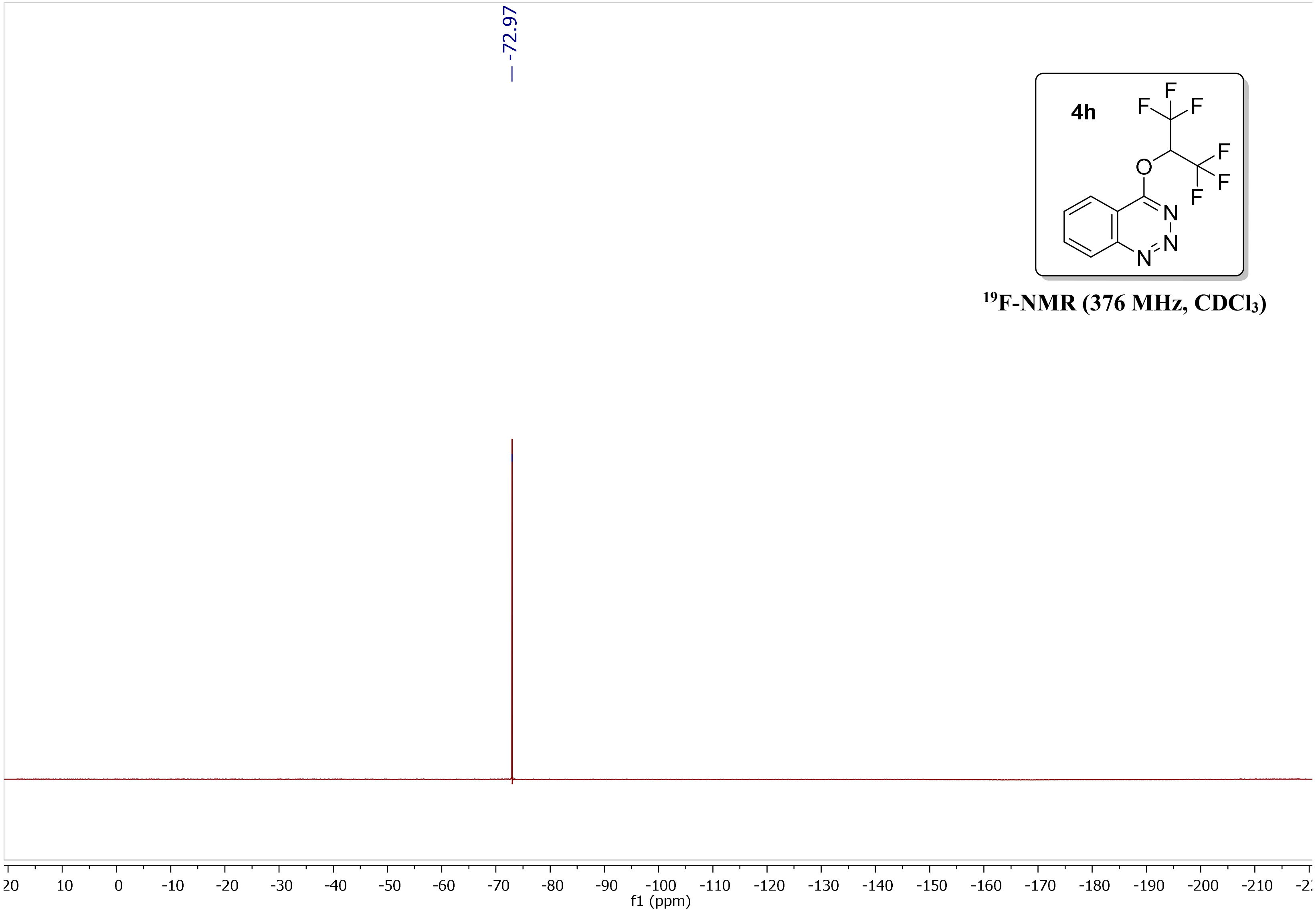
-72.97
4h
F F F O F F F N N N
19F-NMR (376 MHz, CDCl3)
20 10 0 -10 -20 -30 -40 -50 -60 -70 -80 -90 -100 -110 -120 -130 -140 -150 -160 -170 -180 -190 -200 -210
f1 (ppm)

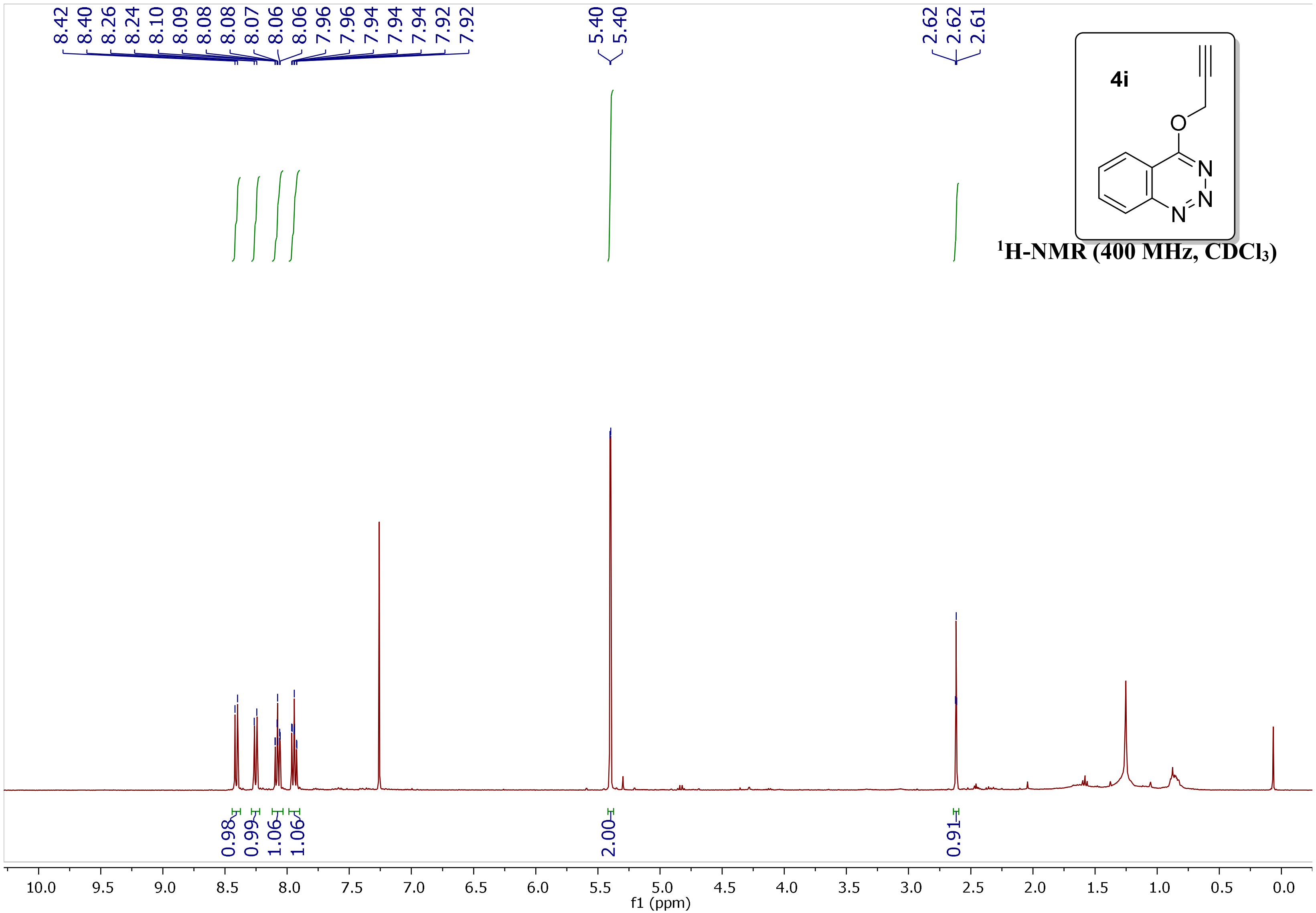

^{1}H-NMR (400 MHz, $CDCl_3$)

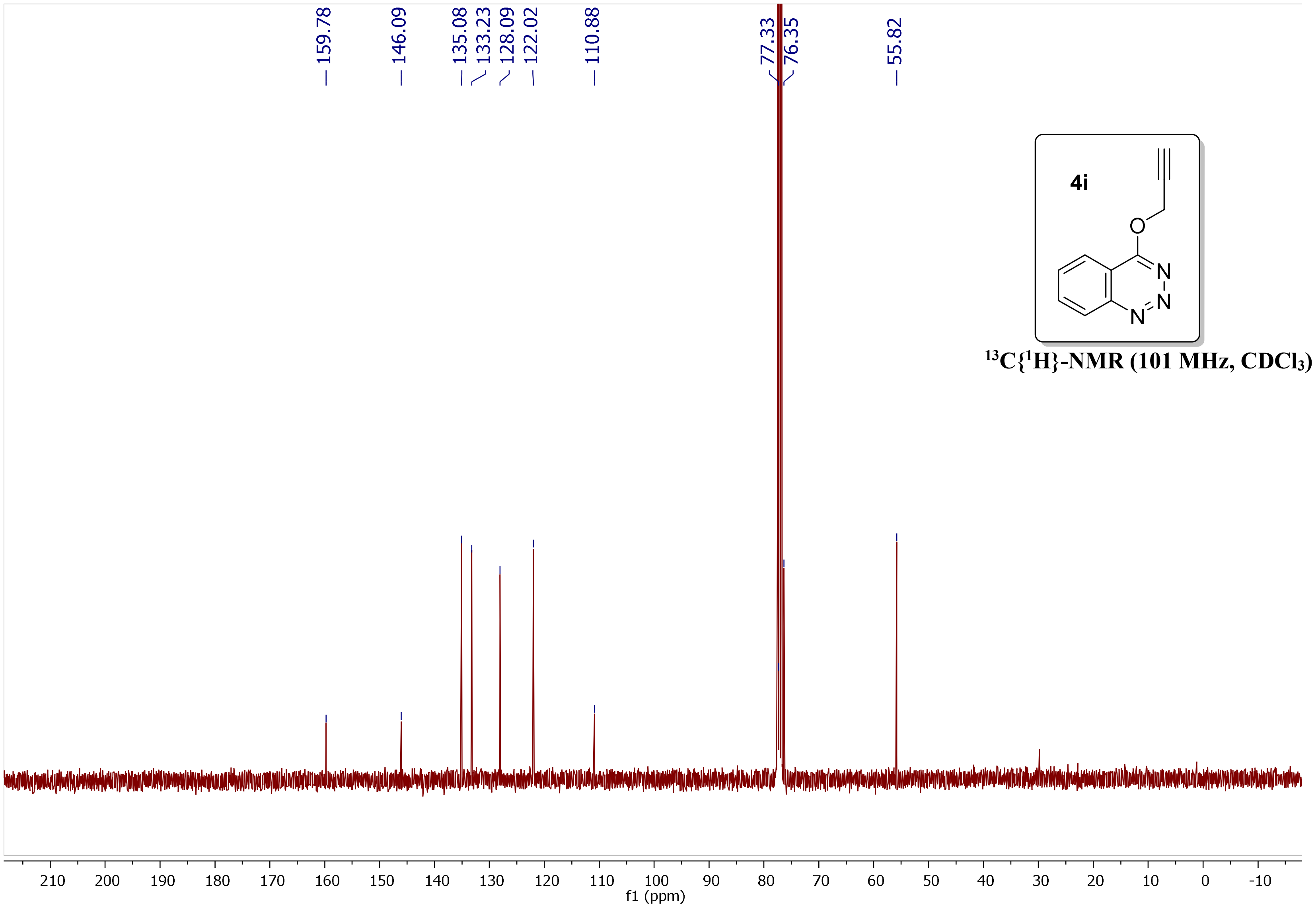
159.78
146.09
135.08
133.23
128.09
122.02
110.88
77.33
76.35
55.82
4i
O
N
N
N
13C{1H}-NMR (101 MHz, CDCl3)
210
200
190
180
170
160
150
140
130
120
110
100
90
80
70
60
50
40
30
20
10
0
-10
f1 (ppm)

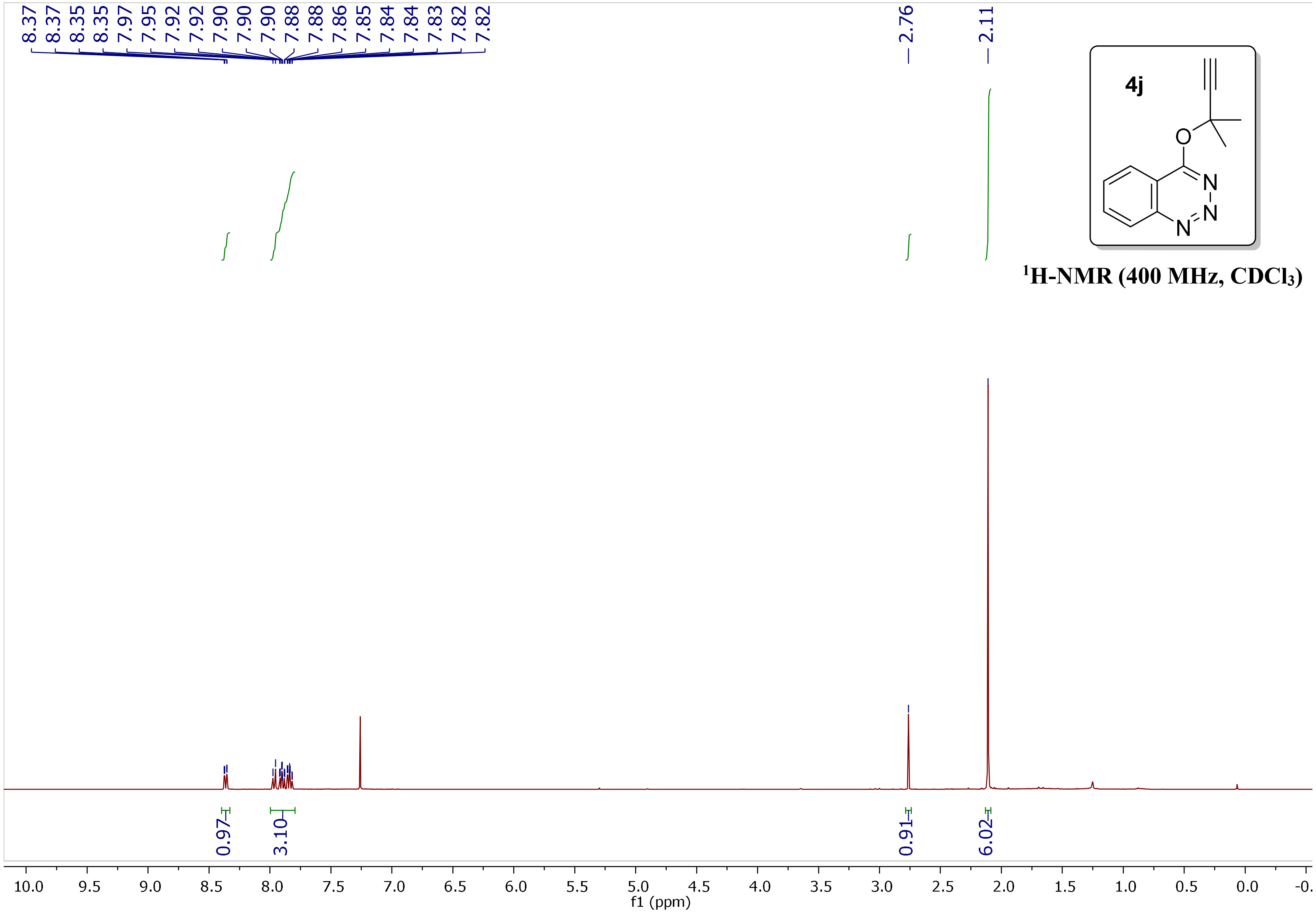

^{1}H-NMR (400 MHz, $CDCl_3$)

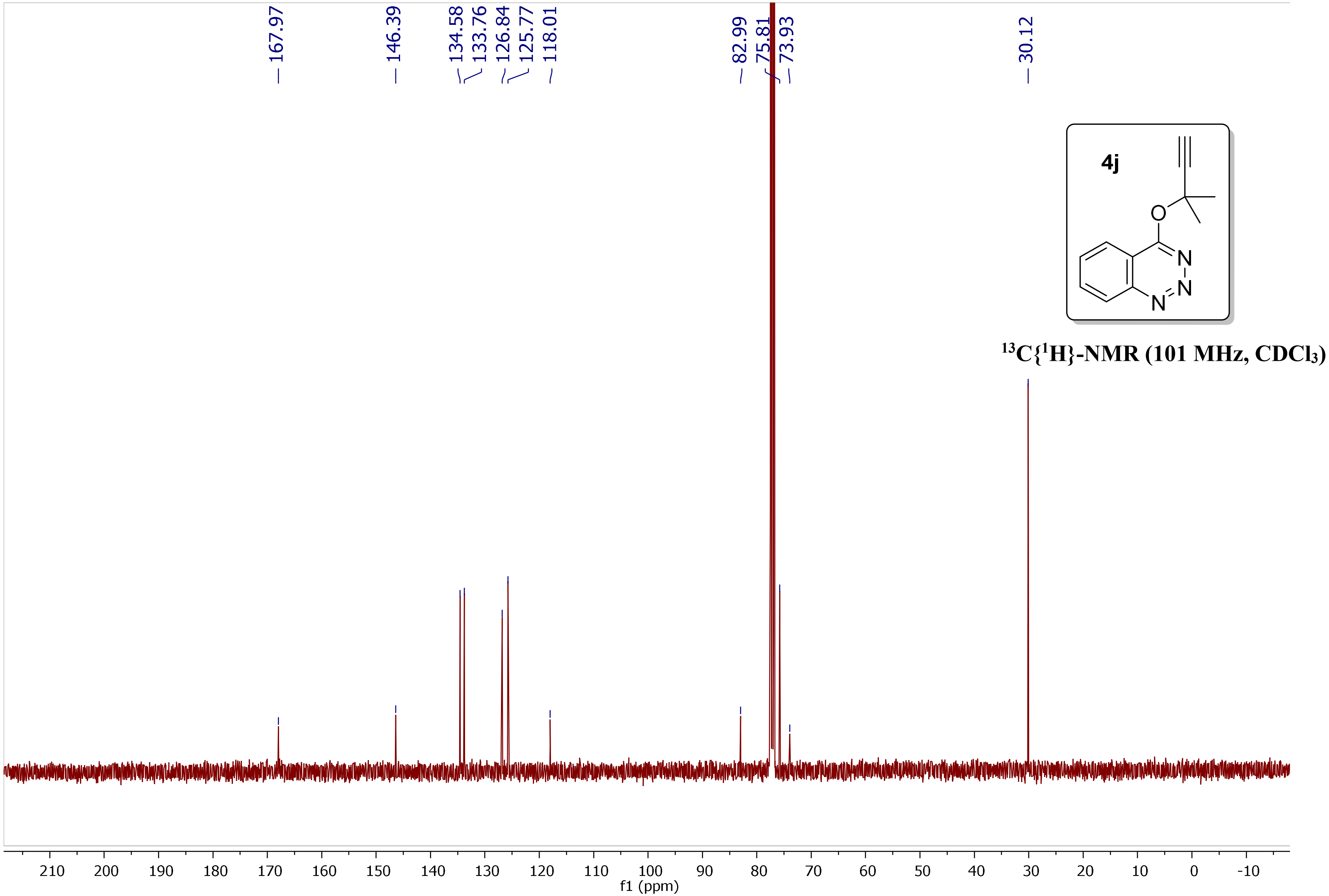
167.97
146.39
134.58
133.76
126.84
125.77
118.01
82.99
75.81
73.93
30.12
4j
O
N
N
N
^{13}C{^{1}H}-NMR (101 MHz, CDCl$_3$)
210 200 190 180 170 160 150 140 130 120 110 100 90 80 70 60 50 40 30 20 10 0 -10
f1 (ppm)

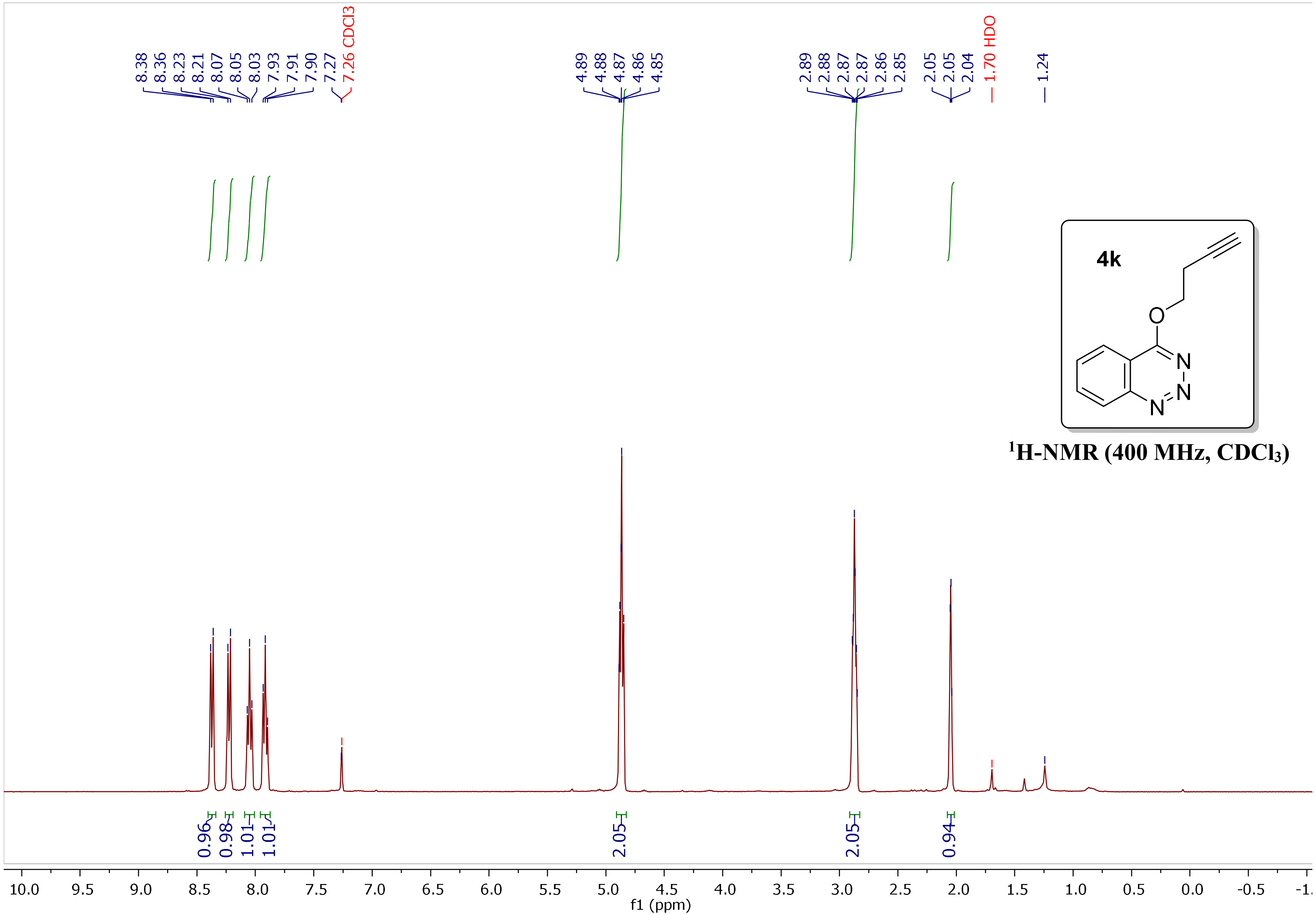

^{1}H-NMR (400 MHz, $CDCl_3$)

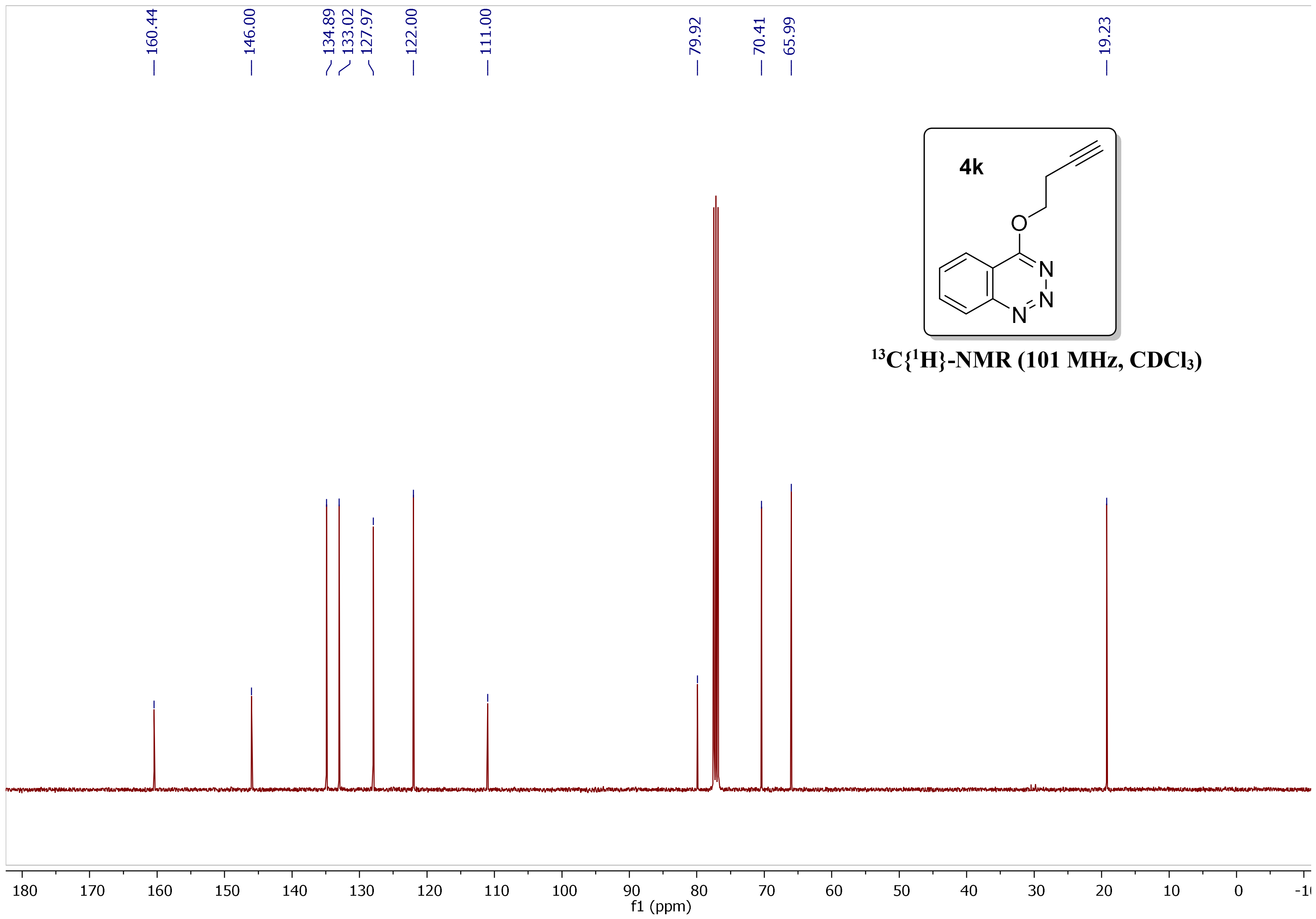
160.44
146.00
134.89
133.02
127.97
122.00
111.00
79.92
70.41
65.99
19.23
4k
O
N
N
N
$^{13}C\{^{1}H\}$-NMR (101 MHz, $CDCl_3$)
180
170
160
150
140
130
120
110
100
90
80
70
60
50
40
30
20
10
0
f1 (ppm)

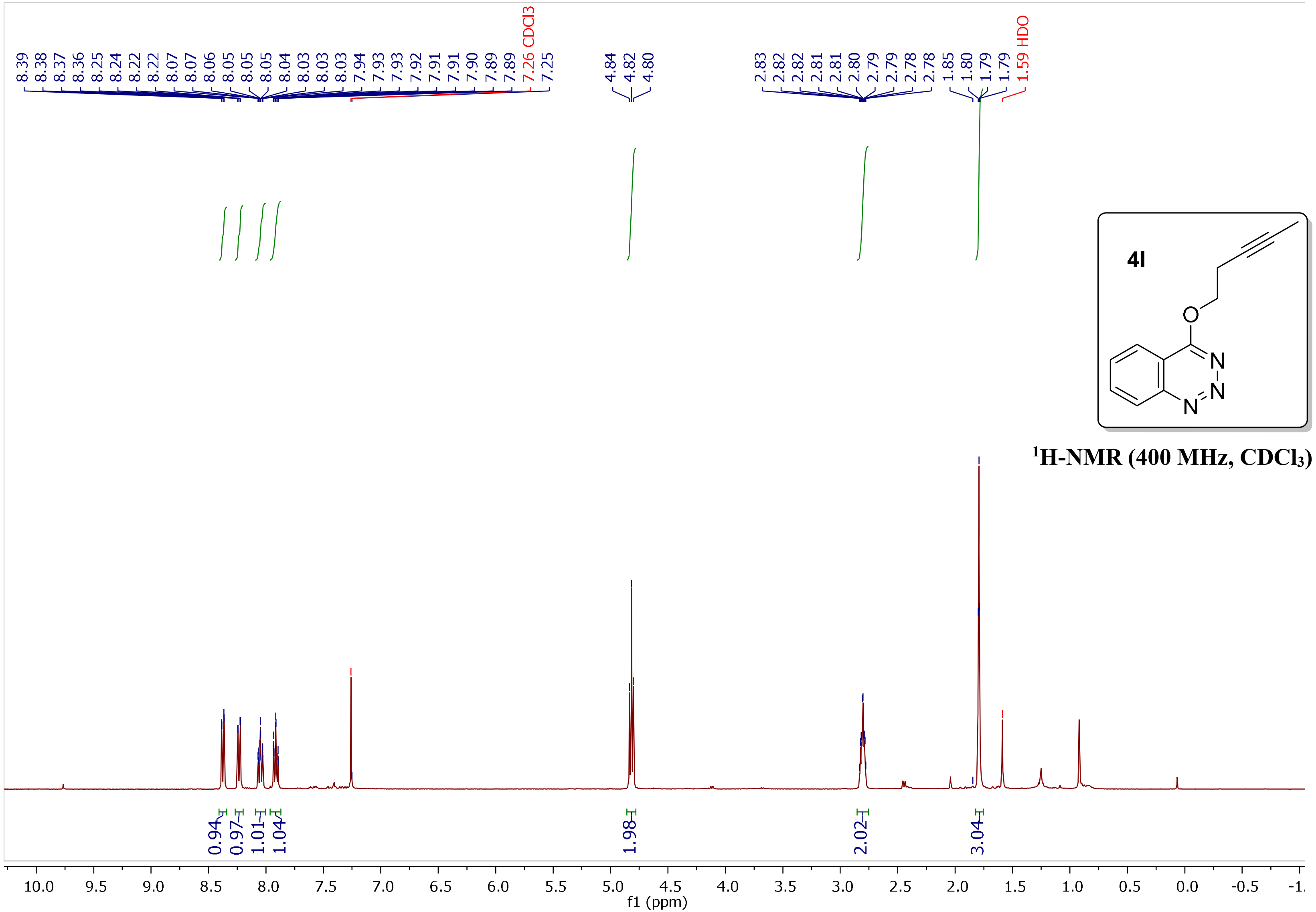

^{1}H-NMR (400 MHz, $CDCl_3$)

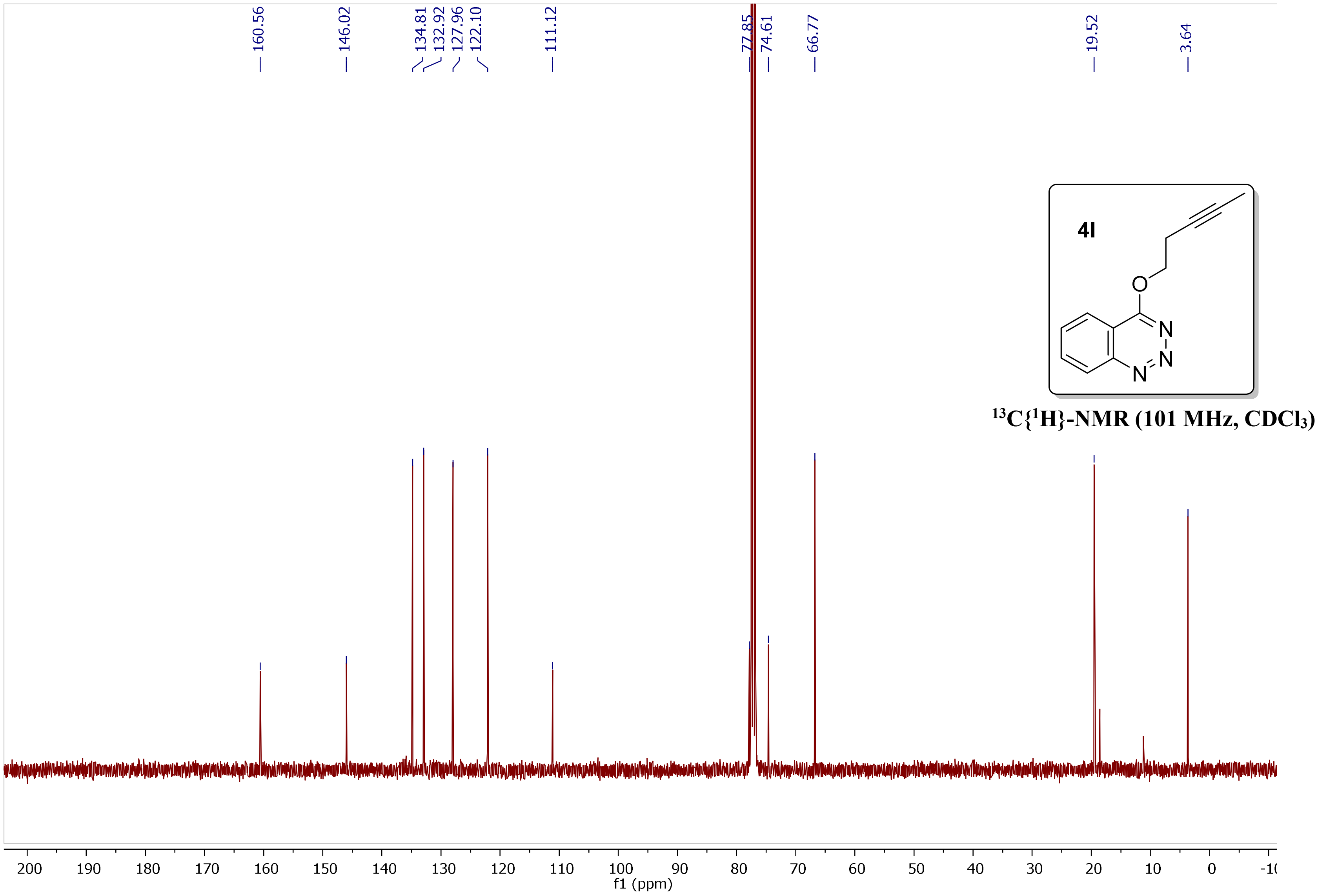
160.56
146.02
134.81
132.92
127.96
122.10
111.12
77.85
74.61
66.77
19.52
3.64
4l
O
N
N
N
13C{1H}-NMR (101 MHz, CDCl3)
f1 (ppm)

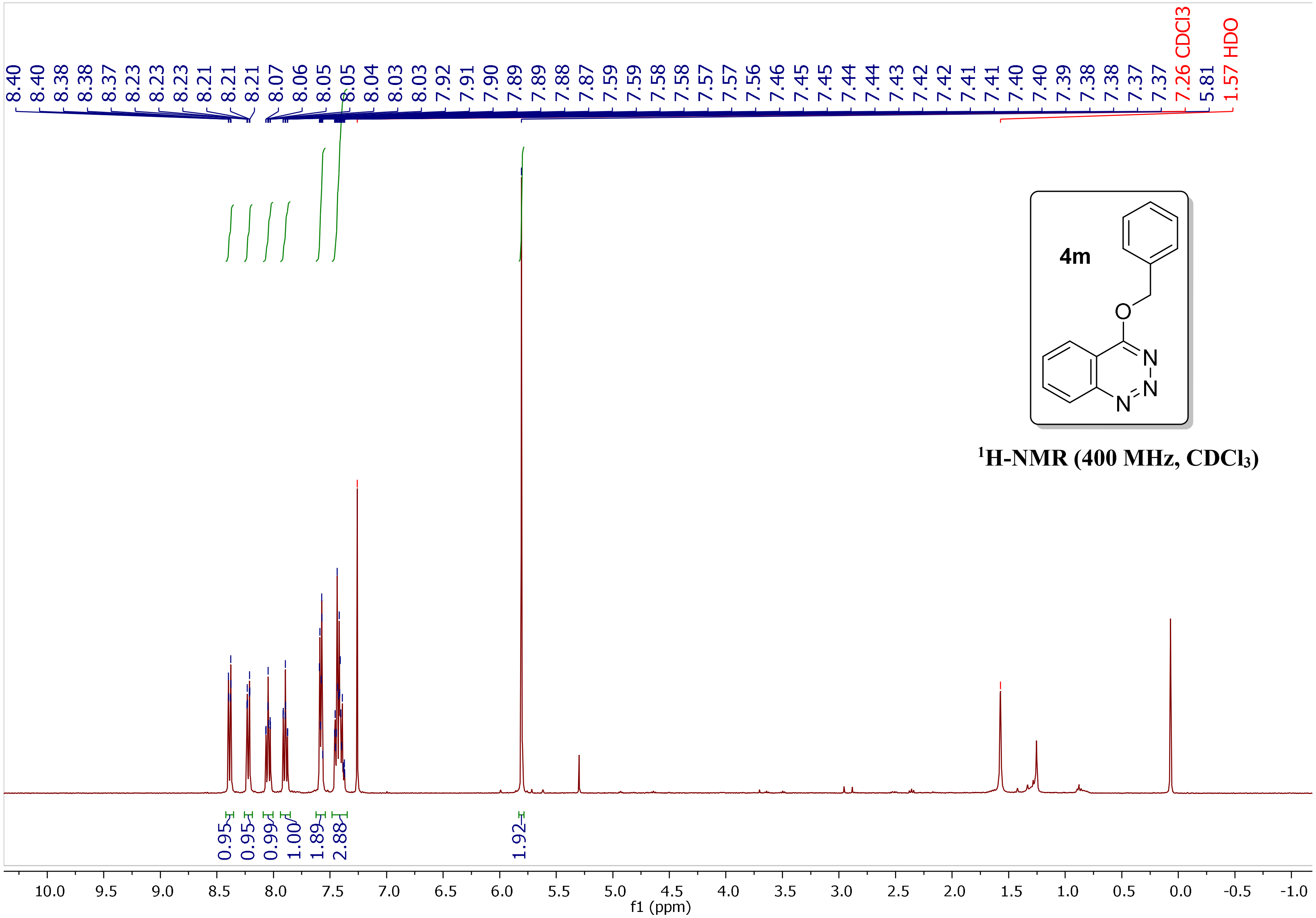
4m
1H-NMR (400 MHz, CDCl3)
f1 (ppm)
7.26 CDCl3
1.57 HDO
5.81

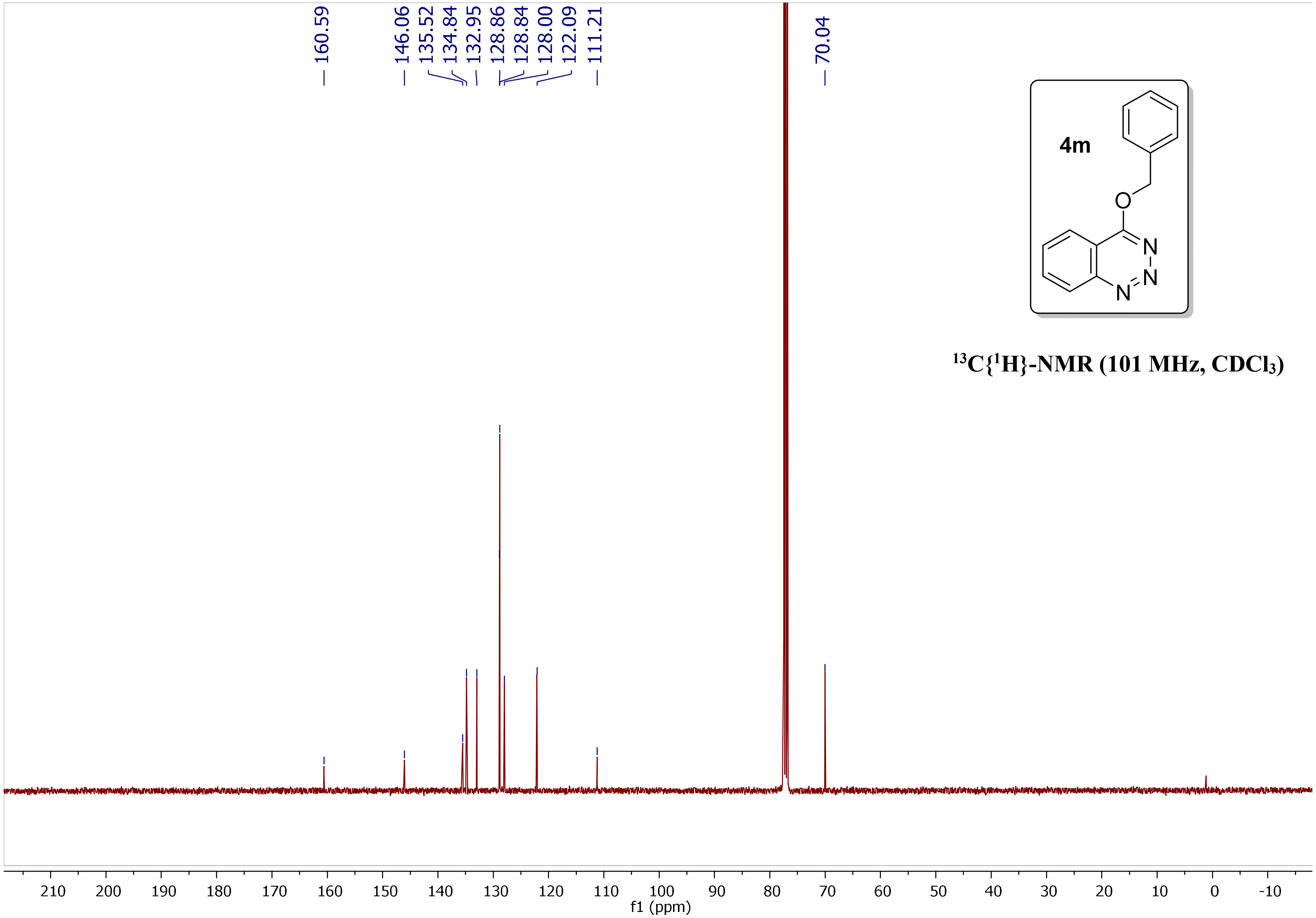

$^{13}C\{^{1}H\}$-NMR (101 MHz, $CDCl_3$)

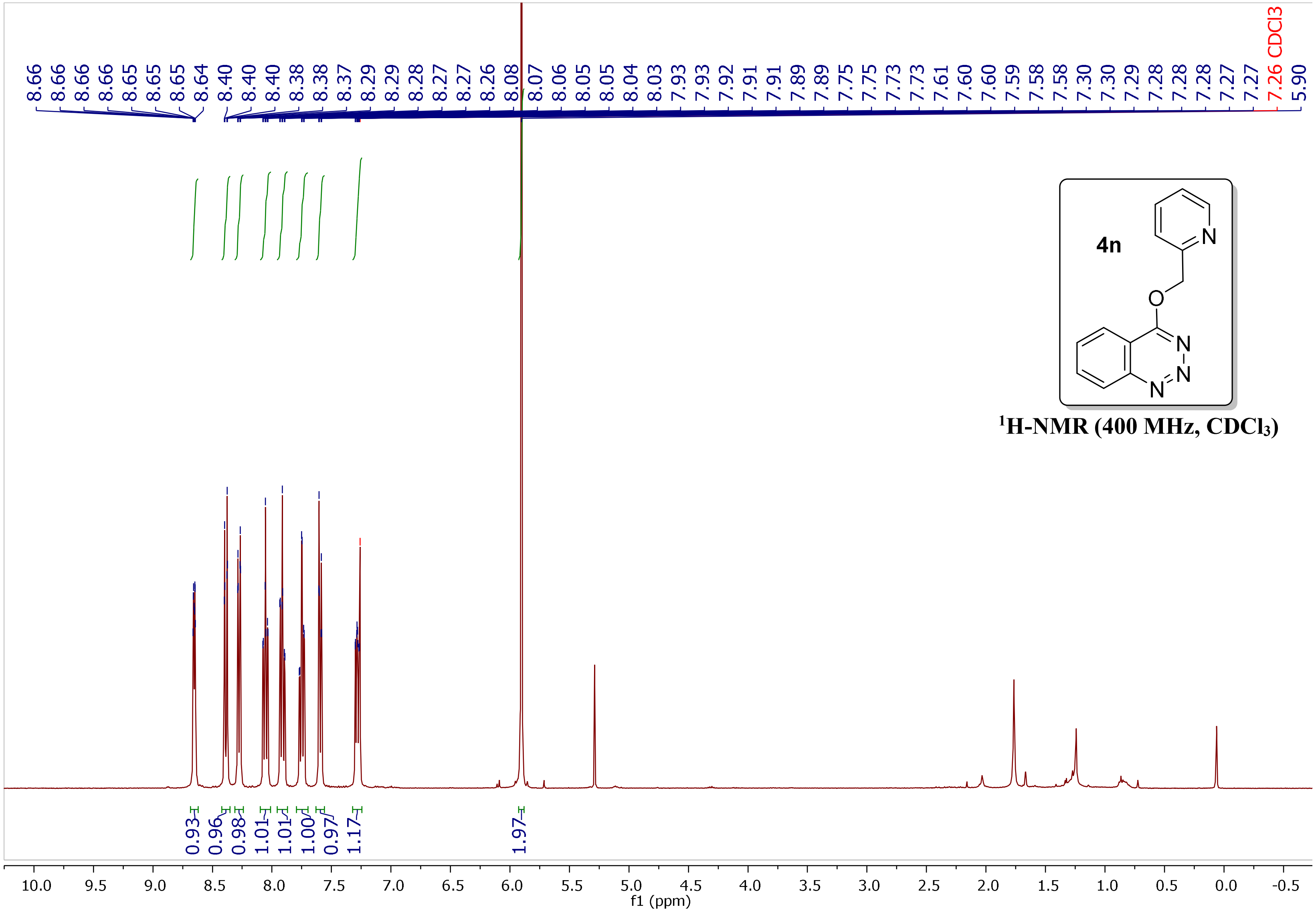

^{1}H-NMR (400 MHz, $CDCl_3$)

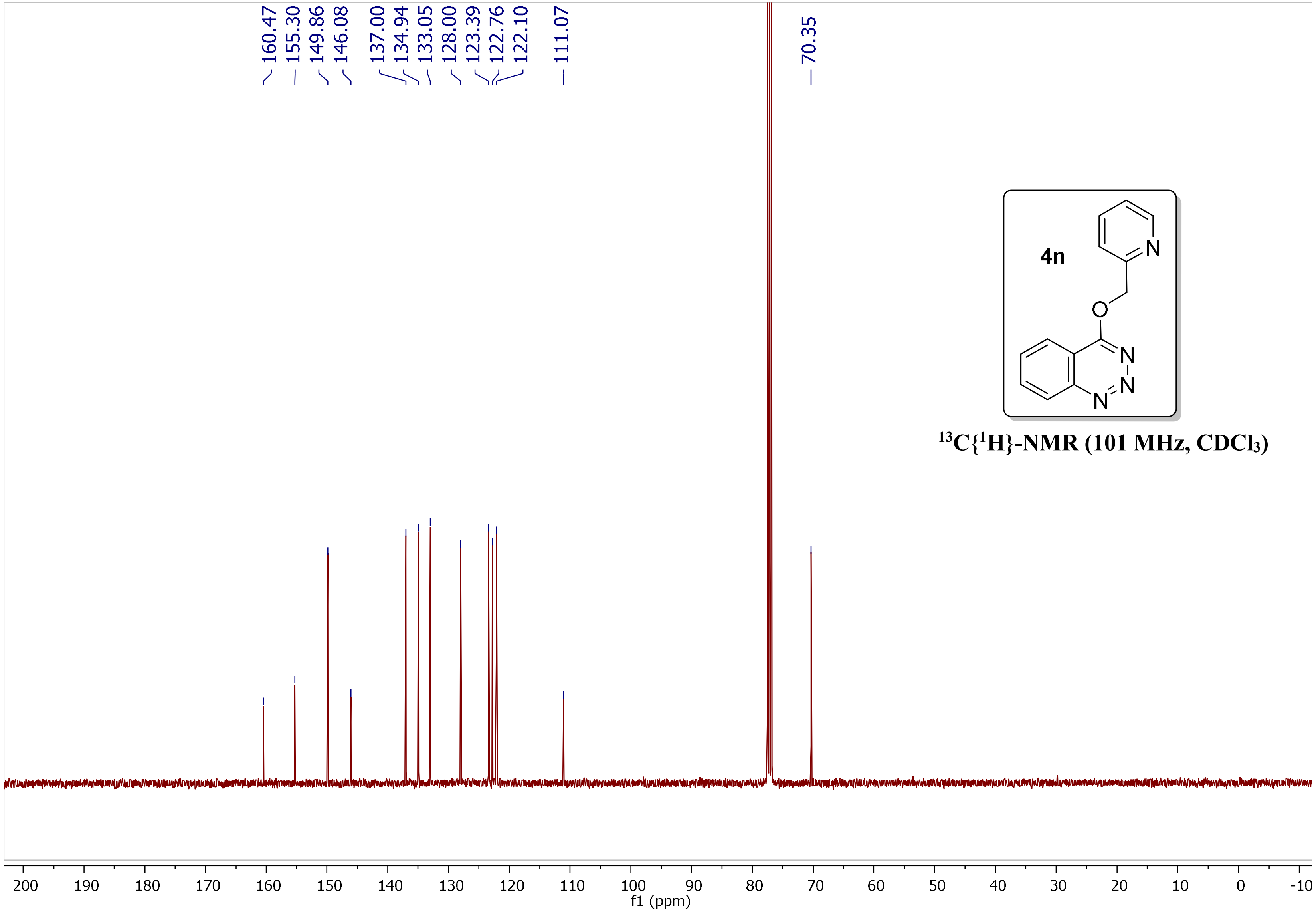
160.47
155.30
149.86
146.08
137.00
134.94
133.05
128.00
123.39
122.76
122.10
111.07
70.35
4n
N
O
N
N
N
13C{1H}-NMR (101 MHz, CDCl3)
200 190 180 170 160 150 140 130 120 110 100 90 80 70 60 50 40 30 20 10 0 -10
f1 (ppm)

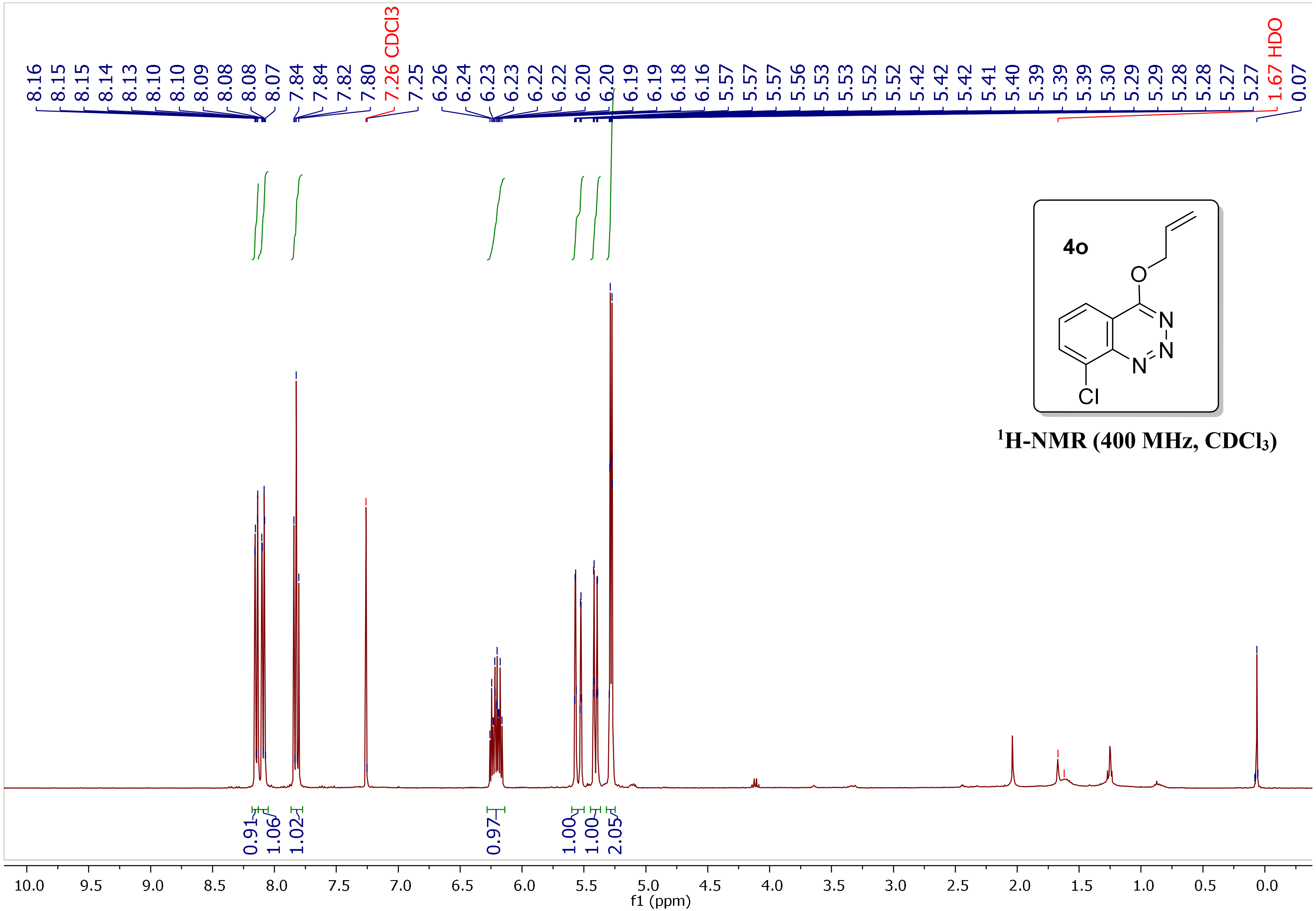

^{1}H-NMR (400 MHz, $CDCl_3$)

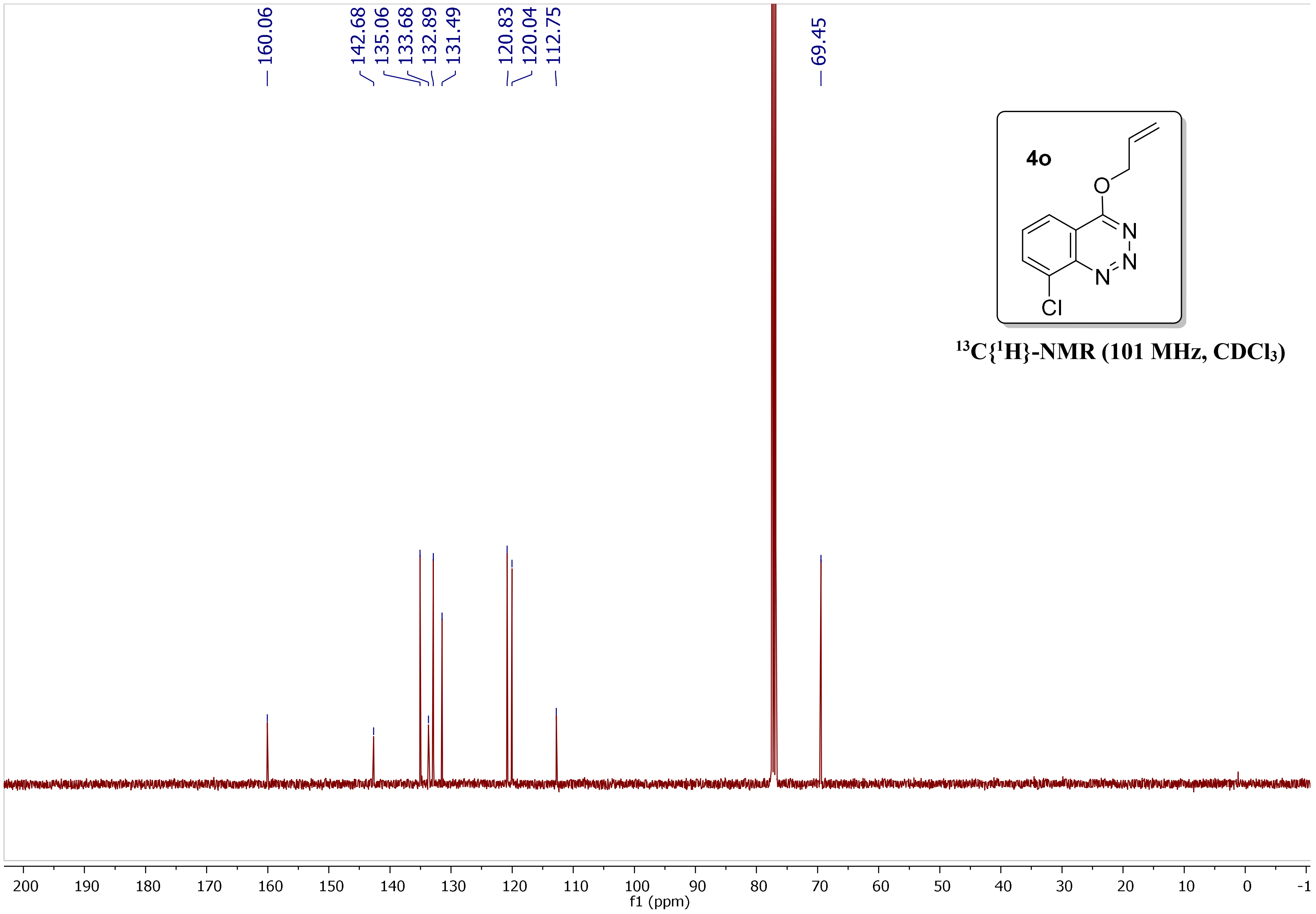
160.06
142.68
135.06
133.68
132.89
131.49
120.83
120.04
112.75
69.45
4o
O
N
N
N
Cl
$^{13}C\{^1H\}$-NMR (101 MHz, $CDCl_3$)
200
190
180
170
160
150
140
130
120
110
100
90
80
70
60
50
40
30
20
10
0
-1
f1 (ppm)

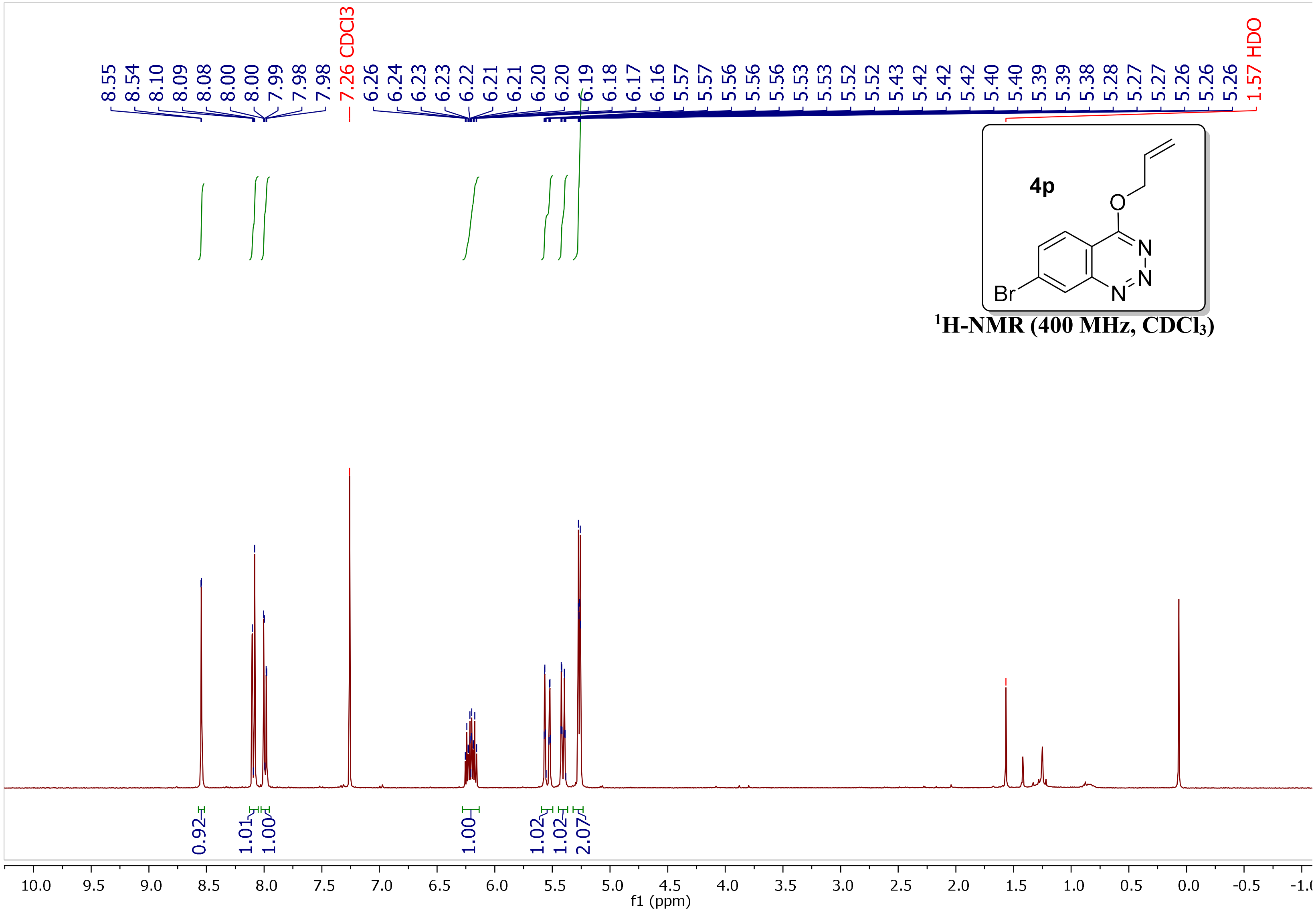

8.55
8.54
8.10
8.09
8.08
8.00
8.00
7.99
7.98
7.98
7.26 CDCl3
6.26
6.24
6.23
6.23
6.22
6.21
6.21
6.20
6.20
6.19
6.18
6.17
6.16
5.57
5.57
5.56
5.56
5.56
5.53
5.53
5.52
5.52
5.43
5.42
5.42
5.40
5.40
5.39
5.39
5.38
5.28
5.27
5.27
5.26
5.26
5.26
1.57 HDO
4p
O
N
N
N
Br
¹H-NMR (400 MHz, CDCl₃)
0.92
1.01
1.00
1.00
1.02
1.02
2.07
10.0
9.5
9.0
8.5
8.0
7.5
7.0
6.5
6.0
5.5
5.0
4.5
4.0
3.5
3.0
2.5
2.0
1.5
1.0
0.5
0.0
-0.5
f1 (ppm)

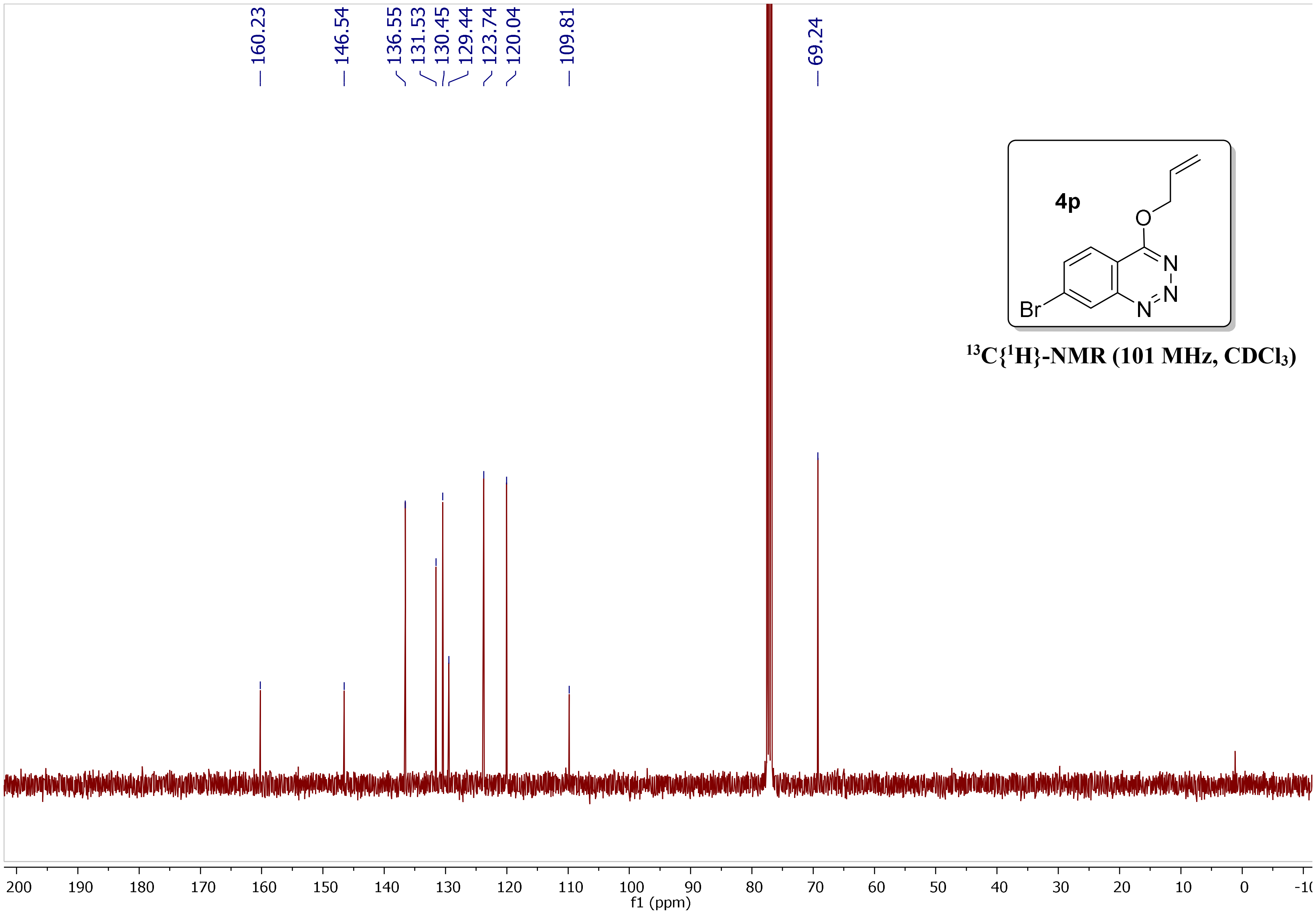

$^{13}C\{^{1}H\}$-NMR (101 MHz, $CDCl_3$)

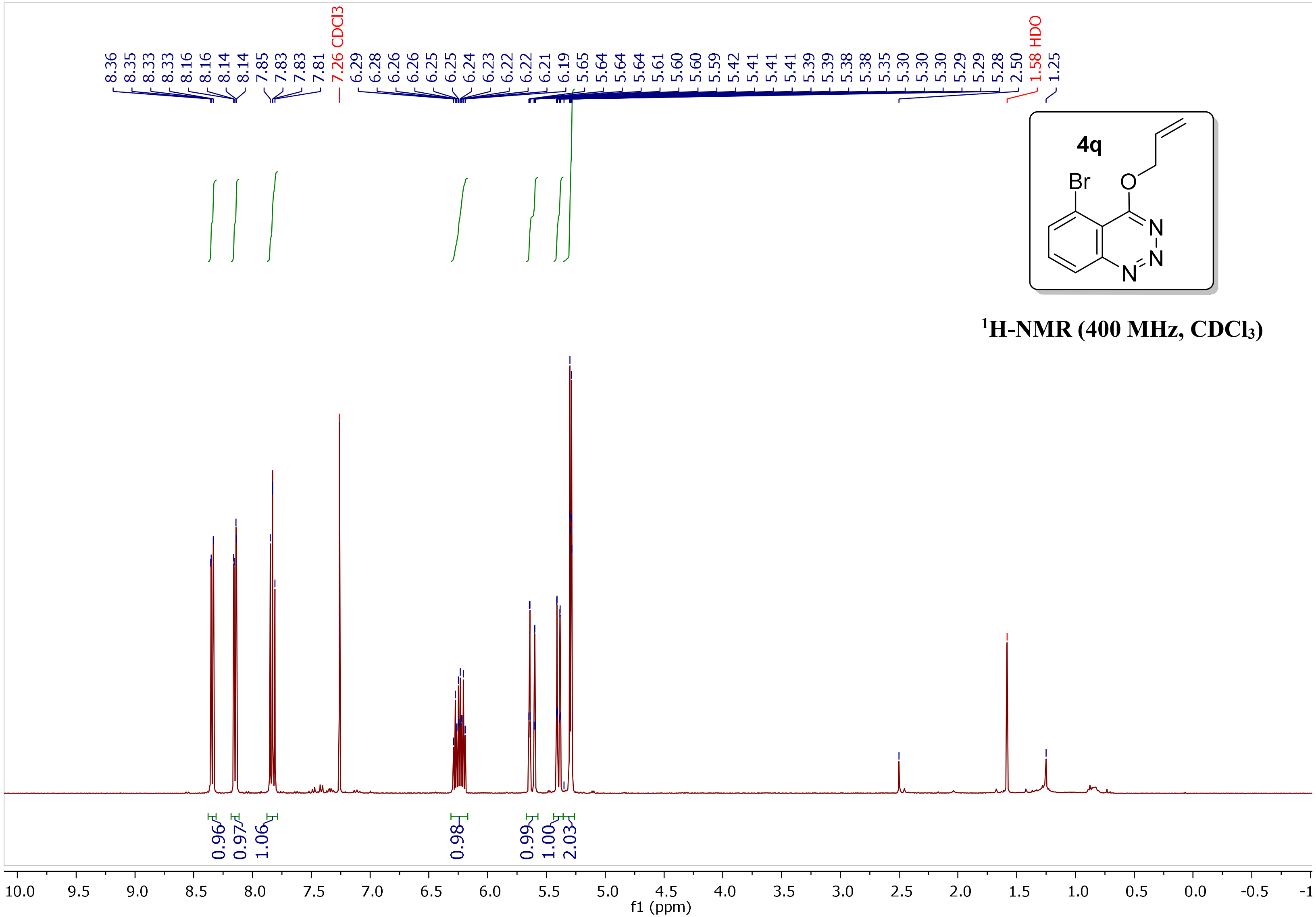

^{1}H-NMR (400 MHz, $CDCl_3$)

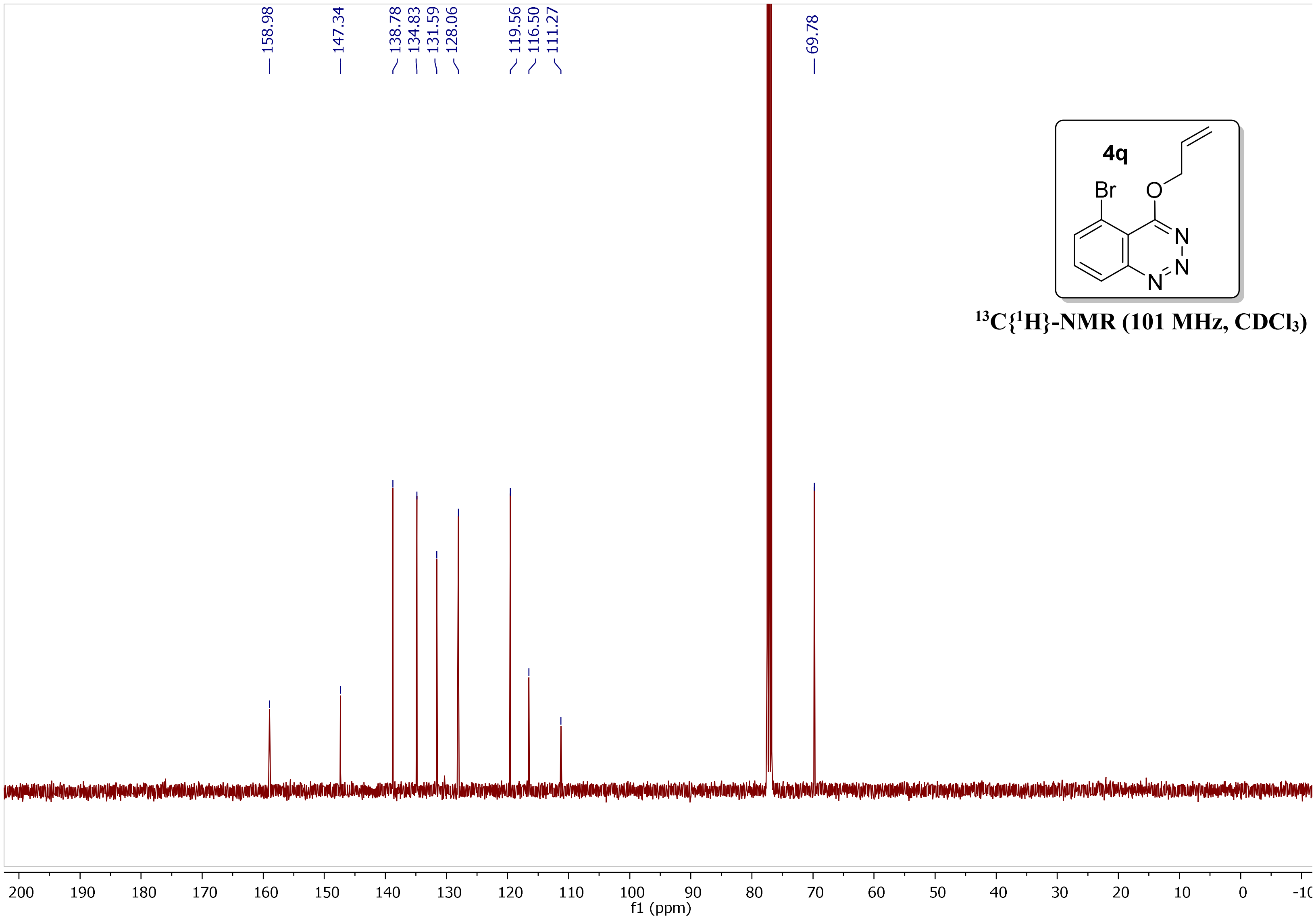
158.98
147.34
138.78
134.83
131.59
128.06
119.56
116.50
111.27
69.78
4q
Br
O
N
N
N
13C{1H}-NMR (101 MHz, CDCl3)
200 190 180 170 160 150 140 130 120 110 100 90 80 70 60 50 40 30 20 10 0 -10
f1 (ppm)

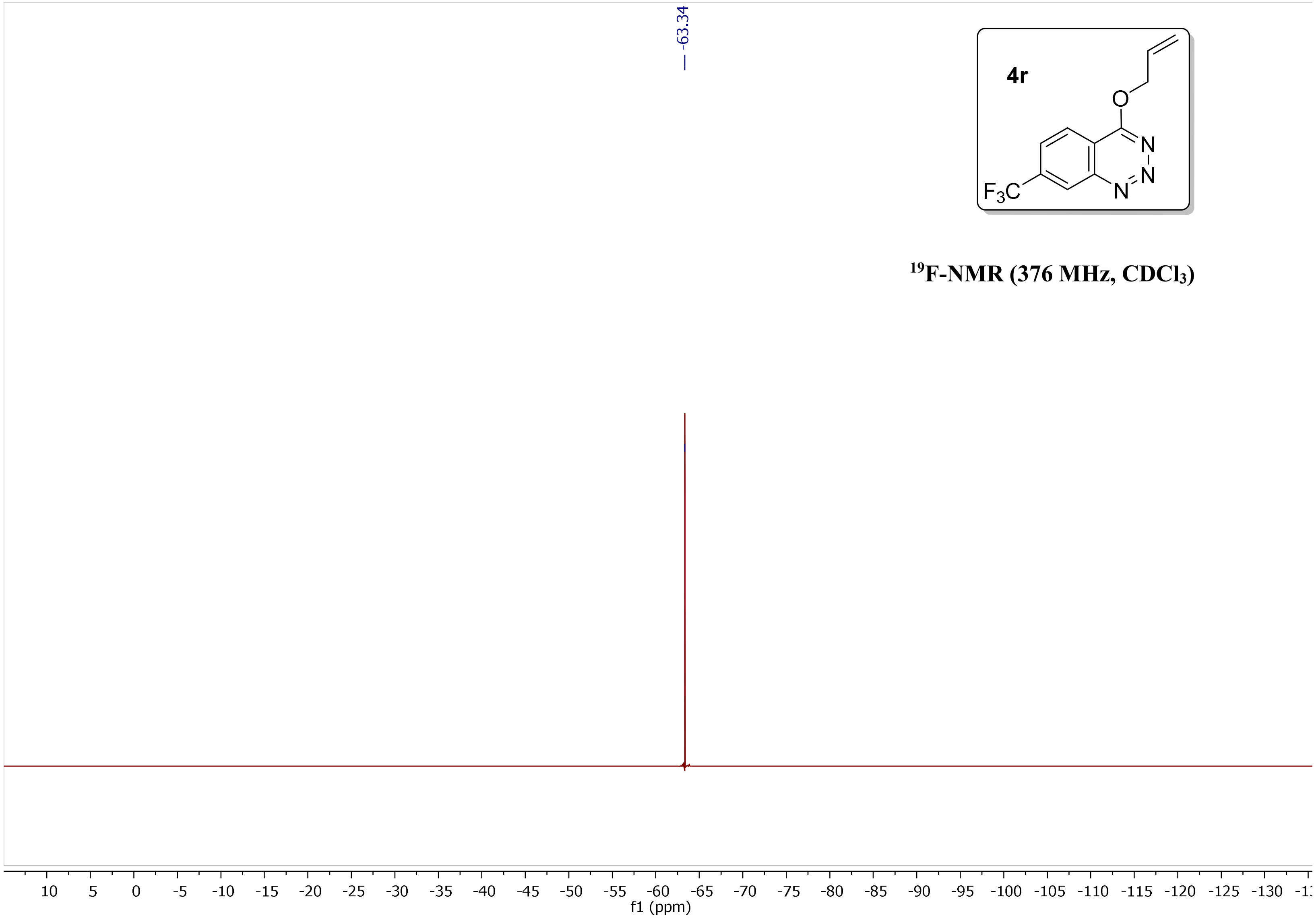
-63.34
4r
O
N
N
N
F3C
19F-NMR (376 MHz, CDCl3)
10 5 0 -5 -10 -15 -20 -25 -30 -35 -40 -45 -50 -55 -60 -65 -70 -75 -80 -85 -90 -95 -100 -105 -110 -115 -120 -125 -130
f1 (ppm)

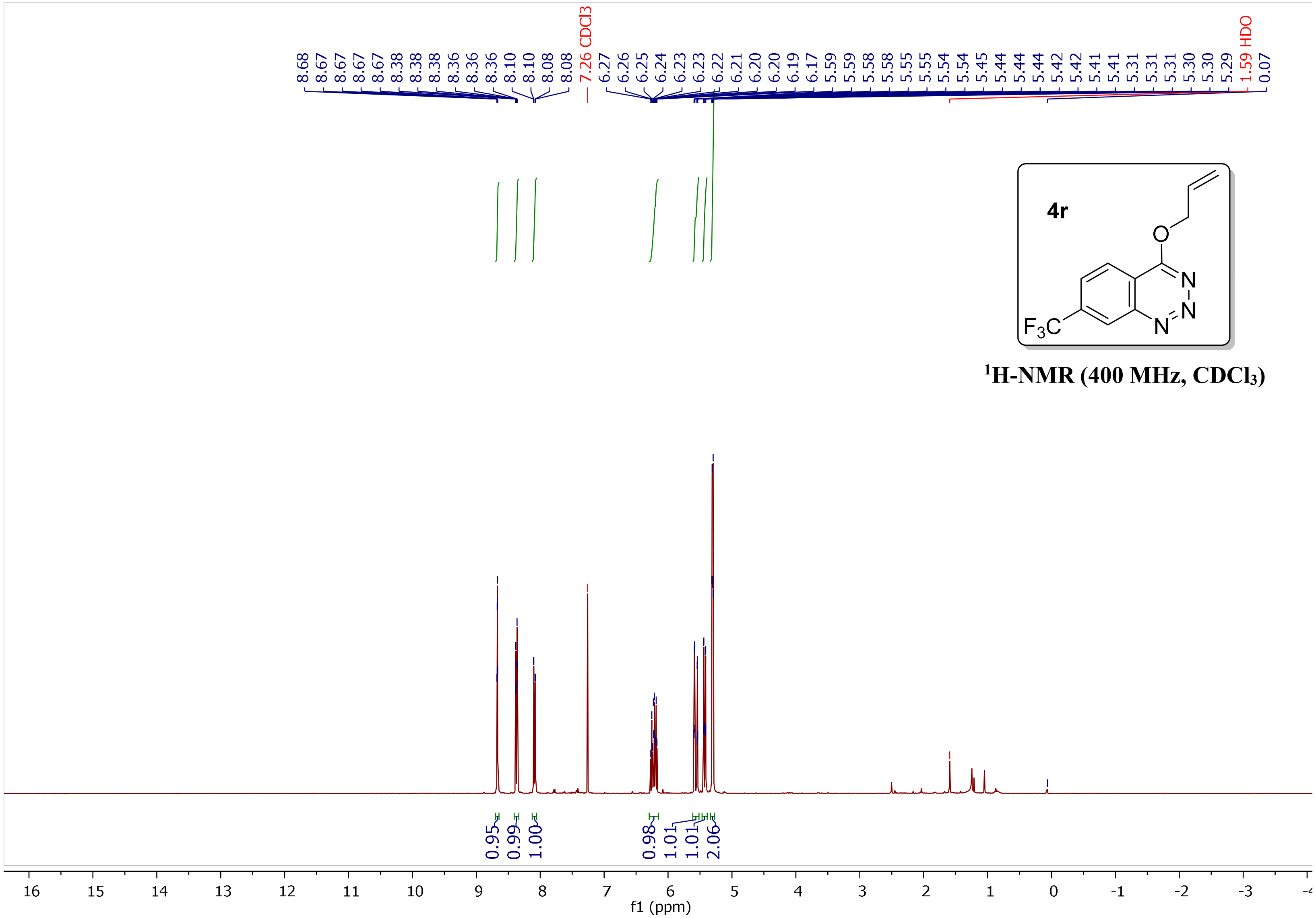

^{1}H-NMR (400 MHz, CDCl$_3$)

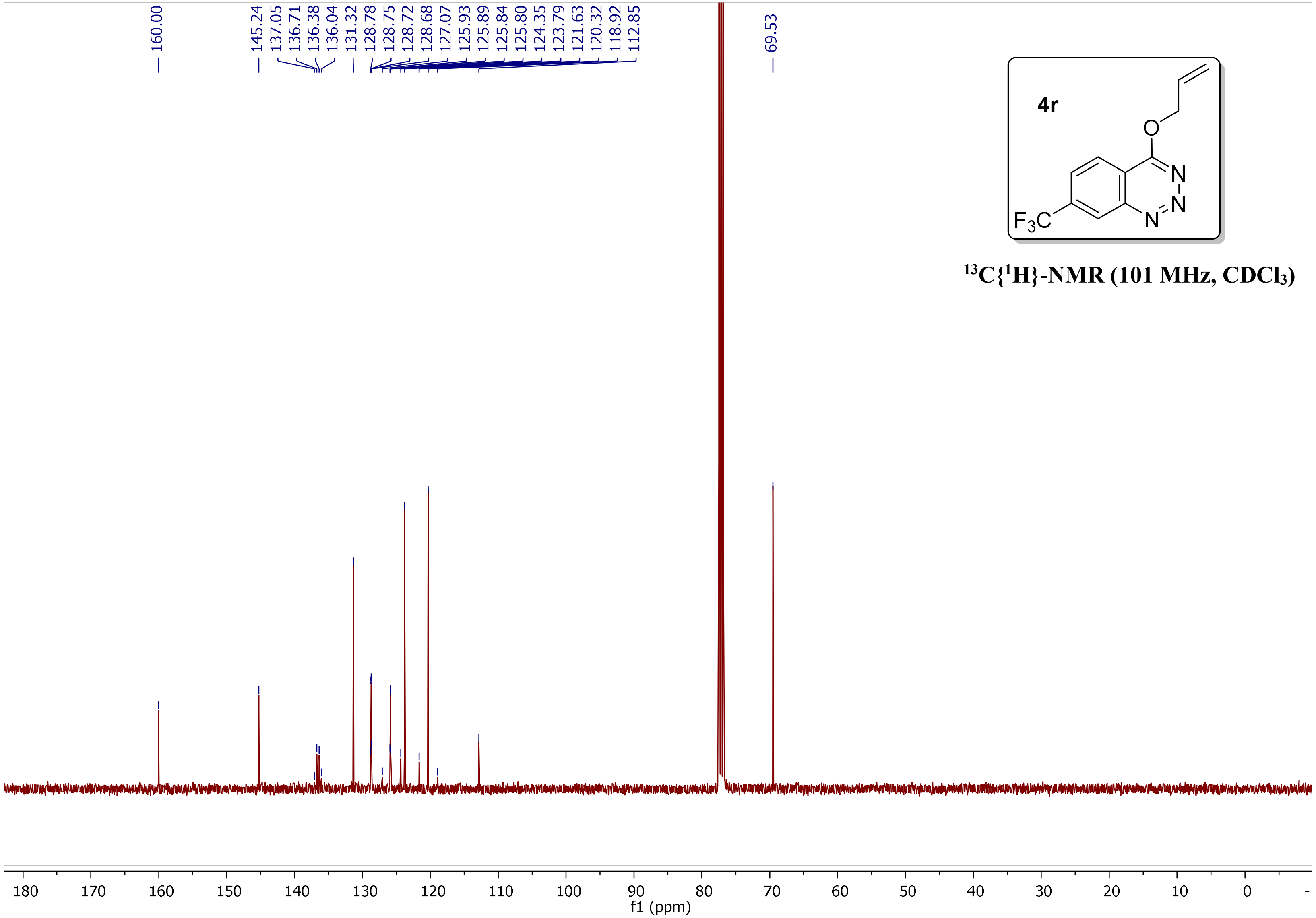

$^{13}C\{^{1}H\}$-NMR (101 MHz, $CDCl_3$)

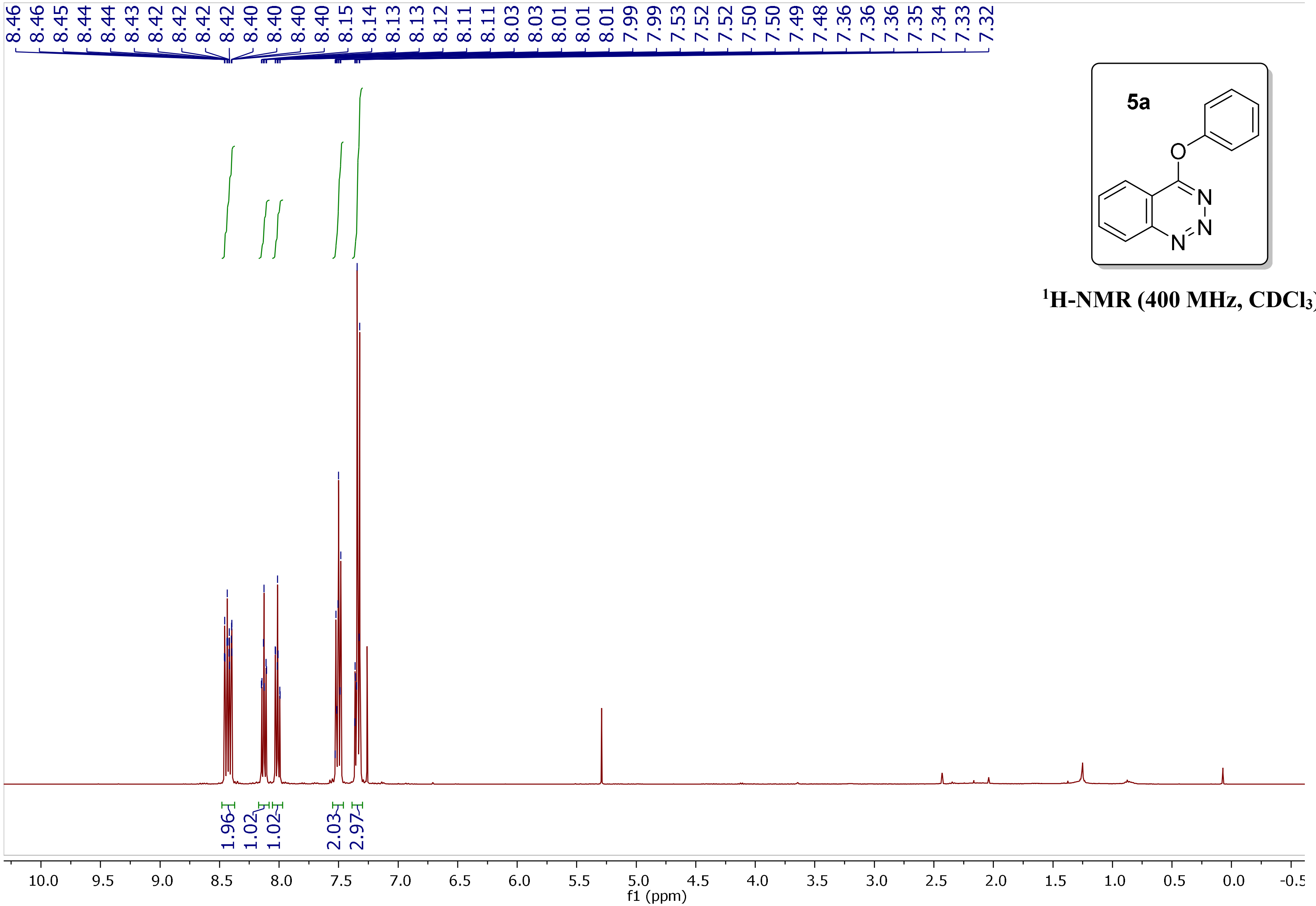

^{1}H-NMR (400 MHz, $CDCl_3$)

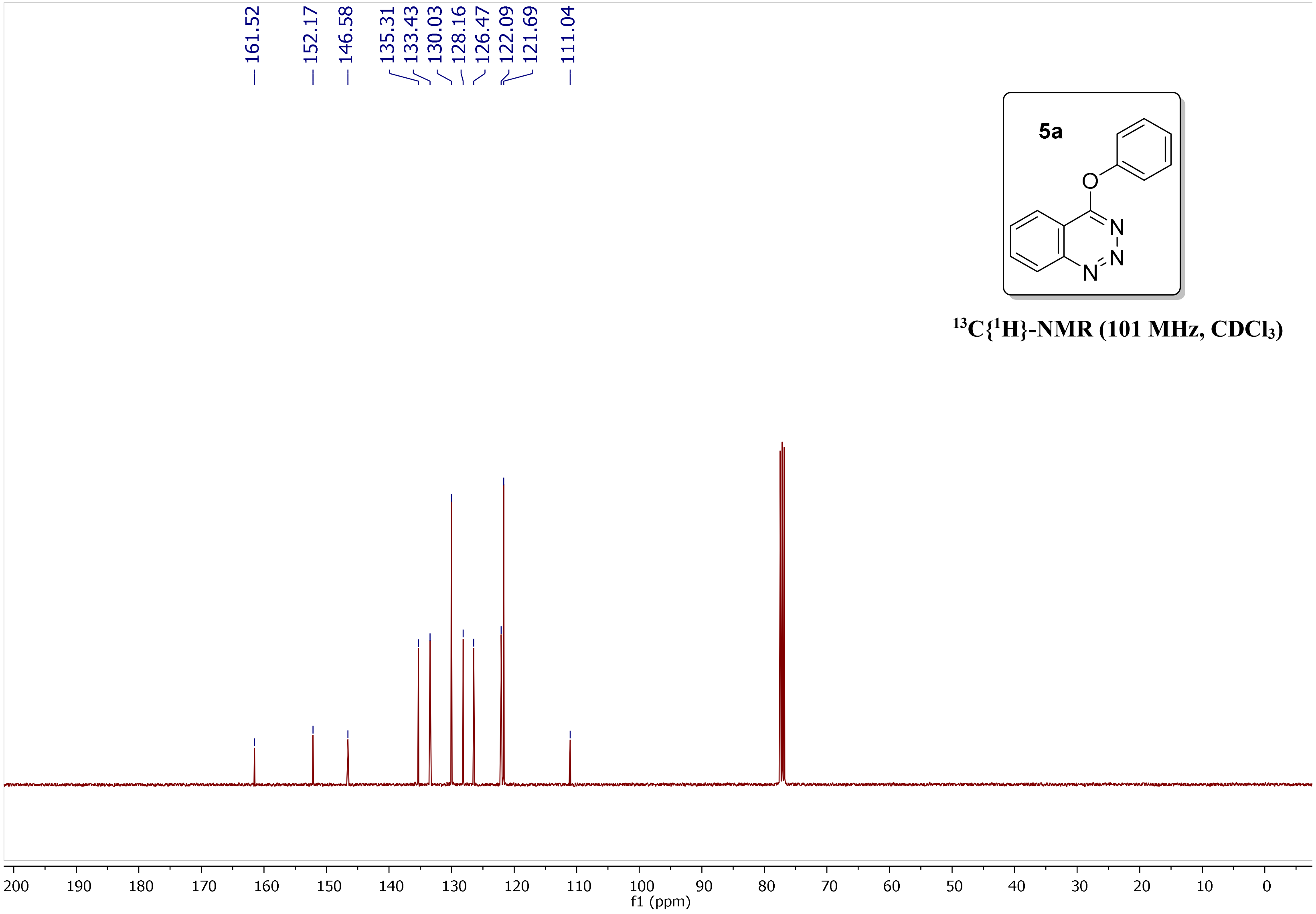

$^{13}C\{^{1}H\}$-NMR (101 MHz, $CDCl_3$)

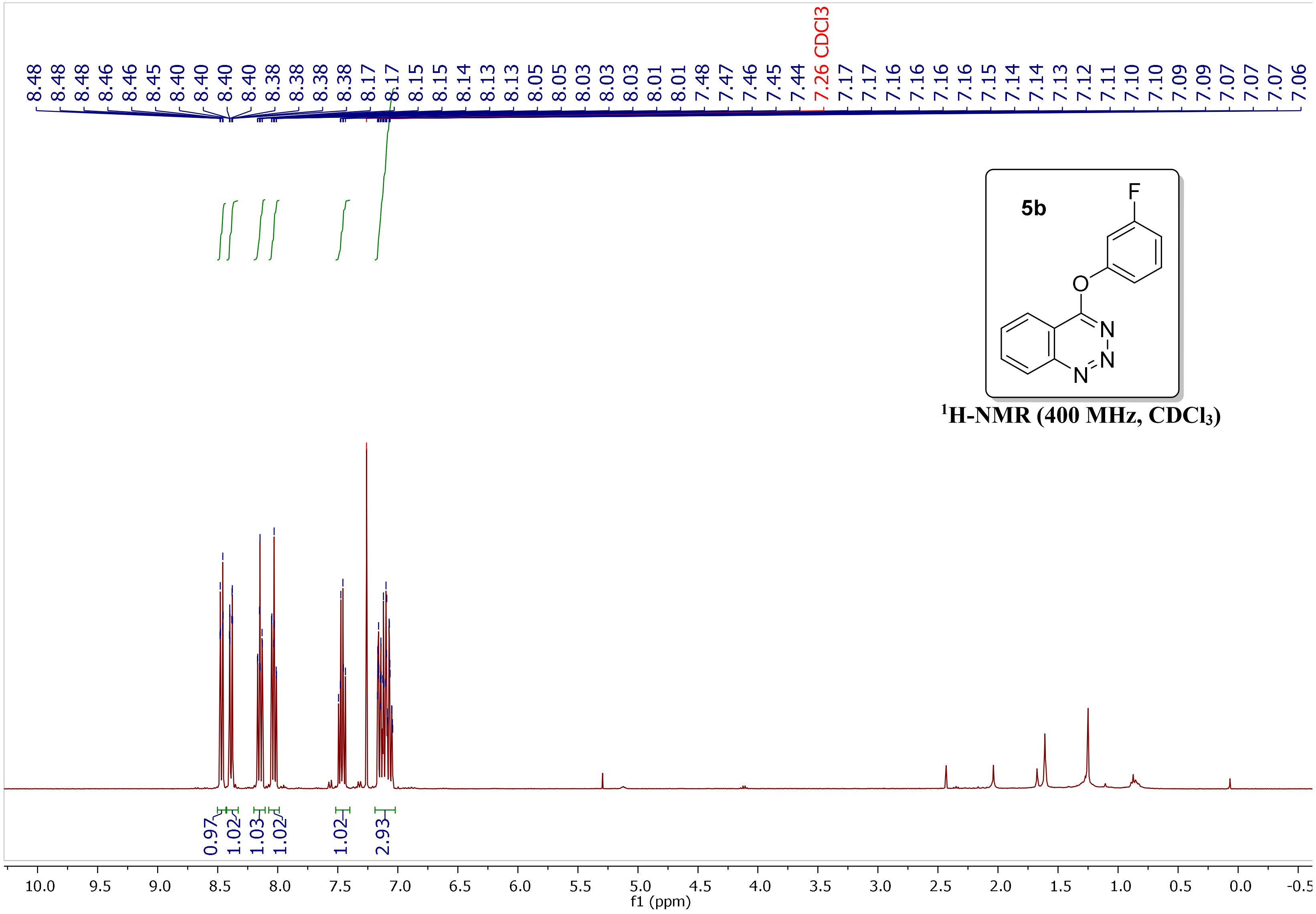
5b
F
O
N
N
N
¹H-NMR (400 MHz, CDCl₃)
7.26 CDCl3
f1 (ppm)

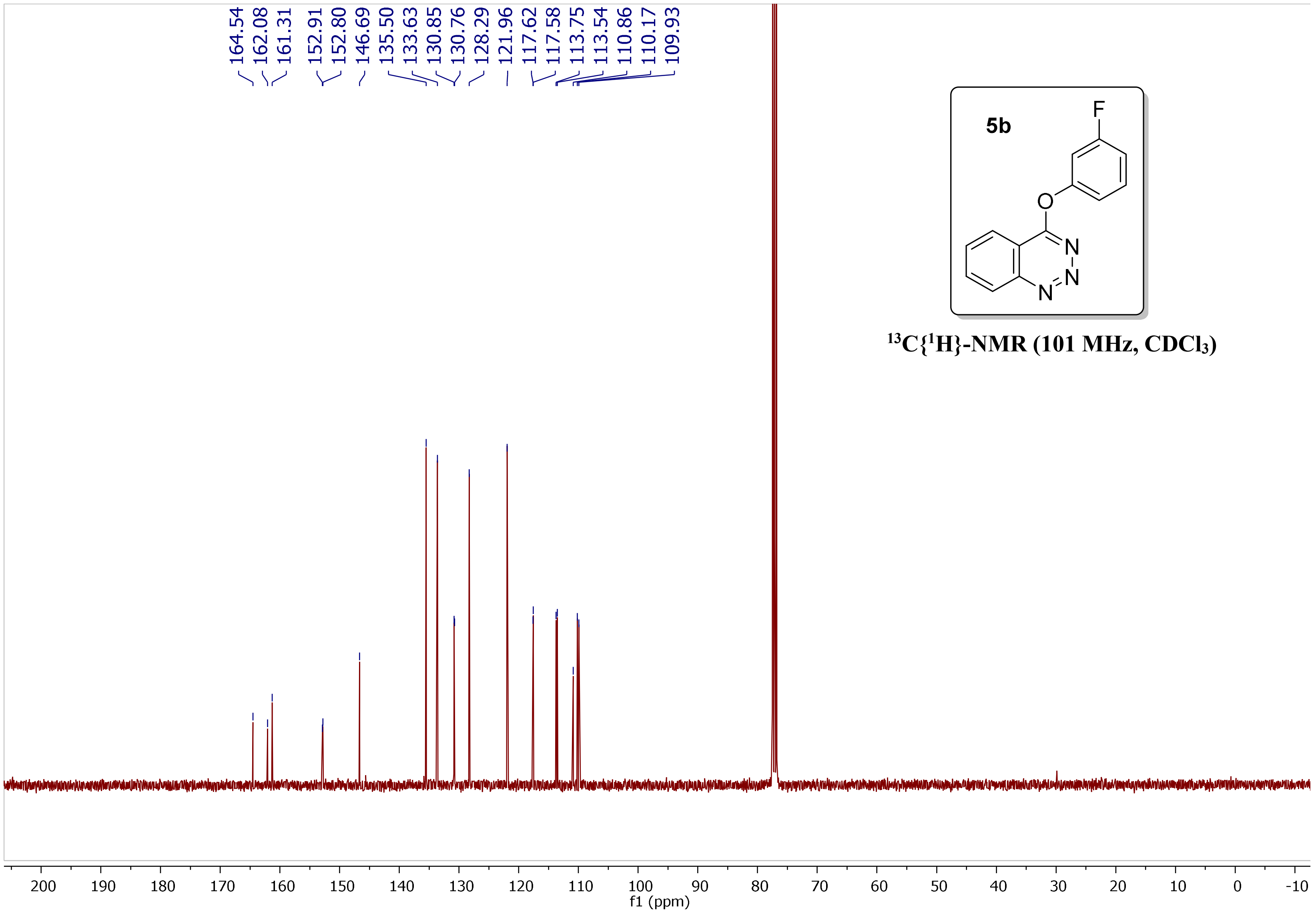
164.54
162.08
161.31
152.91
152.80
146.69
135.50
133.63
130.85
130.76
128.29
121.96
117.62
117.58
113.75
113.54
110.86
110.17
109.93
5b
F
O
N
N
N
13C{1H}-NMR (101 MHz, CDCl3)
200
190
180
170
160
150
140
130
120
110
100
90
80
70
60
50
40
30
20
10
0
-10
f1 (ppm)

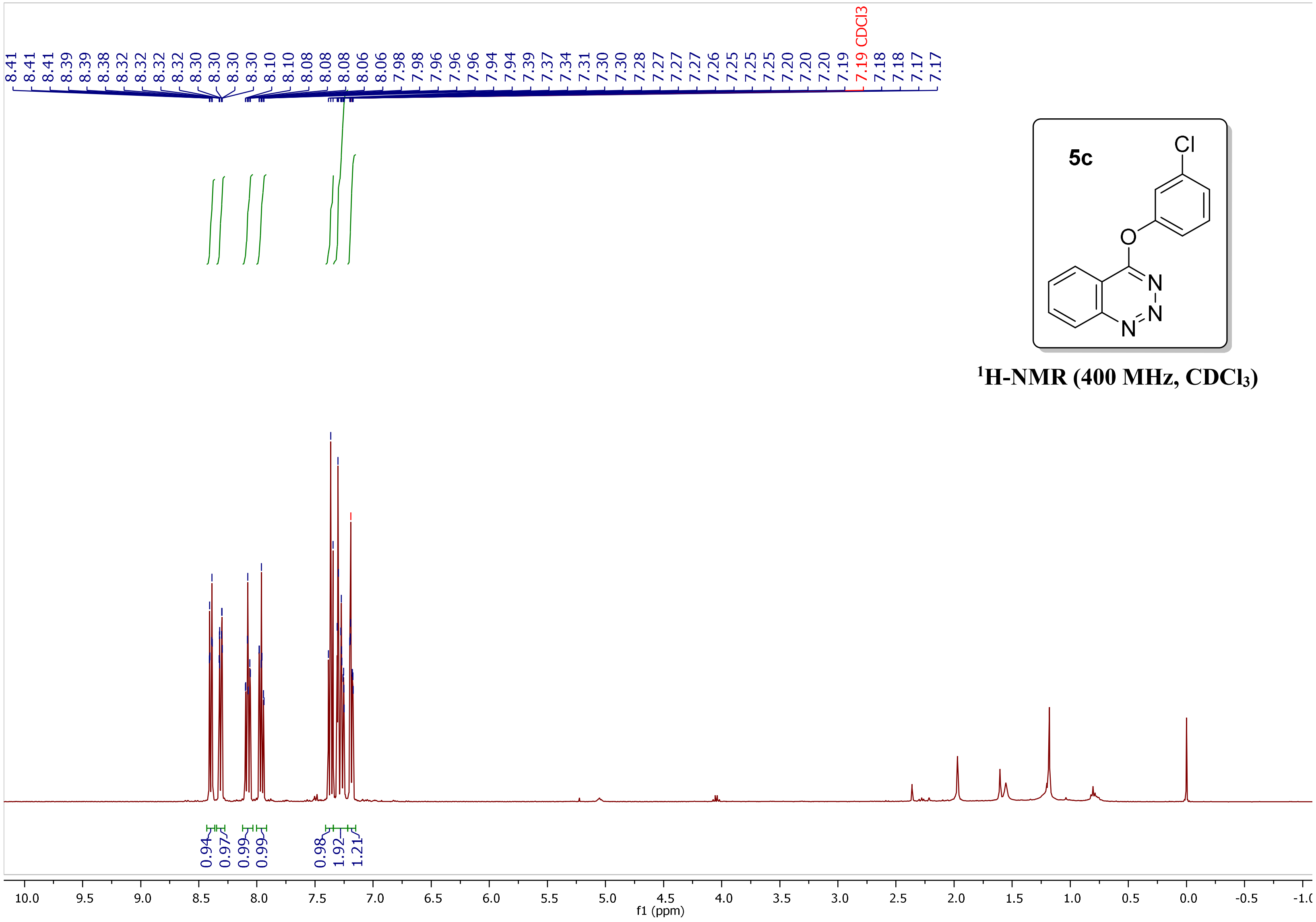

5c

^{1}H-NMR (400 MHz, $CDCl_3$)

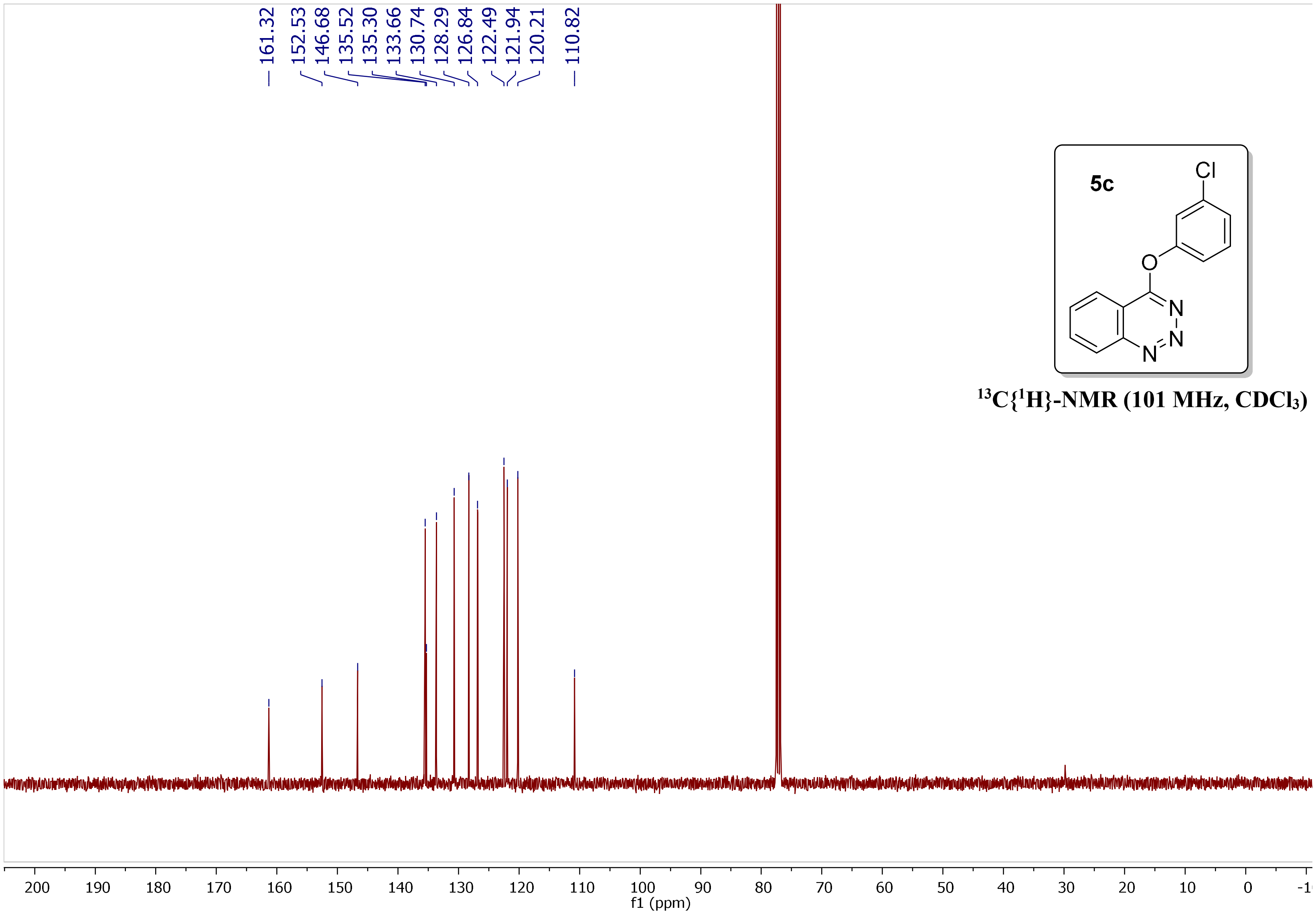

161.32
152.53
146.68
135.52
135.30
133.66
130.74
128.29
126.84
122.49
121.94
120.21
110.82
5c
Cl
O
N
N
N
$^{13}C\{^{1}H\}$-NMR (101 MHz, $CDCl_3$)
200
190
180
170
160
150
140
130
120
110
100
90
80
70
60
50
40
30
20
10
0
-1
f1 (ppm)

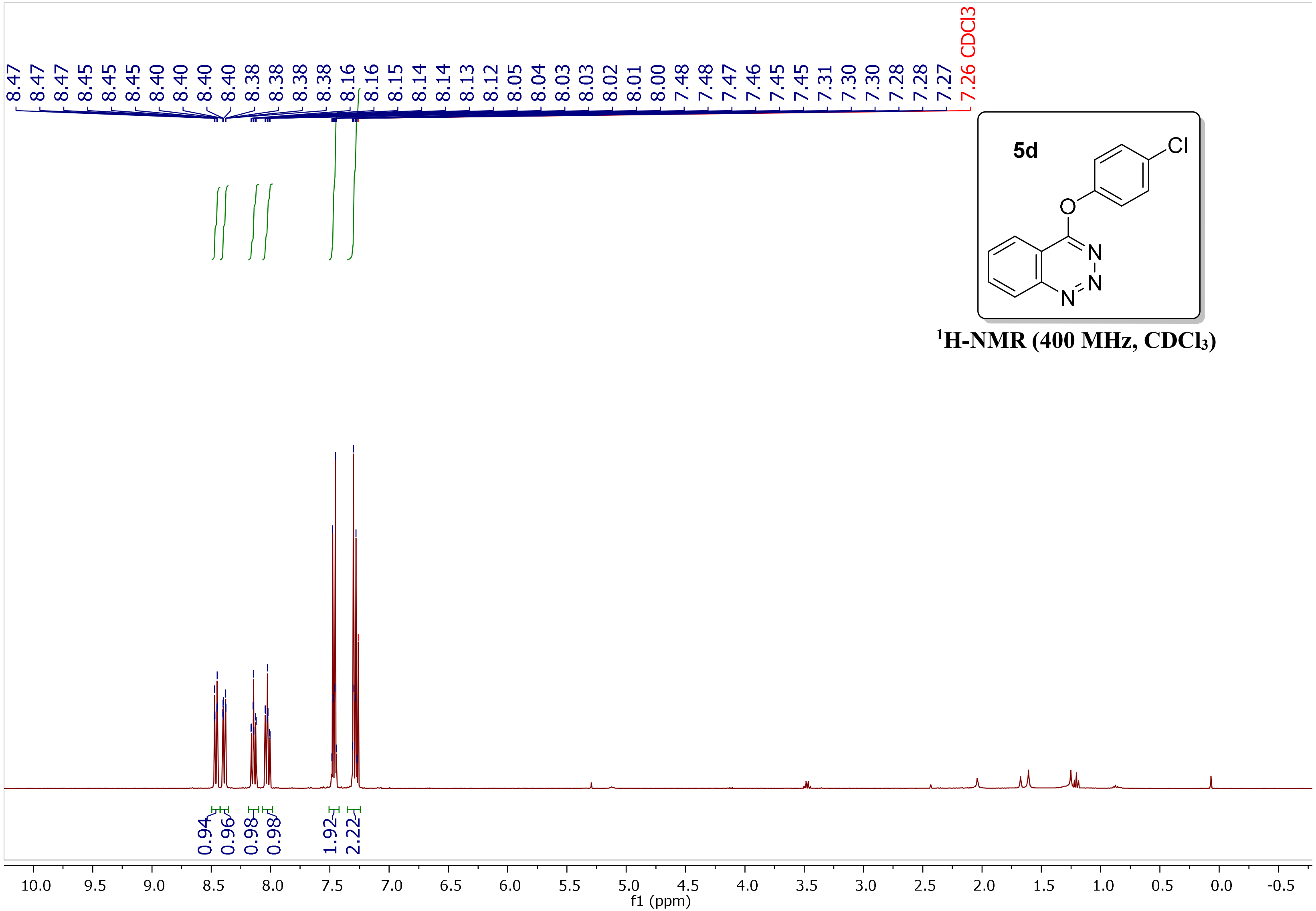

8.47
8.47
8.47
8.45
8.45
8.45
8.40
8.40
8.40
8.40
8.38
8.38
8.38
8.38
8.16
8.16
8.15
8.14
8.14
8.13
8.12
8.05
8.04
8.03
8.03
8.02
8.01
8.00
7.48
7.48
7.47
7.46
7.45
7.45
7.31
7.30
7.30
7.28
7.28
7.27
7.26 CDCl3
5d
Cl
O
N
N
N
1H-NMR (400 MHz, CDCl3)
0.94
0.96
0.98
0.98
1.92
2.22
10.0
9.5
9.0
8.5
8.0
7.5
7.0
6.5
6.0
5.5
5.0
4.5
4.0
3.5
3.0
2.5
2.0
1.5
1.0
0.5
0.0
-0.5
f1 (ppm)

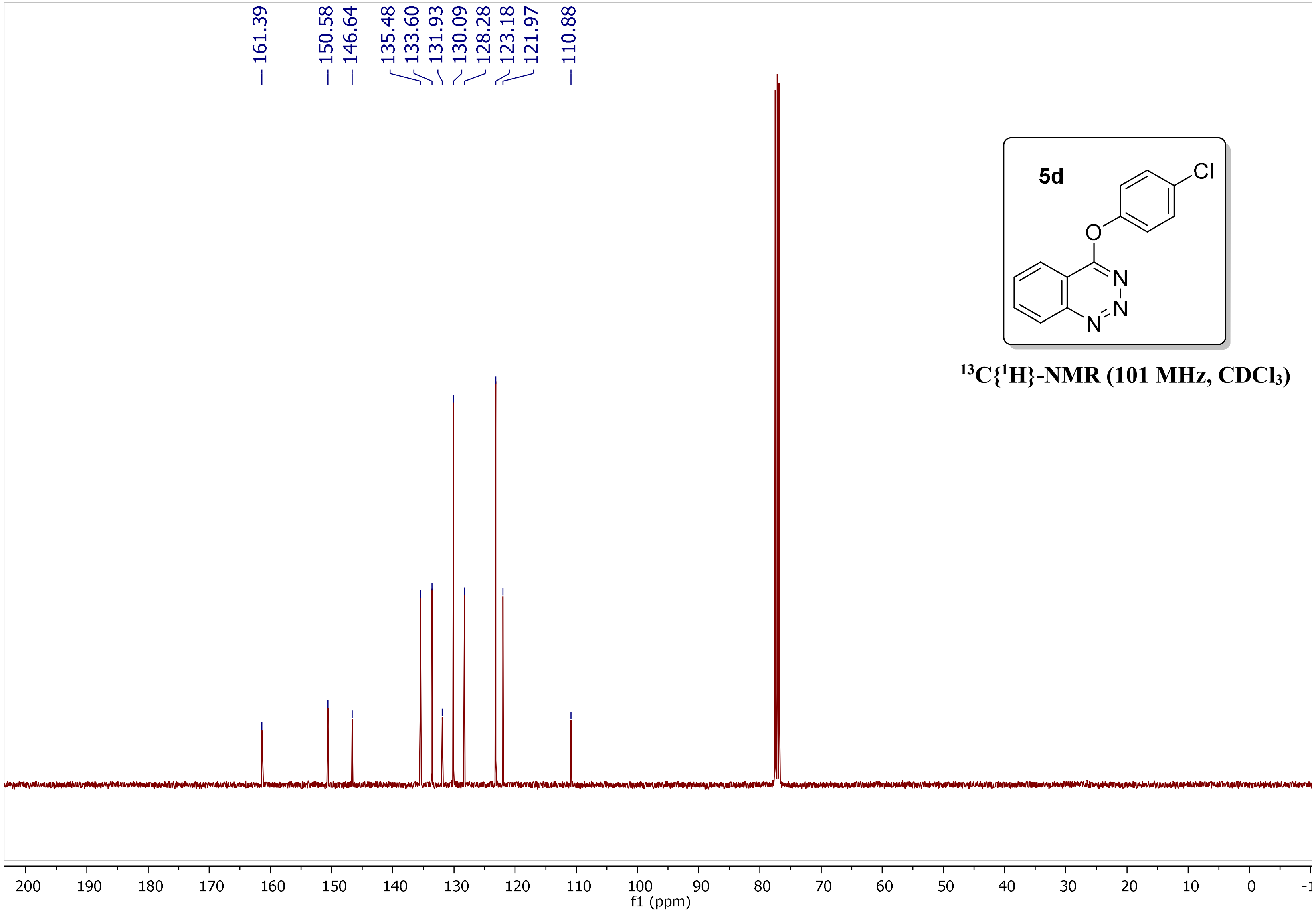

$^{13}C\{^{1}H\}$-NMR (101 MHz, $CDCl_3$)

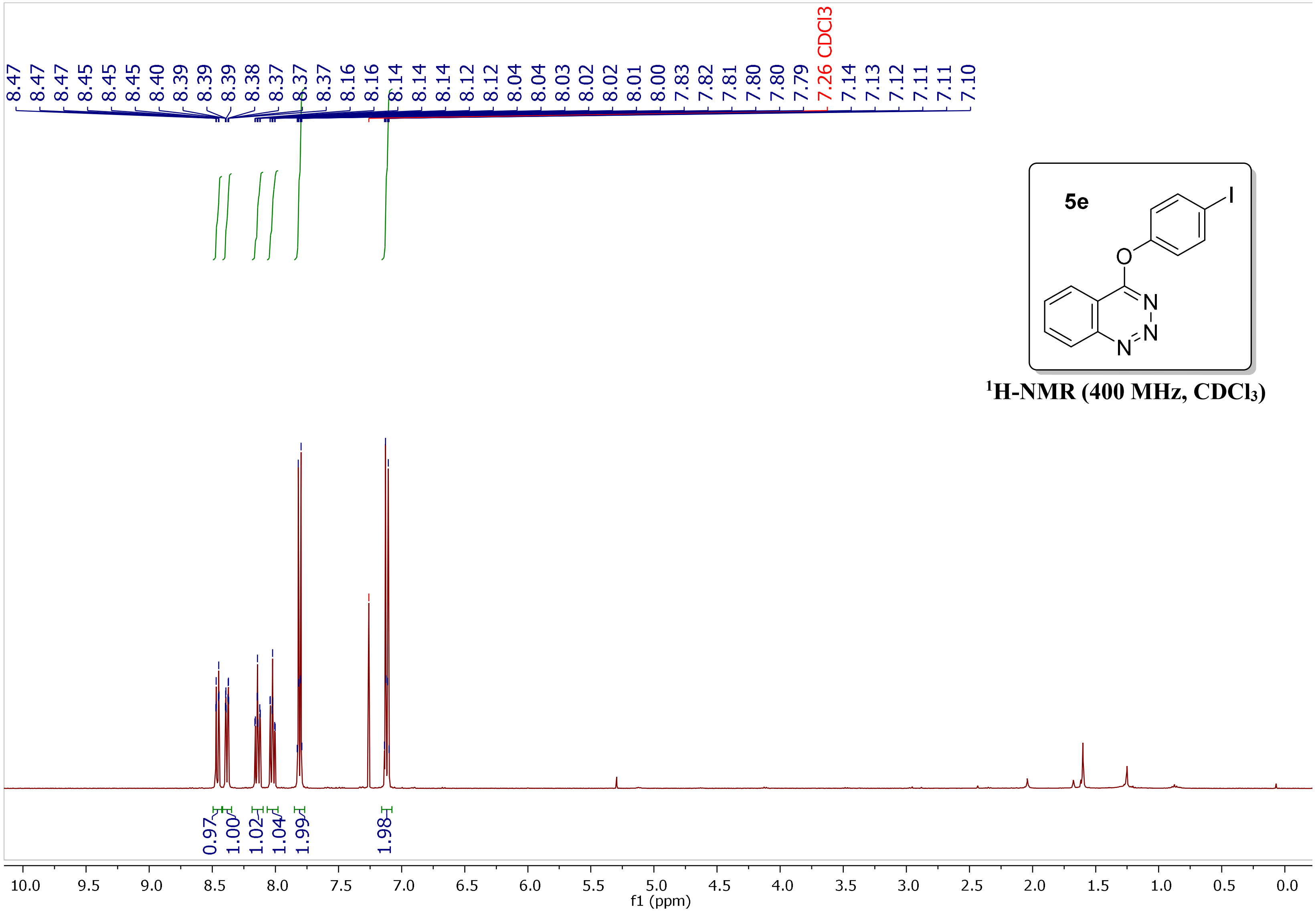

^{1}H-NMR (400 MHz, $CDCl_3$)

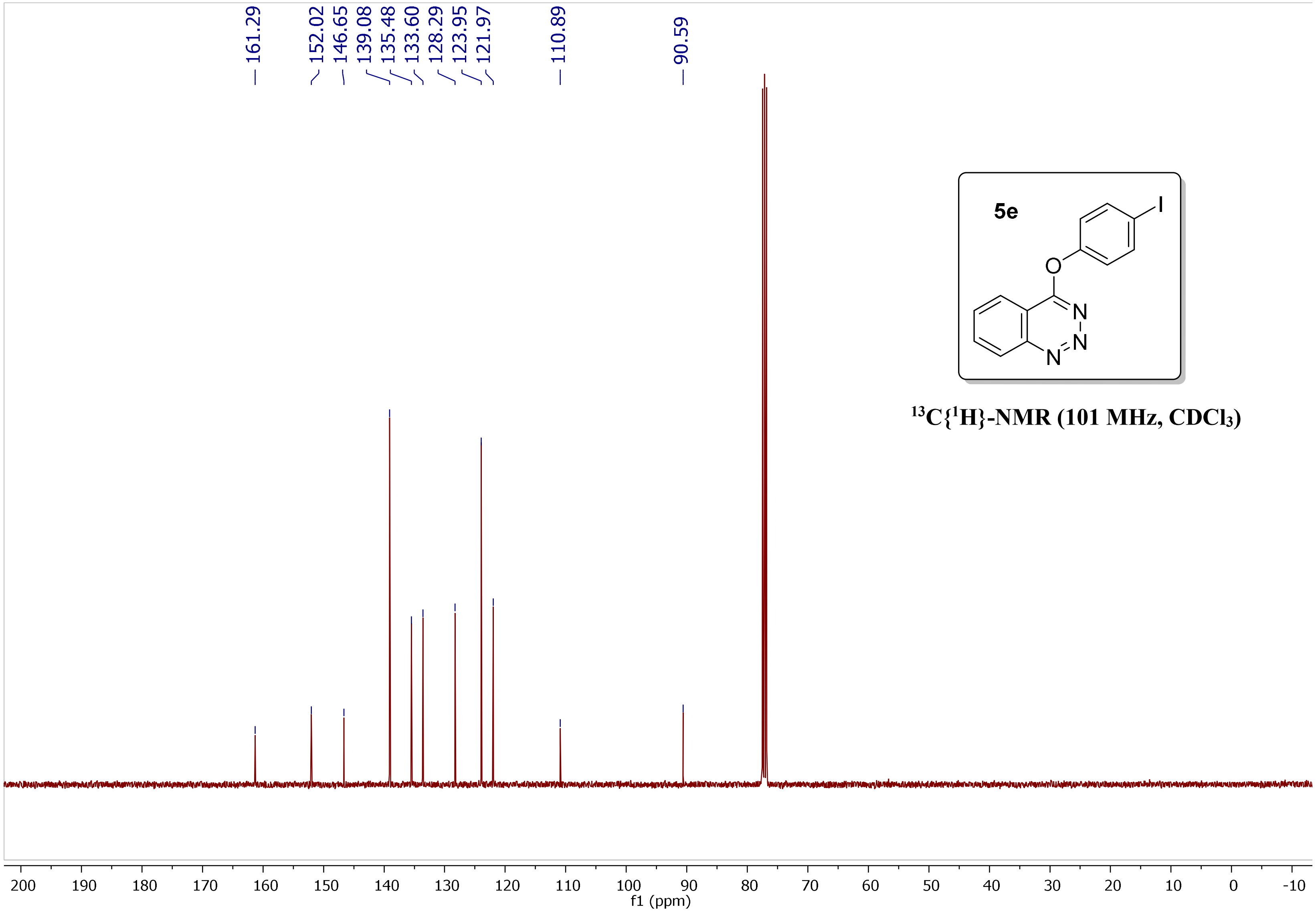

$^{13}C\{^1H\}$-NMR (101 MHz, $CDCl_3$)

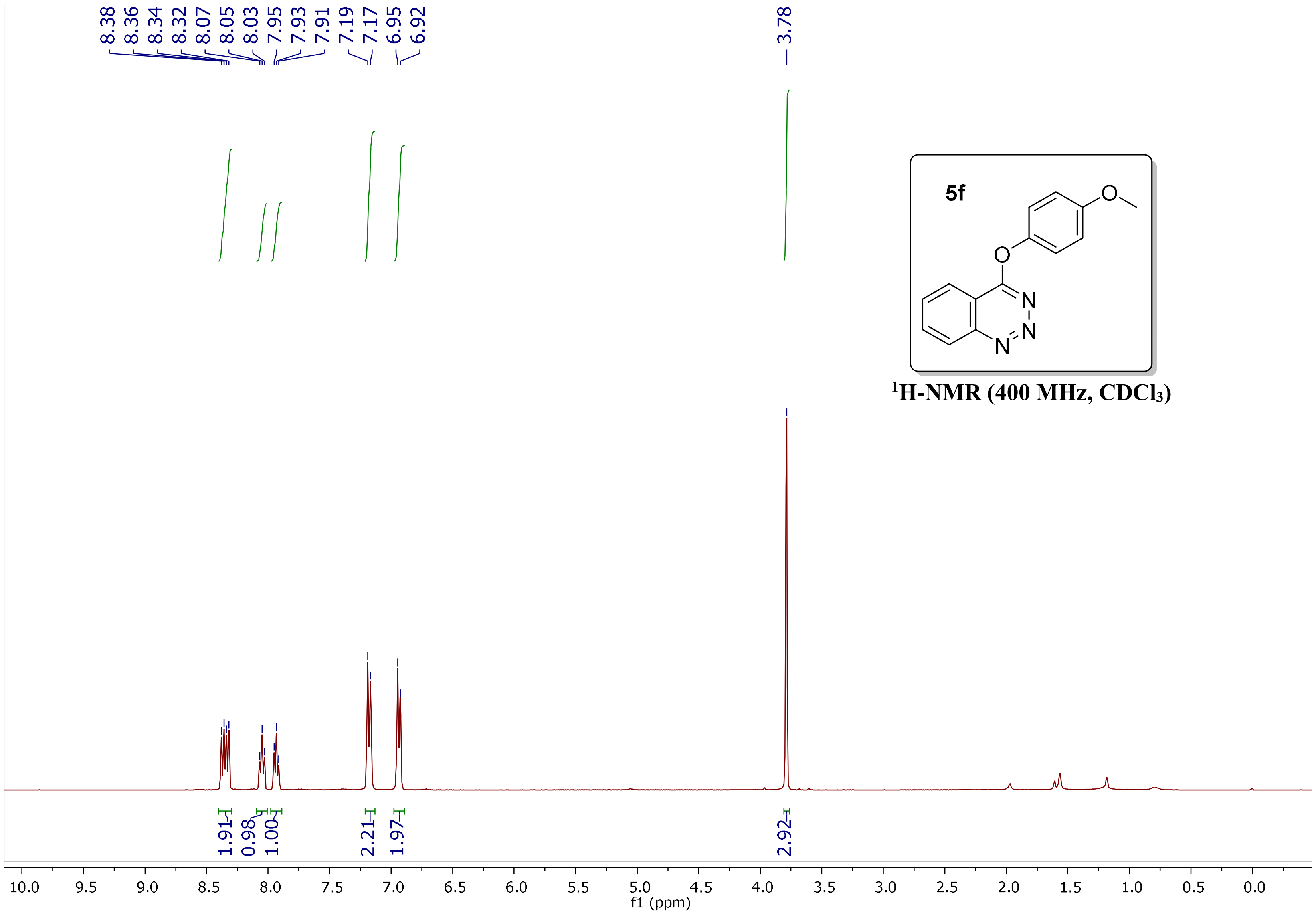
8.38
8.36
8.34
8.32
8.07
8.05
8.03
7.95
7.93
7.91
7.19
7.17
6.95
6.92
3.78
5f
O
O
N
N
N
^{1}H-NMR (400 MHz, CDCl$_3$)
1.91
0.98
1.00
2.21
1.97
2.92
10.0
9.5
9.0
8.5
8.0
7.5
7.0
6.5
6.0
5.5
5.0
4.5
4.0
3.5
3.0
2.5
2.0
1.5
1.0
0.5
0.0
f1 (ppm)

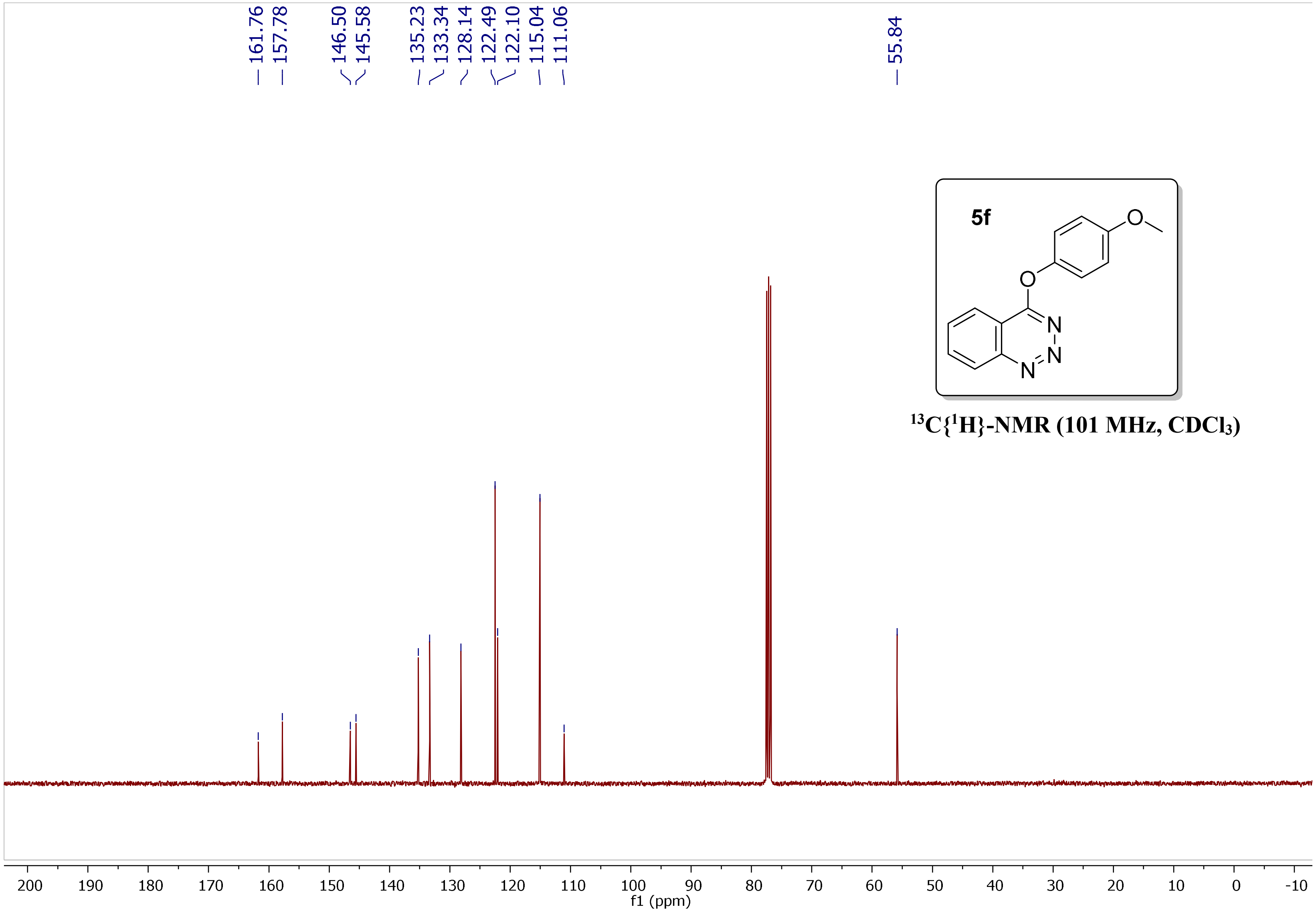

$^{13}C\{^{1}H\}$-NMR (101 MHz, $CDCl_3$)

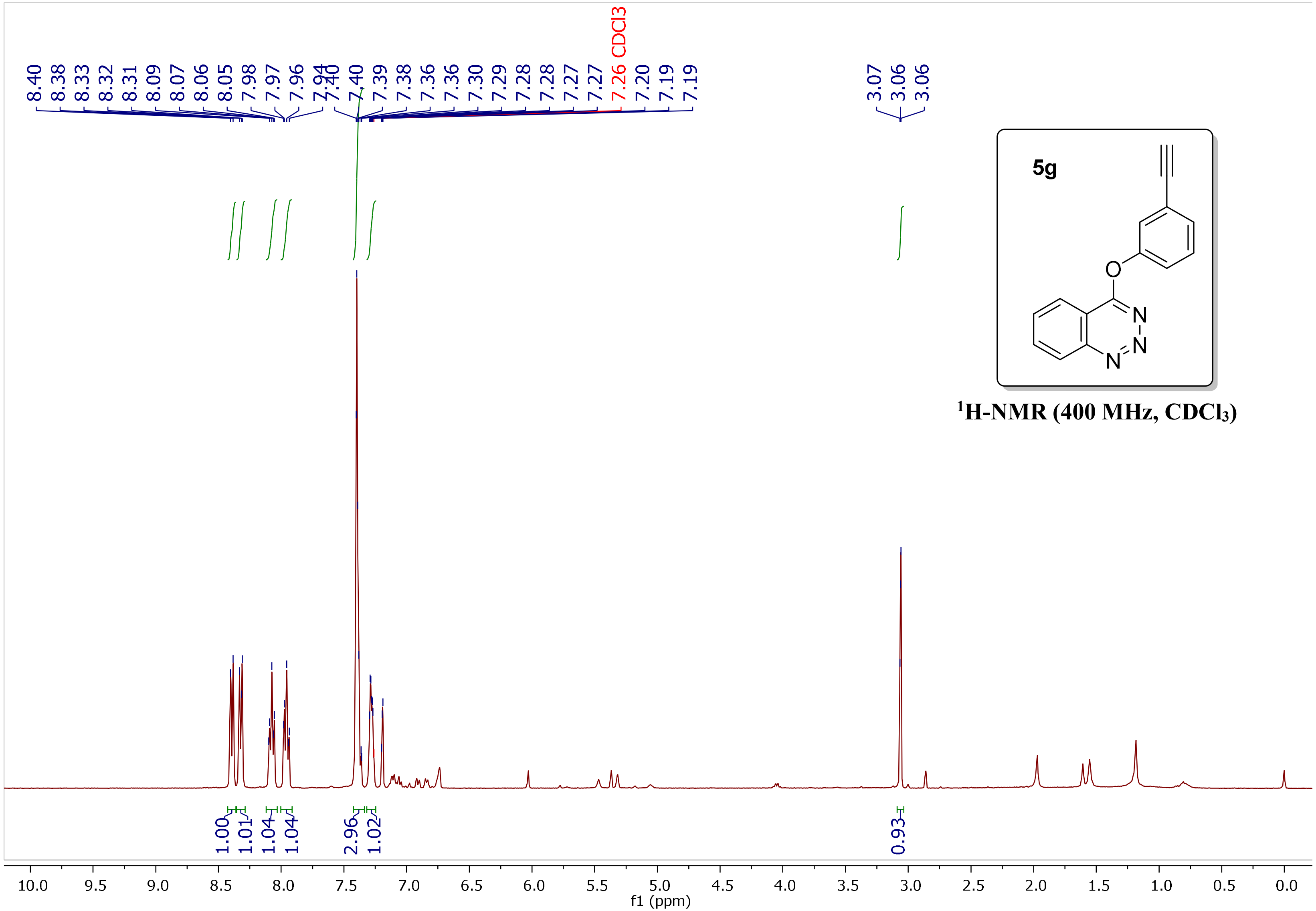

^{1}H-NMR (400 MHz, $CDCl_3$)

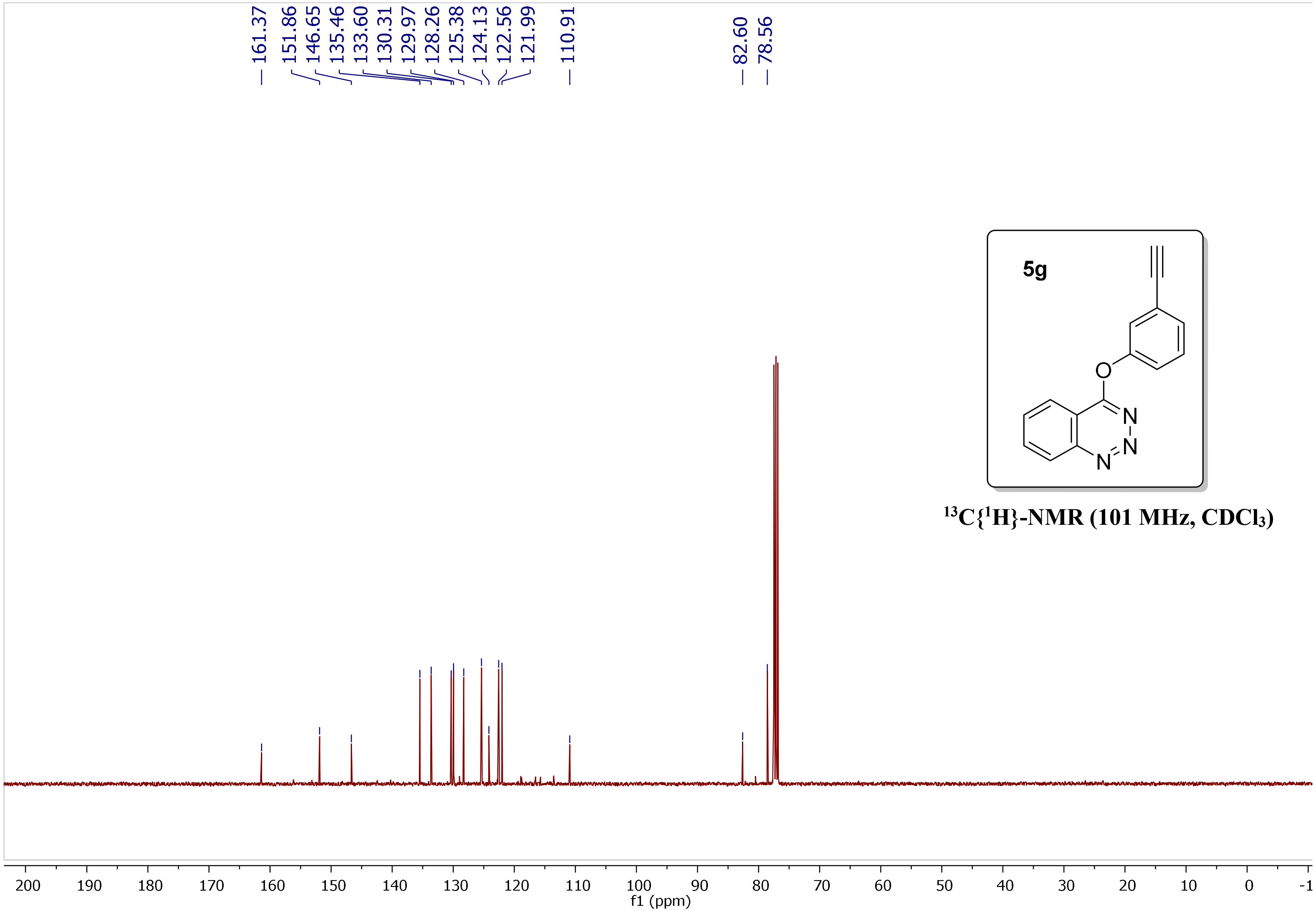
161.37
151.86
146.65
135.46
133.60
130.31
129.97
128.26
125.38
124.13
122.56
121.99
110.91
82.60
78.56
5g
O
N
N
N
$^{13}C\{^{1}H\}$-NMR (101 MHz, $CDCl_3$)
200 190 180 170 160 150 140 130 120 110 100 90 80 70 60 50 40 30 20 10 0 -1
f1 (ppm)

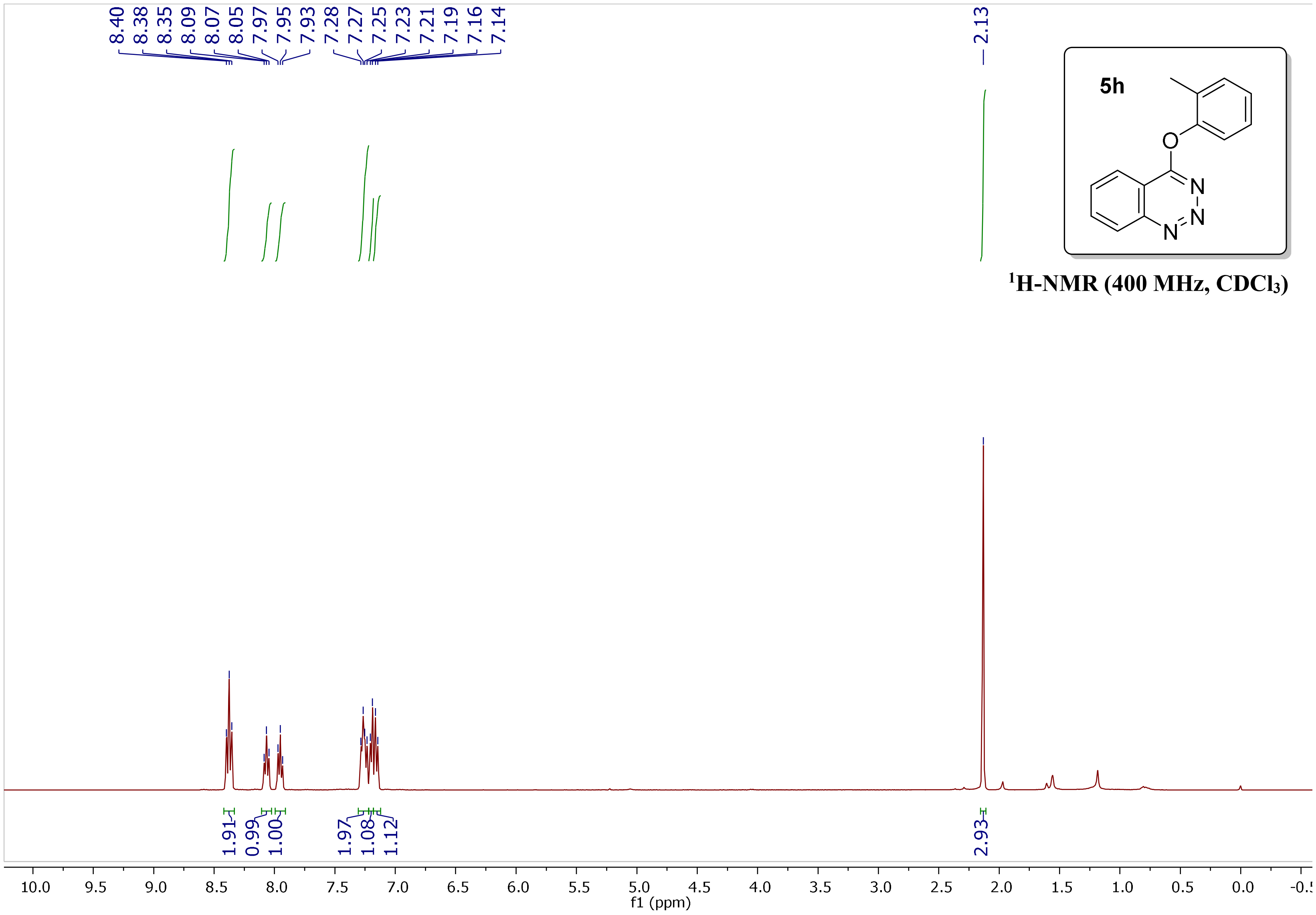

8.40
8.38
8.35
8.09
8.07
8.05
7.97
7.95
7.93
7.28
7.27
7.25
7.23
7.21
7.19
7.16
7.14
2.13
5h
N
N
N
O
^{1}H-NMR (400 MHz, CDCl$_3$)
1.91
0.99
1.00
1.97
1.08
1.12
2.93
f1 (ppm)

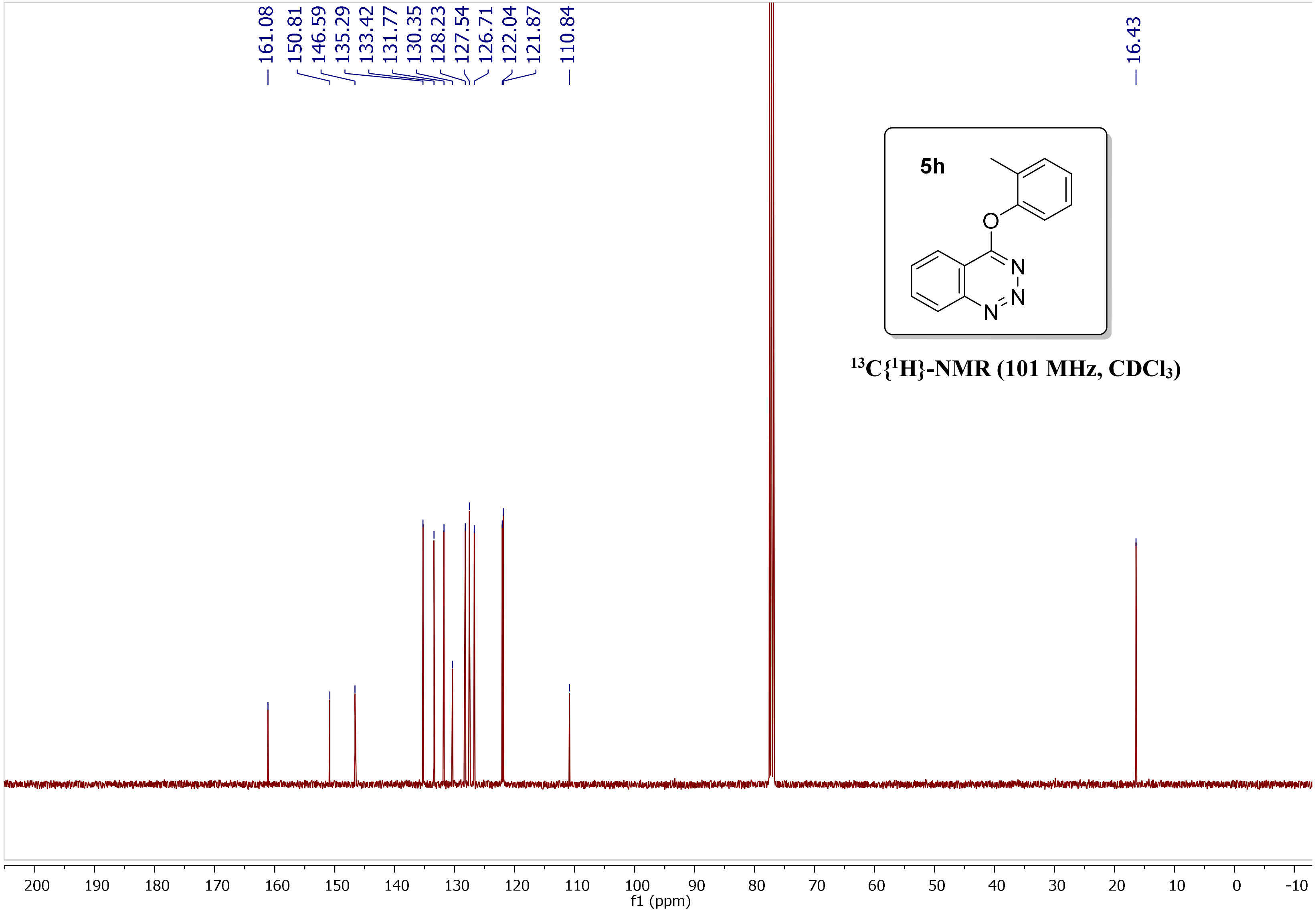
161.08
150.81
146.59
135.29
133.42
131.77
130.35
128.23
127.54
126.71
122.04
121.87
110.84
16.43
5h
O
N
N
N
$^{13}C\{^{1}H\}$-NMR (101 MHz, $CDCl_3$)
200 190 180 170 160 150 140 130 120 110 100 90 80 70 60 50 40 30 20 10 0 -10
f1 (ppm)

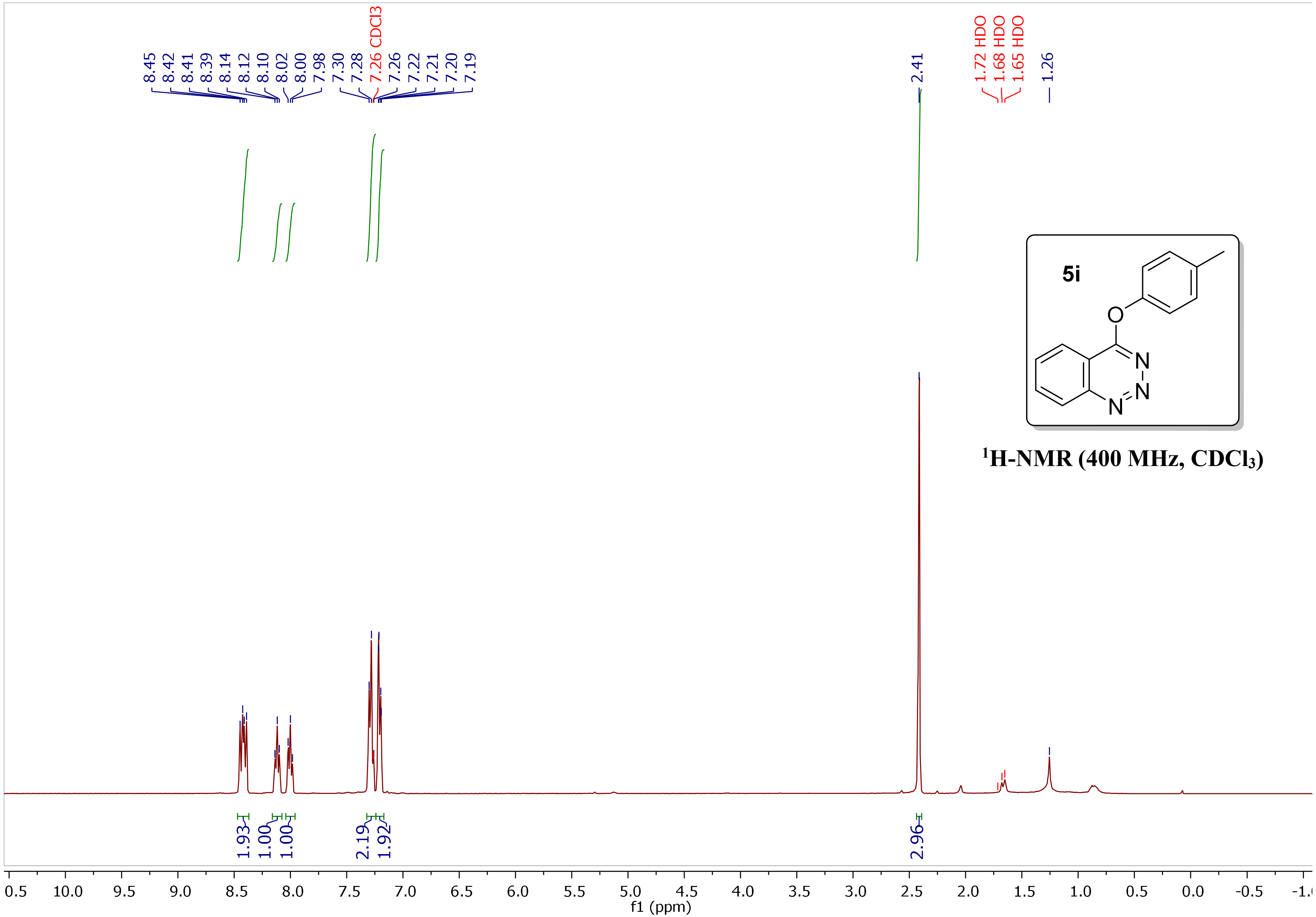

^{1}H-NMR (400 MHz, CDCl$_3$)

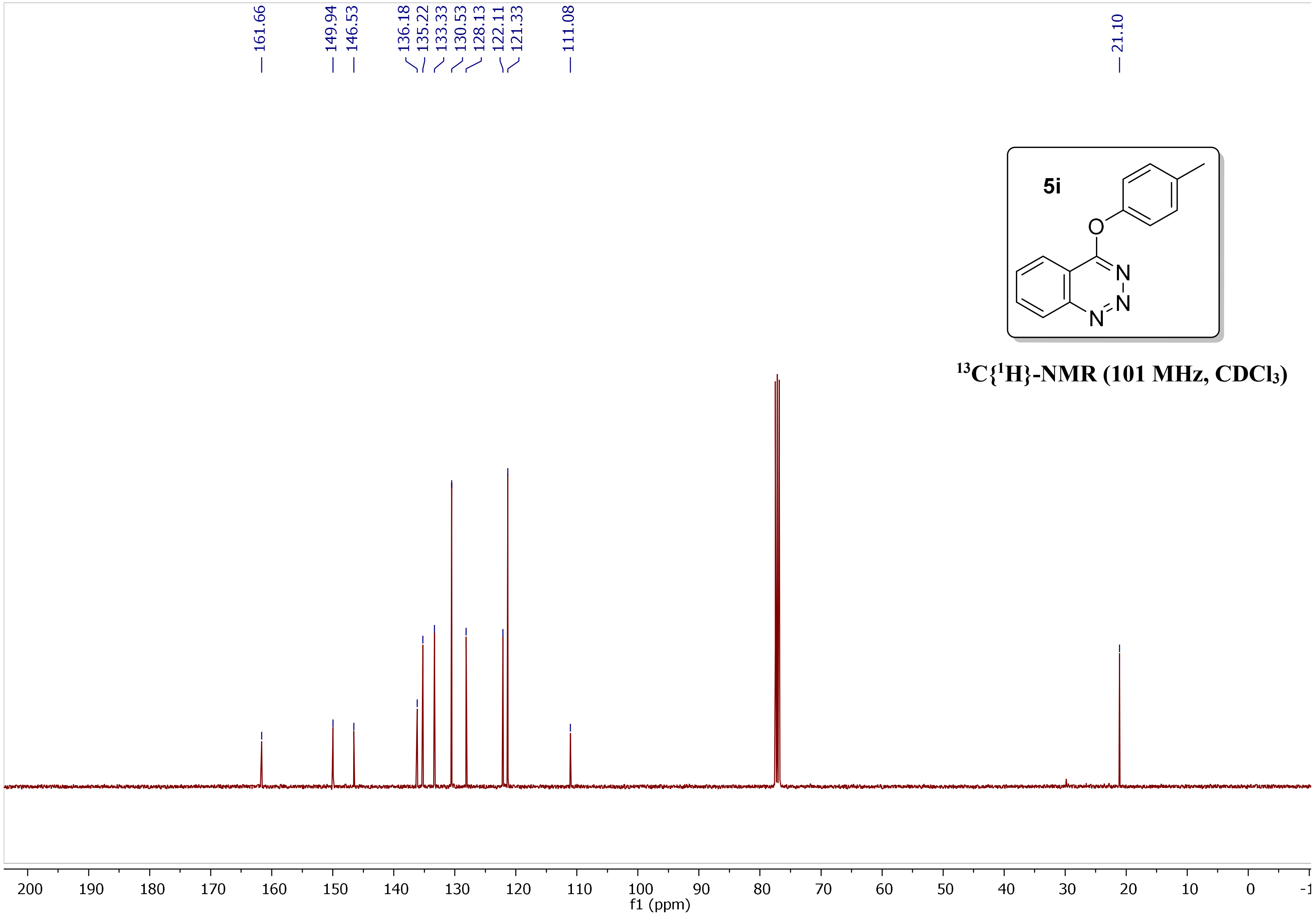
161.66
149.94
146.53
136.18
135.22
133.33
130.53
128.13
122.11
121.33
111.08
21.10
5i
O
N
N
N
13C{1H}-NMR (101 MHz, CDCl3)
200 190 180 170 160 150 140 130 120 110 100 90 80 70 60 50 40 30 20 10 0
f1 (ppm)

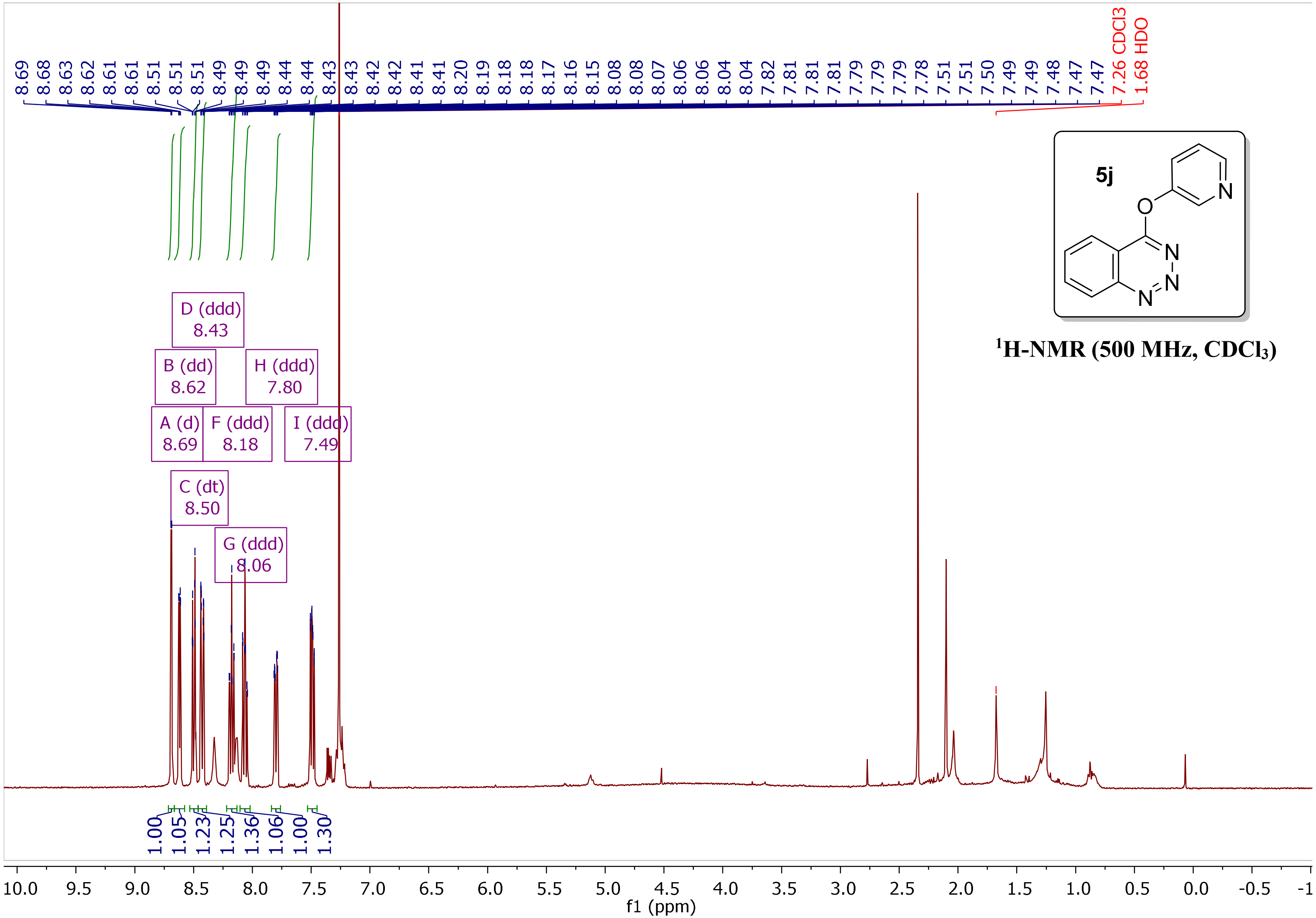
5j
N
O
N
N
N
¹H-NMR (500 MHz, CDCl3)
7.26 CDCl3
1.68 HDO
A (d) 8.69
B (dd) 8.62
C (dt) 8.50
D (ddd) 8.43
F (ddd) 8.18
G (ddd) 8.06
H (ddd) 7.80
I (ddd) 7.49
1.00
1.05
1.23
1.25
1.36
1.06
1.00
1.30
f1 (ppm)

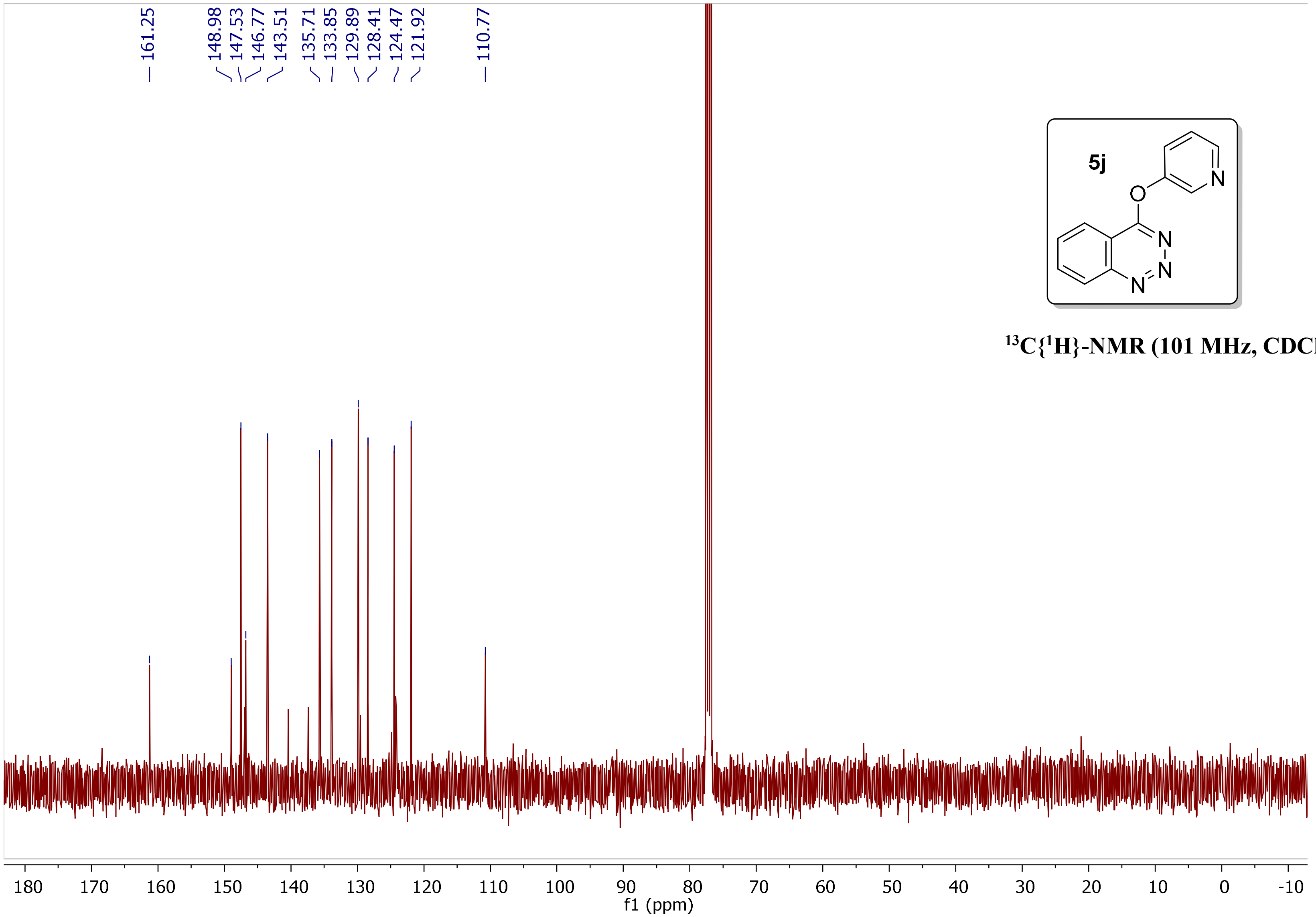

$^{13}C\{^{1}H\}$-NMR (101 MHz, $CDCl_3$)

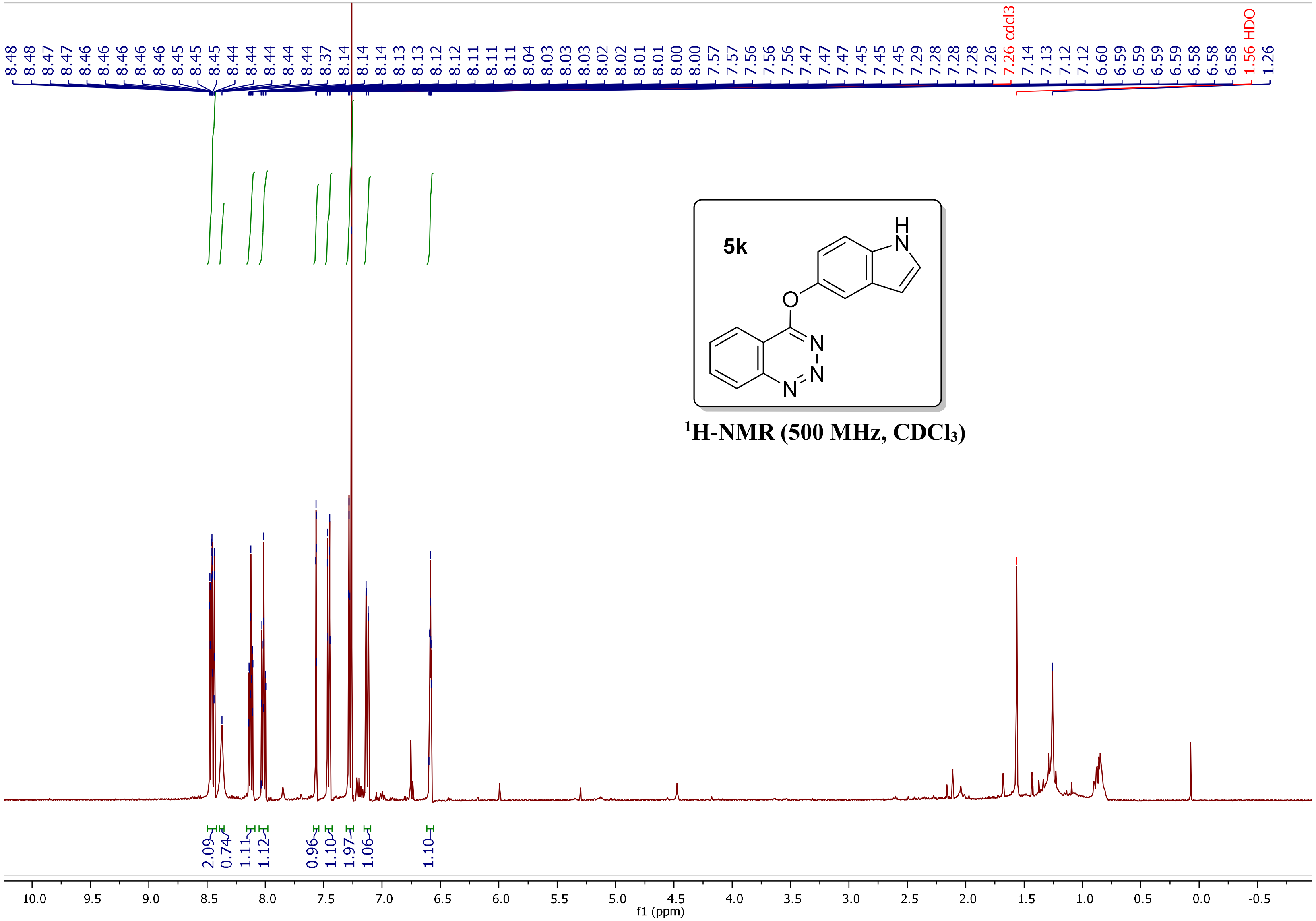

^{1}H-NMR (500 MHz, $CDCl_3$)

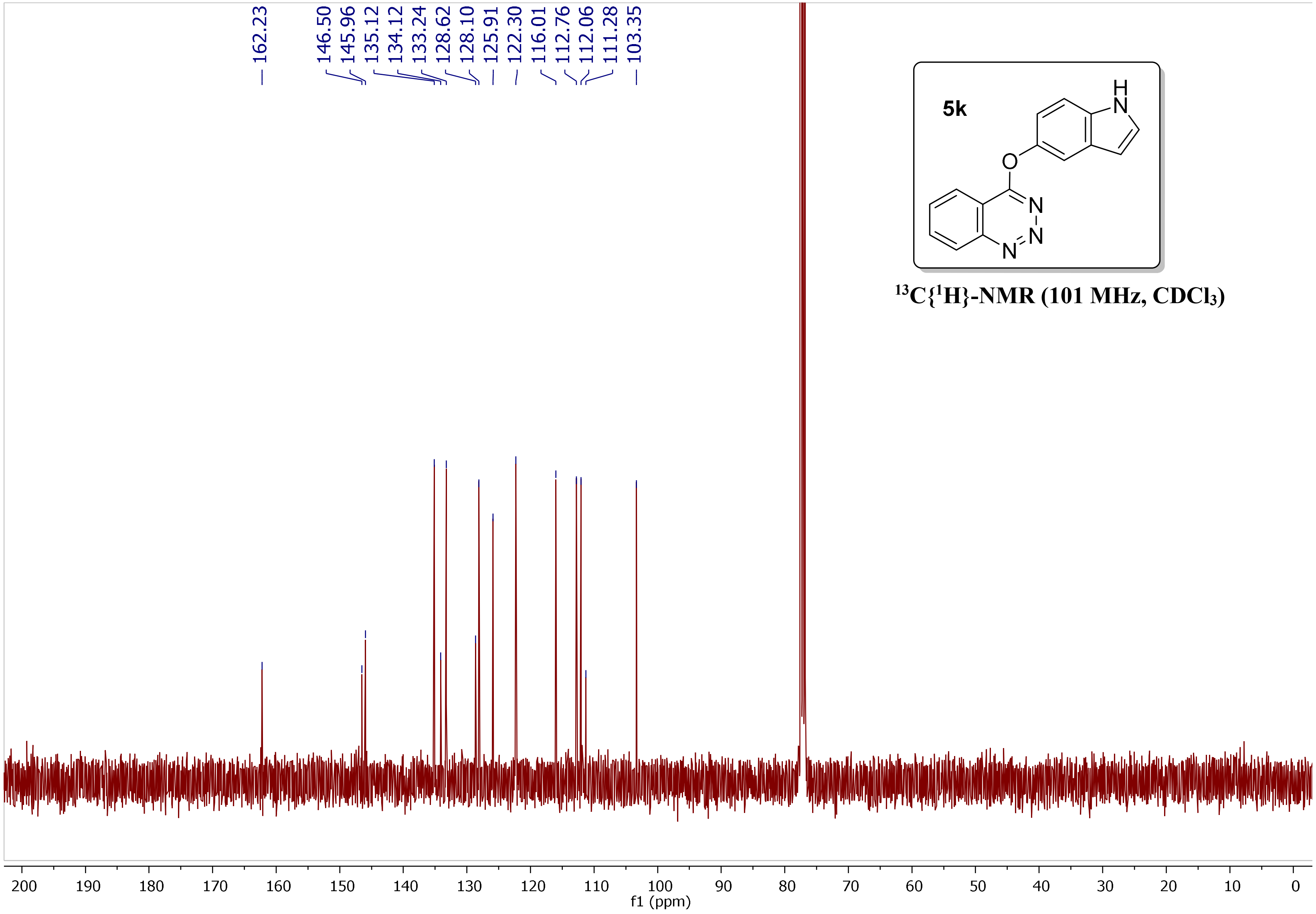
162.23
146.50
145.96
135.12
134.12
133.24
128.62
128.10
125.91
122.30
116.01
112.76
112.06
111.28
103.35
5k
H
N
O
N
N
N
$^{13}C\{^{1}H\}$-NMR (101 MHz, $CDCl_3$)
200 190 180 170 160 150 140 130 120 110 100 90 80 70 60 50 40 30 20 10 0
f1 (ppm)

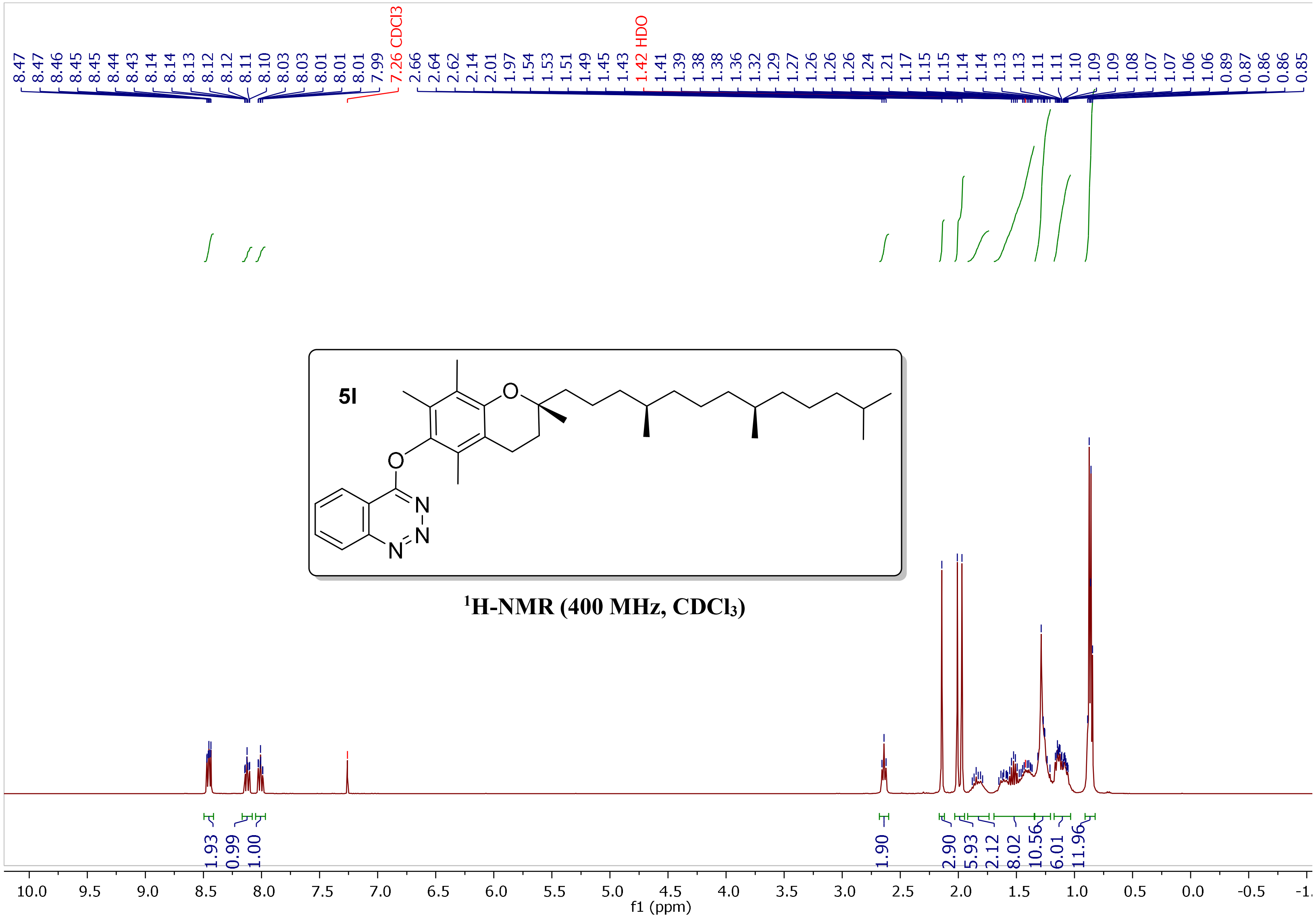

^{1}H-NMR (400 MHz, CDCl$_3$)

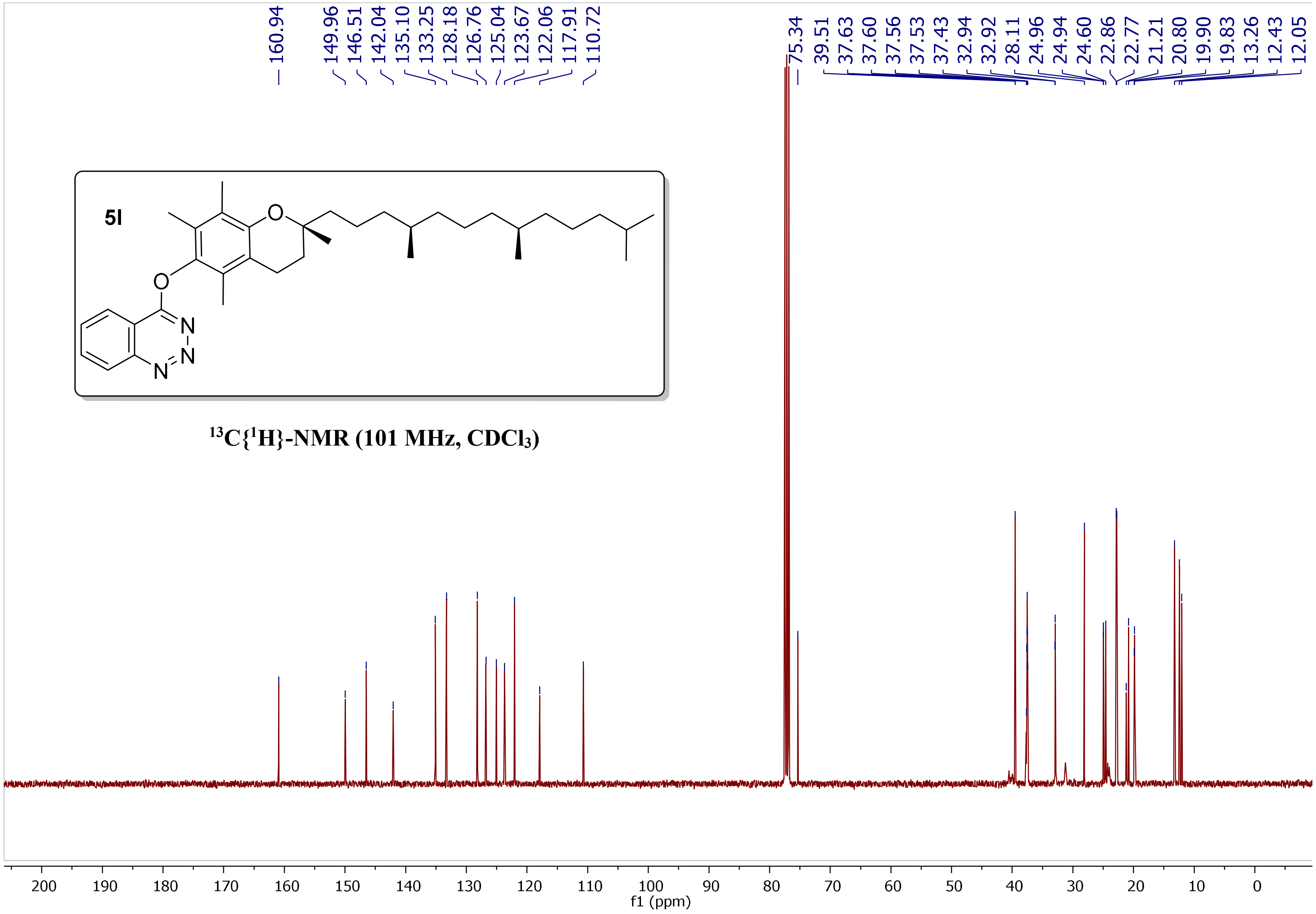

160.94
149.96
146.51
142.04
135.10
133.25
128.18
126.76
125.04
123.67
122.06
117.91
110.72
75.34
39.51
37.63
37.60
37.56
37.53
37.43
32.94
32.92
28.11
24.96
24.94
24.60
22.86
22.77
21.21
20.80
19.90
19.83
13.26
12.43
12.05
5l
O
N
^{13}C{^{1}H}-NMR (101 MHz, $CDCl_3$)
200 190 180 170 160 150 140 130 120 110 100 90 80 70 60 50 40 30 20 10 0
f1 (ppm)

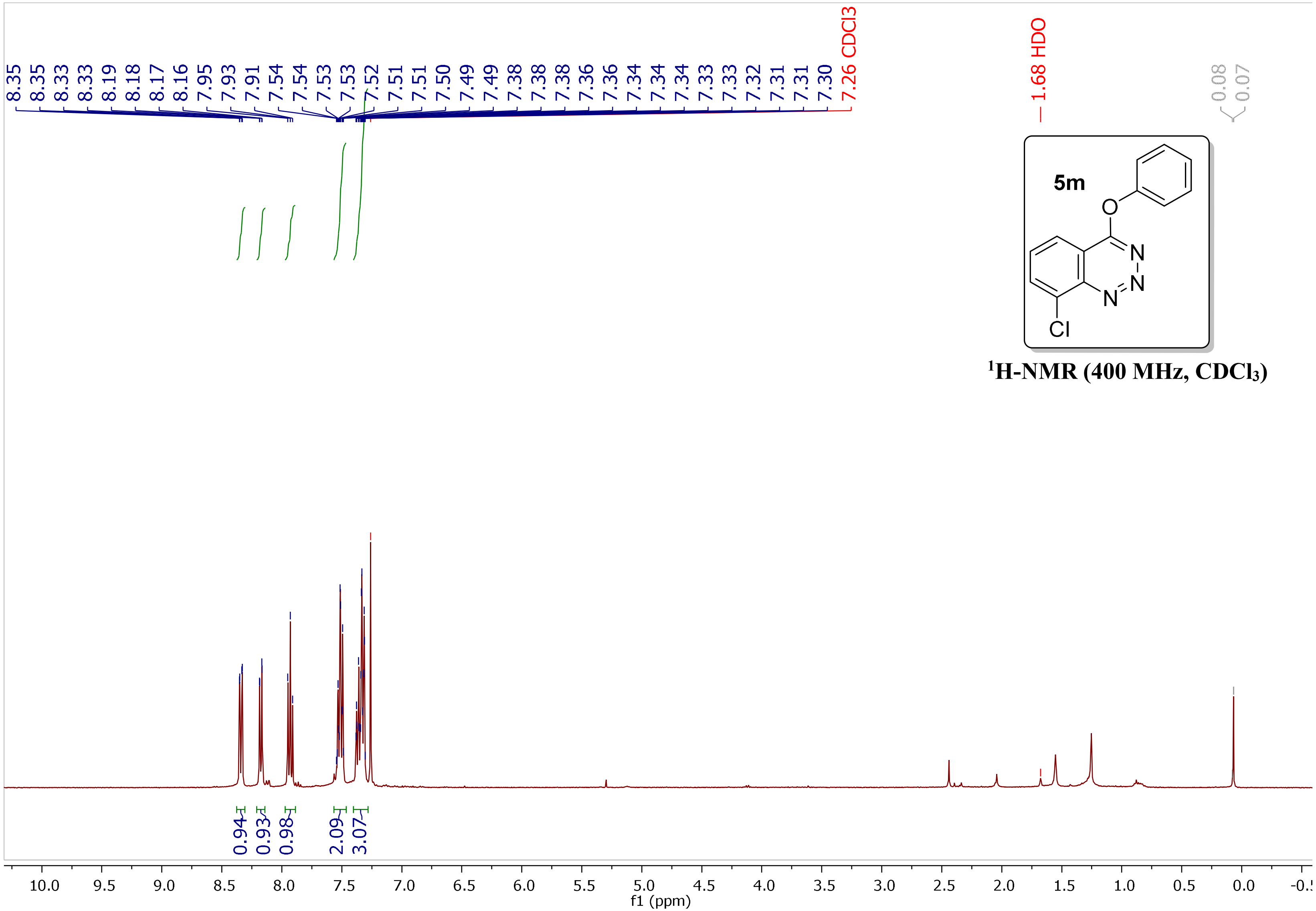

¹H-NMR (400 MHz, CDCl$_3$)

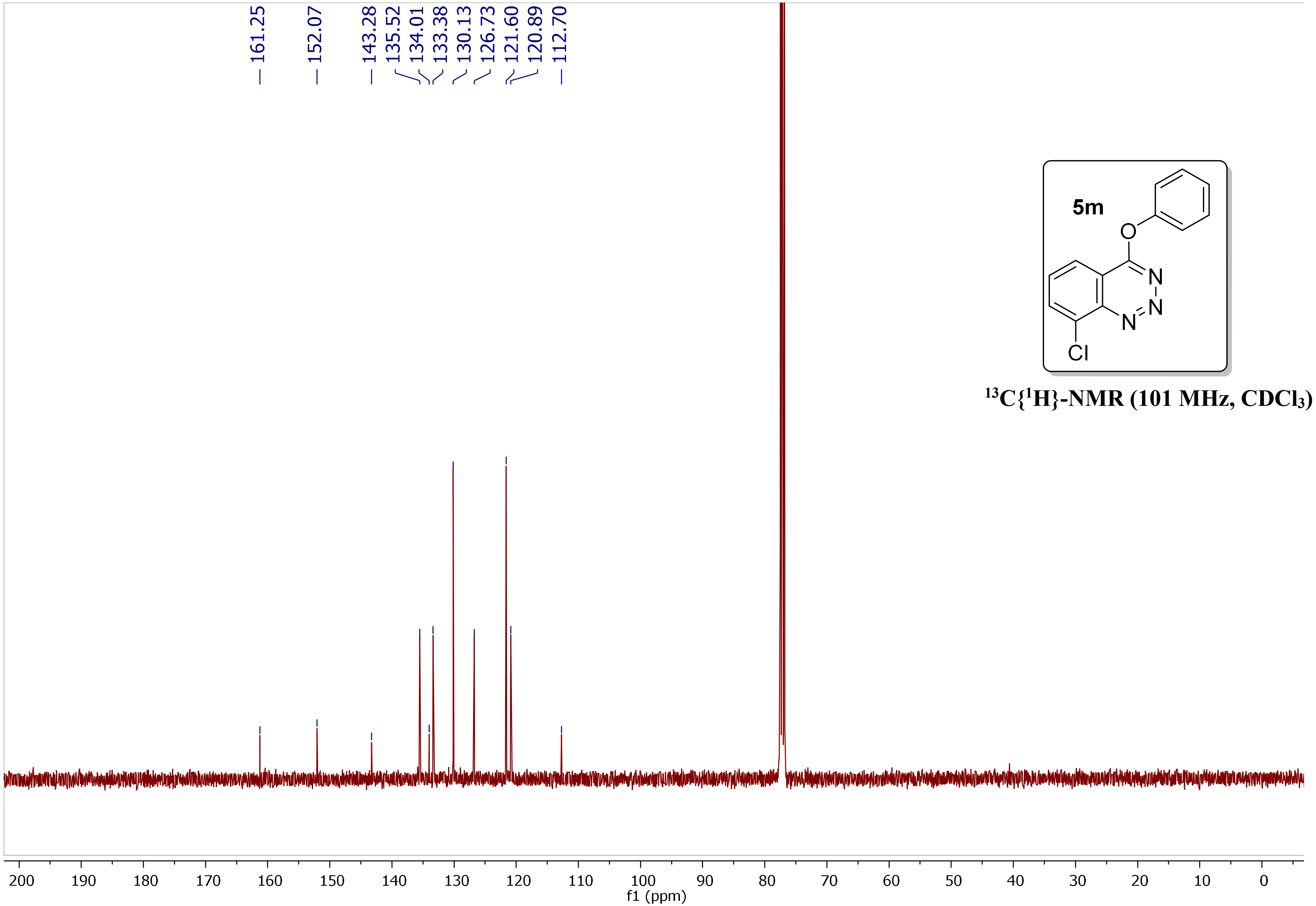

$^{13}C\{^{1}H\}$-NMR (101 MHz, $CDCl_3$)

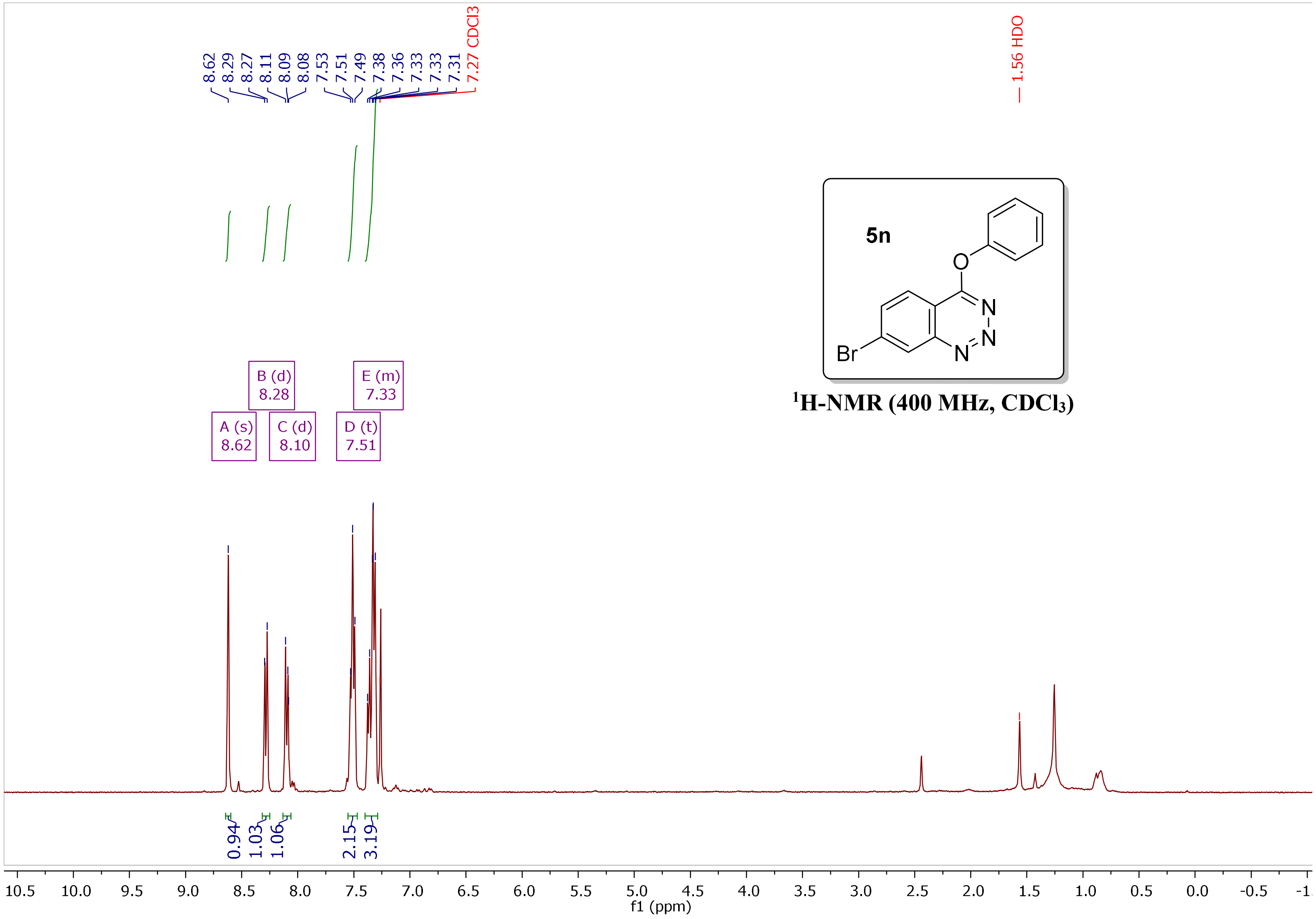

8.62
8.29
8.27
8.11
8.09
8.08
7.53
7.51
7.49
7.38
7.36
7.33
7.33
7.31
7.27 CDCl3
1.56 HDO
5n
Br
O
N
N
N
¹H-NMR (400 MHz, CDCl₃)
B (d) 8.28
E (m) 7.33
A (s) 8.62
C (d) 8.10
D (t) 7.51
0.94
1.03
1.06
2.15
3.19
10.5
10.0
9.5
9.0
8.5
8.0
7.5
7.0
6.5
6.0
5.5
5.0
4.5
4.0
3.5
3.0
2.5
2.0
1.5
1.0
0.5
0.0
-0.5
f1 (ppm)

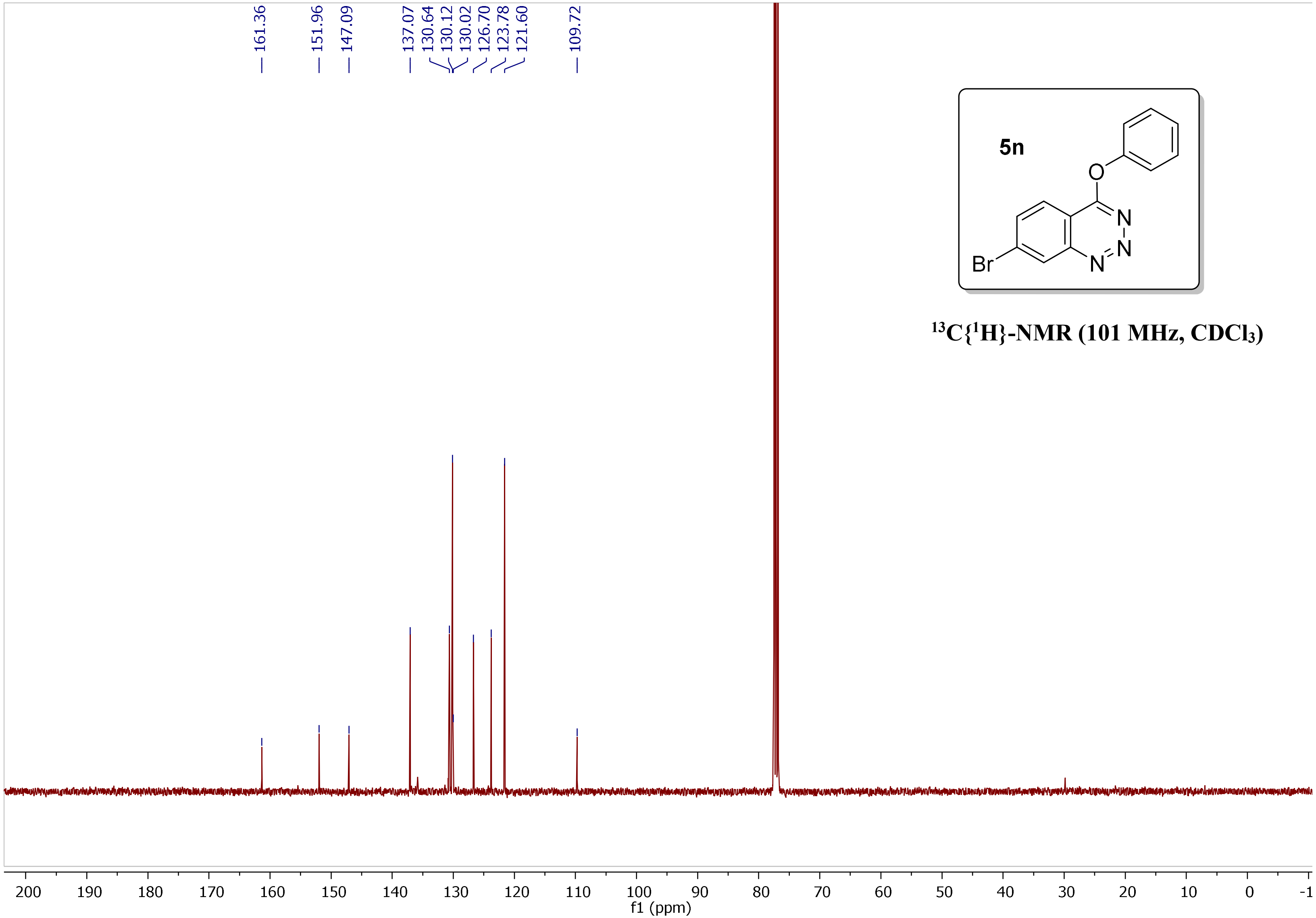

$^{13}C\{^{1}H\}$-NMR (101 MHz, $CDCl_3$)

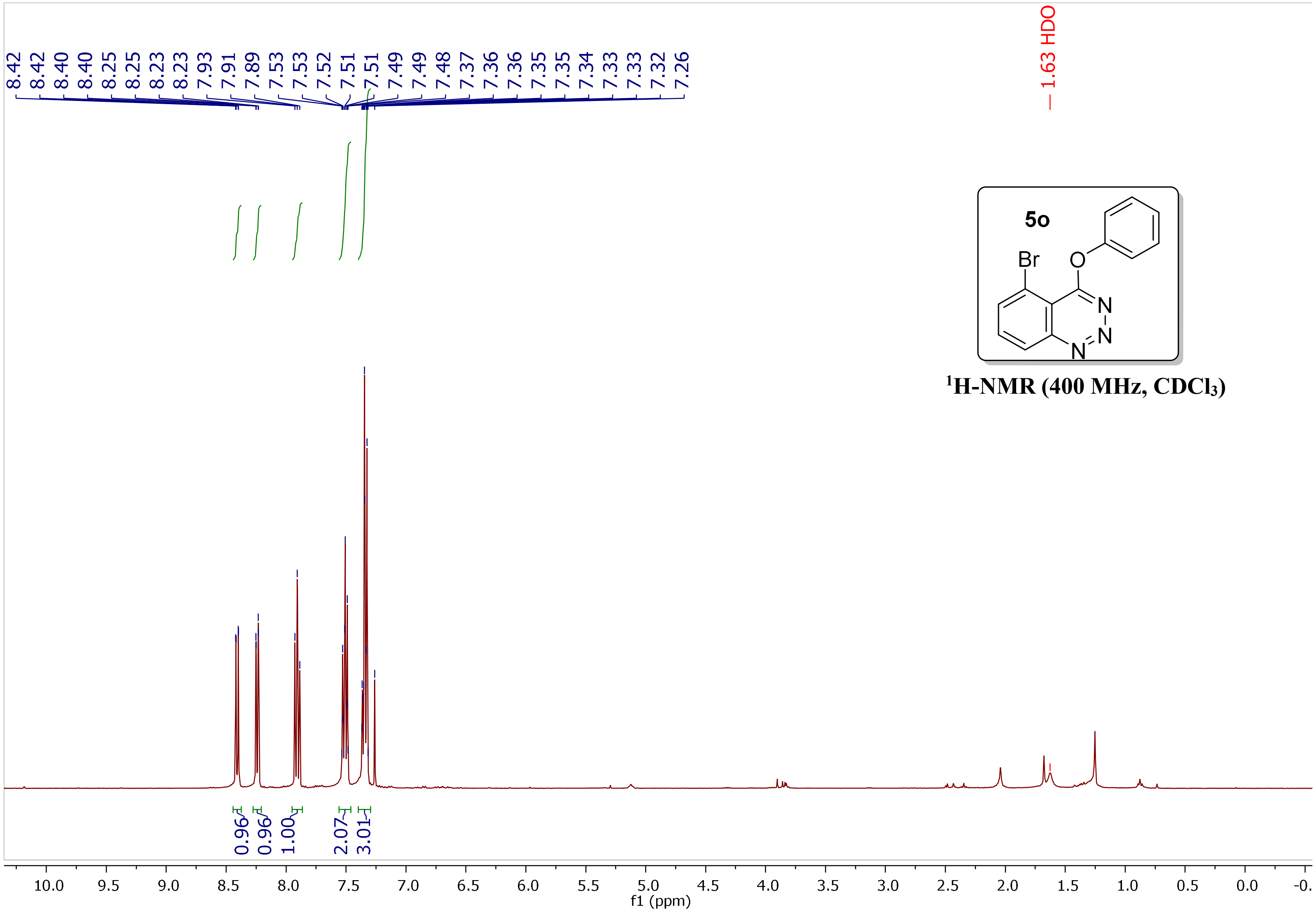
8.42
8.42
8.40
8.40
8.25
8.25
8.23
8.23
7.93
7.91
7.89
7.53
7.53
7.52
7.51
7.51
7.49
7.49
7.48
7.37
7.36
7.36
7.35
7.35
7.34
7.33
7.33
7.32
7.26
1.63 HDO
5o
Br
O
N
N
N
^{1}H-NMR (400 MHz, $CDCl_3$)
0.96
0.96
1.00
2.07
3.01
10.0 9.5 9.0 8.5 8.0 7.5 7.0 6.5 6.0 5.5 5.0 4.5 4.0 3.5 3.0 2.5 2.0 1.5 1.0 0.5 0.0 -0.
f1 (ppm)

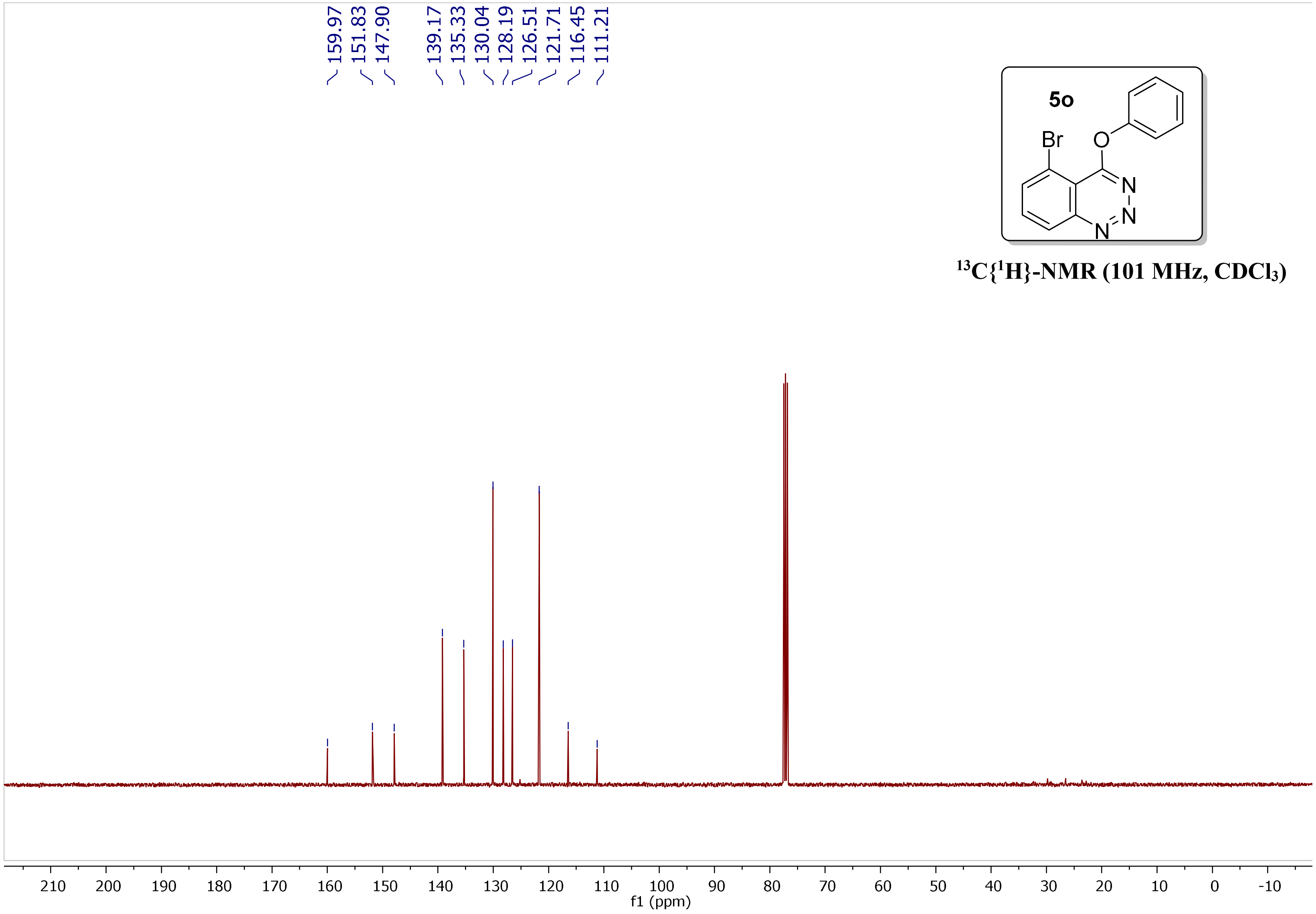
159.97
151.83
147.90
139.17
135.33
130.04
128.19
126.51
121.71
116.45
111.21
5o
Br
O
N
N
N
$^{13}C\{^1H\}$-NMR (101 MHz, $CDCl_3$)
f1 (ppm)

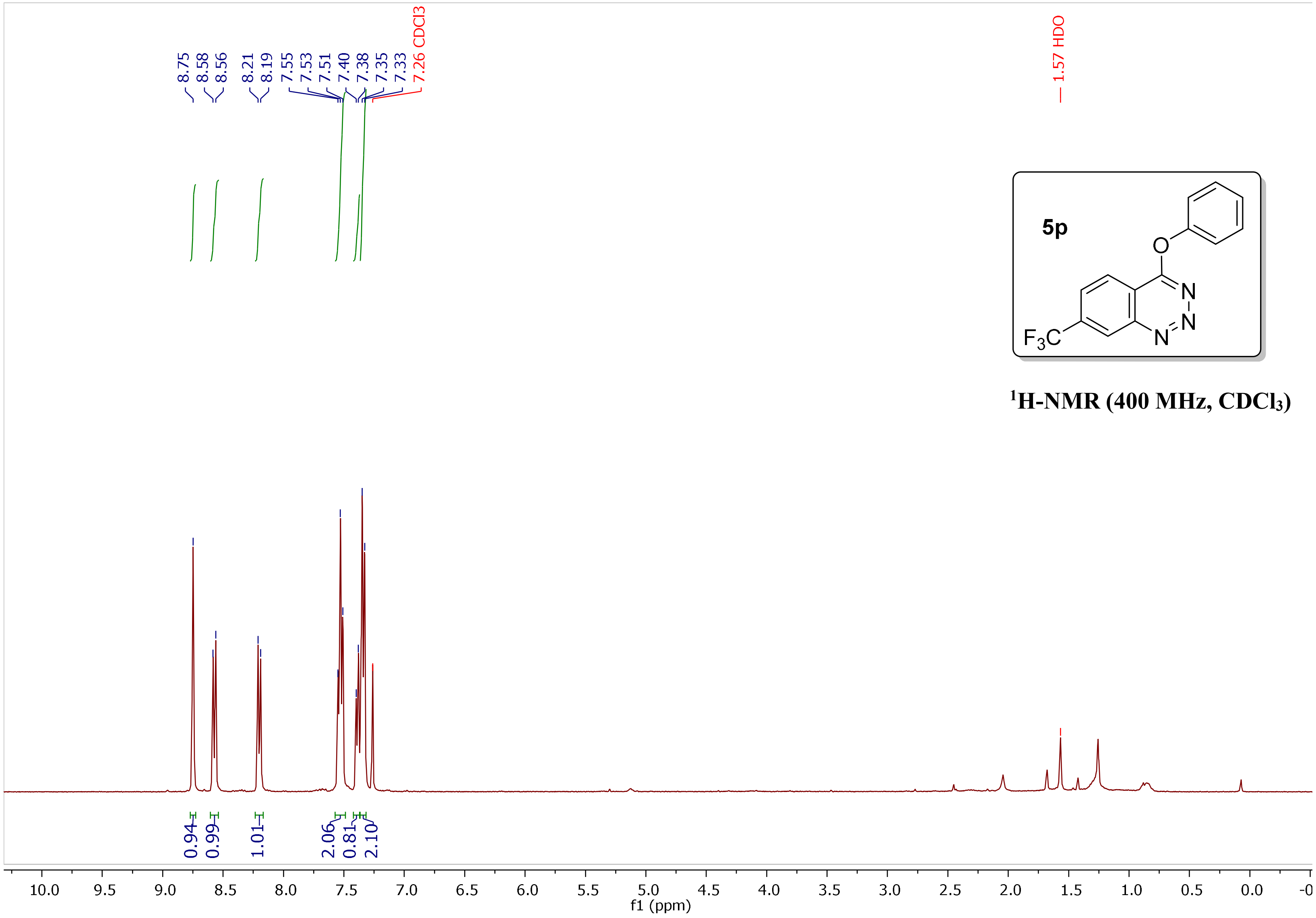
8.75
8.58
8.56
8.21
8.19
7.55
7.53
7.51
7.40
7.38
7.35
7.33
7.26 CDCl3
1.57 HDO
5p
O
N
N
N
F3C
¹H-NMR (400 MHz, CDCl3)
0.94
0.99
1.01
2.06
0.81
2.10
10.0
9.5
9.0
8.5
8.0
7.5
7.0
6.5
6.0
5.5
5.0
4.5
4.0
3.5
3.0
2.5
2.0
1.5
1.0
0.5
0.0
f1 (ppm)

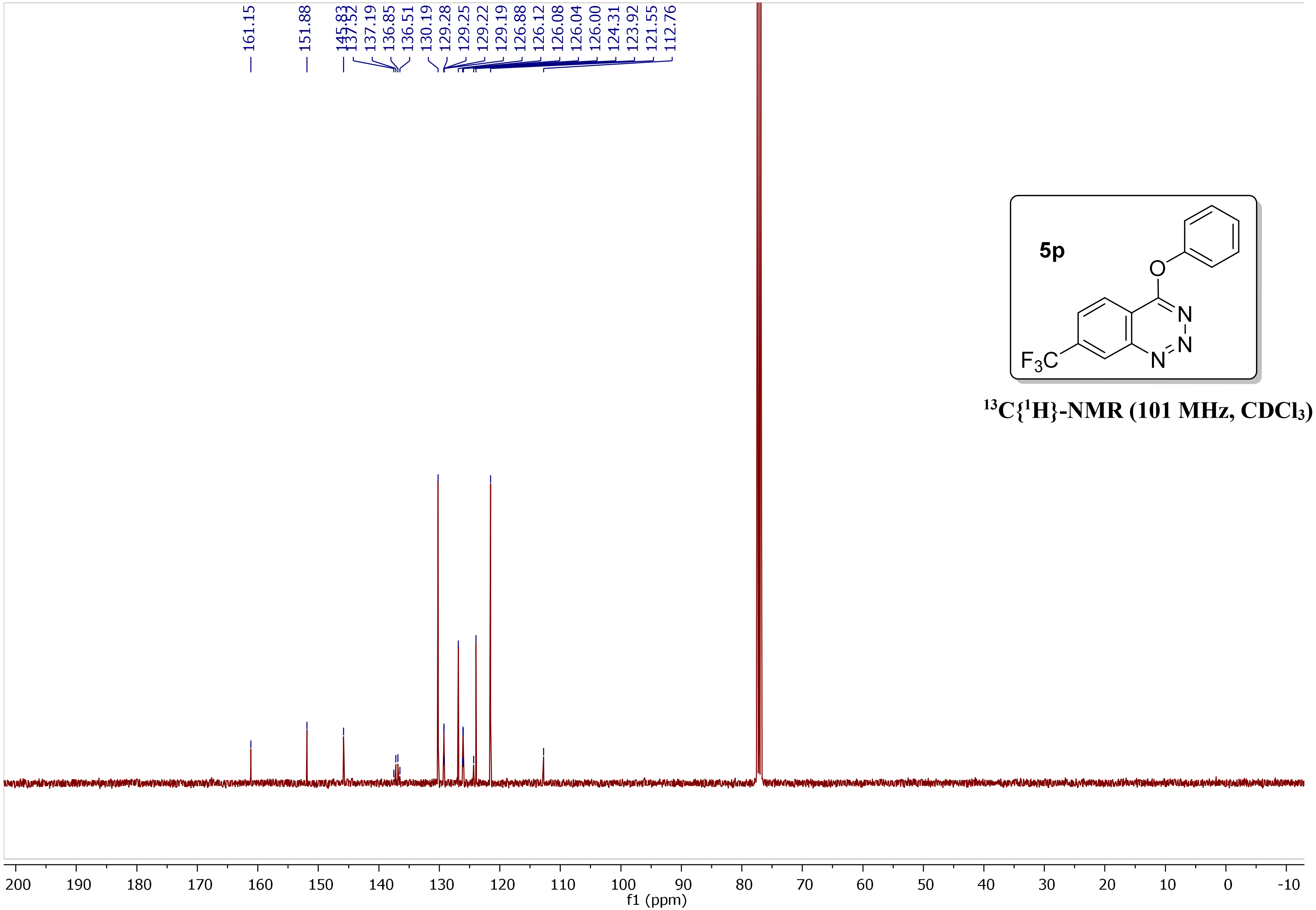

$^{13}C\{^{1}H\}$-NMR (101 MHz, $CDCl_3$)

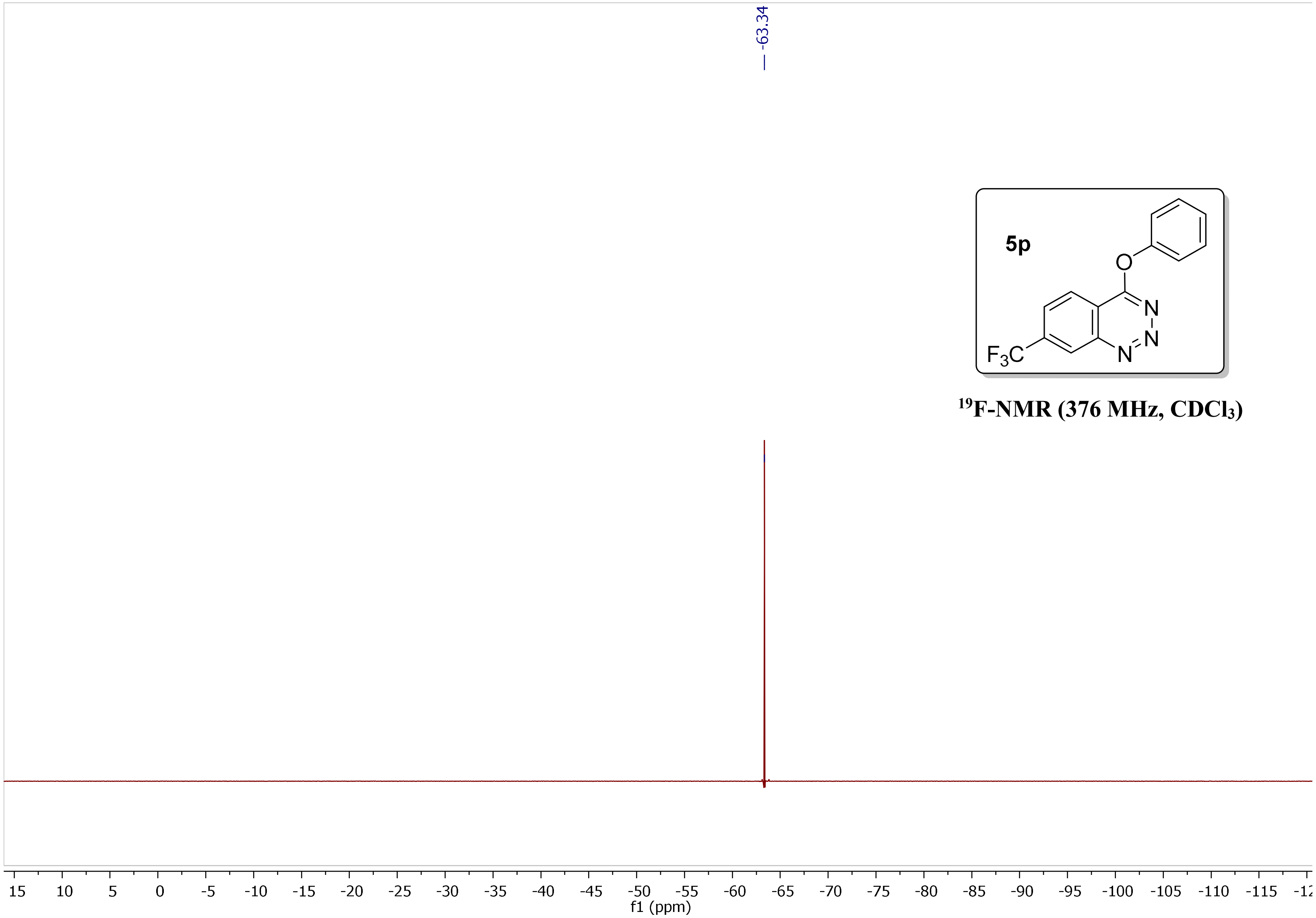

^{19}F-NMR (376 MHz, $CDCl_3$)
